# TfOH-Promoted Cascade Reaction of Nitriles and 1,5-Enynes: An Efficient Route to Amido-Functionalized Fluorenes

**DOI:** 10.1021/acs.joc.5c02263

**Published:** 2025-12-11

**Authors:** Babasaheb Sopan Gore, Wan-Jing Liu, Lin-Wei Pan, Chien-Hung Li, Jeh-Jeng Wang

**Affiliations:** † Department of Medicinal and Applied Chemistry, 38023 Kaohsiung Medical University, No. 100, Shih-Chuan first Rd, Sanmin District, Kaohsiung City 807, Taiwan; ‡ Department of Medical Research, Kaohsiung Medical University Hospital, Kaohsiung City 807, Taiwan

## Abstract

We have demonstrated a highly efficient synthetic strategy for assembling amide-functionalized fluorenes via cascade reactions between nitriles and 1,5-enynes in a one-pot assembly. This approach provides a simple, atom-economic, practical, and robust path to synthesize highly versatile rare carbocyclic scaffolds under transition-metal- and additive-free conditions with good reaction yields. Its most noteworthy features include high compatibility with various functionalities, mild conditions, easily accessible precursors, and gram-scale synthesis.

## Graphical abstract

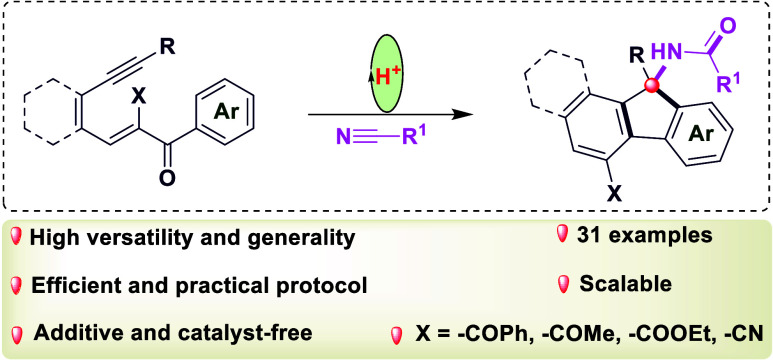

## Introduction

Polycyclic aromatic hydrocarbons (PAHs) are becoming increasingly popular in material science, pharmaceuticals, and high-end electronics due to their versatile structures, and they are also utilized in medicinal fields, fine chemicals, and natural products.^1^ In particular, fluorenes are among the most important compounds that play a vital role in synthetic intermediates and the further development of organic synthesis (Figure 1).^2^ They also exhibit excellent biological activity and have anticancer, antibacterial, antioxidant, and antidiabetic effects.^3^ Aside from their privileged position in naturally occurring and biologically active molecules, these structural motifs also possess unique physical and chemical properties.^4^ To date, various synthetic routes toward the construction of fluorenes have been developed, including transition-metal catalysis,^5^ radical cyclization,^6^ and Friedel–Crafts-type reaction^7^ (Scheme 1a). Despite these significant advances, previously reported methods often suffer from tedious multistep procedures and harsh reaction conditions. In addition, poor regioselectivity, limited functional group tolerance, and reliance on expensive metal catalysts restrict their broader applicability. Several transition metal-mediated cascade cyclization methods have also been developed for the synthesis of substituted fluorenes.^8^ For example, Hamze and co-workers reported the copper-catalyzed cross-coupling reaction of 2′-bromo-biaryl-*N*-tosylhydrazones with different amines, leading to 9*H*-fluoren-9-amine derivatives through the formation of intermolecular C–N and C–C bonds (Scheme 1b).^9a^ Meanwhile, Liang et al. demonstrated the silver-catalyzed oxidative cyclization of enynes with disubstituted phosphine oxides for the synthesis of fluorene derivatives (Scheme 1c).^9b^ Li et al. recently reported the catalytic bicyclization of conjugated enynes with arylsulfonyl radicals generated in situ from sulfonyl hydrazides using oxidants and copper catalysts under convenient conditions to construct tosylate-incorporating fluorene analogs (Scheme 1d).^9c^ As a result, the development of a practical, efficient, metal-free synthetic strategy for the synthesis of such challenging myriad scaffolds is urgently demanded. On the other hand, amides represent a significant class of compounds that have been widely utilized in various fields. They serve as catalysts, reagents, and substrates in synthetic chemistry and constitute the key structural backbone of peptides, proteins, and numerous other biomolecules.^10^ Furthermore, amide bonds are abundant motifs in pharmacologically active compounds, including approved drugs and materials.^11^ The most efficient and highly utilized methods for their synthesis are based on the condensation of carboxylic acids (their derivatives) and amines under various coupling reagents and catalysis.^12^ Therefore, it is also crucial to introduce the amide group into organic skeletons, such as complex molecules, which could enhance their synthetic diversity or biological properties. However, the synthesis of carbocyclic skeletons containing amide functionality, especially under metal-/metal-free conditions, is highly imperative. Furthermore, enynes are readily accessible building blocks and versatile backbones and are crucial precursors for cascade processes in synthetic chemistry.^13^ Therefore, several synthetic procedures have been reported to build complex skeletons through domino cascade reactions.^14^ An intramolecular direct functionalization of enynes in the absence of transition metals could be an ideal route for the fluorene synthesis. An extensive literature survey revealed that the nucleophilic-triggered annulation of enynes for the practical synthesis of amido-functionalized fluorenes remains elusive to date.

**Figure 1:**
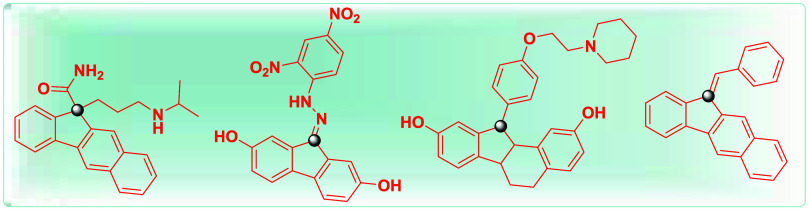
Selected bioactive products bearing a fluorene skeleton.

**Scheme 1:**
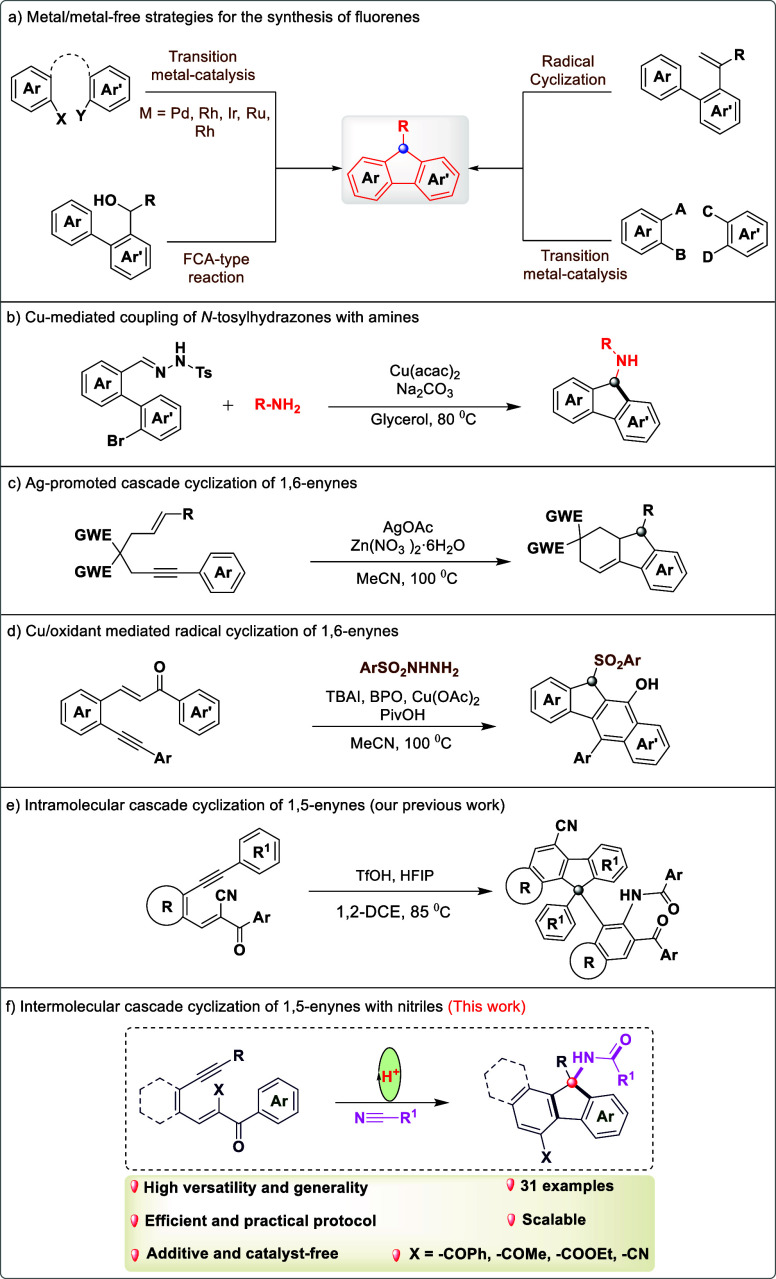
Synthetic Design for Fluorenes and Nitrile Functionalization

## Results and Discussion

Considering these facts, we recently reported an HFIP-mediated, acid-catalyzed, regioselective functionalization of enynes containing nitrile groups to construct naphthamide-substituted fluorenes under metal-free conditions via nitrile activation (Scheme 1e).^15a^ Therefore, based on our continuing interest in the transformation of enynes and nitriles,^15b–15f^ we have developed a one-pot, acid-catalyzed synthesis of amido-functionalized fluorene scaffolds (Scheme 1f). This transformation proceeds with a high atom economy under external oxidant- and transition-metal-free conditions. It should be noted that direct access to amide-incorporated fluorenes is unknown and more challenging than the synthesis of other types of fluorenes. Therefore, this is an emerging area of research that also provides rapid access to new chemical spaces. We commenced our study by optimizing the reaction conditions with conjugated 1,5-enynes­(**1a**) and acetonitrile (**2a**) as model substrates. Initially, the feasibility of the hypothesis was confirmed by product **3aa**, which was obtained in 45% yield when the reaction was conducted in the presence of TfOH as a proton source (0.5 equiv) at 90 °C for 4 h in 2.0 mL of acetonitrile solvent (Table 1, entry 1). Encouraged by this observation, further studies on other reaction parameters were carried out. Subsequent experiments (Table 1, entries 2–6) eventually revealed that 1.0 equiv of TfOH and 4 h gave **3aa** in an 86% reaction yield (Table 1, entry 2). Other proton sources such as TFA, AcOH, PivOH, and TsOH resulted in low yields when evaluated in this process (Table 1, entries 3–6). Next, the effect of solvents was also investigated by using CH_3_CN (2.0 equiv) with 1,4-dioxane and 1,2-dichloroethane (entries 7–8). However, none of them gave a better yield than that of entry 2. Additionally, the product yield decreased when the reaction was conducted for a prolonged period (Table 1, entry 9). The reaction also failed to improve the yield of compound **3aa** when the reaction temperature was decreased, or the proton source was increased to 2.0 equiv. (Table 1, entries 10–11). Finally, no reaction occurred without TfOH (Table 1, entry 12), which indicated that the proton source is crucial to the success of the reaction. Thus, the reaction conditions mentioned in Table 1, entry 2, were chosen as the optimum conditions. As shown in Table 1. The scope and limitations of this method were investigated by systematically varying substrate **1** with acetonitrile **2a** under standard conditions. To enhance the utility of the new method in the synthesis of diverse amido-fused fluorenes, a series of (*E*)-2-aroyl-3-(2-(aryl/alkylalkynes)­aryl/alkyl)­acryloketone/esters/nitriles (**1**) bearing different groups were evaluated to investigate the effect of the substituent on the yield of the cascade cyclization of conjugate enynes with nitriles. As expected, compounds with electron-rich (dimethoxy, methoxy, methyl, ethyl) and electron-deficient (trifluoromethyl, fluoro, chloro, cyano, ester) groups on the aryl ring performed well, with the formation of the corresponding amido-substituted fluorene products in average to good yields and with complete regioselectivity (Scheme 2, **3ba–f**, **g**, **h**, **i**, **q**, **r**, and **s**). Pyridine-incorporated enynes gave the corresponding amido-fluorene product **3ja** in 46% yield. Alternatively, a straight-chain alkyl substituent linked to the carbon–carbon triple bond was also well-tolerated, affording product **3ka** in a 42% chemical yield. In addition, an array of aliphatic compounds also reacted successfully (Scheme 2, **3la**). The substrate scope of other functionalities was also explored in this two-component reaction, as shown in Scheme 2. Various groups, such as methyl ketones, esters, and nitriles, are subjected to a two-component reaction, and amido fluorenes are produced in moderate to good yields, ranging from 52% to 92%, as shown in Scheme 2 (**3ma**, **3na**, **3oa**, and **3pa**). However, 1,5-enynes containing hydrogen or methyl groups did not yield the desired products (**3ta** and **3ua**). In view of our successful synthesis of functional amido-fluorene derivatives **3**, next, we turned our attention to the scope of nitrile (**2**) with the respective enyne substrates, which would expand their utility for the synthesis of substituted fluorenes (Scheme 3). To our delight, the reaction with other nitriles such as benzonitrile and *p*-tolunitrile proceeded smoothly (Scheme 3, **4ab**–**ac**, and **4nb**). It should be noted that the reactions of butyronitrile (**2d**), isopropyl nitrile (**2e**), propionitrile (**2f**) and cyclohexyl nitrile (**2g**), benzyl nitriles (**2j**), and phenyl bearing butyronitrile (**2k**) functionalities showed similar reactivity in this reaction system, affording the desired products **4ad**–**4ag** and **4nj**–**4nk** and **4of** in 69% to 81% yields, respectively (Scheme 3). The replacement of aromatic/aliphatic nitriles with acrylonitrile (**2h**) and cinnamonitrile (**2i**) smoothly led to the corresponding products (**4ah**-**4ai**) in good yields. However, bromoacetonitrile (**2l**) was inert and failed to react with **1a** to produce product **4ol**. Additionally, the exact structure of compounds (**4nb** and **4of**) was unambiguously confirmed by X-crystallographic analysis.^16^ To demonstrate the practicality of the developed intermolecular nitrile addition to enynes, we synthesized compound **3aa** on a gram scale while maintaining good reaction yields (Scheme 4a). We then successfully achieved the benzylation of **3aa**, proceeding through *N*-alkylation to access the corresponding products **6aa** in 79% yield (Scheme 4b). To shed light on the reaction mechanism, several control experiments were carried out (Scheme 4). Initially, an isotope labeling experiment was performed. In deuterated acetonitrile (**2a**-***d***_***3***_) (Scheme 4c), the methyl group of the amide was completely deuterated, suggesting that the amide is derived from the nitrile. Further, when acetamide **7a** was used instead of acetonitrile **2a**, the desired product was not produced, indicating that nitrile was not hydrolyzed to the amide under acidic conditions before the reaction with conjugated enyne **1a** (Scheme 4d). Therefore, the obtained results clearly demonstrated that the nitrile was hydrolyzed in the reaction. Based on the above experimental results and previous works,^17^ a plausible mechanism for the cascade cyclization of enynes with nitriles is depicted in Scheme 5. Protonolysis of **1** to give intermediate **A** generates developing positive charges that are stabilized by the electron-withdrawing groups.

**Table 1:**
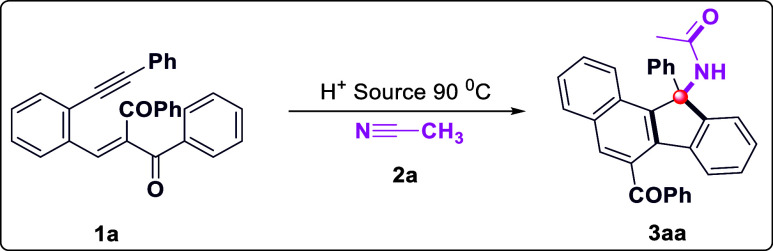
Optimization of the Reaction Conditions^a^

entry	H^+^ source (equiv)	solvent	time, h	yield (%)^b^
1	TfOH (0.5)	neat	4	45
**2**	TfOH (1.0)	neat	4	86
3	TFA (1.0)	neat	4	67
4	AcOH (1.0)	neat	4	27
5	PivOH (1.0)	neat	4	0
6	TsOH (1.0)	neat	4	trace
7^c^	TfOH (1.0)	1,4- dioxane	4	trace
8^d^	TfOH (1.0)	1,2-DCE	4	25
9	TfOH (1.0)	neat	6	80
10^e^	TfOH (1.0)	neat	4	61
11	TfOH (2.0)	neat	4	74
12		neat	4	0

a Reaction conditions: **1a** (0.15 mmol), neat represents acetonitrile (**2a**, 2.0 mL of reagent and solvent), 90 °C (oil bath) in a sealed tube.

b Isolated yields.

c 2.0 equiv of acetonitrile and 1,4-dioxane (2.0 mL) were used.

d 2.0 equiv of acetonitrile and 1,2-DCE (2.0 mL) were used.

e The reaction mixture was stirred at 60 °C. TfOH = trifluoromethanesulfonic acid, TFA = trifluoroacetic acid, AcOH = acetic acid, PivOH = pivalic acid, TsOH = *p*-toluenesulfonic acid.

**Scheme 2:**
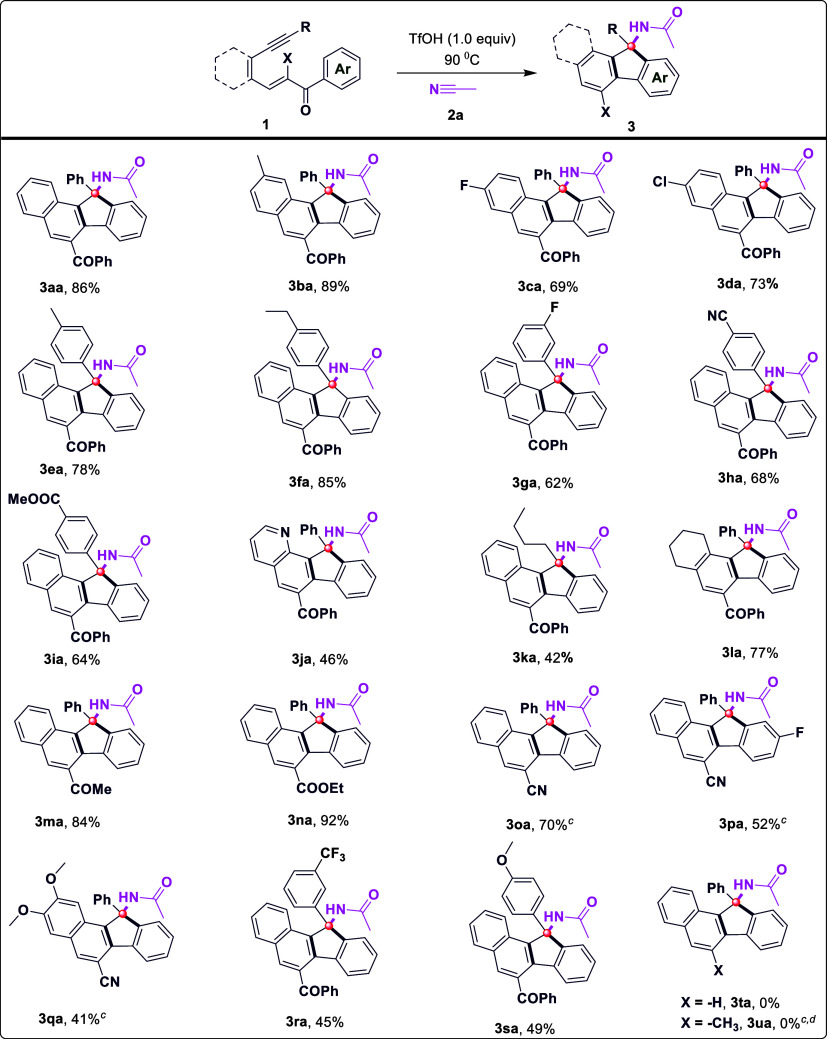
Substrate Scope of Enynes^a^,^b^,^c^,^d^

a Reaction conditions: **1** (0.15 mmol), TfOH (1.0 equiv), acetonitrile (**2a**, 2.0 mL, reagent and solvent), 90 °C, 4 h (oil bath) in a sealed tube.
b Isolated yields.
c TfOH (3.0 equiv) and the reaction mixture was stirred for 24 h.
d Starting material was recovered.

**Scheme 3:**
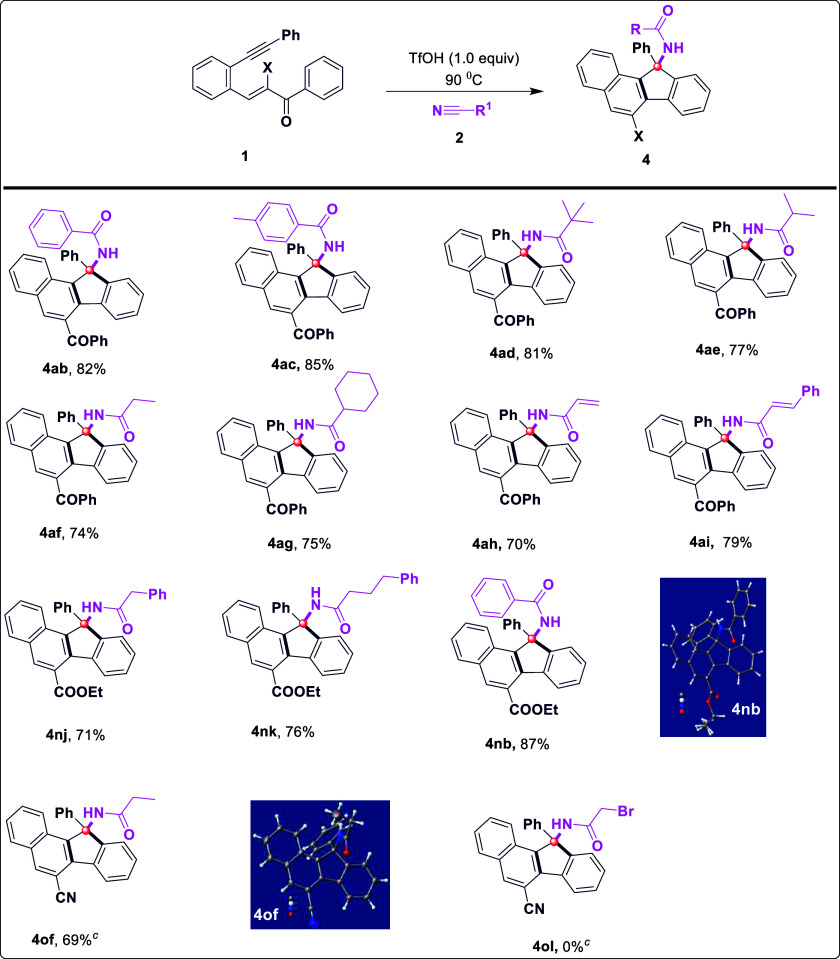
Substrate Scope with Respect to Nitriles^a^,^b^,^c^

a All reactions were carried out using **1** (0.15 mmol), TfOH (1.0 equiv), nitriles (**2**, 2.0 mL, reagent and solvent), 90 °C, 4 h (oil bath) in a sealed tube.
b Isolated yields.
c TfOH (3.0 equiv) and the reaction mixture was stirred for 24 h.

**Scheme 4:**
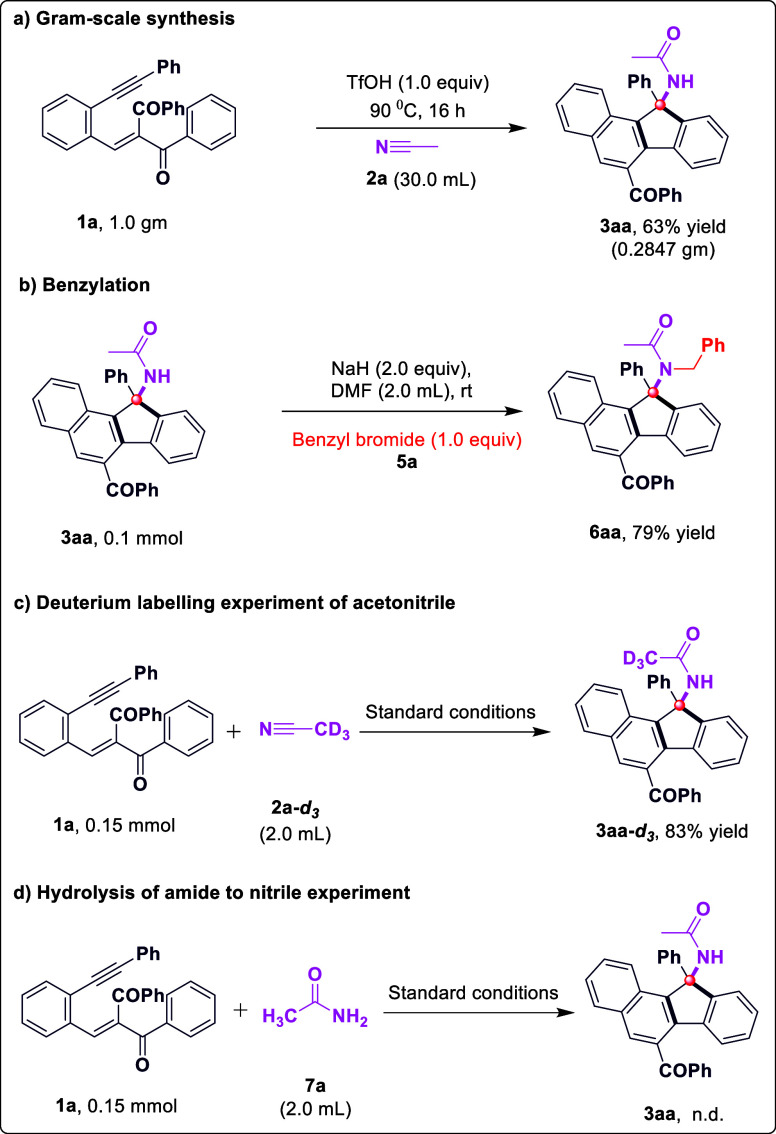
Synthetic Applications and Control Experiments

**Scheme 5:**
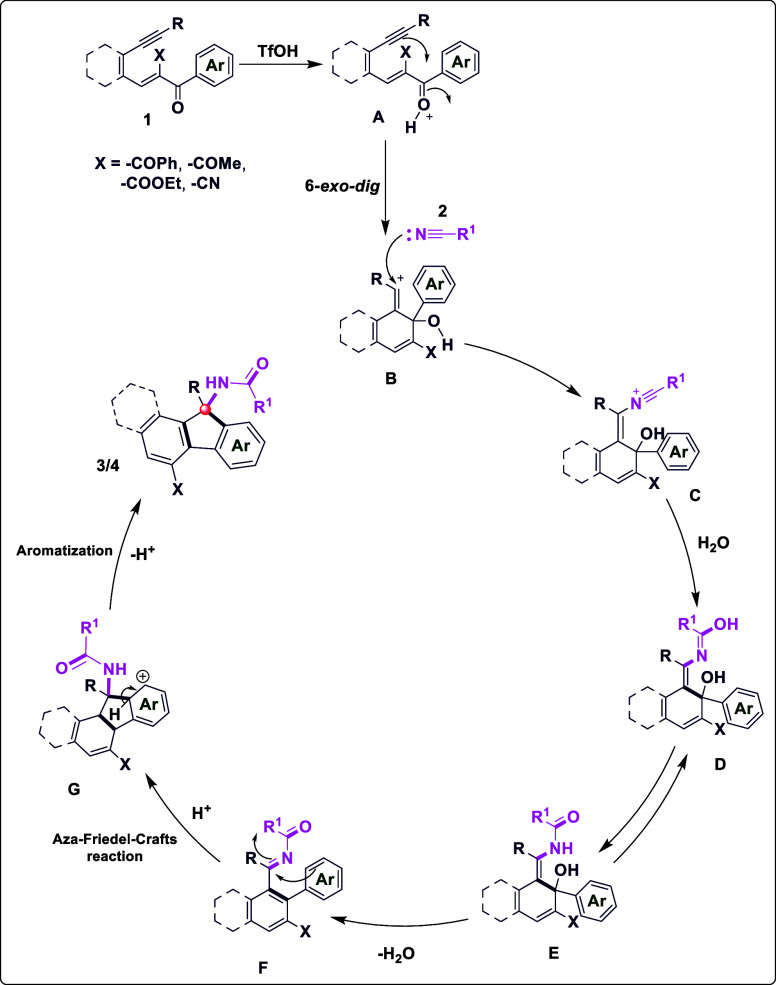
Proposed Mechanistic Rationales

This lowers the barrier for the subsequent 6-*exo-dig* cyclization to the vinylic cation **B**. The enhanced stabilization and increased electrophilicity of **B** ^15d^ promote efficient intermolecular nucleophilic attack by the nitrile, forming adduct **C**. This adduct then undergoes transformation via oxime intermediate **D** to generate intermediate **E**. Furthermore, the strong electron-withdrawing effect facilitates subsequent dehydration to afford intermediate **F**. Finally, under the acidic conditions used, the EWG lowers the energy of the transition state for the intramolecular Aza–Friedel–Crafts-type cyclization and helps drive the final aromatization step, thus enhancing both the yield and selectivity for observed products **3** and **4**.

## Conclusions

In conclusion, we have demonstrated a cascade cyclization reaction of 1,5-enynes with nitriles using a metal-free strategy in a one-pot assembly. This method provides an important alternative approach with broad implications for the synthesis of functionalized amido-substituted fluorene with good yields. This mild method provides easy access to constructing valuable skeletons with high atom economy and chemoselectivity in organic and medicinal chemistry, as they are difficult to synthesize using conventional approaches. We are currently developing more efficient chiral catalysts and expanding the synthesis of such complex architectures.

## Supplementary Material



## Data Availability

The data underlying this study are available in the published article and its online Supporting Information.

## Notes

Experimental data, spectral characterization, solvent system, and methods for crystal growth of **4nb** and **4of**, and X-ray crystal data; copies of ^1^H, ^13^C NMR spectra for all products; HRMS data (PDF)
