# Supplementary material for: TfOH-Promoted Cascade Reaction of Nitriles and 1,5-Enynes: An Efficient Route to Amido-Functionalized Fluorenes
Source: J Org Chem. 2025 Dec 11;90(51):18217–23. doi: 10.1021/acs.joc.5c02263 (PMC12751013; doi:10.1021/acs.joc.5c02263)

Supporting Information

TfOH-Promoted Cascade Reaction of Nitriles and 1,5-Enynes: An Efficient Route to Amido-Functionalized Fluorenes

Babasaheb Sopan Gore†, Wan-Jing Liu†, Lin-Wei Pan,† Chien-Hung Li† and Jeh-Jeng Wang†‡*

†Department of Medicinal and Applied Chemistry, Kaohsiung Medical University, No. 100, Shih-Chuan 1st Rd, Sanmin District, Kaohsiung City, 807 (Taiwan).

‡Department of Medical Research, Kaohsiung Medical University Hospital, No. 100, Tzyou 1st Rd, Sanmin District, Kaohsiung City, 807 (Taiwan). E-mail: jjwang@kmu.edu.tw

1. Table of contents

1. Table of contents S2

2. Materials and methods..S3

3. Experimental data..S5

3.1 General Procedure for the Synthesis of substrate **1**..S6

3.2 General procedure for the synthesis of final compounds **3** and **4**..S7

3.3 Gram scale reaction for the synthesis of compound **3aa**..S7

4. Spectral characterization S8

5. Solvent system and method for crystal growth of **4nb**, and **4of**

(Ellipsoid contour % probability levels) S23

6. Reference..S24

7. Copies of ^{1}H, and ^{13}C S25

8. X-ray crystal data..S121

2. Materials and methods

All air- and moisture-insensitive reactions were carried out under ambient atmosphere and monitored by thin layer chromatography (TLC). Concentration under reduced pressure was performed by rotary evaporation at 35-45 °C at an appropriate pressure. Purified compounds were further dried under high vacuum. Yields refer to purified and spectroscopically pure compounds, unless otherwise stated. Mass spectra and High-Resolution Mass spectral (HRMS) data was carried out using an Agilent6890N GC (JEOL JMS-700) TOF instrument, and the ion source is electrospray ionization (ESI), electronic ionization (EI), CI, and FAB as ion source at National Taiwan Normal University, Taipei City, Taiwan and ESI-TOF(FT-MS solariX) at National Sun Yat-Sen University, Kaohsiung, Taiwan, and LTQ Orbitrap XL (Thermo Fischer Scientific) at National Chung Hsing University. Liquid-chromatography mass spectra (LCMS) were measured using the LC-MS/MS-8045 (Shimadzu Corporation, Japan) at Kaohsiung Medical University, Kaohsiung, Taiwan. Melting points were determined on an EZ-Melt (Automated melting point apparatus).

Solvents

Bottle grade CAN, Dioxane, DCE were purchased from Alfa acer. Anhydrous DCM was obtained from Phoenix Solvent Drying Systems. All deuterated solvents were purchased from Sigma-Aldrich.

Chromatography

Thin layer chromatography (TLC) was performed using Merck TLC aluminum sheets (silica gel 60 F254) and visualized by fluorescence quenching under UV light. Flash column chromatography was performed using silica gel (40-63 µm particle size) purchased from Merck.

Spectroscopy and Instruments

^{1}H, ^{13}C, and DEPT NMR spectra were recorded on a 400 MHz Varian Unity Plus or Varian Mercury plus spectrometer or JEOL ECS-400. The chemical shift (δ) values are reported in ppm, and the coupling constants (J) are given in Hz. For ^{1}H NMR: $CDCl_3$, δ 7.26; CD_3CN, δ 1.96; CD_2Cl_2, δ 5.32; $(CD_3)_2CO$, δ 2.50; For ^{13}C NMR: $CDCl_3$, δ 77.16; 1.32; CD_2Cl_2, δ 53.84; $(CD_3)_2SO$, δ 39.52. The abbreviations used are as follows: s = singlet, d = doublet, t = triplet, q = quartet, dd = doublet of doublet, ddd = doublet of doublet of doublet, dt = doublet of triplets, td = triplet of doublet, m = multiplet, br = broad; coupling constants in Hz; integration.

Starting materials

All substrates were used as received from commercial suppliers, or prepared according to published procedures,[1] respectively, unless otherwise stated. TfOH, was purchased from Alfa Aesar, and stored in an -5 ^{0}C at refrigerator. Aldehydes, Alkynes, nitriles were purchased from Sigma-Aldrich, Acros, TCI, or Alfa Aesar.

3. Experimental data

The following 1,5-enynes substrates (**1a** - **1u**) were synthesized and utilized for further transformation under designed reaction conditions. Their preparation and characterization data of are as follows:

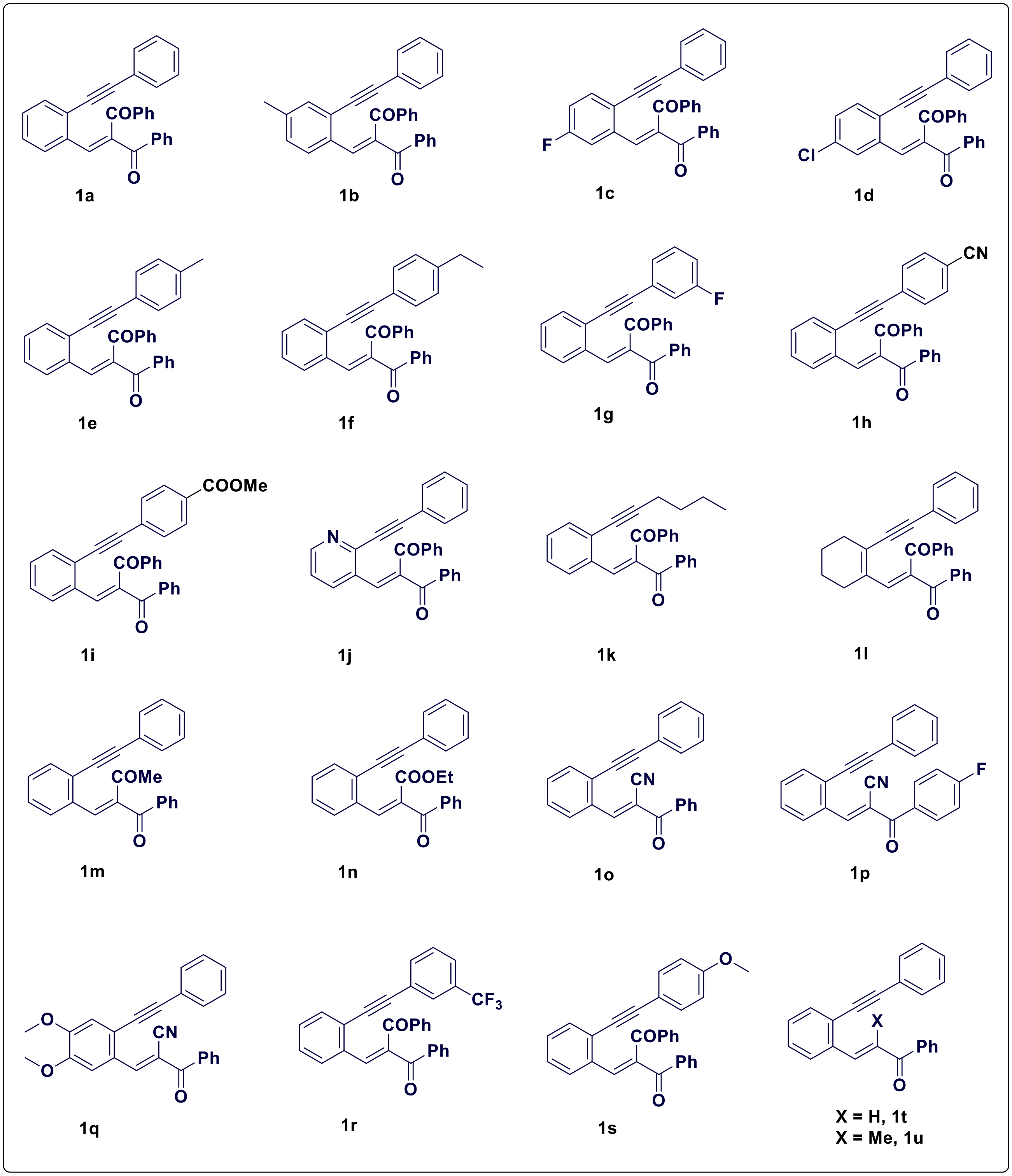

3.1 General Procedure for the Synthesis of substrate 1

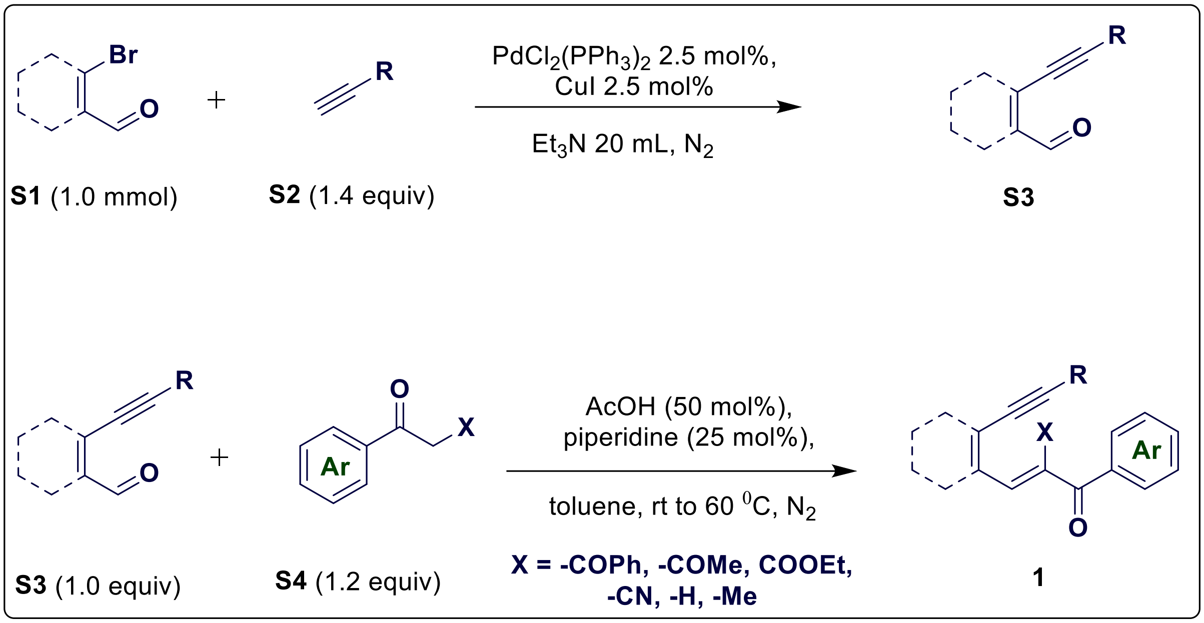

Step 1: To an oven dried round bottom flask, 2-bromobenzaldehyde/2-bromocyclohex-1-ene-1-carbaldehyde **S1** (1.0 mmol, 1.0 equiv), and $Pd(PPh_3)_2Cl_2$ (2.5 mol%) and CuI (2.5 mol%), in triethyl amine (20 mL) were added. The RB flask was then sealed and flushed with nitrogen. Then, alkyne **S2** (1.2 equiv) was added to the reaction mixture. Afterwards the reaction was stirred at 60 °C until TLC revealed complete conversion of the starting material. After completion of the reaction, the reaction mixture was allowed to cool. Then, organic layer was washed with aqueous saturated ammonium chloride solution, and finally extracted with EtOAc and water (3 × 10 mL). The combined organic layers were dried over Na_2SO_4, concentrated under vacuum. The crude material obtained was purified by column chromatography on silica gel (100–200 mesh) (hexane: ethyl acetate; 98/02) to afford the corresponding product **S3**.

Step 2: A reaction tube was charged with 1,3-diphenylpropane-1,3-dione/3-oxo-3-arylpropanenitrile/ester/methyl/ketone/acetophenone derivatives (**S4**) (0.75 mmol, 1.5 equiv), 2-(aryl/alkyl-(aryl/alkyl)-alkynyl) benzaldehyde (**S3**) (0.5 mmol, 1.0 equiv.), acetic acid (50 mol%) and piperidine (25 mol%) in 3.0 mL of toluene. The reaction suspension was stirred at room temperature to 60 °C and depending upon the reaction progress which was monitored by TLC. Upon completion water was added to quench the reaction mixture and then extracted with the ethyl acetate (3 × 30 mL). Finally combined organic layer was dried over sodium sulphate, filtered and concentrated in vacuum. The residue was purified by column chromatography on silica gel to afford the corresponding **1a-1u** derivatives.

3.2 General procedure for the synthesis of final compounds (3/4)

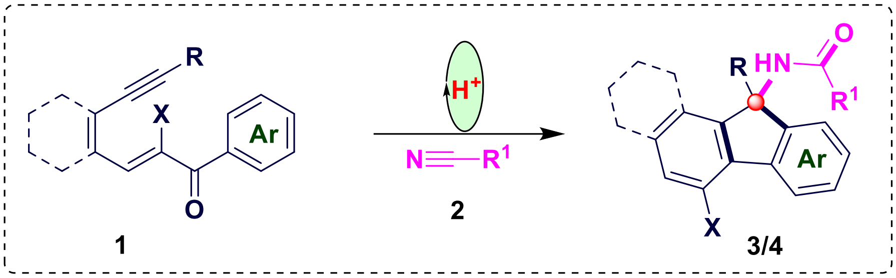

A clean Schlenk tube vial (15 mL) equipped with a magnetic stir bar was added to **1** (0.15 mmol, 1.0 equiv.), and 2.0 mL of respective nitriles (**2**). Next, TfOH (1.0 equiv) was added at room temperature, and then solution was heated 90 ^{0}C for required time. The progress of the reaction was monitored by thin layer chromatography. When the reaction was complete, water was added to quench the reaction mixture, followed by extraction with ethyl acetate (3×10 mL). Finally, the combined organic layer was dried over sodium sulfate, filtered, and concentrated under vacuum. The residue was purified by column chromatography (Hex/EA = 60:40) on silica gel to afford the corresponding **3/4** derivatives.

3.3 Gram scale reaction for the synthesis (3aa)

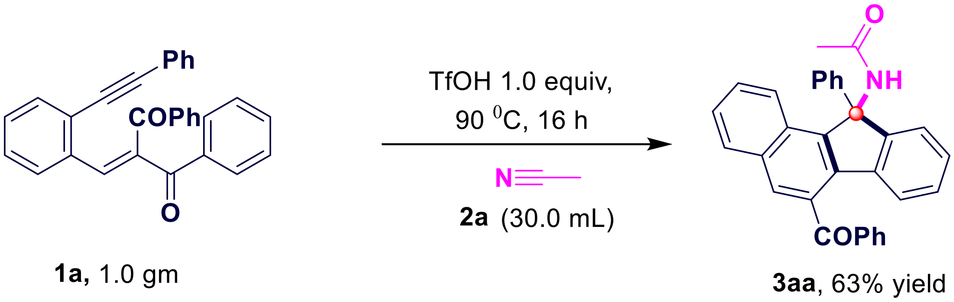

A clean flame-dried round bottom flask vial (100 mL) equipped with a magnetic stir bar was added to **1a** (1.0 gm, 2.4271 mmol, 1.0 equiv.), and 30.0 mL of acetonitrile (**2a**). Next, TfOH (214.16 µmL, 1.0 equiv) was added at room temperature, and then solution was heated 90 ^{0}C for 16 hours. The progress of the reaction was monitored by thin layer chromatography. When the reaction was complete, water was added to quench the reaction mixture, followed by extraction with ethyl acetate (3×10 mL). Finally, the combined organic layer was dried over sodium sulfate, filtered, and concentrated under vacuum. The residue was purified by column chromatography (Hex/EA = 60:40) on silica gel to afford the corresponding **3aa** derivative in (0.2847 gm) 63% yield.

4. Characterization Data for Key Compounds

The 1,5-enynes **1a, 1c, 1m, 1n, 1o, 1p, 1q** and **1t** were known products.[1]

1,3-diphenyl-2-(2-(phenylethynyl)benzylidene)propane-1,3-dione (1a): The title compound **1a** was prepared

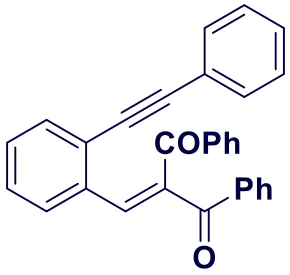

via procedure Step 2 (ethyl acetate/hexane, 1:9). (yield: 166.86 mg, 81%, white solid); ^{1}H NMR (400 MHz, $CDCl_3$) δ 8.22 (s, 1H), 8.02 – 7.96 (m, 2H), 7.95 – 7.88 (m, 2H), 7.57 – 7.47 (m, 3H), 7.44 – 7.36 (m, 5H), 7.35 – 7.16 (m, 6H), 7.10 (td, *J* = 7.8, 1.2 Hz, 1H); ^{13}C{^{1}H} NMR (100 MHz, $CDCl_3$) δ 196.6, 195.1, 142.5, 139.9, 137.4, 136.2, 134.7, 133.8, 132.4, 132.3, 131.4, 130.0, 129.5, 129.3, 128.8, 128.8, 128.7, 128.5, 128.4, 128.3, 124.5, 122.3, 96.4, 86.8.

2-(4-methyl-2-(phenylethynyl)benzylidene)-1,3-diphenylpropane-1,3-dione (1b): The title compound **1b** was

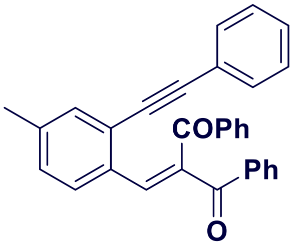

prepared via procedure Step 2 (ethyl acetate/hexane, 1:9). (yield: 183.18 mg, 86%, pale yellow solid), m.p. = 122-124 °C; HRMS (ESI) m/z: $[M+Na]^+$ Calcd for $C_{31}H_{22}O_2Na$, 449.1512; found, 449.1507; ^{1}H NMR (400 MHz, $CDCl_3$) δ 8.23 (s, 1H), 7.99 (dd, *J* = 8.0, 0.9 Hz, 2H), 7.90 (dd, *J* = 8.2, 1.2 Hz, 2H), 7.53 (dddd, *J* = 8.7, 5.5, 2.8, 1.3 Hz, 2H), 7.45 – 7.37 (m, 4H), 7.37 – 7.24 (m, 5H), 7.18 – 7.10 (m, 2H), 6.92 (d, *J* = 8.1 Hz, 1H), 2.26 (s, 3H); ^{13}C{^{1}H} NMR (100 MHz, $CDCl_3$) δ 197.0, 195.3, 142.7, 140.7, 138.9, 137.7, 136.2, 133.8, 132.8, 132.2, 131.8, 131.4, 129.5, 129.4, 129.3, 128.8, 128.6, 128.4, 128.3, 124.7, 122.4, 95.9, 86.9, 21.1.

2-(5-chloro-2-(phenylethynyl)benzylidene)-1,3-diphenylpropane-1,3-dione (1d): The title compound **1d** was

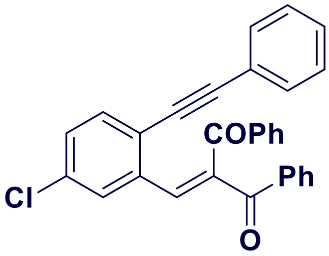

prepared via procedure Step 2 (ethyl acetate/hexane, 1:9). (yield: 160.56 mg, 72%, yellow solid), m.p. = 138-140 °C; HRMS (ESI) m/z: $[M+Na]^+$ Calcd for $C_{30}H_{19}ClO_2Na$, 469.0965; found, 469.0963; ^{1}H NMR (400 MHz, $CDCl_3$) δ 8.06 (s, 1H), 8.01 – 7.90 (m, 4H), 7.59 – 7.49 (m, 2H), 7.45 – 7.39 (m, 5H), 7.38 – 7.34 (m, 2H), 7.30 (ddd, *J* = 6.7, 4.5, 1.4 Hz, 2H), 7.25 – 7.18 (m, 3H); ^{13}C NMR (100 MHz, $CDCl_3$) δ 195.8, 194.6, 141.1, 141.6, 137.1, 136.4, 136.1, 134.3, 134.0, 133.2, 132.6, 131.5, 129.9, 129.5, 129.3, 129.0, 128.8, 128.7, 128.6, 128.4, 127.1, 122.6, 122.1, 97.1, 85.9.

1,3-diphenyl-2-(2-(p-tolylethynyl)benzylidene)propane-1,3-dione (1e): The title compound **1e** was prepared

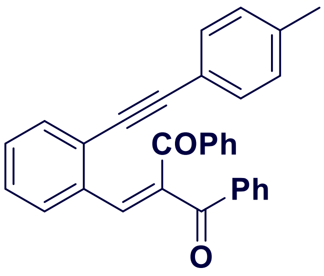

via procedure Step 2 (ethyl acetate/hexane, 1:9). (yield: 166.14 mg, 78%, yellow solid), m.p. = 125-127 °C; HRMS (ESI) m/z: $[M+Na]^+$ Calcd for $C_{31}H_{22}O_2Na$, 449.1512; found, 449.1507; ^{1}H NMR (400 MHz, $CDCl_3$) δ 8.22 (s, 1H), 8.01 – 7.96 (m, 2H), 7.95 – 7.89 (m, 2H), 7.58 – 7.47 (m, 3H), 7.46 – 7.37 (m, 5H), 7.26 – 7.21 (m, 1H), 7.13 – 7.03 (m, 5H), 2.37 (s, 3H); ^{13}C{^{1}H} NMR (100 MHz, $CDCl_3$) δ

196.6, 195.1, 142.6, 142.3, 139.8, 139.0, 137.5, 136.2, 134.6, 133.8, 132.9, 132.3, 132.2, 131.4, 130.2, 130.0, 129.7, 129.5, 129.3, 12.9.2, 129.1, 128.8, 128.8, 128.6, 128.5, 128.2, 124.8, 119.3, 96.6, 86.2, 21.5.

2-(2-((4-ethylphenyl)ethynyl)benzylidene)-1,3-diphenylpropane-1,3-dione (1f): The title compound **1f** was

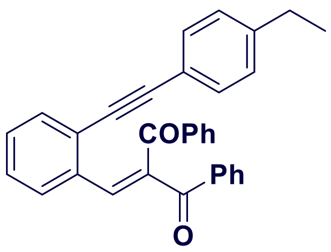

prepared via procedure Step 2 (ethyl acetate/hexane, 1:9). (yield: 160.6 mg, 73%, yellow solid), m.p. = 108-110 °C; HRMS (ESI) m/z: $[M+Na]^+$ Calcd for $C_{32}H_{24}O_2Na$, 463.1663; found, 463.1668; ^{1}H NMR (400 MHz, $CDCl_3$) δ 8.22 (s, 1H), 7.98 (dd, *J* = 8.4, 1.3 Hz, 2H), 7.92 (dd, *J* = 8.3, 1.3 Hz, 2H), 7.58 – 7.48 (m, 3H), 7.47 – 7.36 (m, 5H), 7.24 (d, *J* = 1.2 Hz, 1H), 7.11 (dd, *J* = 6.4, 2.0 Hz, 5H), 2.67 (q, *J* = 7.6 Hz, 2H), 1.24 (s, 3H); $^{13}C\{^1H\}$ NMR (100 MHz, $CDCl_3$) δ 196.6, 195.1, 145.3, 142.7, 139.8, 137.5, 136.2, 134.7, 133.8, 132.3, 132.2, 131.5, 130.0, 129.5, 129.3, 128.9, 128.8, 128.5, 128.2, 127.9, 127.1, 124.8, 119.5, 114.0, 110.0, 96.7, 86.2, 29.7, 16.4.

2-(2-((3-fluorophenyl)ethynyl)benzylidene)-1,3-diphenylpropane-1,3-dione (1g): The title compound **1g** was

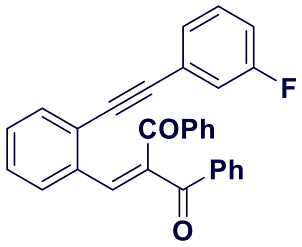

prepared via procedure Step 2 (ethyl acetate/hexane, 1:9). (yield: 150.5 mg, 70%, yellow solid), m.p. = 132-134 °C; HRMS (ESI) m/z: $[M+Na]^+$ Calcd for $C_{30}H_{19}FO_2Na$, 453.1267; found, 453.1262. ^{1}H NMR (400 MHz, $CDCl_3$) δ 8.17 (s, 1H), 8.01 – 7.95 (m, 2H), 7.94 – 7.89 (m, 2H), 7.60 – 7.49 (m, 3H), 7.43 (ddd, *J* = 8.7, 7.6, 1.6 Hz, 5H), 7.29 – 7.24 (m, 2H), 7.13 (td, *J* = 7.7, 1.0 Hz, 1H), 7.05 (tdd, *J* = 8.5, 2.6, 0.9 Hz, 1H), 7.01 – 6.95 (m, 1H), 6.83 (ddd, *J* = 9.3, 2.5, 1.5 Hz, 1H); $^{13}C\{^1H\}$ NMR (100 MHz, $CDCl_3$) δ 196.5, 195.1, 163.5, 161.0 (d, J_{C-F} = 245 Hz), 142.1, 140.1, 137.4, 136.1, 134.8, 133.9, 132.5, 132.4 (d, J_{C-F} = 7 Hz), 130.1, 130.0, 129.9, 129.4, 129.3, 128.9, 128.8 (t, J_{C-F} = 8 Hz), 128.5, 127.3, (d, J_{C-F} = 3 Hz), 124.2, 124.1 (t, J_{C-F} = 9 Hz), 118.3, 118.1 (d, J_{C-F} = 23 Hz), 116.2, 116.0 (d, J_{C-F} = 21 Hz), 95.0, 94.9, 87.5.

4-((2-(2-benzoyl-3-oxo-3-phenylprop-1-en-1-yl)phenyl)ethynyl)benzonitrile (1h): The title compound **1h** was

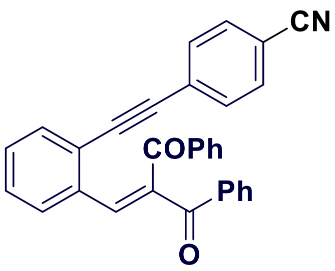

prepared via procedure Step 2 (ethyl acetate/hexane, 1:9). (yield: 150.76 mg, 69%, yellow solid), m.p. = 165-167 °C; HRMS (ESI) m/z: $[M+Na]^+$ Calcd for $C_{31}H_{19}NO_2Na$, 460.1308; found, 460.1307. ^{1}H NMR (400 MHz, $CDCl_3$) δ 8.15 (s, 1H), 8.01 – 7.88 (m, 4H), 7.60 – 7.50 (m, 5H), 7.43 (dt, *J* = 20.0, 7.5 Hz, 5H), 7.34 – 7.24 (m, 2H), 7.18 (dd, *J* = 16.7, 9.1 Hz, 2H); $^{13}C\{^1H\}$ NMR (100 MHz, $CDCl_3$) δ 196.3, 194.7, 141.6, 140.4, 137.4, 136.1, 135.0, 133.9, 132.6, 132.5, 132.0, 131.8, 130.1, 129.5, 129.3, 128.9, 128.8, 128.5, 127.2, 123.4, 118.3, 111.9, 94.2, 90.9.

methyl 4-((2-(2-benzoyl-3-oxo-3-phenylprop-1-en-1-yl)phenyl)ethynyl)benzoate (1i): The title compound **1i** was prepared via procedure Step 2 (ethyl acetate/hexane, 1:9). (yield: 58.1 mg, 86%, yellow solid), m.p. = 133-135 °C; HRMS (ESI) m/z: $[M+Na]^+$ Calcd for $C_{32}H_{22}O_4Na$, 493.1410; found, 493.1406; ^1H NMR (400 MHz, $CDCl_3$) δ 8.19 (s, 1H), 8.02 – 7.89 (m, 6H), 7.61 – 7.49 (m, 3H), 7.44 (ddd, *J* = 18.3, 11.5, 4.4 Hz, 5H), 7.27 (ddd, *J* = 7.6, 6.8, 1.1 Hz, 1H), 7.24 – 7.19 (m, 2H), 7.15 (dd, *J* = 7.7, 1.0 Hz, 1H), 3.94 (s, 3H); $^{13}C\{^1H\}$ NMR (100 MHz, $CDCl_3$) δ 196.4, 195.0, 166.3, 142.0, 140.2, 137.4, 136.1, 134.8, 133.9, 132.5, 132.4, 131.3, 130.0, 129.8, 129.5, 129.4, 129.3, 128.9, 128.9, 128.8, 128.5, 127.0, 123.9, 95.3, 89.5, 52.3.

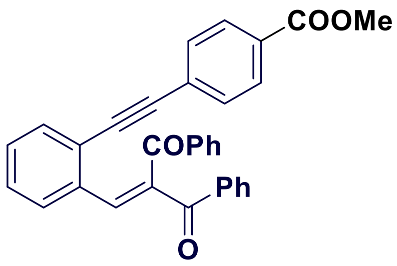

1,3-diphenyl-2-((2-(phenylethynyl)pyridin-3-yl)methylene)propane-1,3-dione (1j): The title compound **1j** was prepared via procedure Step 2 (ethyl acetate/hexane, 1:9). (yield: 119.77 mg, 58%, brown solid), m.p. = 154-156 °C; HRMS (ESI) m/z: $[M+Na]^+$ Calcd for $C_{29}H_{19}NO_2Na$, 436.1308; found, 436.1305; ^1H NMR (400 MHz, $CDCl_3$) δ 8.49 (dd, J = 4.8, 1.5 Hz, 1H), 8.14 (s, 1H), 7.95 (dd, J = 12.8, 5.0 Hz, 4H), 7.71 (dd, *J* = 8.1, 1.5 Hz, 1H), 7.61 – 7.52 (m, 2H), 7.49 – 7.36 (m, 5H), 7.34 – 7.23 (m, 4H), 7.06 (dd, J = 8.1, 4.8 Hz, 1H); $^{13}C\{^1H\}$ NMR (100 MHz, $CDCl_3$) δ 195.8, 194.4, 150.9, 143.5, 142.0, 139.5, 136.9, 135.9, 135.8, 134.2, 132.7, 131.9, 131.2, 129.5, 129.3, 128.9, 128.6, 128.4, 122.5, 121.4, 96.1, 86.4.

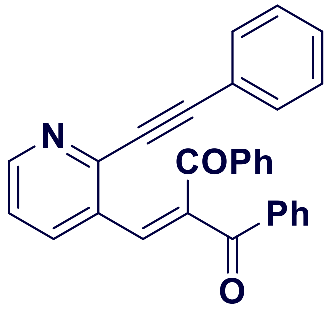

2-(2-(hex-1-yn-1-yl)benzylidene)-1,3-diphenylpropane-1,3-dione (1k): The title compound **1k** was prepared via procedure Step 2 (ethyl acetate/hexane, 1:9). (yield: 101.92 mg, 52%, yellow liquid); HRMS (ESI) m/z: $[M+Na]^+$ Calcd for $C_{28}H_{24}O_2Na$, 415.1668; found, 415.1665; ^1H NMR (400 MHz, $CDCl_3$) δ 8.07 (s, 1H), 7.98 – 7.88 (m, 4H), 7.62 – 7.55 (m, 1H), 7.49 (tdt, *J* = 6.7, 4.4, 1.0 Hz, 3H), 7.42 – 7.29 (m, 4H), 7.16 (td, *J* = 7.6, 1.1 Hz, 1H), 7.03 (td, *J* = 7.7, 1.0 Hz, 1H), 2.32 (t, *J* = 6.9 Hz, 2H), 1.44 – 1.38 (m, 2H), 1.34 – 1.28 (m, 2H), 0.88 – 0.83 (m, 3H); $^{13}C\{^1H\}$ NMR (100 MHz, $CDCl_3$) δ 196.6, 195.0, 142.9, 139.5, 137.4, 136.2, 134.8, 133.7, 132.4, 132.3, 129.8, 129.6, 129.3, 128.7, 128.7, 128.6, 128.3, 127.6, 127.1, 125.3, 97.9, 78.2, 30.4, 21.9, 19.1, 13.5.

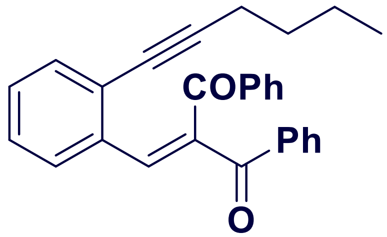

1,3-diphenyl-2-((2-(phenylethynyl)cyclohex-1-en-1-yl)methylene)propane-1,3-dione (1l): The title compound **1l** was prepared via procedure Step 2 (ethyl acetate/hexane, 1:9). (yield: 141.44 mg, 68%, yellow solid), m.p. = 198-200 °C; HRMS (ESI) m/z: $[M+Na]^+$ Calcd for $C_{30}H_{24}O_2Na$, 439.1668; found, 439.1665; ^1H NMR (400 MHz, $CDCl_3$) δ 8.09 (s, 1H), 8.03 – 7.95 (m, 2H), 7.83 (dd, *J* = 5.1, 3.4 Hz, 2H), 7.62 – 7.39 (m, 6H), 7.33 – 7.27 (m, 1H), 7.22 (dd, *J* = 10.2, 4.6 Hz, 2H), 6.94 (dd, *J* = 5.1, 3.4 Hz, 2H), 2.42 (t, *J* =

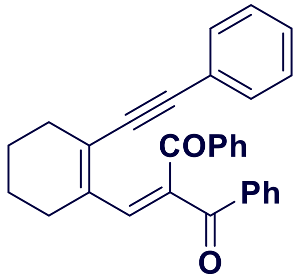

5.9 Hz, 2H), 2.09 (t, J = 5.8 Hz, 2H), 1.58 – 1.49 (m, 4H); $^{13}C\{^1H\}$ NMR (100 MHz, $CDCl_3$) δ 197.1, 196.3, 145.5, 138.7, 138.2, 137.3, 137.0, 133.6, 132.3, 131.8, 131.4, 129.4, 129.2, 128.8, 128.6, 128.4, 128.1, 122.4, 99.0, 88.5, 31.8, 27.5, 22.1, 21.4.

(*E*)-1-phenyl-2-(2-(phenylethynyl)benzylidene)butane-1,3-dione (1m): The title compound **1m** was prepared via procedure Step 2 (ethyl acetate/hexane, 1:9). (yield: 134.75 mg, 77%, yellow solid); ^1H NMR (400 MHz, $CDCl_3$) δ 8.46 (s, 1H), 7.98 – 7.81 (m, 2H), 7.61 (dd, J = 6.5, 3.1 Hz, 2H), 7.55 – 7.47 (m, 2H), 7.45 – 7.36 (m, 5H), 7.30 – 7.20 (m, 2H), 7.07 (t, J = 7.7 Hz, 1H), 2.49 (s, 3H); ^{13}C NMR (100 MHz, $CDCl_3$) δ 197.5, 196.1, 140.3, 139.7, 135.9, 134.5, 134.0, 132.4, 131.5, 130.0, 129.1, 128.9, 128.8, 128.7, 128.6, 128.4, 124.6, 122.5, 96.5, 86.8, 27.1.

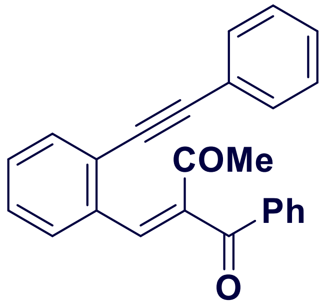

1,3-diphenyl-2-(2-((3-(trifluoromethyl)phenyl)ethynyl)benzylidene)propane-1,3-dione (1r): The title compound **1r** was prepared via procedure Step 2 (ethyl acetate/hexane, 1:9). (yield: 124.8 mg, 52%, brown liquid); HRMS (ESI) m/z: $[M+Na]^+$ Calcd for $C_{31}H_{19}F_3O_2Na$, 503.1229; found, 503.1225; ^1H NMR (400 MHz, $CDCl_3$) δ 8.16 (s, 1H), 8.03 – 7.96 (m, 2H), 7.95 – 7.89 (m, 2H), 7.60 (d, J = 7.8 Hz, 1H), 7.57 – 7.49 (m, 4H), 7.47 – 7.38 (m, 6H), 7.35 (d, J = 7.8 Hz, 1H), 7.29 (dd, J = 7.6, 1.2 Hz, 1H), 7.15 (td, J = 7.8, 1.3 Hz, 1H); $^{13}C\{^1H\}$ NMR (100 MHz, $CDCl_3$) δ 196.4, 195.0, 142.0, 140.3, 137.3, 136.1 (d, J_{C-F} = 120 Hz), 134.8, 134.7, 133.9, 132.6, 132.5 (d, J_{C-F} = 11 Hz), 130.1, 129.9, 129.5, 129.3 (d, J_{C-F} = 13 Hz), 128.9, 128.9, 128.9, 128.8, 128.7 (d, J_{C-F} = 34 Hz), 128.5, 128.3, 128.0, (d, J_{C-F} = 4 Hz), 127.7, 125.3, 125.2 (q, J_{C-F} = 8 Hz), 123.8, 123.4, 94.4, 88.2.

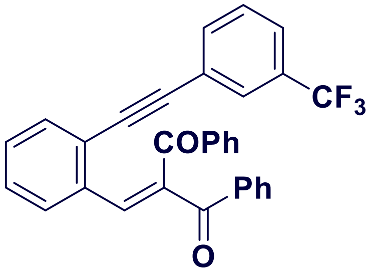

2-(2-((4-methoxyphenyl)ethynyl)benzylidene)-1,3-diphenylpropane-1,3-dione (1s): The title compound **1s** was prepared via procedure Step 2 (ethyl acetate/hexane, 1:9). (yield: 148.07 mg, 67%, yellow solid), m.p. = 102-104 °C; HRMS (ESI) m/z: $[M+Na]^+$ Calcd for $C_{31}H_{22}O_3Na$, 465.1467; found, 465.1464; ^1H NMR (400 MHz, $CDCl_3$)δ 8.22 (s, 1H), 8.03 – 7.96 (m, 2H), 7.95 – 7.89 (m, 2H), 7.58 – 7.48 (m, 3H), 7.47 – 7.41 (m, 3H), 7.40 – 7.37 (m, 2H), 7.23 (dt, J = 7.6, 3.8 Hz, 1H), 7.15 – 7.03 (m, 3H), 6.85 – 6.77 (m, 2H), 3.84 (s, 3H); $^{13}C\{^1H\}$ NMR (100 MHz, $CDCl_3$) δ 196.7, 195.1, 160.0, 142.7, 139.7, 137.5, 136.2, 136.3, 134.5, 133.8, 133.0, 132.3, 132.1, 131.5, 130.0, 129.5, 129.3, 128.8, 128.8, 128.5, 128.2, 128.0, 125.0, 114.5, 114.1, 114.0, 96.6, 85.7, 55.3, 29.7.

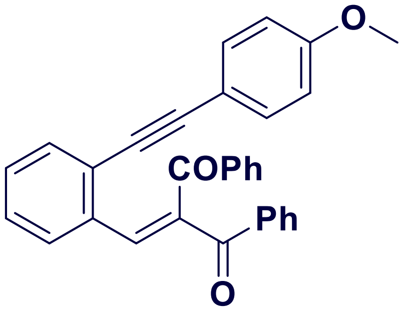

(*E*)-2-methyl-1-phenyl-3-(2-(phenylethynyl)phenyl)prop-2-en-1-one (1u): The title compound **1u** was prepared via procedure Step 2 (ethyl acetate/hexane, 1:9). (yield: 77.28 mg, 48%, pale yellow solid), m.p. = 79-81 °C; HRMS (ESI) m/z: $[M+Na]^+$ Calcd for $C_{24}H_{18}ONa$, 345.1256; found, 345.1252; ^1H NMR (400 MHz, $CDCl_3$) δ 7.86 – 7.77 (m, 2H), 7.63 (d, *J* = 0.7 Hz, 1H), 7.59 (dd, *J* = 7.6, 1.5 Hz, 1H), 7.51 (d, *J* = 7.3 Hz, 1H), 7.46 – 7.37 (m, 2H), 7.37 – 7.22 (m, 8H), 2.23 (d, *J* = 1.4 Hz, 3H); ^{13}C NMR (100 MHz, $CDCl_3$) δ 199.4, 140.9, 138.3, 137.8, 137.6, 132.3, 131.5, 131.5, 129.4, 128.9, 128.5, 128.3, 128.3, 128.1, 128.0, 123.6, 122.8, 94.9, 87.5, 14.3.

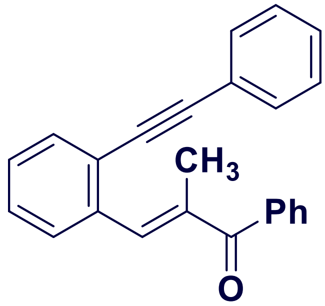

***N*-(6-benzoyl-11-phenyl-11*H*-benzo[*a*]fluoren-11-yl)acetamide (3aa):** The title compound **3aa** was prepared via procedure 3.2 (ethyl acetate/hexane, 4:6). (yield: 58.1 mg, 86%, white solid), m.p. = 244-246 °C; HRMS (ESI) m/z: $[M+Na]^+$ Calcd for $C_{32}H_{23}NO_2Na$, 476.1621; found, 476.1617; ^1H NMR (400 MHz, $CDCl_3$) δ 8.05 (d, *J* = 7.4 Hz, 2H), 7.95 – 7.86 (m, 2H), 7.77 (d, *J* = 7.9 Hz, 1H), 7.65 – 7.54 (m, 2H), 7.52 – 7.41 (m, 4H), 7.37 (d, *J* = 7.6 Hz, 1H), 7.28 (d, *J* = 4.8 Hz, 2H), 7.26 – 7.21 (m, 3H), 7.11 (dt, *J* = 25.5, 7.3 Hz, 2H), 6.78 (s, 1H), 1.99 (s, 3H); $^{13}C\{^1H\}$ NMR (100 MHz, $CDCl_3$) δ 197.7, 168.9, 150.6, 143.6, 141.1, 138.2, 137.1, 136.6, 133.8, 132.9, 132.7, 130.7, 130.1, 129.9, 129.2, 128.9, 128.8, 128.0, 127.8, 127.8, 126.1, 125.5, 124.1, 123.8, 123.1, 70.0, 23.9.

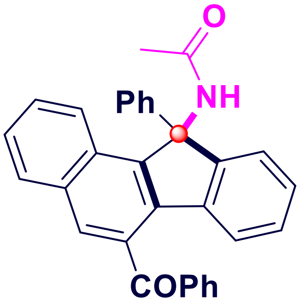

***N*-(6-benzoyl-2-methyl-11-phenyl-11*H*-benzo[*a*]fluoren-11-yl)acetamide (3ba):** The title compound **3ba** was prepared via procedure 3.2 (ethyl acetate/hexane, 4:6). (yield: 62.4 mg, 89%, white solid), m.p. = 214-216 °C; HRMS (ESI) m/z: $[M+Na]^+$ Calcd for $C_{33}H_{25}NO_2Na$, 490.1777; found, 490.1773; ^1H NMR (400 MHz, $CDCl_3$) δ 8.03 (d, *J* = 7.3 Hz, 2H), 7.87 (s, 1H), 7.78 (d, *J* = 8.4 Hz, 1H), 7.64 – 7.53 (m, 3H), 7.47 (t, *J* = 7.7 Hz, 2H), 7.38 (d, *J* = 7.5 Hz, 1H), 7.28 (dd, *J* = 10.5, 5.0 Hz, 3H), 7.22 (dd, *J* = 8.8, 3.1 Hz, 3H), 7.10 (dt, *J* = 22.3, 7.1 Hz, 2H), 6.78 (s, 1H), 2.38 (s, 3H), 1.97 (s, 3H); $^{13}C\{^1H\}$ NMR (100 MHz, $CDCl_3$) δ 197.8, 168.9, 150.7, 142.9, 141.0, 138.3, 137.8, 137.2, 136.6, 133.6, 132.0, 130.9, 130.6, 130.0, 129.7, 129.5, 128.8, 128.7, 128.4, 127.9, 127.7, 125.6, 124.2, 123.0, 122.7, 70.1, 23.9, 22.2.

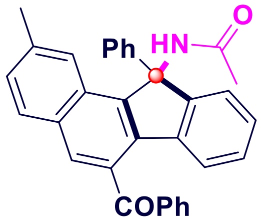

***N*-(6-benzoyl-3-fluoro-11-phenyl-11*H*-benzo[*a*]fluoren-11-yl)acetamide (3ca):** The title compound **3ca** was prepared via procedure 3.2 (ethyl acetate/hexane, 4:6). (yield: 48.7 mg, 69 white solid), m.p. = 285-287 °C; HRMS (ESI) m/z: $[M+Na]^+$ Calcd for $C_{32}H_{22}FNO_2Na$, 494.1527; found, 494.1525; ^1H NMR (400 MHz, $CDCl_3$) δ 8.04 (d, *J* = 7.4 Hz, 2H), 7.84 (s, 1H), 7.80 (dd, *J* = 9.2, 5.4 Hz, 1H), 7.62 (t, *J* = 7.3 Hz, 1H), 7.50 (dd, *J* = 14.5, 7.2 Hz, 4H), 7.33 (d, *J* = 7.6 Hz, 1H), 7.27 – 7.04 (m, 8H), 6.77 (s, 1H), 1.99 (s, 3H); $^{13}C\{^1H\}$ NMR

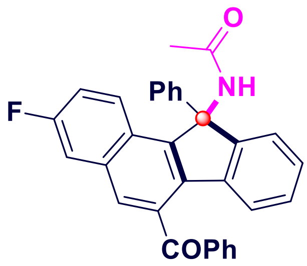

(100 MHz, $CDCl_3$) δ 197.5, 168.8, 161.7, 159.2 (d, J_{C-F} = 248 Hz), 150.3, 143.9, 140.9, 138.1, 136.9, 136, 134.1, 133.9, 133.7, 133.6 (d, J_{C-F} = 9 Hz), 130.6, 129.0 (t, J_{C-F} = 157 Hz), 128.8, 128.1, 128.0 (t, J_{C-F} = 6 Hz), 127.8, 126.4, 126.3, 125.3, 123.7, 123.0 (d, J_{C-F} = 75 Hz), 118.3, 118.0 (d, J_{C-F} = 25 Hz), 113.0, 112.8 (d, J_{C-F} = 20 Hz), 109.9, 69.9, 23.9.

***N*-(6-benzoyl-3-chloro-11-phenyl-11*H*-benzo[*a*]fluoren-11-yl)acetamide (3da):** The title compound **3da** was prepared via procedure 3.2 (ethyl acetate/hexane, 4:6). (yield: 53.4 mg, 73%, white solid), m.p. = 277-279 °C; HRMS (ESI) m/z: $[M+Na]^+$ Calcd for $C_{32}H_{22}ClNO_2Na$, 510.1231; found, 510.1230. ^1H NMR (400 MHz, $CDCl_3$) δ 8.03 (d, J = 7.8 Hz, 2H), 7.88 (s, 1H), 7.82 (s, 1H), 7.73 (d, J = 8.6 Hz, 1H), 7.62 (t, J = 7.3 Hz, 1H), 7.49 (t, J = 7.6 Hz, 3H), 7.34 (d, J = 7.4 Hz, 2H), 7.26 (d, J = 1.9 Hz, 5H), 7.16 (t, J = 6.3 Hz, 1H), 7.08 (t, J = 7.4 Hz, 1H), 6.76 (s, 1H), 1.99 (s, 3H); $^{13}C\{^1H\}$ NMR (100 MHz, $CDCl_3$) δ 197.4, 168.7, 150.5, 143.8, 140.9, 137.9, 136.9, 136.86, 134.06, 133.96, 133.36, 131.94, 130.62, 129.09, 128.83, 128.62, 128.42, 128.1, 127.4, 125.4, 125.3, 123.8, 123.2, 69.9, 30.9.

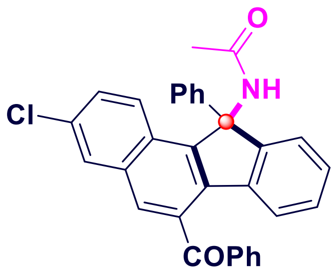

***N*-(6-benzoyl-11-(*p*-tolyl)-11H-benzo[*a*]fluoren-11-yl)acetamide (3ea):** The title compound **3ea** was prepared via procedure 3.2 (ethyl acetate/hexane, 4:6). (yield: 54.7 mg, 78%, off-white solid), m.p. = 203-205 °C; HRMS (ESI) m/z: $[M+Na]^+$ Calcd for $C_{33}H_{25}NO_2Na$, 490.1777; found, 490.1775; ^1H NMR (400 MHz, $CDCl_3$) δ 8.05 (d, J = 7.3 Hz, 2H), 7.90 (d, J = 11.0 Hz, 2H), 7.78 (d, J = 7.8 Hz, 1H), 7.60 (d, J = 7.4 Hz, 1H), 7.54 – 7.39 (m, 5H), 7.35 (d, J = 7.6 Hz, 1H), 7.15 (dd, J = 19.2, 7.8 Hz, 3H), 7.05 (dd, J = 13.1, 7.8 Hz, 3H), 6.77 (s, 1H), 2.26 (s, 3H), 1.97 (s, 3H); $^{13}C\{^1H\}$ NMR (100 MHz, $CDCl_3$) δ 197.8, 168.7, 150.8, 143.6, 138.2, 138.1, 137.6, 137.1, 136.6, 133.7, 132.9, 132.6, 130.6, 129.9, 129.9, 129.6, 129.2, 128.7, 127.8, 127.7, 126.0, 125.3, 123.9, 123.7, 123.1, 69.8, 23.9, 20.9.

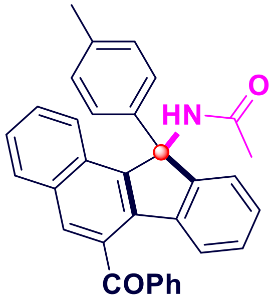

***N*-(6-benzoyl-11-(4-ethylphenyl)-11*H*-benzo[*a*]fluoren-11-yl)acetamide (3fa):** The title compound **3fa** was prepared via procedure 3.2 (ethyl acetate/hexane, 4:6). (yield: 61.4 mg, 85%, off-white solid), m.p. = 183-185 °C; HRMS (ESI) m/z: $[M+Na]^+$ Calcd for $C_{34}H_{27}NO_2Na$, 504.1934; found, 504.1931. ^1H NMR (400 MHz, $CDCl_3$) δ 8.05 (d, J = 7.2 Hz, 2H), 7.94 – 7.86 (m, 2H), 7.79 (d, J = 8.3 Hz, 1H), 7.61 (t, J = 7.5 Hz, 1H), 7.56 – 7.41 (m, 5H), 7.35 (d, J = 7.7 Hz, 1H), 7.32 – 7.28 (m, 1H), 7.21 (s, 1H), 7.14 (t, J = 7.3 Hz, 1H), 7.11 – 7.03 (m, 3H), 6.69 (d, J = 31.1 Hz, 1H), 2.57 (d, J = 7.6 Hz, 2H), 2.00 (d, J = 11.4 Hz, 3H), 1.17 (t, J = 7.6 Hz, 3H); $^{13}C\{^1H\}$ NMR (101 MHz, $CDCl_3$) δ 197.8, 168.7, 150.7, 143.9, 138.3, 137.2, 136.6, 133.7, 132.7, 130.7, 129.9, 129.8, 128.7, 128.4, 127.9, 127.7, 126.0, 125.6, 125.4, 124.4, 124.4, 123.9, 123.7, 123.1, 122.7, 110.0, 69.8, 28.3, 23.9, 15.2.

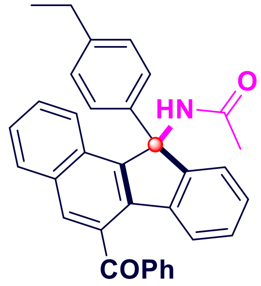

N-(6-benzoyl-11-(3-fluorophenyl)-11H-benzo[a]fluoren-11-yl)acetamide (3ga): The title compound **3ga** was prepared via procedure 3.2 (ethyl acetate/hexane, 4:6). (yield: 43.8 mg, 62%, off-white solid), m.p. = 138-140 °C; HRMS (ESI) m/z: $[M+Na]^+$ Calcd for $C_{32}H_{22}FNO_2Na$, 494.1527; found, 494.1523.^{1}H NMR (400 MHz, $CDCl_3$) δ 8.05 (d, *J* = 7.4 Hz, 2H), 7.98 – 7.86 (m, 2H), 7.82 – 7.73 (m, 1H), 7.64 (dd, *J* = 7.3, 5.6 Hz, 2H), 7.46 (ddd, *J* = 30.1, 20.9, 7.6 Hz, 5H), 7.25 – 7.04 (m, 4H), 7.03 – 6.86 (m, 2H), 6.59 (s, 1H), 2.02 (s, 3H); ^{13}C{^{1}H} NMR (150 MHz, $CDCl_3$) δ 197.5, 169.1, 163.8, 162.1 (d, J_{C-F} = 170 Hz), 150.1, 143.6 (d, J_{C-F} = 4 Hz), 143.4, 138.3, 137.1, 136.5, 133.9, 132.9, 132.7, 130.7, 130.4, 130.1, 129.0, 128.8, 128.3, 128.1, 128.0 (t, J_{C-F} = 22 Hz), 126.3, 124.8 (d, J_{C-F} = 145 Hz), 123.5, 123.2 (d, J_{C-F} = 35 Hz), 121.4, 114.8, 114.6 (d, J_{C-F} = 20 Hz), 113.2, 113.0 (d, J_{C-F} = 27 Hz), 69.8, 30.3.

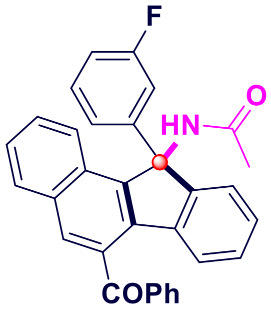

N-(6-benzoyl-11-(4-cyanophenyl)-11H-benzo[a]fluoren-11-yl)acetamide (3ha): The title compound **3ha** was prepared via procedure 3.2 (ethyl acetate/hexane, 4:6). (yield: 48.8 mg, 68%, off-white solid), m.p. = 188-190 °C; HRMS (ESI) m/z: $[M+Na]^+$ Calcd for $C_{33}H_{22}N_2O_2Na$, 501.1573; found 501.1569; ^{1}H NMR (400 MHz, $CDCl_3$) δ 8.04 (dd, *J* = 8.4, 1.2 Hz, 2H), 7.74 – 7.68 (m, 2H), 7.65 – 7.59 (m, 3H), 7.58 – 7.53 (m, 3H), 7.49 (dt, *J* = 8.6, 1.2 Hz, 1H), 7.45 – 7.40 (m, 1H), 7.36 – 7.30 (m, 2H), 7.28 (dd, *J* = 7.8, 1.4 Hz, 1H), 7.22 (td, *J* = 7.5, 1.2 Hz, 1H), 7.18 – 7.11 (m, 2H), 6.37 (dd, J = 8.6, 3.7 Hz, 1H), 6.18 (d, *J* = 3.8 Hz, 1H), 2.05 (s, 3H); ^{13}C{^{1}H} NMR (100 MHz, $CDCl_3$) δ 196.2, 193.3, 169.6, 141.9, 136.5, 135.2, 133.9, 133.8, 133.0, 132.3, 132.1, 129.6, 128.9, 128.8, 128.6, 128.2, 127.7, 127.6, 127.5, 120.1, 118.4, 111.9, 93.4, 91.9, 57.6, 51.4, 23.3.

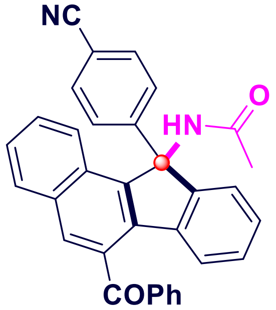

methyl-4-(11-acetamido-6-benzoyl-11H-benzo[a]fluoren-11-yl)benzoate (3ia): The title compound **3ia** was prepared via procedure 3.2 (ethyl acetate/hexane, 4:6). (yield: 49.1 mg, 64%, off-white solid), m.p. = 110-112 °C; HRMS (ESI) m/z: $[M+Na]^+$ Calcd for $C_{34}H_{25}NO_4Na$, 534.1676; found, 534.1671; ^{1}H NMR (400 MHz, $CDCl_3$) δ 8.05 (d, *J* = 7.2 Hz, 2H), 7.92 (dd, *J* = 8.9, 7.1 Hz, 4H), 7.76 (d, *J* = 7.9 Hz, 1H), 7.64 (t, *J* = 7.4 Hz, 2H), 7.46 (ddd, *J* = 16.1, 14.8, 7.5 Hz, 5H), 7.35 (d, *J* = 8.5 Hz, 2H), 7.15 (dd, *J* = 7.3, 5.5 Hz, 2H), 6.60 (s, 1H), 3.86 (s, 3H), 2.04 (s, 3H); ^{13}C{^{1}H} NMR (100 MHz, $CDCl_3$) δ 197.5, 169.3, 166.5, 150.2, 145.8, 143.5, 138.3, 137.0, 136.4, 133.9, 132.8, 132.7, 130.6, 130.5, 130.0, 129.4, 128.9, 128.8, 128.4, 128.2, 128.0, 126.3, 125.9, 125.2, 123.4, 123.1, 70.3, 52.1, 24.1.

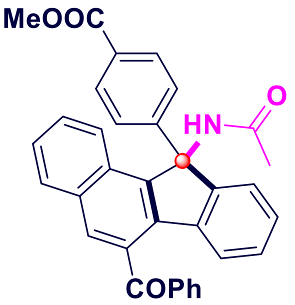

_N_-(6-benzoyl-11-phenyl-11_H_-indeno[1,2-_h_]quinolin-11-yl)acetamide (3ja): The title compound **3ja** was prepared via procedure 3.2 (ethyl acetate/hexane, 4:6). (yield: 31.3 mg, 46%, light-brown solid), m.p. = 184-186 °C; HRMS (ESI) m/z: $[M+Na]^+$ Calcd for $C_{31}H_{22}N_2O_2Na$, 477.1579; found, 477.1577; ^1H NMR (400 MHz, $CDCl_3$) δ 8.83 (dd, J = 4.2, 1.6 Hz, 1H), 8.20 (d, J = 7.4 Hz, 1H), 8.07 – 7.97 (m, 2H), 7.90 (s, 1H), 7.65 – 7.57 (m, 2H), 7.49 (t, J = 7.7 Hz, 3H), 7.39 – 7.32 (m, 4H), 7.20 (tt, J = 6.0, 3.0 Hz, 4H), 7.08 (t, J = 7.3 Hz, 1H), 2.00 (s, 3H); $^{13}C\{^1H\}$ NMR (100 MHz, $CDCl_3$) δ 197.0, 170.1, 151.3, 150.8, 137.4, 136.8, 134.0, 130.6, 129.6, 128.9, 128.6, 128.4, 127.8, 127.6, 127.5, 125.3, 124.4, 123.6, 121.1, 109.9, 70.1, 23.9.

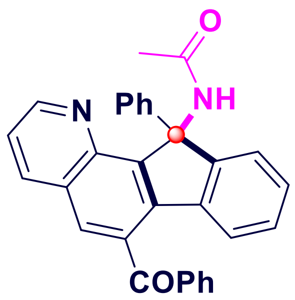

_N_-(6-benzoyl-11-butyl-11_H_-benzo[_a_]fluoren-11-yl)acetamide (3ka): The title compound **3ka** was prepared via procedure 3.2 (ethyl acetate/hexane, 4:6). (yield: 27.3 mg, 42%, light brown solid), m.p. = 242-244 °C; HRMS (ESI) m/z: $[M+Na]^+$ Calcd for $C_{30}H_{27}NO_2Na$, 456.1940; found 456.1937; ^1H NMR (400 MHz, $CDCl_3$) δ 8.28 (d, J = 8.4 Hz, 1H), 8.02 – 7.88 (m, 4H), 7.69 – 7.56 (m, 4H), 7.48 (dt, J = 15.5, 7.4 Hz, 4H), 7.32 (t, J = 6.1 Hz, 1H), 7.25 – 7.20 (m, 1H), 7.13 (dd, J = 10.9, 4.3 Hz, 1H), 6.23 (s, 1H), 2.71 – 2.61 (m, 1H), 2.53 (td, J = 12.1, 5.5 Hz, 1H), 1.94 (s, 3H), 1.29 – 1.23 (m, 2H), 1.04 (dd, J = 9.0, 5.7 Hz, 2H), 0.61 (t, J = 7.3 Hz, 3H); $^{13}C\{^1H\}$ NMR (100 MHz, $CDCl_3$) δ 197.8, 168.7, 149.3, 142.5, 138.9, 137.2, 135.5, 133.7, 132.4, 130.6, 130.1, 129.4, 128.7, 128.0, 127.7, 127.5, 126.8, 126.0, 123.4, 123.3, 122.9, 122.8, 122.3, 122.1, 67.6, 38.7, 24.8, 23.8, 22.5, 13.6.

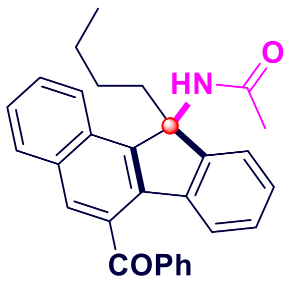

_N_-(6-benzoyl-11-phenyl-2,3,4,11-tetrahydro-1_H_-benzo[_a_]fluoren-11-yl)acetamide (3la): The title compound **3la** was prepared via procedure 3.2 (ethyl acetate/hexane, 4:6). (yield: 52.8 mg, 77%, white solid), m.p. = 186-188 °C; HRMS (ESI) m/z: $[M+Na]^+$ Calcd for $C_{32}H_{27}NO_2Na$, 480.1934; found, 480.1930; ^1H NMR (400 MHz, $CDCl_3$) δ 7.99 (t, J = 12.0 Hz, 2H), 7.57 (t, J = 7.3 Hz, 1H), 7.46 (t, J = 7.7 Hz, 2H), 7.38 – 7.26 (m, 6H), 7.18 (d, J = 7.6 Hz, 1H), 7.06 (dd, J = 15.4, 7.9 Hz, 2H), 6.96 (t, J = 7.1 Hz, 1H), 6.57 (s, 1H), 2.82 (d, J = 5.8 Hz, 2H), 2.74 – 2.63 (m, 1H), 2.31 (dt, J = 16.6, 5.1 Hz, 1H), 2.05 (s, 3H), 1.79 – 1.70 (m, 3H), 1.33 – 1.24 (m, 1H); $^{13}C\{^1H\}$ NMR (100 MHz, $CDCl_3$) δ 198.4, 168.4, 149.6, 145.5, 140.9, 137.7, 137.1, 137.0, 136.6, 135.5, 133.5, 131.9, 130.5, 130.3, 129.3, 128.9, 128.6, 127.8, 127.6, 127.5, 124.7, 123.6, 123.1, 123.0, 69.6, 29.6, 26.2, 23.7, 22.6, 22.5.

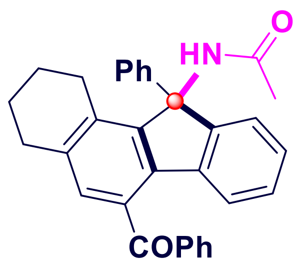

***N*-(6-acetyl-11-phenyl-11*H*-benzo[*a*]fluoren-11-yl)acetamide (3ma):** The title compound **3ma** was prepared via procedure 3.2 (ethyl acetate/hexane, 4:6). (yield: 49.3 mg, 84%, off-white solid), m.p. = 238-239 °C; HRMS (ESI) m/z: $[M+Na]^+$ Calcd for $C_{27}H_{21}NO_2Na$, 414.1458; found, 414.1461; ^{1}H NMR (400 MHz, $CDCl_3$) δ 8.13 (s, 1H), 7.95 (d, *J* = 7.5 Hz, 1H), 7.84 (d, *J* = 7.3 Hz, 1H), 7.72 (d, *J* = 7.2 Hz, 1H), 7.60 – 7.36 (m, 4H), 7.23 (s, 6H), 6.69 (s, 1H), 2.87 (s, 3H), 1.97 (d, *J* = 20.4 Hz, 3H); ^{13}C{^{1}H} NMR (100 MHz, $CDCl_3$) δ 202.8, 168.7, 150.8, 144.5, 141.1, 138.4, 135.8, 134.6, 132.6, 130.2, 130.1, 129.6, 128.9, 128.2, 128.1, 128.0, 127.8, 126.1, 125.4, 123.9, 123.6, 69.8, 30.4, 24.0.

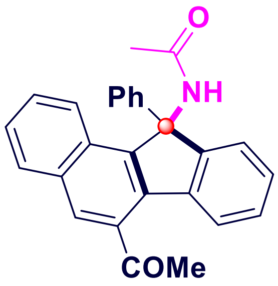

ethyl 11-acetamido-11-phenyl-11*H*-benzo[*a*]fluorene-6-carboxylate (3na): The title compound **3na** was prepared via procedure 3.2 (ethyl acetate/hexane, 4:6). (yield: 58.1 mg, 92%, white solid), m.p. = 258-260 °C; HRMS (ESI) m/z: $[M+Na]^+$ Calcd for $C_{28}H_{23}NO_3Na$, 444.1564; found, 444.1566. ^{1}H NMR (600 MHz, $CDCl_3$) δ 8.37 (s, 1H), 8.16 (d, *J* = 7.8 Hz, 1H), 7.95 (d, *J* = 7.8 Hz, 1H), 7.73 (d, *J* = 8.2 Hz, 1H), 7.60 (d, *J* = 7.5 Hz, 1H), 7.42 (dt, *J* = 14.7, 6.9 Hz, 2H), 7.31 (t, *J* = 7.4 Hz, 1H), 7.22 (dd, *J* = 14.8, 6.8 Hz, 6H), 6.62 (s, 1H), 4.59 – 4.54 (m, 2H), 1.94 (s, 3H), 1.51 (t, *J* = 7.1 Hz, 3H); ^{13}C{^{1}H} NMR (150 MHz, $CDCl_3$) δ 168.7, 168.2, 150.8, 144.3, 141.2, 138.5, 136.8, 132.6, 130.2, 129.8, 128.8, 128.2, 128.0, 127.9, 127.7, 126.0, 125.8, 125.5, 124.1, 123.8, 69.9, 61.6, 23.9, 14.4.

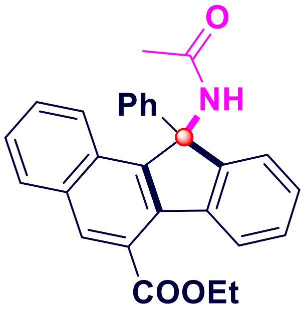

***N*-(6-cyano-11-phenyl-11*H*-benzo[*a*]fluoren-11-yl)acetamide (3oa):** The title compound **3oa** was prepared via procedure 3.2 (ethyl acetate/hexane, 4:6). (yield: 39.3 mg, 70%, light-brown solid), m.p. = 323-325 °C; HRMS (ESI) m/z: $[M+Na]^+$ Calcd for $C_{26}H_{18}N_2ONa$, 397.1311; found, 397.1309; ^{1}H NMR (400 MHz, DMSO-*d*$_6$ + $CDCl_3$) δ 9.05 (s, 1H), 8.40 (s, 1H), 8.33 (d, J = 7.4 Hz, 1H), 8.06 – 7.98 (m, 2H), 7.80 (s, 1H), 7.58 – 7.49 (m, 3H), 7.36 (td, J = 7.5, 1.1 Hz, 1H), 7.28 (td, J = 7.5, 1.0 Hz, 1H), 7.24 – 7.20 (m, 4H), 1.90 (s, 3H); ^{13}C{^{1}H} NMR (100 MHz, DMSO-*d*$_6$ + $CDCl_3$) δ 168.9, 151.4, 144.4, 140.4, 136.5, 136.2, 135.7, 131.7, 130.0, 129.2, 128.7, 128.1, 128.0, 127.3, 126.1, 125.3, 124.1, 122.6, 120.7, 118.0, 102.3, 69.0, 22.6.

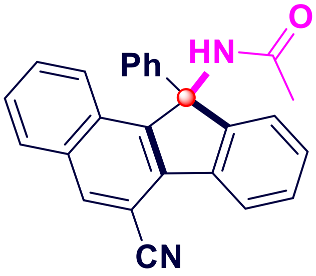

***N*-(6-cyano-9-fluoro-11-phenyl-11*H*-benzo[*a*]fluoren-11-yl)acetamide (3pa):** The title compound **3pa** was prepared via procedure 3.2 (ethyl acetate/hexane, 4:6). (yield: 38.4 mg, 52%, off-white solid), m.p. = 116-118 °C; HRMS (ESI) m/z: $[M+Na]^+$ Calcd for $C_{26}H_{17}FN_2ONa$, 415.1217; found, 415.1214; ^{1}H NMR (400 MHz, DMSO-d$_6$ + $CDCl_3$) δ 9.01 (s, 1H), 8.38 (s, 1H), 8.31 (dd, *J* = 8.5, 4.9 Hz, 1H), 8.04 – 7.93 (m, 2H), 7.56 – 7.48 (m, 2H), 7.29 – 7.18 (m, 6H), 7.05 (td, *J* = 8.8, 2.4 Hz, 1H), 1.94 (s, 3H); ^{13}C{^{1}H} NMR (100

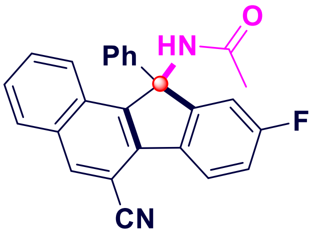

MHz, DMSO-d$_6$ + CDCl$_3$) δ 169.8, 164.2, 161.7(d, J_{C-F} = 250 Hz), 154.5, 154.3 (d, J_{C-F} = 30 Hz), 144.6, 140.3, 136.3, 136.1, 133.0, 132.0, 130.4(d, J_{C-F} = 156 Hz), 129.6, 129.3 (d, J_{C-F} = 32 Hz), 128.7, 128.0, 126.6, 125.7(q, J_{C-F} = 227 Hz), 124.4, 122.6, 122.5 (d, J_{C-F} = 9 Hz), 118.4, 114.8, 114.5(d, J_{C-F} = 22 Hz), 111.0, 110.7 (d, J_{C-F} = 24 Hz), 102.6, 69.2, 23.0.

***N*-(6-cyano-2,3-dimethoxy-11-phenyl-11*H*-benzo[*a*]fluoren-11-yl)acetamide (3qa):** The title compound **3qa** was prepared via procedure 3.2 (ethyl acetate/hexane, 4:6). (yield: 26.69 mg, 41%, off-white solid), m.p. = 297-299 °C; HRMS (ESI) m/z: [M+Na]$^+$ Calcd for $C_{28}H_{22}N_2O_3Na$, 457.1523; found, 457.1520; ^{1}H NMR (400 MHz, DMSO-d$_6$ + CDCl$_3$) 8.89 (s, 1H), 8.31 (d, J = 7.5 Hz, 1H), 8.16 (s, 1H), 7.67 (s, 1H), 7.58 (d, J = 7.5 Hz, 1H), 7.35 (t, J = 7.3 Hz, 1H), 7.28 (d, J = 3.5 Hz, 1H), 7.22 (dt, J = 6.8, 4.9 Hz, 6H), 3.99 (s, 3H), 3.81 (s, 3H), 1.95 (s, 3H); ^{13}C{^{1}H} NMR (100 MHz, DMSO-d$_6$ + CDCl$_3$) δ 169.1, 151.3, 150.9, 149.4, 142.8, 139.9, 137.0, 134.9, 133.3, 128.0, 127.8, 127.6, 127.4, 127.1, 126.6, 125.5, 123.0, 120.4, 118.5, 106.9, 102.9, 10.0, 69.0, 55.8, 55.4, 22.6.

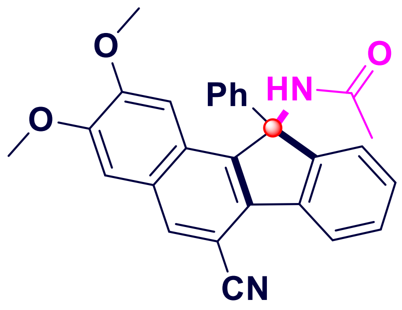

***N*-(6-benzoyl-11-(3-(trifluoromethyl)phenyl)-11*H*-benzo[*a*]fluoren-11-yl)acetamide (3ra):** The title compound **3ra** was prepared via procedure 3.2 (ethyl acetate/hexane, 4:6).(yield: 35.17 mg, 45%, off-white solid), m.p. = 108-110 °C; HRMS (ESI) m/z: [M+Na]$^+$ Calcd for $C_{33}H_{22}F_3NO_2Na$, 544.1495; found, 544.1490; ^{1}H NMR (400 MHz, CDCl$_3$) δ 8.04 (d, J = 7.3 Hz, 2H), 7.93 (dd, J = 9.8, 7.6 Hz, 2H), 7.80 – 7.74 (m, 1H), 7.70 (s, 1H), 7.68 – 7.60 (m, 2H), 7.49 (ddd, J = 13.2, 9.0, 2.4 Hz, 5H), 7.40 (d, J = 7.1 Hz, 1H), 7.30 (t, J = 4.5 Hz, 2H), 7.21 – 7.11 (m, 2H), 6.58 (s, 1H), 2.04 (s, 3H); ^{13}C{^{1}H} NMR (101 MHz, CDCl$_3$) δ 197.4, 169.5, 150.2, 143.2, 141.9, 138.2, 137.0, 136.4, 133.9, 132.9, 132.7, 131.1, 130.8, 130.6 (d, J_{C-F} = 178 Hz), 130.1, 129.6, 129.2, 128.5, 128.2, 128.1, 126.4 (q, J_{C-F} = 309 Hz), 125.2, 124.6 (d, J_{C-F} = 3.0 Hz), 123.3 (d, J_{C-F} = 12 Hz), 122.5 (d, J_{C-F} = 3.0 Hz), 70.2, 24.1.

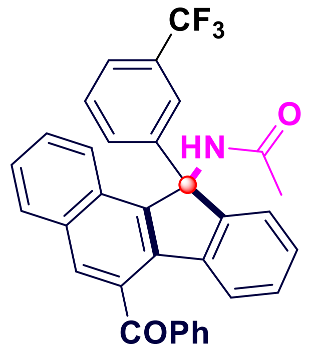

***N*-(6-benzoyl-11-(4-methoxyphenyl)-11*H*-benzo[*a*]fluoren-11-yl)acetamide (3sa):** The title compound **3sa** was prepared via procedure 3.2 (ethyl acetate/hexane, 4:6). (yield: 35.49 mg, 49%, light-brown solid), m.p. = 194-196 °C; HRMS (ESI) m/z: [M+Na]$^+$ Calcd for $C_{33}H_{25}NO_3Na$, 506.1732; found, 506.1730; ^{1}H NMR (400 MHz, CDCl$_3$) δ 8.05 (d, J = 7.1 Hz, 2H), 7.91 (d, J = 8.8 Hz, 2H), 7.82 – 7.77 (m, 1H), 7.62 (t, J = 7.4 Hz, 1H), 7.56 (d, J = 7.6 Hz, 1H), 7.47 (ddd, J = 9.6, 9.2, 3.6 Hz, 4H), 7.35 (d, J = 7.6 Hz, 1H), 7.24 – 7.19 (m, 2H), 7.15 (t, J = 7.0 Hz, 1H), 7.08 (d, J = 6.5 Hz, 1H), 6.81 – 6.74 (m, 2H), 6.66 (s, 1H), 3.73 (s, 3H), 2.00 (s, 3H); ^{13}C{^{1}H} NMR (100 MHz, CDCl$_3$) δ 197.2, 169.1, 151.3, 150.9,

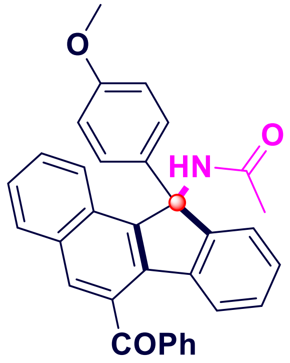

149.4, 142.8, 139.9, 137.0, 134.9, 133.3, 128.0, 127.8, 127.6, 127.4, 127.1, 126.6, 125.5, 123.0, 120.4, 118.5, 106.9, 102.9, 10.0, 69.0, 55.4, 22.6.

***N*-(6-benzoyl-11-phenyl-11H-benzo[*a*]fluoren-11-yl)benzamide (4ab):** The title compound **4ab** was prepared

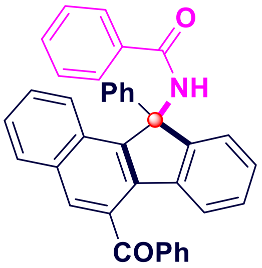

via procedure 3.2 (ethyl acetate/hexane, 4:6). (yield: 63.4 mg, 82%, off-white solid), m.p. = 169-171 °C; HRMS (ESI) m/z: $[M+Na]^+$ Calcd for $C_{37}H_{25}NO_2Na$, 538.1777; found, 538.1775; ^1H NMR (400 MHz, $CDCl_3$) δ 8.09 (dd, *J* = 8.3, 1.3 Hz, 2H), 7.95 (s, 1H), 7.93 – 7.83 (m, 2H), 7.77 (d, *J* = 7.2 Hz, 2H), 7.66 – 7.60 (m, 2H), 7.54 – 7.36 (m, 11H), 7.33 – 7.27 (m, 3H), 7.14 (ddd, *J* = 11.8, 7.4, 1.3 Hz, 2H); $^{13}C\{^1H\}$ NMR (100 MHz, $CDCl_3$) δ 197.8, 150.6, 143.4, 141.3, 138.4, 137.2, 136.8, 134.6, 133.8, 132.9, 132.7, 131.6, 130.7, 130.3, 130.0, 129.1, 128.8, 128.6, 128.1, 127.9, 127.9, 127.0, 126.1, 125.4, 124.0, 123.8, 123.2, 70.1.

***N*-(6-benzoyl-11-phenyl-11*H*-benzo[*a*]fluoren-11-yl)-4-methylbenzamide (4ac):** The title compound **4ac** was

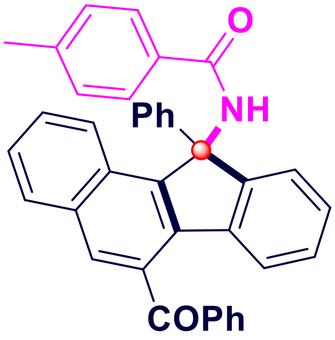

prepared via procedure 3.2 (ethyl acetate/hexane, 4:6). (yield: 67.5 mg, 85%, off-white solid), m.p. = 216-218 °C; HRMS (ESI) m/z: $[M+Na]^+$ Calcd for $C_{38}H_{27}NO_2Na$, 552.1934; found, 552.1931; ^1H NMR (400 MHz, $CDCl_3$) δ 8.09 (dd, *J* = 8.4, 1.3 Hz, 2H), 7.95 (s, 1H), 7.90 (d, *J* = 7.3 Hz, 2H), 7.66 (t, *J* = 5.0 Hz, 2H), 7.62 (dd, *J* = 7.5, 0.9 Hz, 2H), 7.51 (t, *J* = 7.6 Hz, 2H), 7.44 – 7.36 (m, 6H), 7.32 – 7.26 (m, 3H), 7.20 (d, *J* = 7.9 Hz, 2H), 7.14 (ddd, *J* = 12.0, 7.4, 1.4 Hz, 2H), 2.37 (s, 3H); $^{13}C\{^1H\}$ NMR (100 MHz, $CDCl_3$) δ 197.7, 165.8, 150.6, 143.6, 142.1, 141.5, 138.4, 137.2, 136.7, 133.8, 132.9, 132.7, 131.7, 130.7, 130.3, 130.0, 129.4, 129.3, 129.1, 128.8, 128.1, 127.9, 127.8, 127.0, 126.1, 125.4, 124.0, 123.8, 123.2, 70.1, 21.5.

***N*-(6-benzoyl-11-phenyl-11*H*-benzo[*a*]fluoren-11-yl)pivalamide (4ad):** The title compound **4ad** was prepared

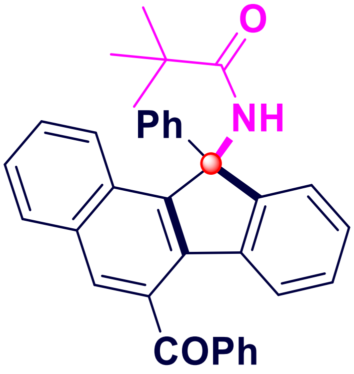

via procedure 3.2 (ethyl acetate/hexane, 4:6). (yield: 60.3 mg, 81%, white solid), m.p. = 181-183 °C; HRMS (ESI) m/z: $[M+Na]^+$ Calcd for $C_{35}H_{29}NO_2Na$, 518.2090; found, 518.2086; ^1H NMR (400 MHz, $CDCl_3$) δ 8.11 – 8.03 (m, 2H), 7.91 (s, 1H), 7.89 (dd, *J* = 6.3, 3.1 Hz, 1H), 7.77 (dd, *J* = 8.0, 1.7 Hz, 1H), 7.64 – 7.58 (m, 1H), 7.52 – 7.46 (m, 3H), 7.45 – 7.36 (m, 3H), 7.29 – 7.21 (m, 5H), 7.16 – 7.06 (m, 2H), 6.96 (s, 1H), 1.19 (s, 9H); $^{13}C\{^1H\}$ NMR (100 MHz, $CDCl_3$) δ 197.6, 176.4, 150.7, 143.8, 141.9, 138.2, 137.2, 136.6, 133.6, 132.9, 132.5, 130.7, 130.2, 129.9, 129.3, 129.1, 128.7, 127.9, 127.8, 127.7, 127.5, 126.0, 125.1, 123.8, 123.2, 123.0, 69.3, 39.2, 27.7.

***N*-(6-benzoyl-11-phenyl-11*H*-benzo[*a*]fluoren-11-yl)isobutyramide (4ae):** The title compound **4ae** was prepared via procedure 3.2 (ethyl acetate/hexane, 4:6). (yield: 55.6 mg, 77%, white solid), m.p. = 214-216 °C; HRMS (ESI) m/z: $[M+Na]^+$ Calcd for $C_{34}H_{27}NO_2Na$, 504.1934; found, 504.1934; ^{1}H NMR (400 MHz, $CDCl_3$) δ 8.11 – 8.02 (m, 2H), 7.95 – 7.85 (m, 2H), 7.83 – 7.76 (m, 1H), 7.65 – 7.58 (m, 1H), 7.56 – 7.46 (m, 3H), 7.45 – 7.36 (m, 3H), 7.31 – 7.19 (m, 5H), 7.11 (dtd, *J* = 21.5, 7.4, 1.2 Hz, 2H), 6.73 (s, 1H), 2.42 (dd, *J* = 13.8, 6.9 Hz, 1H), 1.15 (d, *J* = 6.9 Hz, 3H), 1.04 (d, *J* = 6.9 Hz, 3H); ^{13}C{^{1}H} NMR (100 MHz, $CDCl_3$) δ 197.6, 175.3, 150.8, 143.8, 141.5, 138.2, 137.2, 136.5, 133.7, 132.9, 132.6, 130.7, 130.2, 129.9, 129.3, 128.9, 128.7, 127.9, 127.8, 127.6, 126.0, 125.3, 123.8, 123.6, 123.1, 69.6, 36.0, 19.7, 19.6.

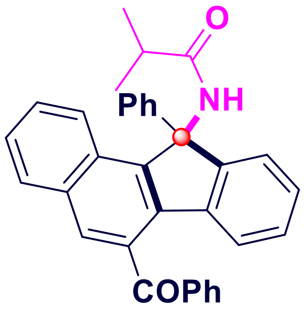

***N*-(6-benzoyl-11-phenyl-11H-benzo[*a*]fluoren-11-yl)propionamide (4af):** The title compound **4af** was prepared via procedure 3.2 (ethyl acetate/hexane, 4:6). (yield: 51.9 mg, 74%, white solid), m.p. = 217-219 °C; HRMS (ESI) m/z: $[M+Na]^+$ Calcd for $C_{33}H_{25}NO_2Na$, 490.1777; found, 490.1773; ^{1}H NMR (400 MHz, $CDCl_3$) δ 8.05 (d, *J* = 7.4 Hz, 2H), 7.95 – 7.85 (m, 2H), 7.78 (d, *J* = 8.0 Hz, 1H), 7.65 – 7.35 (m, 7H), 7.31 – 7.18 (m, 5H), 7.10 (ddd, *J* = 13.9, 10.8, 6.7 Hz, 2H), 6.74 (s, 1H), 2.32 – 2.13 (m, 2H), 1.06 (t, *J* = 7.5 Hz, 3H); ^{13}C{^{1}H} NMR (100 MHz, $CDCl_3$) δ 197.7, 172.3, 150.7, 143.8, 141.3, 138.2, 137.2, 136.5, 133.7, 132.9, 132.6, 130.6, 130.1, 129.9, 129.2, 128.9, 128.7, 127.9, 127.7, 126.0, 125.4, 123.9, 123.8, 123.1, 69.8, 30.1, 9.7.

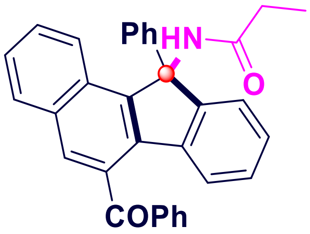

***N*-(6-benzoyl-11-phenyl-11*H*-benzo[*a*]fluoren-11-yl)cyclohexanecarboxamide (4ag):** The title compound **4ag** was prepared via procedure 3.2 (ethyl acetate/hexane, 4:6). (yield: 58.7 mg, 75%, white solid), m.p. = 293-295 °C; HRMS (ESI) m/z: $[M+Na]^+$ Calcd for $C_{37}H_{31}NO_2Na$, 544.2247; found, 544.2246; ^{1}H NMR (400 MHz, DMSO-d_6 + $CDCl_3$) δ 8.85 (s, 1H), 8.09 – 7.94 (m, 4H), 7.91 (s, 1H), 7.73 – 7.61 (m, 1H), 7.59 – 7.41 (m, 5H), 7.29 (dd, *J* = 8.0, 1.7 Hz, 2H), 7.25 – 7.19 (m, 3H), 7.11 (td, *J* = 7.5, 1.0 Hz, 1H), 7.02 (td, *J* = 7.6, 1.1 Hz, 1H), 2.45 (dd, *J* = 15.5, 7.1 Hz, 1H), 1.89 – 1.68 (m, 2H), 1.67 – 1.49 (m, 3H), 1.32 – 1.15 (m, 3H), 1.13 – 0.96 (m, 2H); ^{13}C{^{1}H} NMR (100 MHz, DMSO-d_6 + $CDCl_3$) δ 196.7, 174.6, 151.9, 144.4, 141.4, 137.4, 136.6, 134.9, 133.4, 132.1, 131.7, 129.8, 129.1, 128.9, 128.5, 128.4, 128.0, 127.0, 127.0, 126.9, 126.6, 125.7, 125.5, 124.2, 122.3, 122.0, 68.8, 43.6, 40.1, 29.0, 28.8, 25.2, 25.1, 25.1.

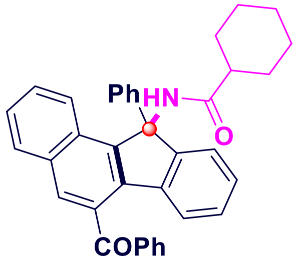

N-(6-benzoyl-11-phenyl-11H-benzo[*a*]fluoren-11-yl)acrylamide (4ah): The title compound **4ah** was prepared

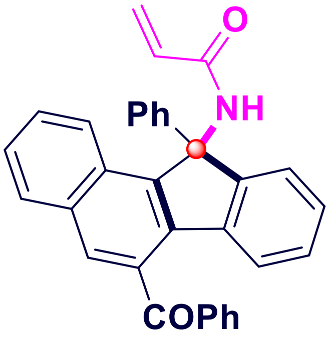

via procedure 3.2 (ethyl acetate/hexane, 4:6). (yield: 48.9 mg, 70%, off-white solid), m.p. = 225-227 °C; HRMS (ESI) m/z: $[M+Na]^+$ Calcd for $C_{33}H_{23}NO_2Na$, 488.1621; found, 488.1619; ^{1}H NMR (400 MHz, $CDCl_3$) δ 8.12 – 8.03 (m, 2H), 7.93 (s, 1H), 7.92 – 7.87 (m, 1H), 7.77 (dd, J = 8.2, 0.9 Hz, 1H), 7.65 – 7.57 (m, 2H), 7.52 – 7.47 (m, 2H), 7.46 – 7.37 (m, 3H), 7.33 – 7.28 (m, 2H), 7.27 – 7.23 (m, 3H), 7.12 (dtd, J = 20.8, 7.4, 1.2 Hz, 2H), 6.85 (s, 1H), 6.17 (d, J = 4.8 Hz, 2H), 5.65 – 5.52 (m, 1H); ^{13}C{^{1}H} NMR (100 MHz, $CDCl_3$) δ 197.6, 164.1, 150.4, 143.4, 141.0, 138.2, 137.1, 136.6, 133.8, 132.8, 132.6, 130.7, 130.5, 130.3, 129.9, 129.2, 129.0, 128.7, 128.1, 127.9, 127.3, 126.1, 125.4, 124.1, 123.8, 123.1, 70.1.

***N*-(6-benzoyl-11-phenyl-11*H*-benzo[*a*]fluoren-11-yl)cinnamamide (4ai):** The title compound **4ai** was

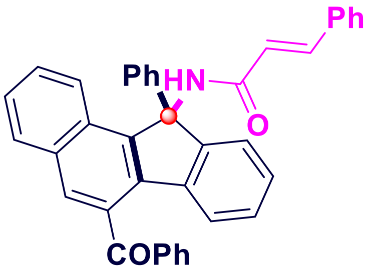

prepared via procedure 3.2 (ethyl acetate/hexane, 4:6). (yield: 64.2 mg, 79%, brown solid), m.p.= 205-207 °C; HRMS (ESI) m/z: $[M+Na]^+$ Calcd for $C_{39}H_{27}NO_2Na$, 564.1934; found, 564.1932; ^{1}H NMR (400 MHz, $CDCl_3$) δ 8.08 (d, J = 7.3 Hz, 2H), 7.94 (s, 1H), 7.92 – 7.87 (m, 1H), 7.86 – 7.78 (m, 1H), 7.64 (dd, J = 10.5, 4.3 Hz, 2H), 7.50 (dd, J = 13.9, 6.2 Hz, 3H), 7.42 (s, 4H), 7.38 – 7.25 (m, 8H), 7.13 (dt, J = 21.8, 7.1 Hz, 3H), 6.90 (s, 1H), 6.51 (d, J = 15.6 Hz, 1H); ^{13}C{^{1}H} NMR (100 MHz, $CDCl_3$) δ 197.8, 164.6, 150.6, 143.6, 142.1, 141.1, 141.1, 138.3, 138.3, 137.2, 136.6, 134.6, 133.8, 132.9, 132.7, 130.7, 130.2, 129.9, 129.8, 129.3, 129.0, 128.8, 128.1, 127.9, 127.8, 126.1, 125.5, 124.2, 123.9, 123.1, 120.1, 70.2.

ethyl-11-phenyl-11-(2-phenylacetamido)-11*H*-benzo[*a*]fluorene-6-carboxylate (4nj): The title compound **4nj**

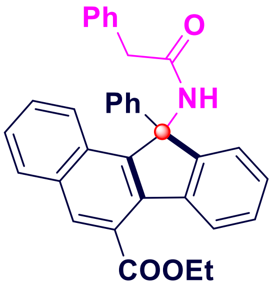

was prepared via procedure 3.2 (ethyl acetate/hexane, 4:6). (yield: 53.0 mg, 71%, white solid), m.p.= 223-225 °C; HRMS (ESI) m/z: $[M+Na]^+$ Calcd for $C_{34}H_{27}NO_3Na$, 520.1883; found, 520.1880; ^{1}H NMR (400 MHz, $CDCl_3$) δ 8.37 (s, 1H), 8.18 (d, J = 7.7 Hz, 1H), 7.94 (d, J = 8.2 Hz, 1H), 7.44 – 7.36 (m, 4H), 7.31 (t, J = 11.3 Hz, 4H), 7.26 – 7.18 (m, 3H), 7.12 (d, J = 6.9 Hz, 3H), 6.96 (d, J = 6.5 Hz, 2H), 6.65 (s, 1H), 4.56 (dt, J = 7.0, 3.9 Hz, 2H), 3.47 (dd, J = 49.3, 16.0 Hz, 2H), 1.51 (t, J = 7.1 Hz, 3H); ^{13}C{^{1}H} NMR (100 MHz, $CDCl_3$) δ 169.0, 168.2, 150.5, 143.9, 141.4, 138.5, 137.0, 135.1, 132.6, 132.5, 130.1, 129.6, 129.5, 129.2, 128.9, 128.1, 128.0, 127.8, 127.7, 127.6, 125.9, 125.7, 124.7, 124.4, 123.7, 123.2, 69.3, 61.5, 44.5, 14.4.

ethyl-11-phenyl-11-(4-phenylbutanamido)-11*H*-benzo[*a*]fluorene-6-carboxylate (4nk): The title compound **4nk** was prepared via procedure 3.2 (ethyl acetate/hexane, 4:6). (yield: 59.9 mg, 76%, white solid), m.p.= 216-218 °C; HRMS (ESI) m/z: $[M+Na]^+$ Calcd for $C_{36}H_{31}NO_3Na$, 548.2196; found, 548.2196; ^1H NMR (400 MHz, $CDCl_3$) δ 8.38 (s, 1H), 8.18 (d, *J* = 7.8 Hz, 1H), 7.95 (d, *J* = 7.9 Hz, 1H), 7.70 (d, *J* = 8.1 Hz, 1H), 7.56 (d, *J* = 7.2 Hz, 1H), 7.40 (dt, *J* = 14.9, 7.0 Hz, 2H), 7.31 (t, *J* = 7.4 Hz, 1H), 7.26 – 7.12 (m, 9H), 7.03 (d, *J* = 7.2 Hz, 2H), 6.62 (s, 1H), 4.56 (d, *J* = 4.9 Hz, 2H), 2.46 (t, *J* = 7.0 Hz, 2H), 2.26 – 2.06 (m, 2H), 1.82 (d, *J* = 6.4 Hz, 2H), 1.51 (t, *J* = 7.0 Hz, 3H); $^{13}C\{^1H\}$ NMR (100 MHz, $CDCl_3$) δ 171.2, 168.2, 150.8, 144.3, 141.3, 141.3, 138.4, 136.8, 132.6, 132.6, 130.1, 129.7, 128.9, 128.4, 128.3, 128.2, 128.0, 127.9, 127.7, 125.9, 125.8, 125.8, 125.4, 124.3, 123.9, 123.7, 69.7, 61.6, 36.0, 34.8, 26.7, 14.4.

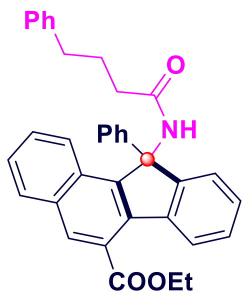

ethyl-11-benzamido-11-phenyl-11*H*-benzo[*a*]fluorene-6-carboxylate (4nb): The title compound **4nb** was prepared via procedure 3.2 (ethyl acetate/hexane, 4:6). (yield: 63.1 mg, 87%, white solid), m.p.= 207-209 °C; HRMS (ESI) m/z: $[M+Na]^+$ Calcd for $C_{33}H_{25}NO_3Na$, 506.1727; found, 506.1725; ^1H NMR (400 MHz, $CDCl_3$) δ 8.41 (s, 1H), 8.23 (d, *J* = 7.8 Hz, 1H), 7.99 – 7.93 (m, 1H), 7.81 (d, *J* = 7.9 Hz, 1H), 7.73 (d, *J* = 7.4 Hz, 2H), 7.64 (d, *J* = 7.3 Hz, 1H), 7.49 – 7.38 (m, 5H), 7.36 – 7.32 (m, 3H), 7.29 – 7.22 (m, 5H), 4.64 – 4.52 (m, 2H), 1.52 (t, *J* = 7.1 Hz, 3H); $^{13}C\{^1H\}$ NMR (100 MHz, $CDCl_3$) δ 168.2, 165.6, 150.6, 144.0, 141.5, 138.6, 137.0, 134.5, 132.8, 132.6, 131.6, 130.2, 129.9, 129.0, 128.6, 128.3, 128.2, 128.0, 127.9, 126.9, 126.0, 125.8, 125.4, 124.4, 123.8, 123.8, 69.9, 61.6, 14.4.

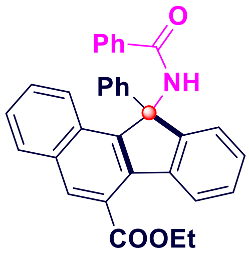

***N*-(6-cyano-11-phenyl-11*H*-benzo[*a*]fluoren-11-yl)propionamide (4of):** The title compound **4of** was prepared via procedure 3.2 (ethyl acetate/hexane, 4:6). (yield: 40.2 mg, 69%, off-white solid), m.p.= 240-242 °C; HRMS (ESI) m/z: $[M+Na]^+$ Calcd for $C_{27}H_{20}N_2ONa$, 411.1468; found, 411.1465; ^1H NMR (400 MHz, $CDCl_3$ + DMSO-d_6) δ 9.01 (s, 1H), 8.44 (s, 1H), 8.32 (d, *J* = 7.5 Hz, 1H), 8.08 – 7.97 (m, 2H), 7.91 (s, 1H), 7.58 – 7.47 (m, 3H), 7.36 (td, *J* = 7.5, 1.2 Hz, 1H), 7.28 (td, *J* = 7.5, 1.1 Hz, 1H), 7.24 – 7.19 (m, 4H), 2.24 (ddt, *J* = 19.6, 15.1, 7.6 Hz, 2H), 0.86 (t, *J* = 7.6 Hz, 3H); $^{13}C\{^1H\}$ NMR (100 MHz, DMSO-d_6 + $CDCl_3$) δ 173.2, 152.2, 145.1, 141.2, 137.1, 136.7, 136.3, 132.3, 130.6, 129.8, 129.3, 128.7, 128.6, 127.8, 126.8, 125.9, 124.7, 123.1, 121.2, 118.6, 102.9, 69.4, 28.9, 9.7.

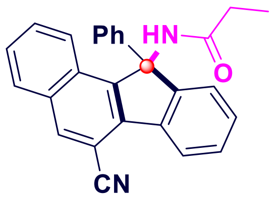

***N*-(6-benzoyl-11-phenyl-11*H*-benzo[*a*]fluoren-11-yl)-N-benzylacetamide (6aa):** A clean flame-dried round bottom flask vial (10 mL) equipped with a magnetic stir bar was added to **3aa** (0.1 mmol, 1.0 equiv.), and 2.0 mL of DMF. Next, NaH (2.0 equiv) was added at room temperature, and then solution was stirred for 1 hours at room temptation. Finally, benzyl bromide (**5a**) added and continue the reaction for 24 h. The progress of the reaction was monitored by thin layer chromatography. When the reaction was complete, water was added to quench

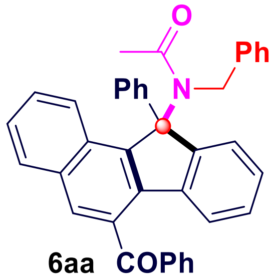

6aa

the reaction mixture, followed by extraction with ethyl acetate (3×10 mL). Finally, the combined organic layer was dried over sodium sulfate, filtered, and concentrated under vacuum. The residue was purified by column chromatography (Hex/EA = 70:30) on silica gel to afford the corresponding **6aa** derivative as a white solid in 79% yield, (64.4 mg), m.p.= 139-141 °C; HRMS (ESI) m/z: $[M+Na]^+$ Calcd for $C_{39}H_{29}NO_2Na$, 566.2090; found, 566.2089; ^1H NMR (400 MHz, $CDCl_3$) δ 8.08 – 7.99 (m, 2H), 7.92 (s, 1H), 7.84 (d, *J* = 8.2 Hz, 1H), 7.74 – 7.61 (m, 2H), 7.56 – 7.26 (m, 12H), 7.22 – 7.06 (m, 6H), 5.49 (q, *J* = 12.7 Hz, 2H), 1.02 (s, 3H); $^{13}C\{^1H\}$ NMR (100 MHz, $CDCl_3$) δ 197.5, 160.7, 153.7, 148.0, 145.8, 138.3, 138.0, 137.4, 135.5, 133.8, 132.4, 132.2, 130.5, 129.8, 129.4, 129.0, 128.7, 128.4, 128.2, 128.1, 127.8, 127.5, 126.4, 126.2, 126.0, 124.2, 123.2, 72.3, 67.0, 16.8.

***N*-(6-benzoyl-11-phenyl-11H-benzo[a]fluoren-11-yl)acetamide-2,2,2-d$_3$ (3aa-*d*$_3$):** The title compound **3aa-*d*$_3$** was prepared via procedure 3.2 (ethyl acetate/hexane, 4:6). (yield: 56.8 mg, 83%, white solid), m.p.= 264-266 °C; HRMS (ESI) m/z: $[M+Na]^+$ Calcd for $C_{32}H_{20}D_3NO_2Na$, 479.1809; found, 479.1808; ^1H NMR (400 MHz, $CDCl_3$) δ 8.10 – 8.01 (m, 2H), 7.94 – 7.86 (m, 2H), 7.80 – 7.75 (m, 1H), 7.61 (t, *J* = 7.4 Hz, 1H), 7.55 (d, *J* = 7.3 Hz, 1H), 7.52 – 7.35 (m, 5H), 7.28 (dt, *J* = 5.3, 3.3 Hz, 2H), 7.24 – 7.19 (m, 3H), 7.10 (dtd, *J* = 24.6, 7.4, 0.9 Hz, 2H), 6.83 (s, 1H); $^{13}C\{^1H\}$ NMR (100 MHz, $CDCl_3$) δ 197.7, 168.9, 150.7, 143.7, 141.1, 138.3, 137.2, 136.6, 133.8, 132.9, 132.7, 130.7, 130.0, 129.9, 129.2, 128.9, 128.8, 127.9, 127.8, 127.8, 126.1, 125.5, 124.0, 123.9, 123.1, 70.0.

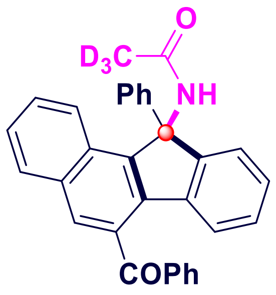

5. Crystal data for compound 4nb and 4of (ellipsoid contour 50% probability levels)

The crystal of **4nb** and **4of** were obtained by crystallization from a solution in ethyl acetate (**4nb**) and chloroform (**4of**) after purification by column chromatography respectively. The instrumentation used for the crystal measurement is Rigaku Oxford XtaLab Proll. The crystallographic data were deposited with the Cambridge Crystallographic Data Centre as supplementary publication with CCDC numbers: **4nb** (2446334), **4of** (2446543).

6. Reference

1. (a) Liu, L.; Wei, L.; Zhang, J. A Facile Route to Polysubstituted Naphthalenes and Benzofluorenols via Scandium Triflate- and Triflic Acid-Catalyzed Benzannulation of 2-(2-Alkynylarylidene)-1,3-Dicarbonyl Compounds. *Adv. Synth. Catal*. **2010**, *352*, 1920-1924. (b) Gore, B. S.; Chiang, C.-H.; Lee, C. C.; Shih, Y.-L.; Wang J.-J. De Novo Protocol for the Construction of Benzo[*a*]fluorenes via Nitrile/Alkene Activation. *Org. Lett.* **2020**, *22*, 7848-7852. (c) Gore B. S.; Lin J.-H.; Wang, J.-J. Unraveling innate substrate-controlled arylation and bicyclization of 1,5-enynes with α,β conjugates: synthesis of substituted benzo[*a*]fluorenes. *Green Chem*. **2021**, *23*, 4144-4149. (d) Liu, L.; Zhang, J. Selectivity-Control in Lewis-acid Catalyzed Regiodivergent Tandem Cationic Cyclization/Ring Expansion Terminated by Pinacol Rearrangement. *Angew. Chem. Int. Ed.* **2009**, *48*, 6093-6096.

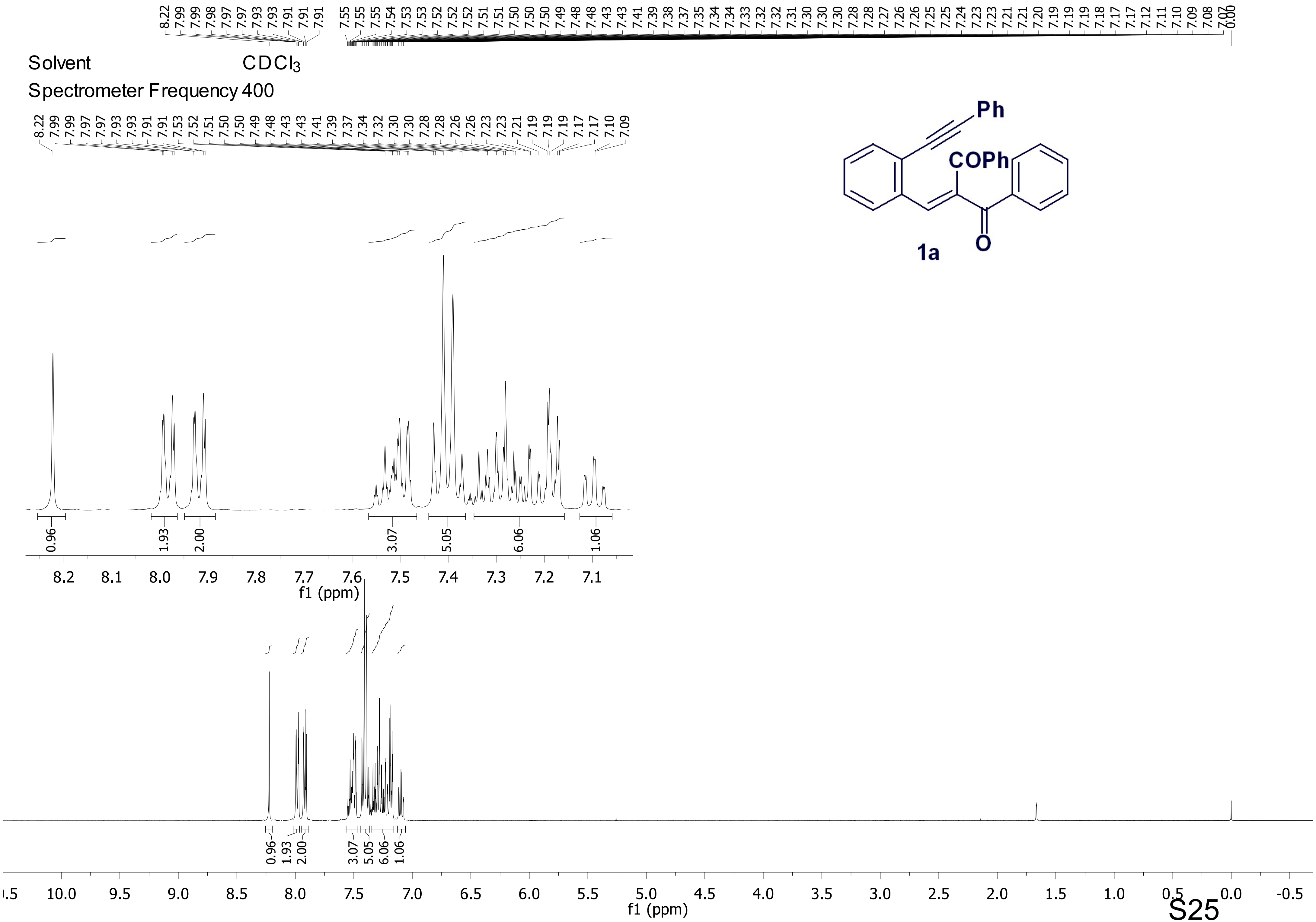
Solvent CDCl3
Spectrometer Frequency 400
Ph
COPh
O
1a
0.96
1.93
2.00
3.07
5.05
6.06
1.06
f1 (ppm)

Solvent CDCl$_3$
Spectrometer Frequency 100

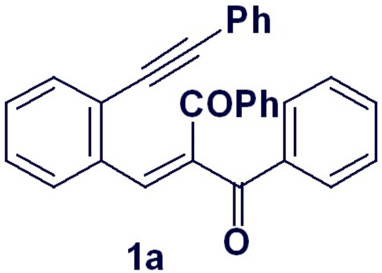

1a

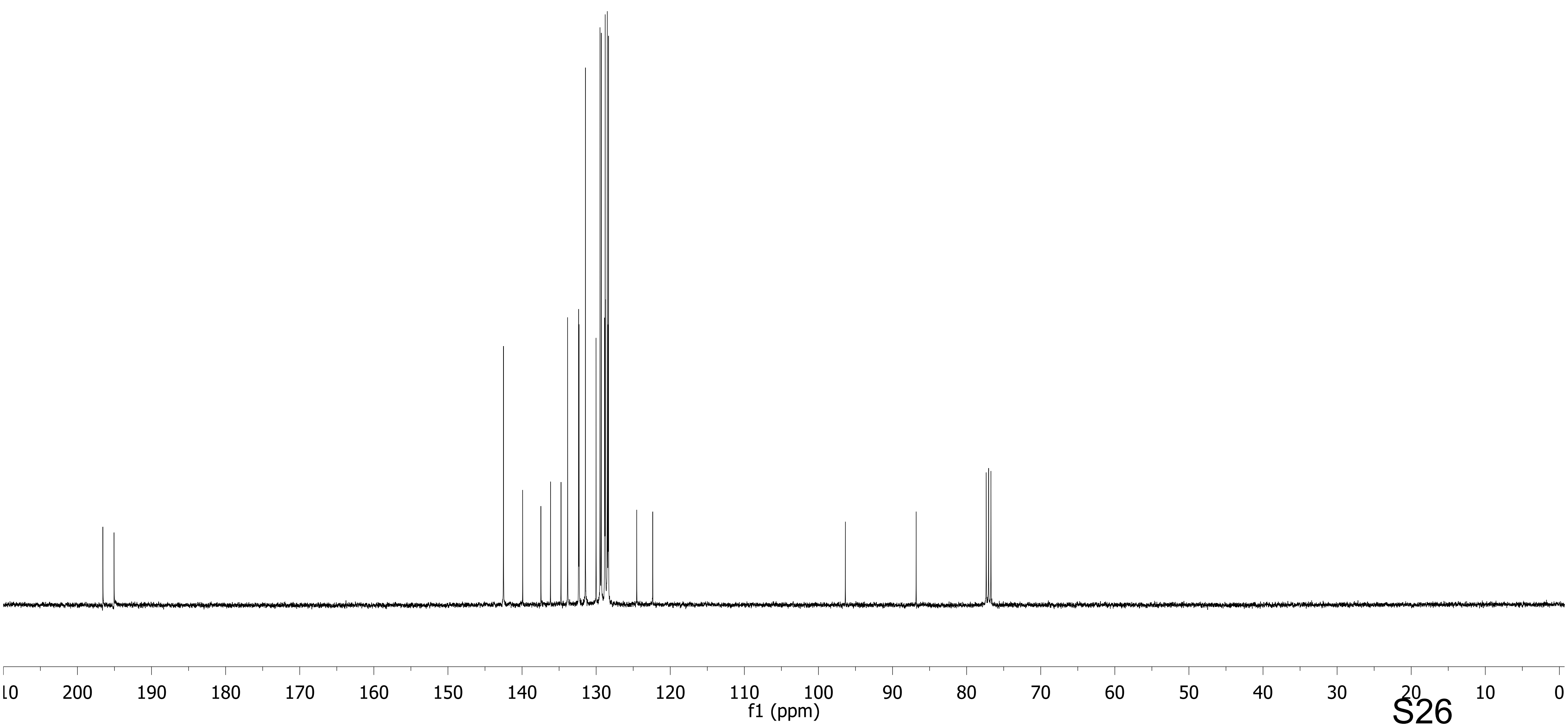

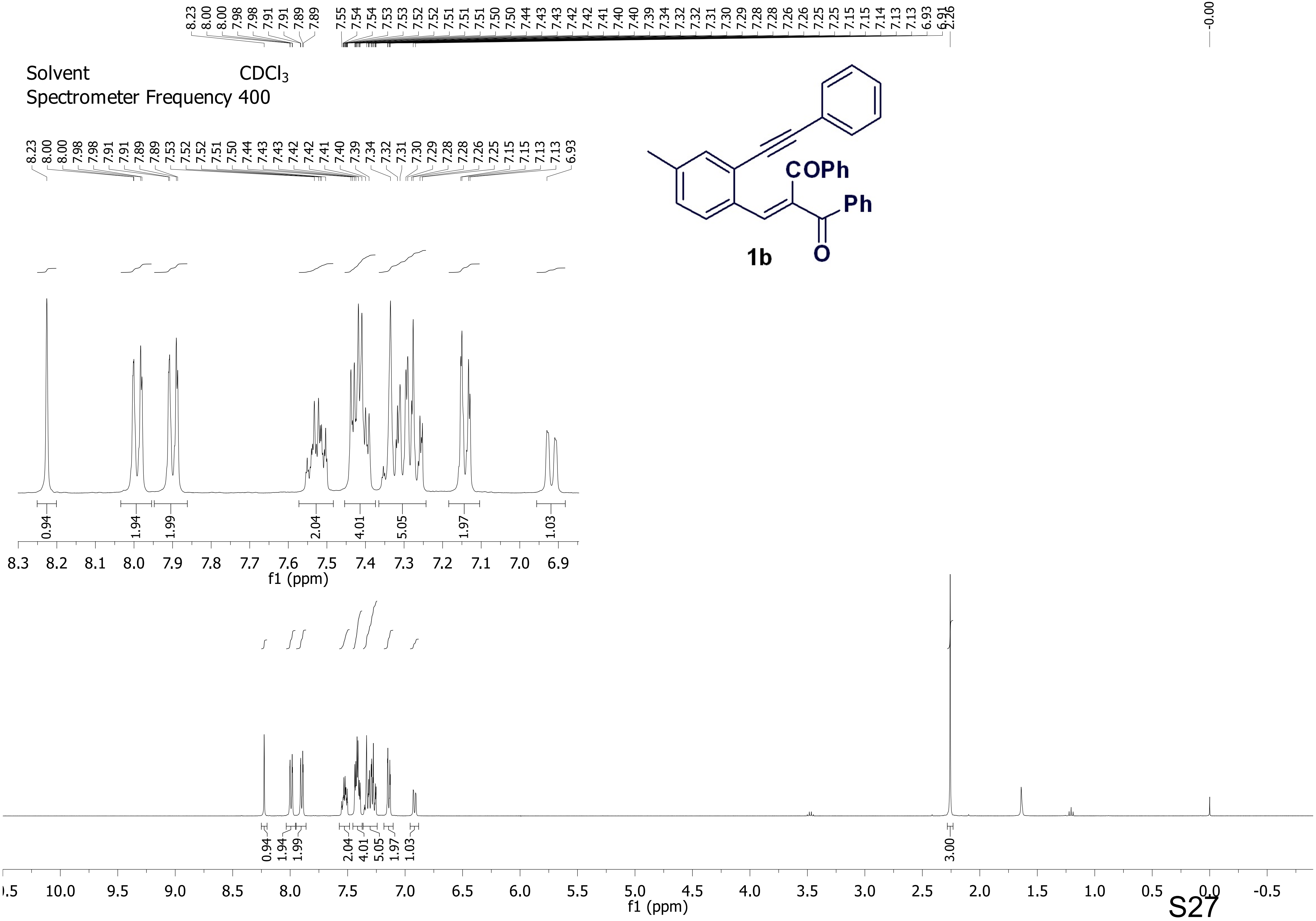
Solvent CDCl$_3$
Spectrometer Frequency 400
COPh
Ph
O
1b
f1 (ppm)
f1 (ppm)

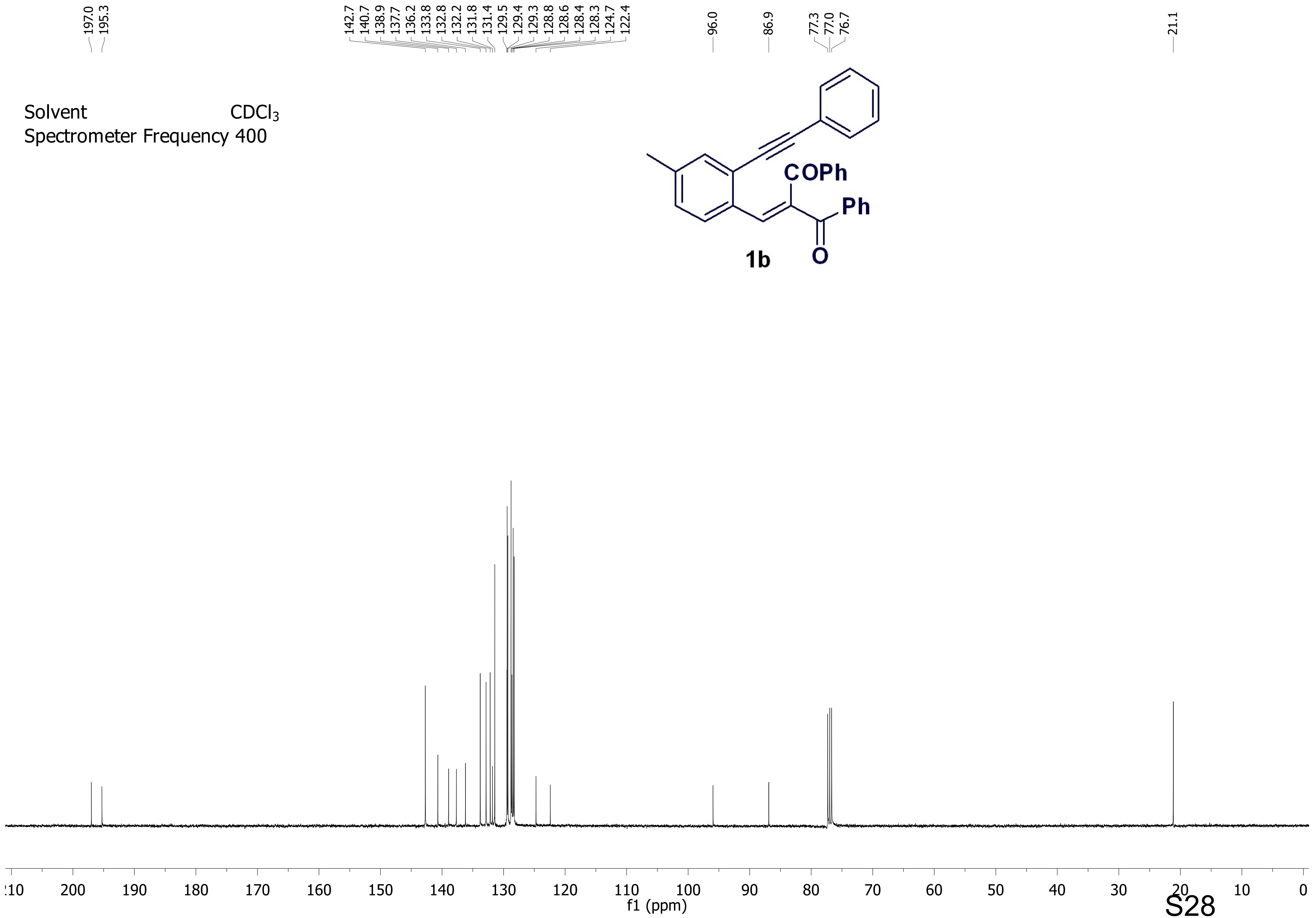
197.0
195.3
142.7
140.7
138.9
137.7
136.2
133.8
132.8
132.2
131.8
131.4
129.5
129.4
129.3
128.8
128.6
128.4
128.3
124.7
122.4
96.0
86.9
77.3
77.0
76.7
21.1
Solvent CDCl3
Spectrometer Frequency 400
COPh
Ph
O
1b
210 200 190 180 170 160 150 140 130 120 110 100 90 80 70 60 50 40 30 20 10 0
f1 (ppm)

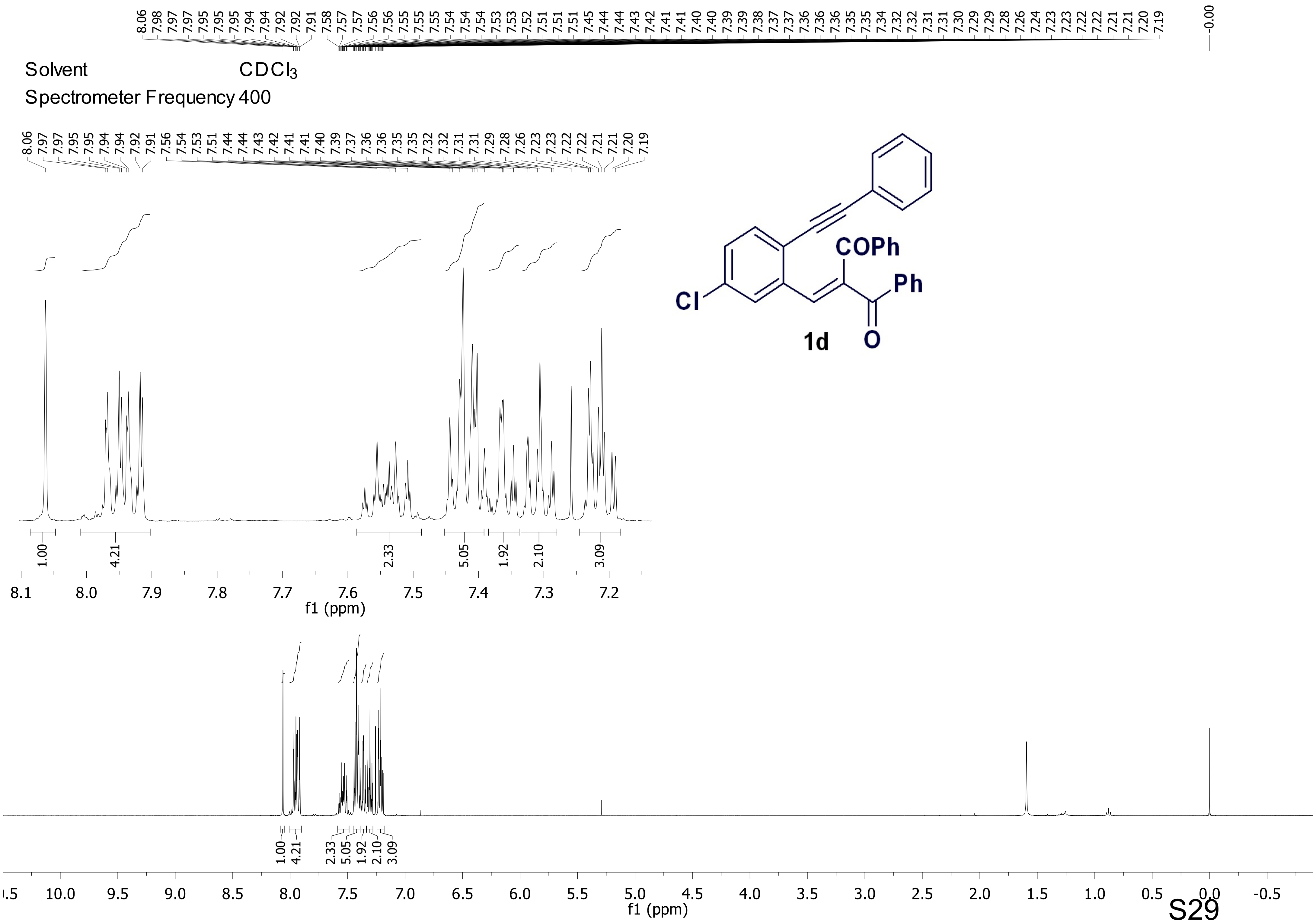

Solvent CDCl$_3$
Spectrometer Frequency 400
COPh
Ph
Cl
O
1d
f1 (ppm)

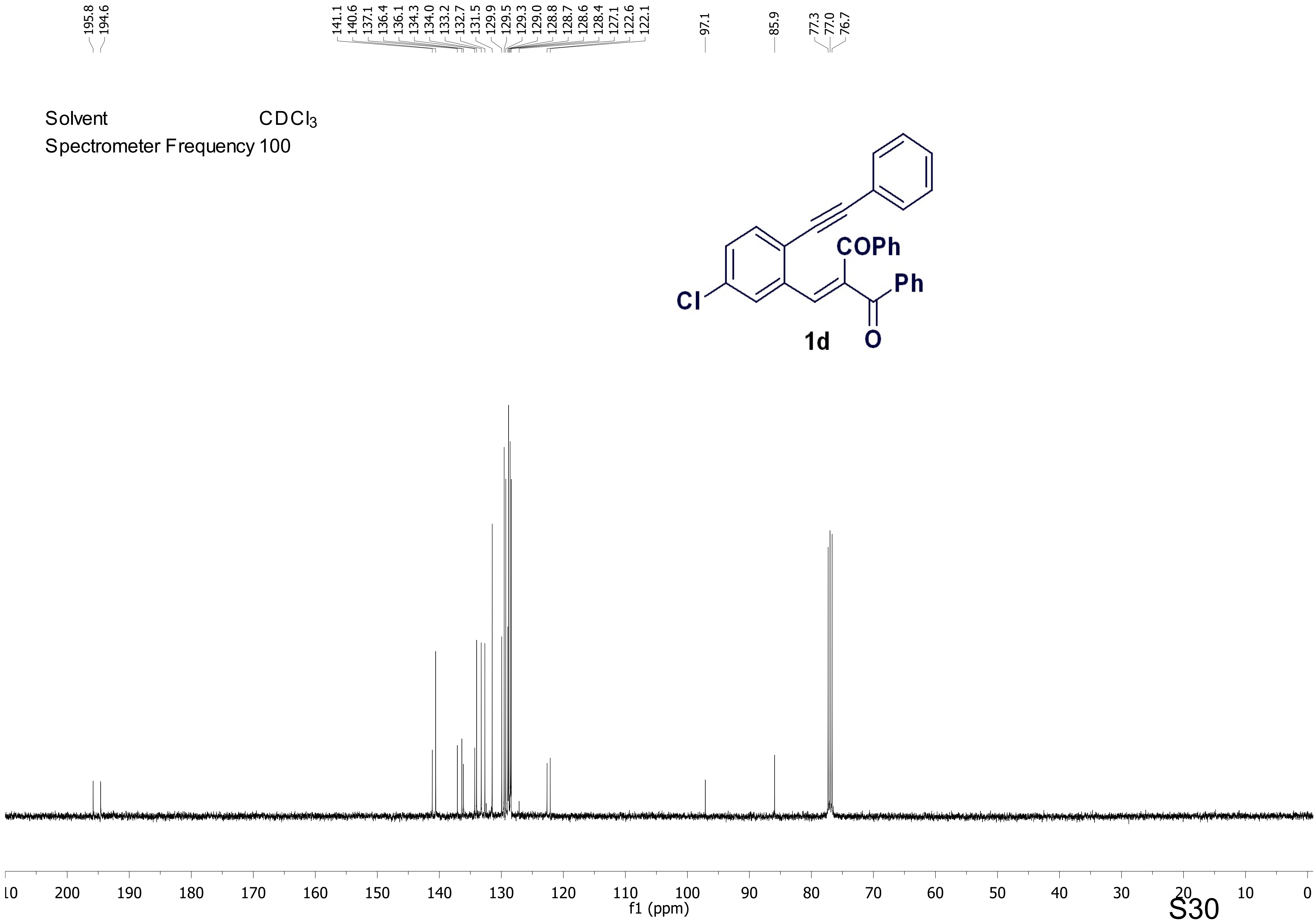
195.8
194.6
141.1
140.6
137.1
136.4
136.1
134.3
134.0
133.2
132.7
131.5
129.9
129.5
129.3
129.0
128.8
128.7
128.6
128.4
127.1
122.6
122.1
97.1
85.9
77.3
77.0
76.7
Solvent CDCl3
Spectrometer Frequency 100
COPh
Ph
Cl
O
1d
f1 (ppm)

Solvent CDCl$_3$
Spectrometer Frequency 400

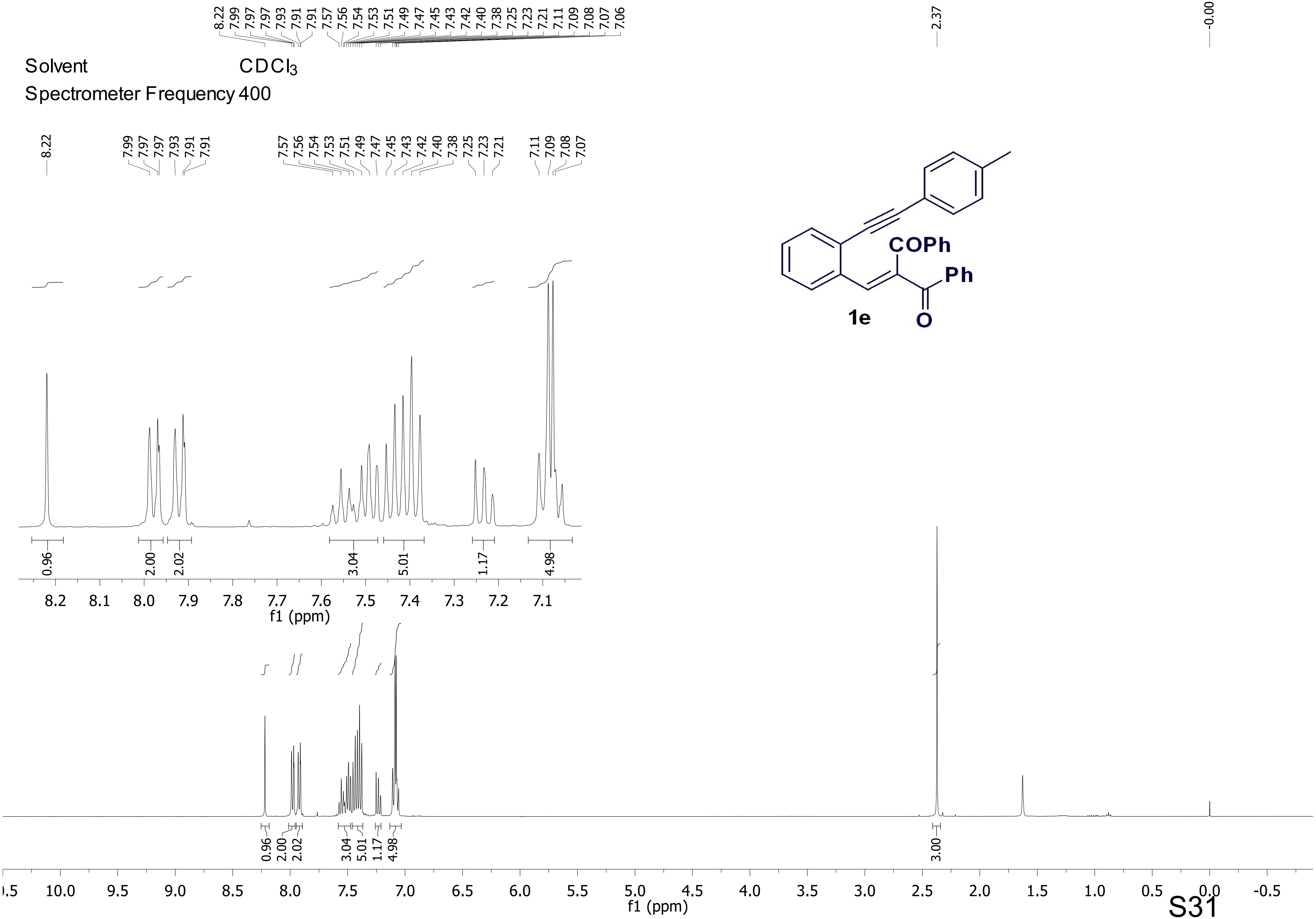

1e

Solvent CDCl$_3$
Spectrometer Frequency 100

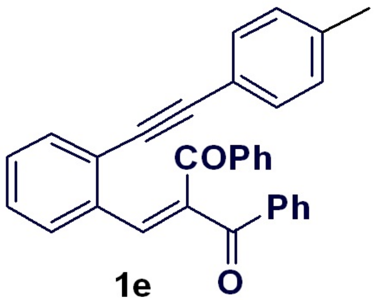

1e

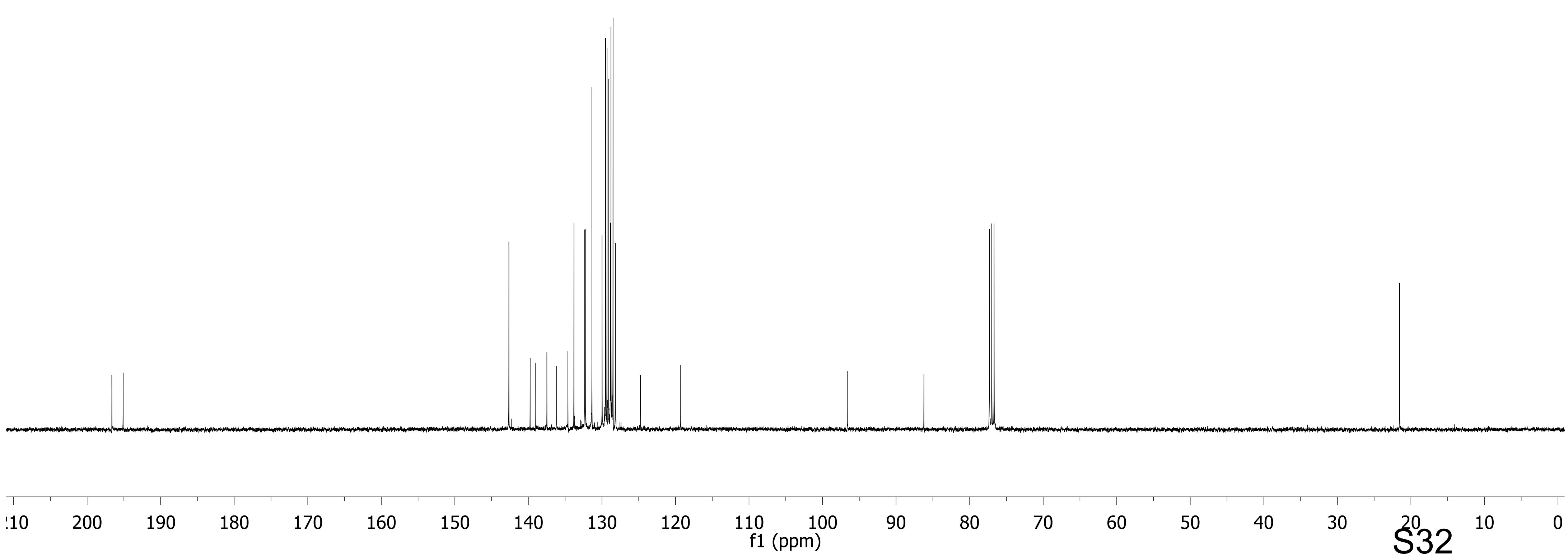

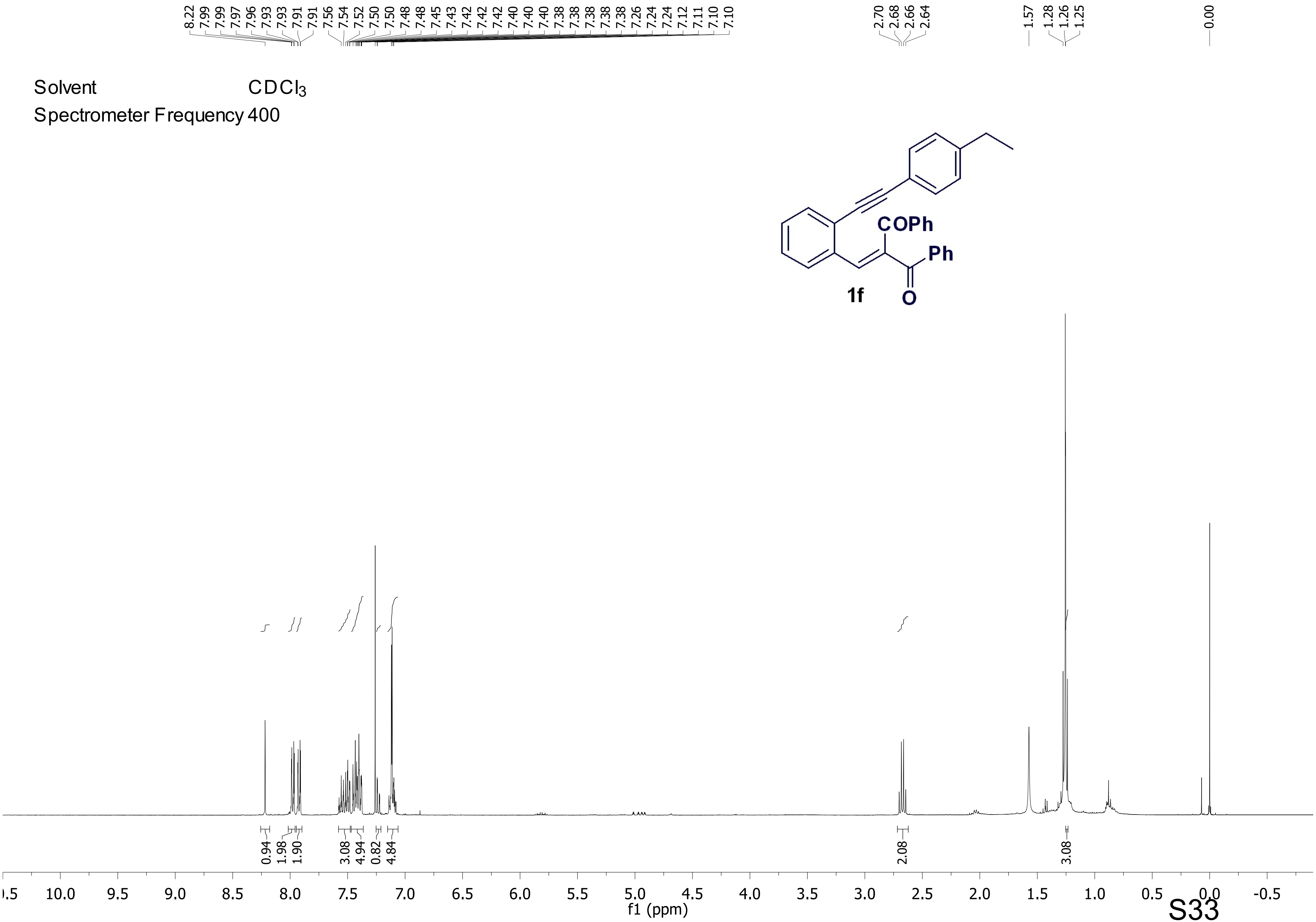

8.22 7.99 7.99 7.97 7.96 7.93 7.93 7.91 7.91 7.56 7.54 7.52 7.50 7.50 7.48 7.48 7.45 7.43 7.42 7.42 7.42 7.40 7.40 7.40 7.38 7.38 7.38 7.38 7.26 7.24 7.24 7.12 7.11 7.10 7.10
2.70 2.68 2.66 2.64
1.57 1.28 1.26 1.25
0.00
Solvent CDCl3
Spectrometer Frequency 400
COPh
Ph
O
1f
0.94 1.98 1.90 3.08 4.94 0.82 4.84
2.08
3.08
10.0 9.5 9.0 8.5 8.0 7.5 7.0 6.5 6.0 5.5 5.0 4.5 4.0 3.5 3.0 2.5 2.0 1.5 1.0 0.5 0.0 -0.5
f1 (ppm)

Solvent CDCl$_3$

Spectrometer Frequency 100

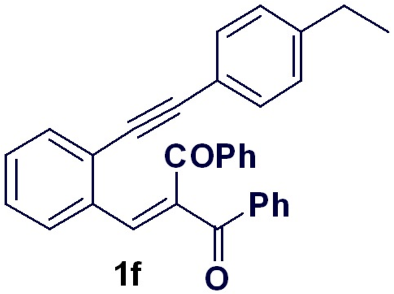

1f

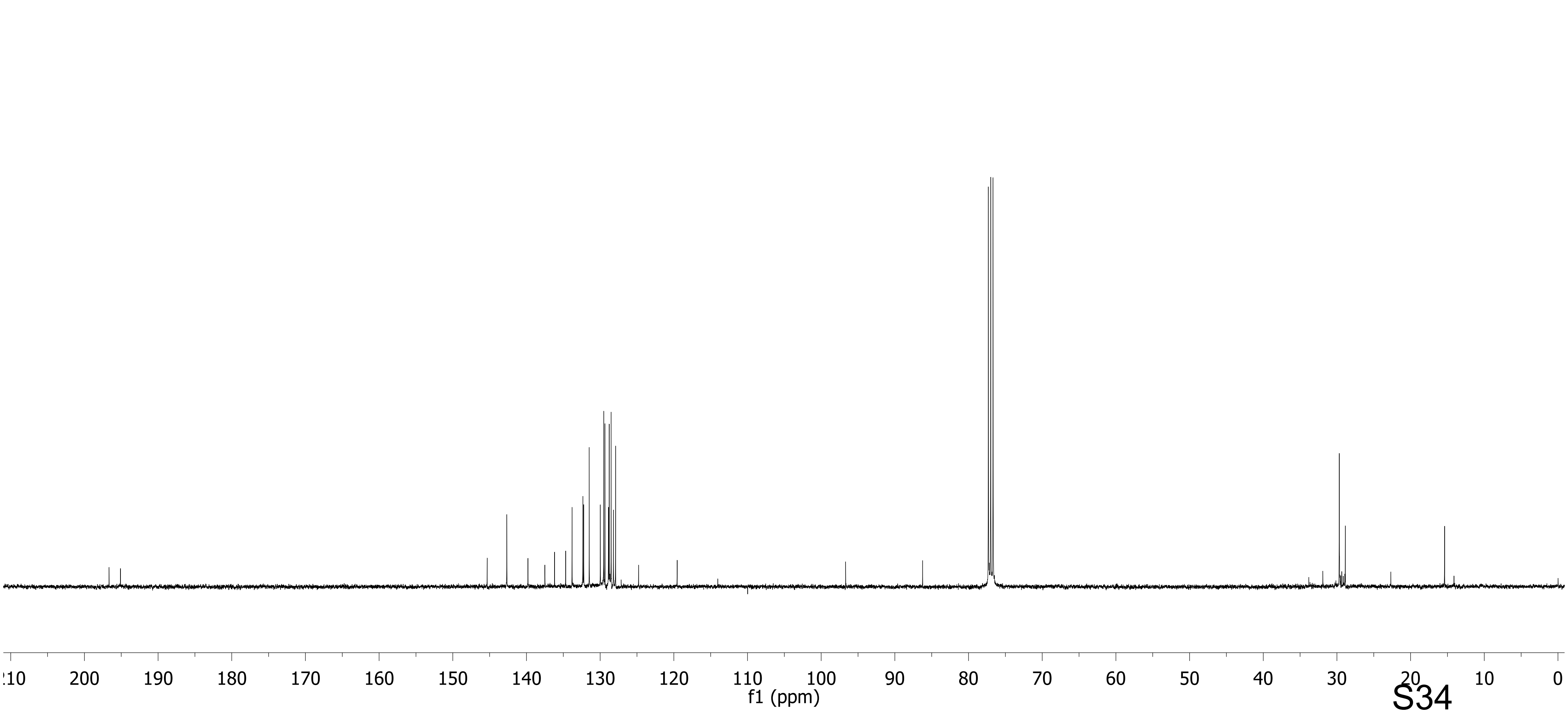

8.17 7.99 7.99 7.98 7.97 7.97 7.92 7.92 7.91 7.90 7.90 7.57 7.55 7.54 7.53 7.52 7.51 7.51 7.51 7.50 7.50 7.47 7.46 7.45 7.43 7.41 7.41 7.40 7.39 7.28 7.28 7.27 7.26 7.26 7.26 7.25 7.24 7.24 7.23 7.16 7.15 7.14 7.13 7.06 7.05 7.05 7.05 6.99 6.99 6.99 6.97 6.97 6.97 6.85 6.84 6.84 6.82 1.60 -0.00

Solvent CDCl$_3$
Spectrometer Frequency 400

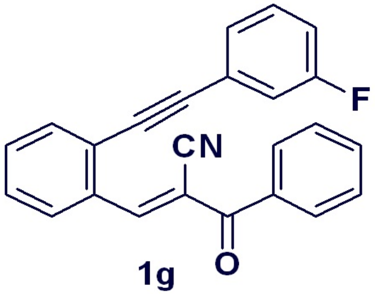

1g

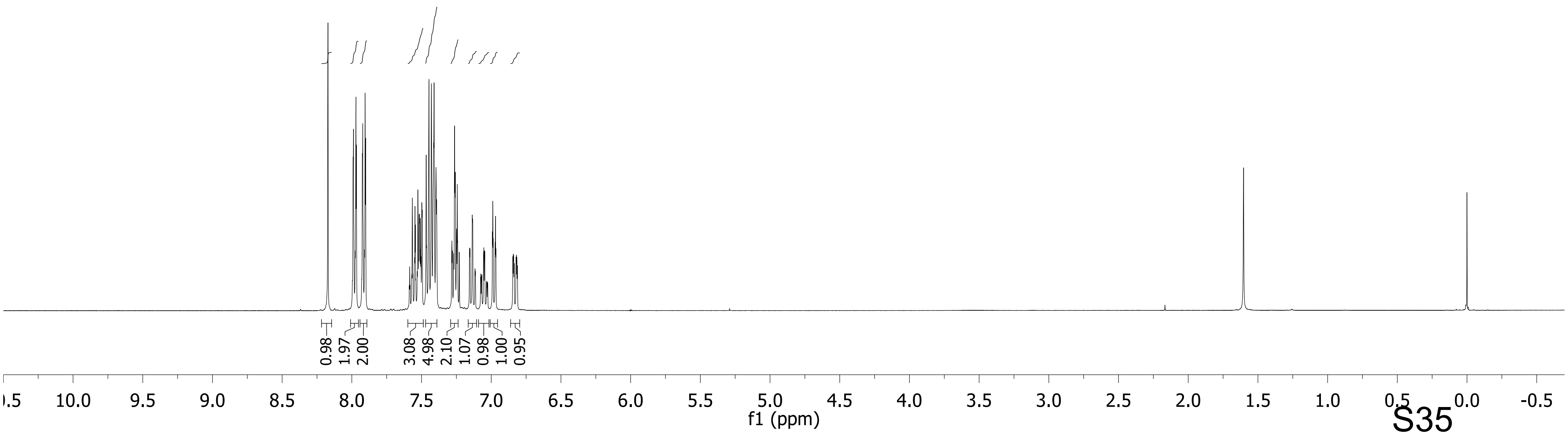

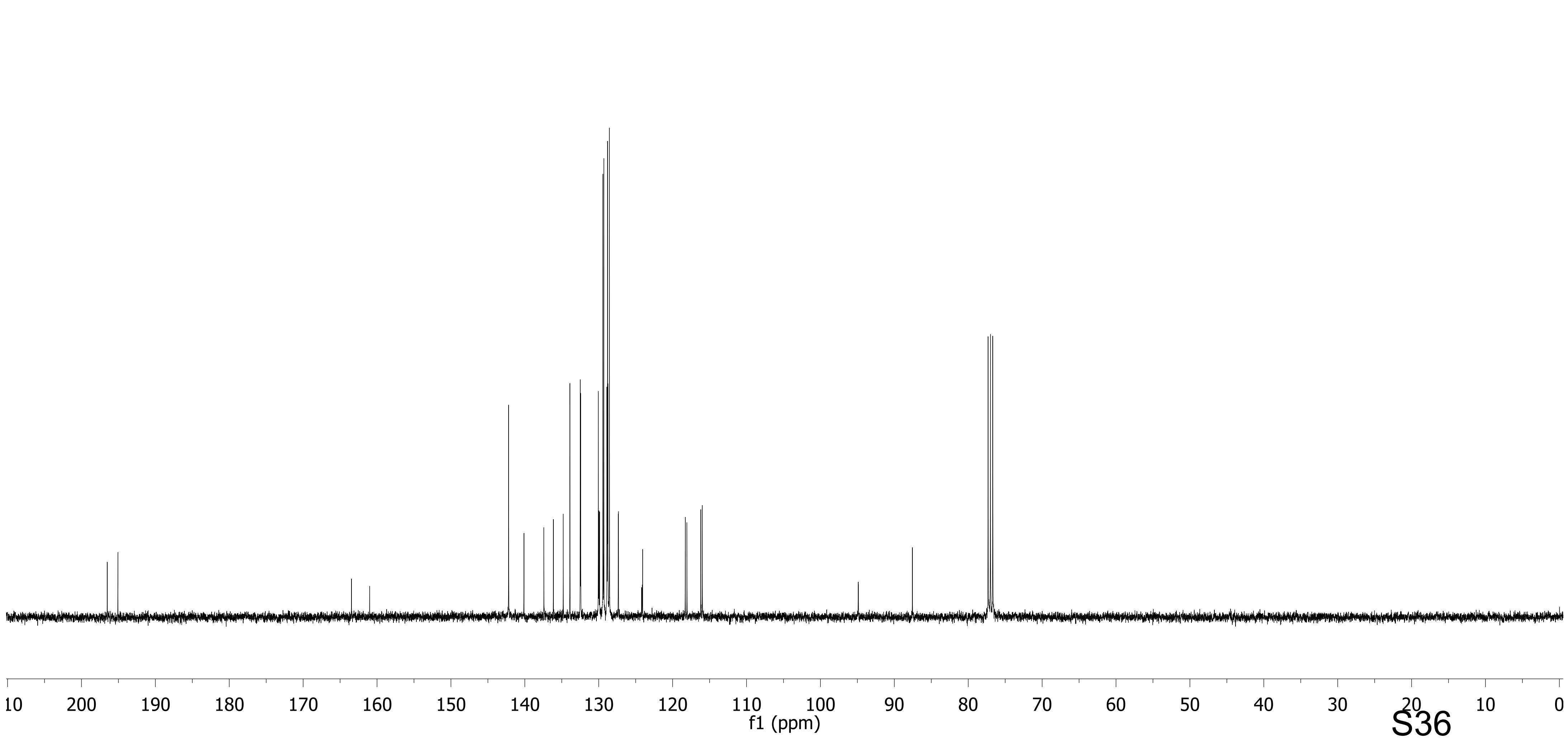
196.5
195.1
163.5
161.0
142.2
133.9
132.5
132.4
130.1
130.0
129.9
129.4
129.3
128.9
128.8
128.8
128.5
127.4
127.3
118.3
118.1
116.2
116.0
94.9
94.9
87.6
77.3
77.0
76.7
Solvent CDCl3
Spectrometer Frequency 100
f1 (ppm)

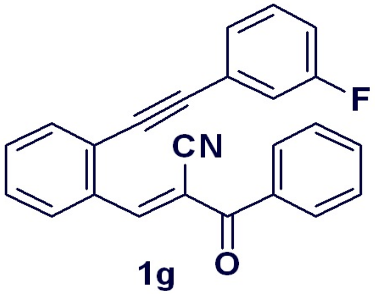
F
CN
O

1g

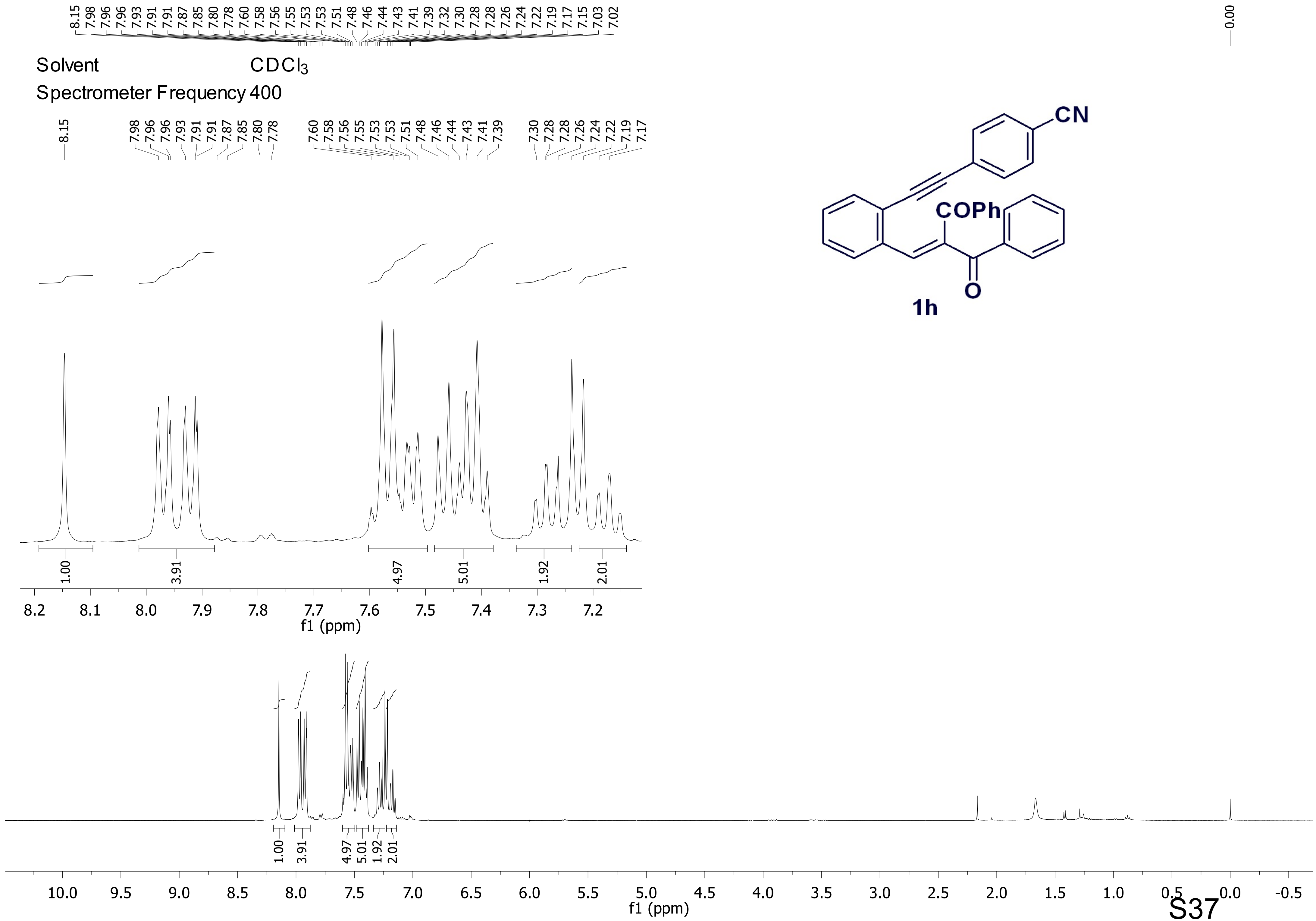
8.15 7.98 7.96 7.96 7.93 7.91 7.91 7.87 7.85 7.80 7.78 7.60 7.58 7.56 7.55 7.53 7.53 7.51 7.48 7.46 7.44 7.43 7.41 7.39 7.32 7.30 7.28 7.28 7.26 7.24 7.22 7.19 7.17 7.15 7.03 7.02
0.00
Solvent CDCl3
Spectrometer Frequency 400
8.15
7.98 7.96 7.96 7.93 7.91 7.91 7.87 7.85 7.80 7.78
7.60 7.58 7.56 7.55 7.53 7.53 7.51 7.48 7.46 7.44 7.43 7.41 7.39
7.30 7.28 7.28 7.26 7.24 7.22 7.19 7.17
1.00
3.91
4.97
5.01
1.92
2.01
8.2 8.1 8.0 7.9 7.8 7.7 7.6 7.5 7.4 7.3 7.2
f1 (ppm)
1.00 3.91 4.97 5.01 1.92 2.01
10.0 9.5 9.0 8.5 8.0 7.5 7.0 6.5 6.0 5.5 5.0 4.5 4.0 3.5 3.0 2.5 2.0 1.5 1.0 0.5 0.0 -0.5
f1 (ppm)
CN
COPh
O

1h

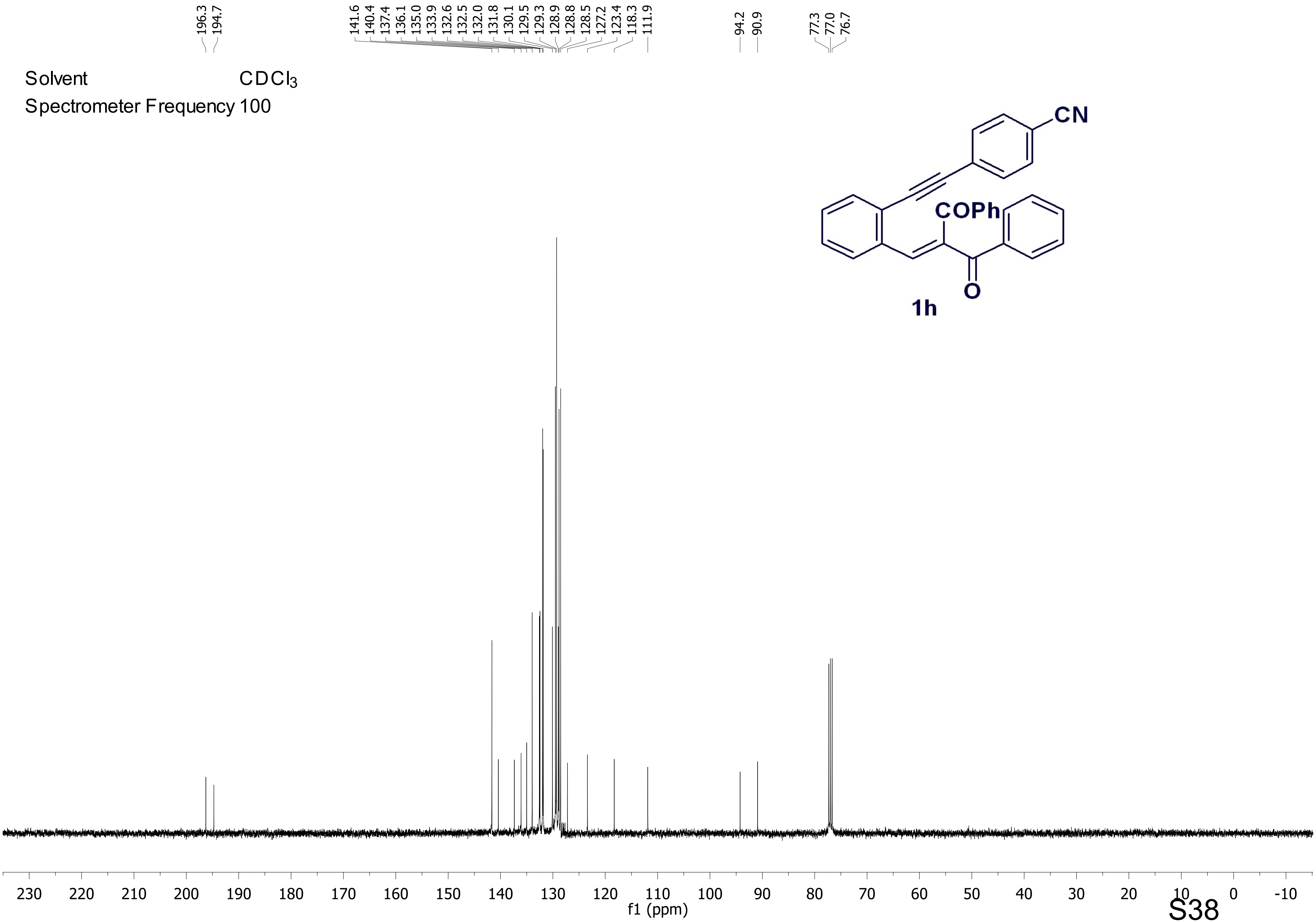
196.3
194.7
141.6
140.4
137.4
136.1
135.0
133.9
132.6
132.5
132.0
131.8
130.1
129.5
129.3
128.9
128.8
128.5
127.2
123.4
118.3
111.9
94.2
90.9
77.3
77.0
76.7
Solvent CDCl3
Spectrometer Frequency 100
CN
COPh
O
1h
230 220 210 200 190 180 170 160 150 140 130 120 110 100 90 80 70 60 50 40 30 20 10 0 -10
f1 (ppm)

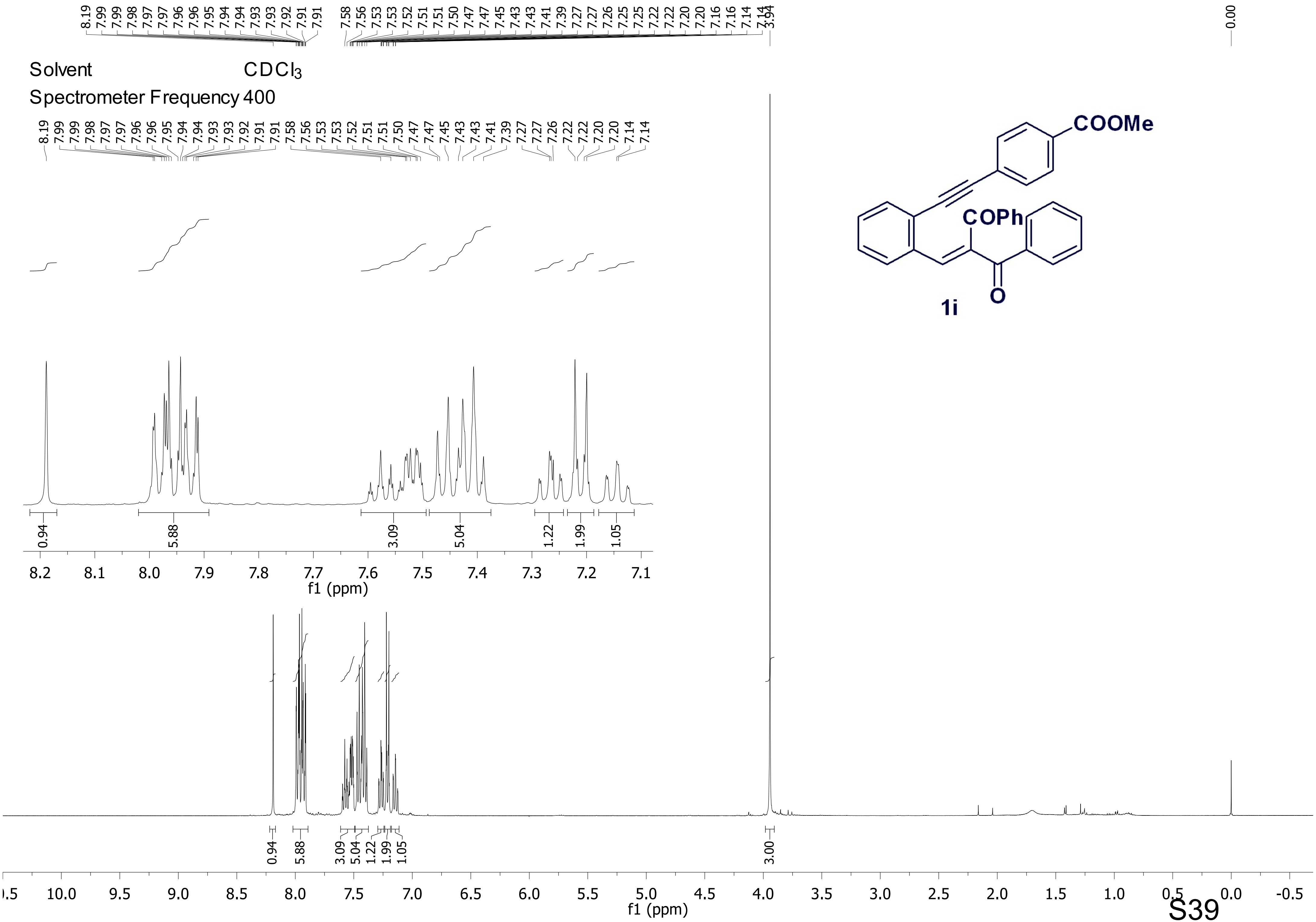
8.19 7.99 7.99 7.98 7.97 7.97 7.96 7.96 7.95 7.94 7.94 7.93 7.93 7.92 7.91 7.91 7.58 7.56 7.53 7.53 7.52 7.51 7.51 7.50 7.47 7.47 7.45 7.43 7.43 7.41 7.39 7.27 7.27 7.26 7.25 7.25 7.22 7.22 7.20 7.20 7.16 7.16 7.14 7.14 3.94
0.00
Solvent CDCl$_3$
Spectrometer Frequency 400
COOMe
COPh
O
1i
0.94
5.88
3.09
5.04
1.22
1.99
1.05
3.00
f1 (ppm)

Solvent CDCl$_3$
Spectrometer Frequency 100

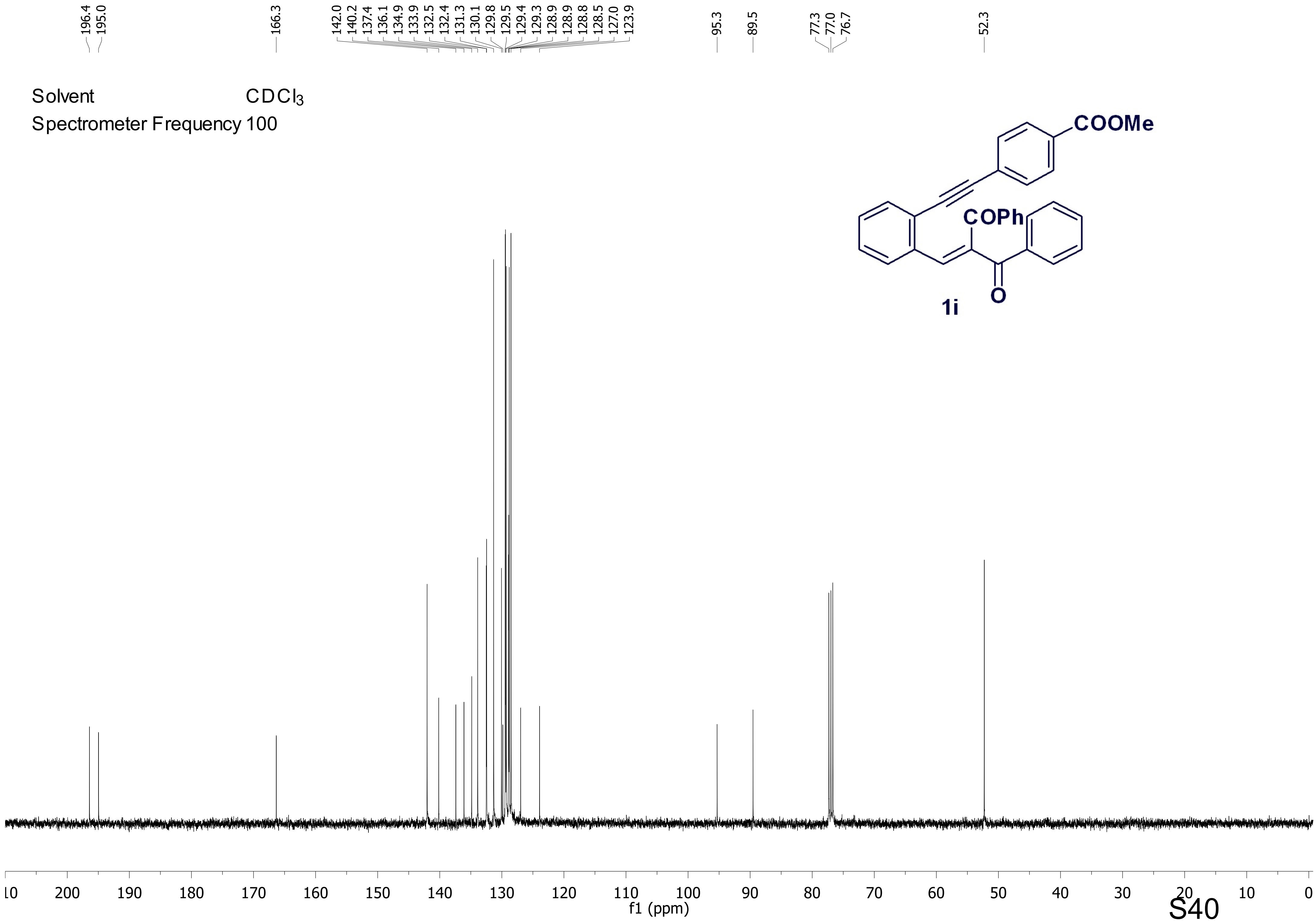

8.50, 8.50, 8.49, 8.49, 8.14, 7.97, 7.95, 7.93, 7.93, 7.72, 7.72, 7.70, 7.70, 7.60, 7.58, 7.56, 7.54, 7.52, 7.47, 7.45, 7.44, 7.43, 7.43, 7.42, 7.41, 7.40, 7.39, 7.38, 7.38, 7.37, 7.34, 7.32, 7.30, 7.29, 7.29, 7.27, 7.27, 7.26, 7.08, 7.07, 7.06, 7.05

-0.00

Solvent CDCl$_3$

Spectrometer Frequency 400

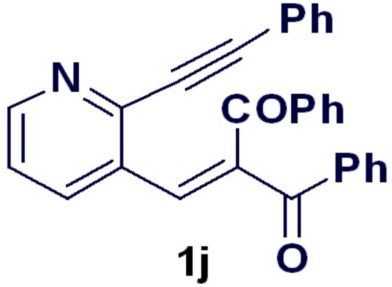

1j

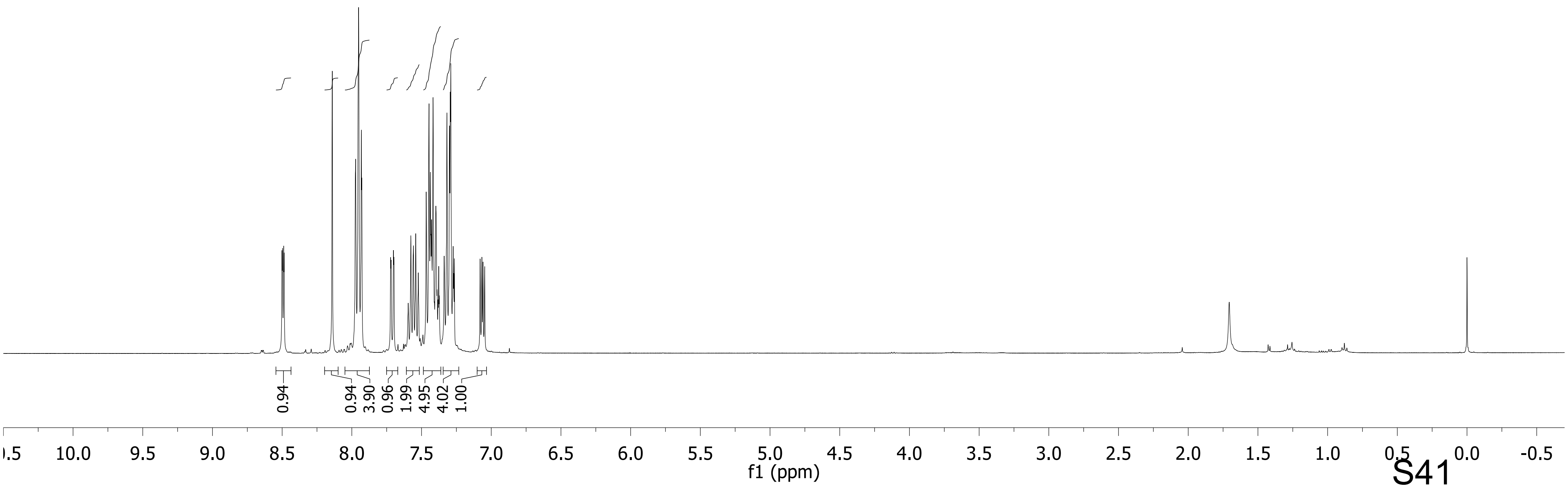

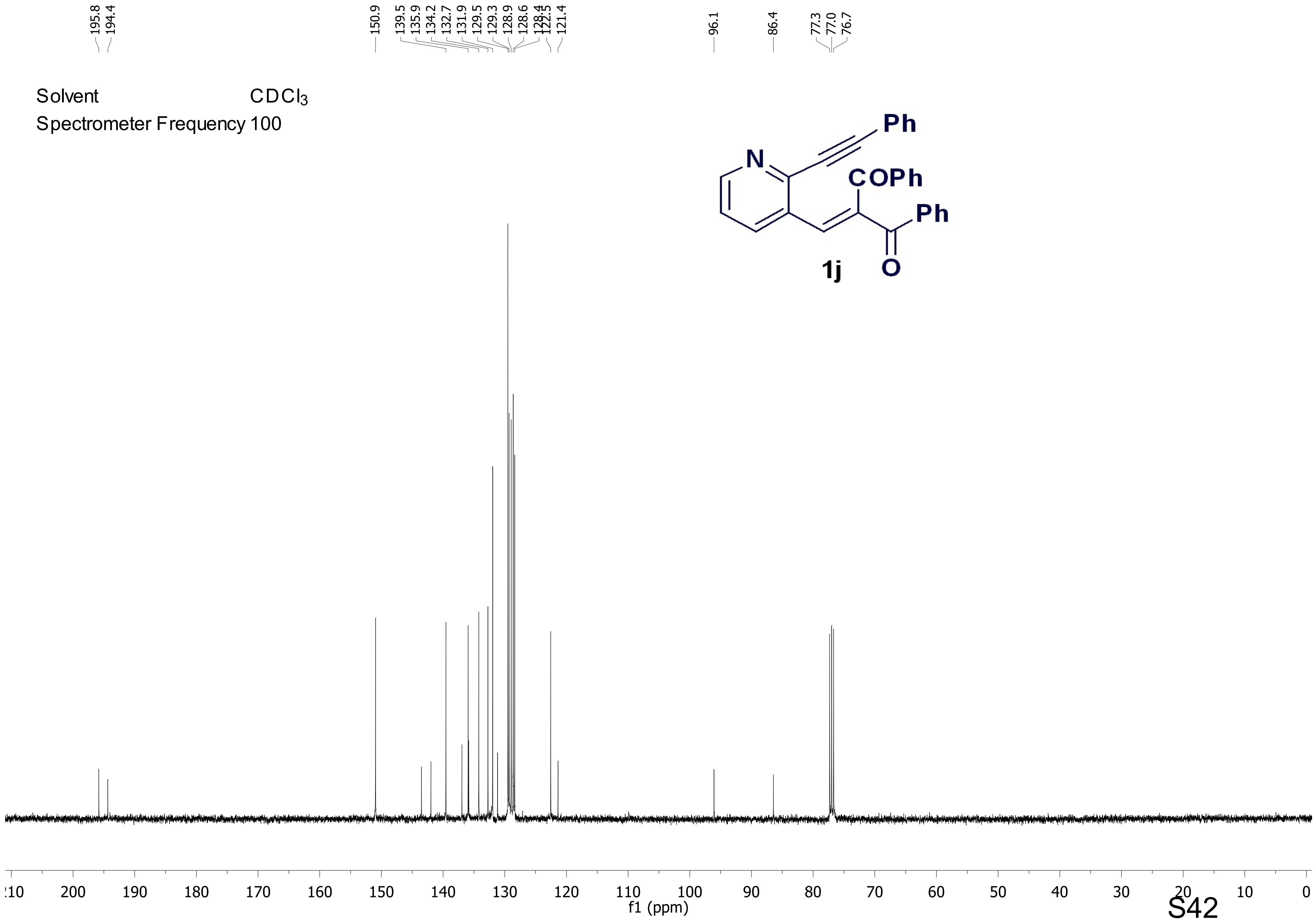
195.8
194.4
150.9
139.5
135.9
134.2
132.7
131.9
129.5
129.3
128.9
128.6
128.4
122.5
121.4
96.1
86.4
77.3
77.0
76.7
Solvent CDCl3
Spectrometer Frequency 100
N
Ph
COPh
Ph
O
1j
f1 (ppm)

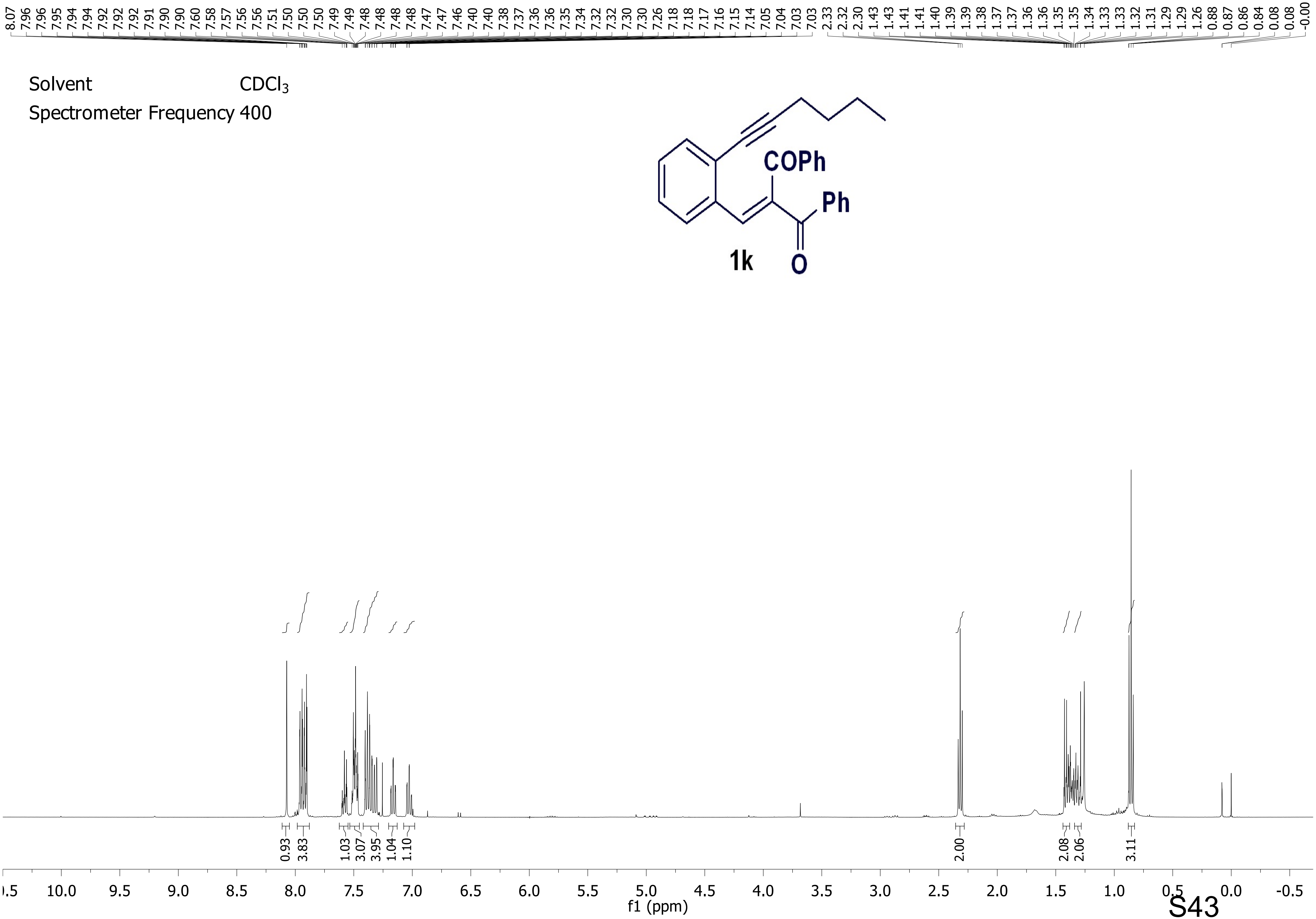
8.07 7.96 7.96 7.95 7.94 7.94 7.92 7.92 7.92 7.91 7.90 7.90 7.60 7.58 7.57 7.56 7.56 7.51 7.50 7.50 7.50 7.49 7.49 7.48 7.48 7.48 7.48 7.47 7.47 7.46 7.40 7.40 7.38 7.37 7.36 7.36 7.35 7.34 7.32 7.32 7.30 7.30 7.26 7.18 7.18 7.17 7.16 7.15 7.14 7.05 7.04 7.03 7.03 2.33 2.32 2.30 1.43 1.43 1.41 1.41 1.40 1.39 1.39 1.38 1.37 1.37 1.36 1.36 1.35 1.35 1.35 1.34 1.33 1.33 1.32 1.31 1.29 1.29 1.26 0.88 0.87 0.86 0.84 0.08 0.08 -0.00
Solvent CDCl$_3$
Spectrometer Frequency 400
COPh
Ph
O
1k
0.93 3.83 1.03 3.07 3.95 1.04 1.10 2.00 2.08 2.06 3.11
10.0 9.5 9.0 8.5 8.0 7.5 7.0 6.5 6.0 5.5 5.0 4.5 4.0 3.5 3.0 2.5 2.0 1.5 1.0 0.5 0.0 -0.5
f1 (ppm)

Solvent CDCl$_3$
Spectrometer Frequency 100

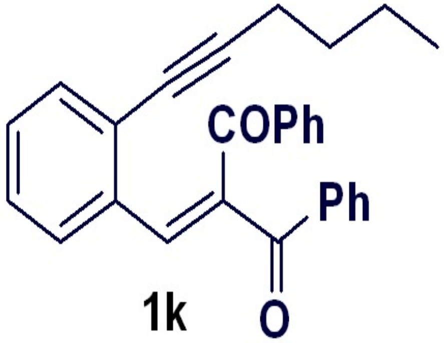

1k

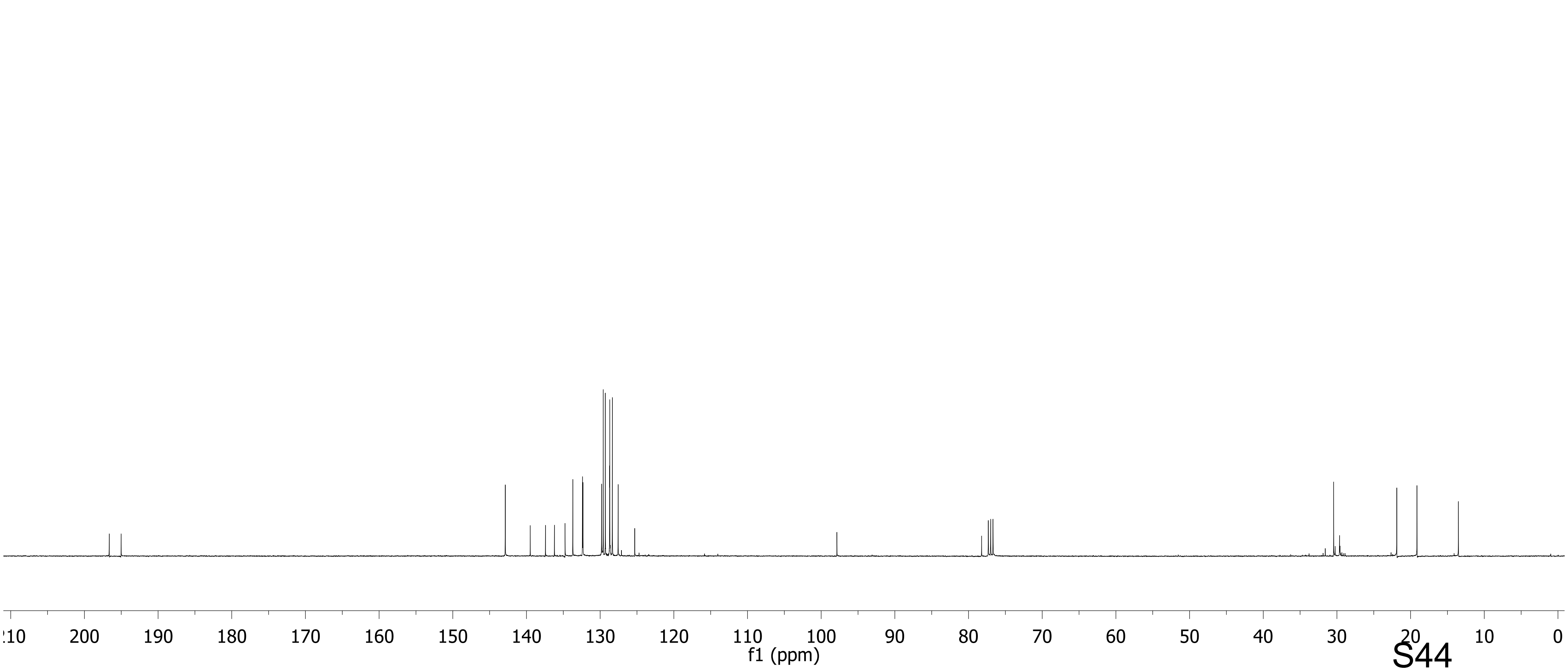

8.09 8.00 8.00 7.99 7.98 7.98 7.84 7.83 7.82 7.82 7.60 7.58 7.57 7.56 7.56 7.56 7.53 7.51 7.51 7.50 7.48 7.46 7.45 7.43 7.42 7.41 7.29 7.29 7.28 7.28 7.27 7.26 7.24 7.22 7.20 7.20 6.96 6.94 6.94 6.94

2.43 2.42 2.40 2.11 2.09 2.08 1.59 1.58 1.57 1.56 1.55 1.54 1.53 1.52 1.52 1.51

-0.00

Solvent $CDCl_3$

Spectrometer Frequency 400

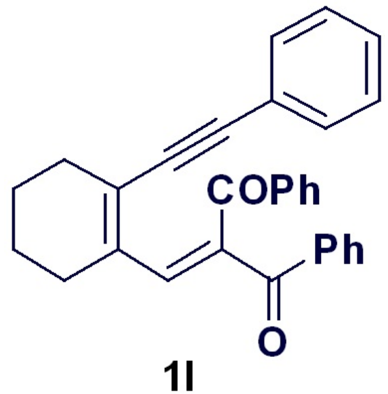

1l

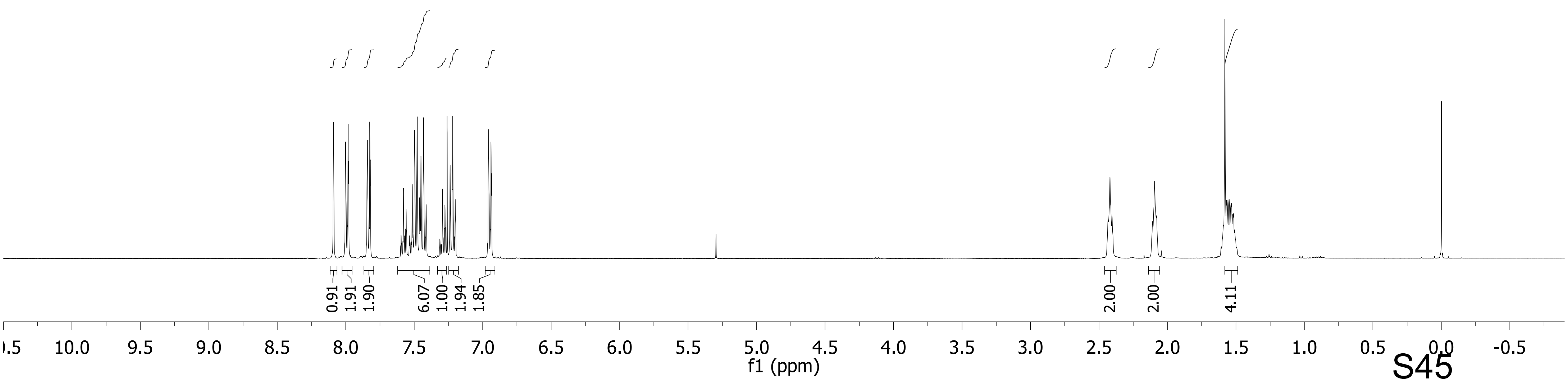

Solvent CDCl$_3$
Spectrometer Frequency 100

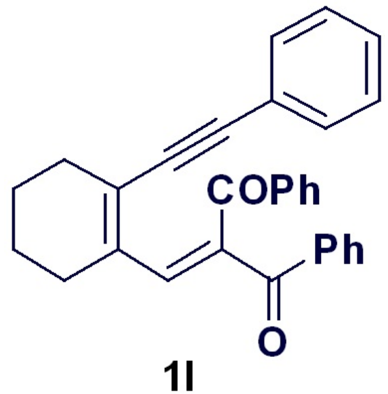

1l

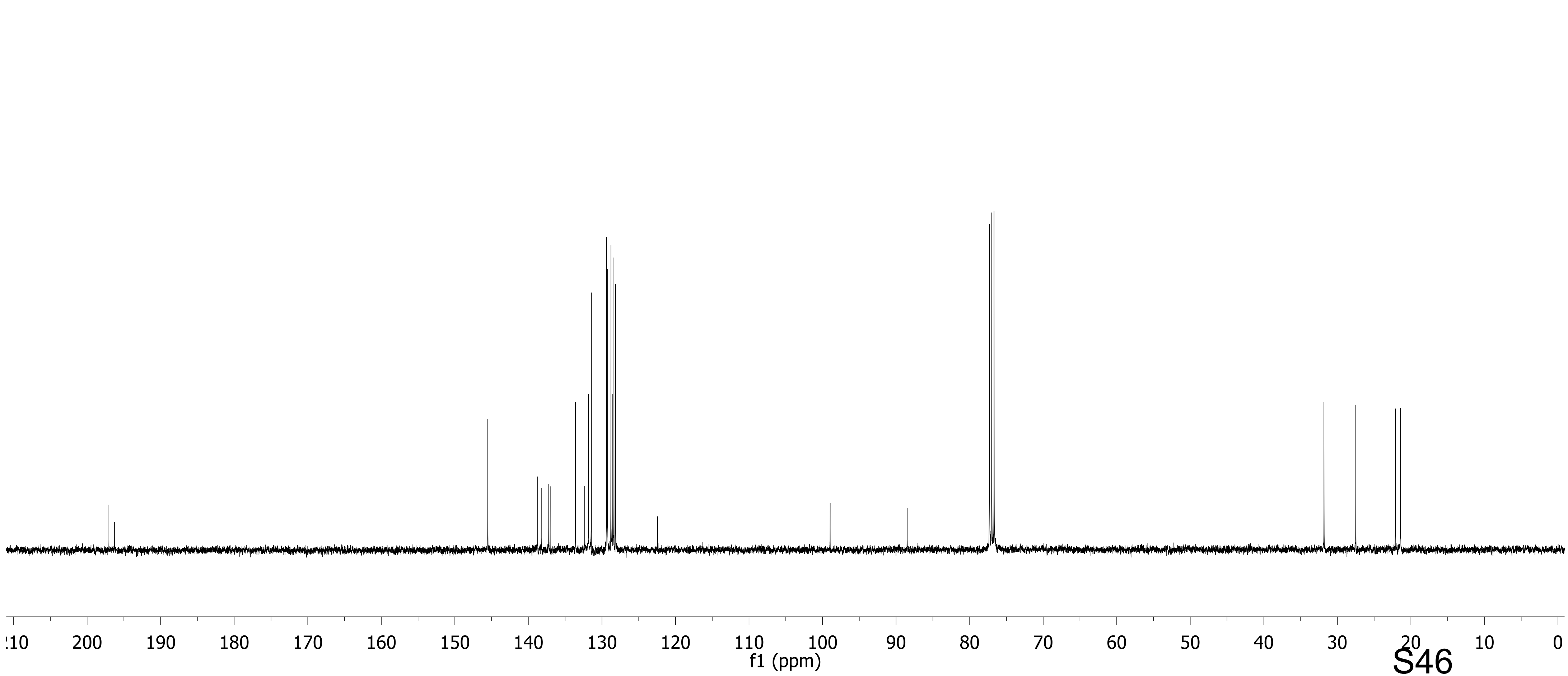

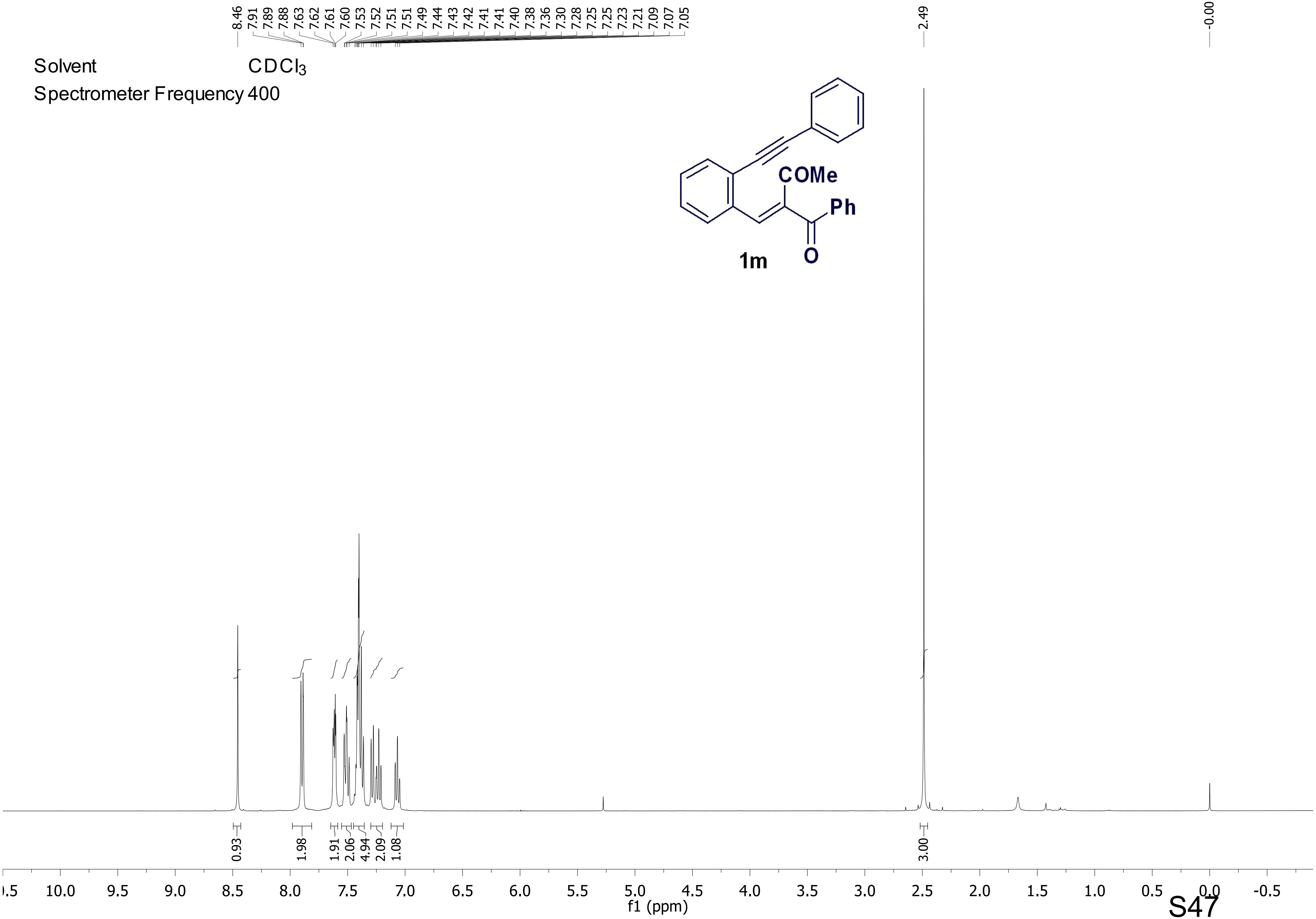

8.46 7.91 7.89 7.88 7.63 7.62 7.61 7.60 7.53 7.52 7.51 7.51 7.49 7.44 7.43 7.42 7.41 7.41 7.40 7.38 7.36 7.30 7.28 7.25 7.25 7.23 7.21 7.09 7.07 7.05
2.49
-0.00
Solvent CDCl3
Spectrometer Frequency 400
COMe
Ph
O
1m
0.93 1.98 1.91 2.06 4.94 2.09 1.08
3.00
10.0 9.5 9.0 8.5 8.0 7.5 7.0 6.5 6.0 5.5 5.0 4.5 4.0 3.5 3.0 2.5 2.0 1.5 1.0 0.5 0.0 -0.5
f1 (ppm)

Solvent CDCl$_3$

Spectrometer Frequency 100

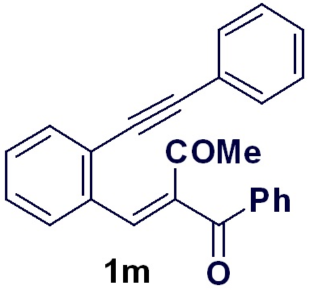

1m

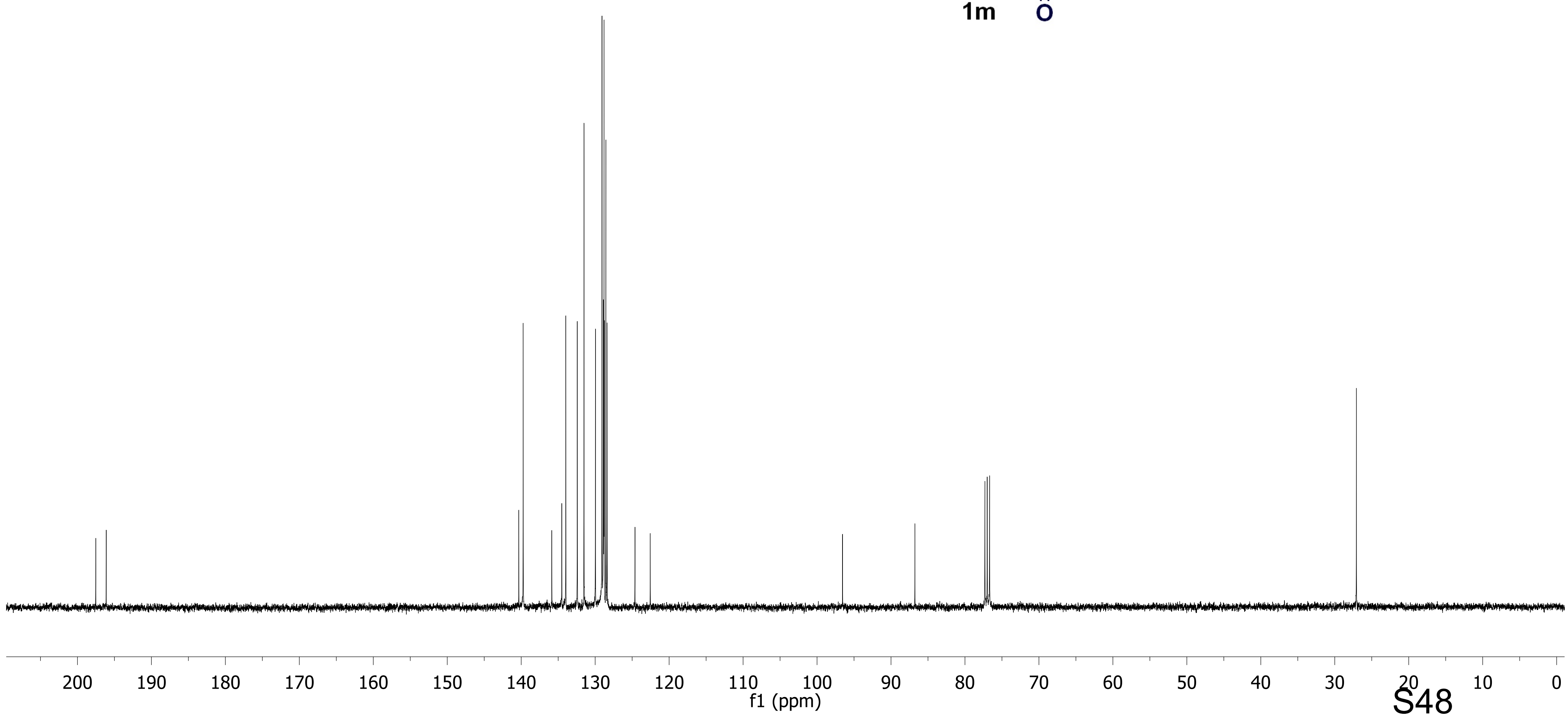

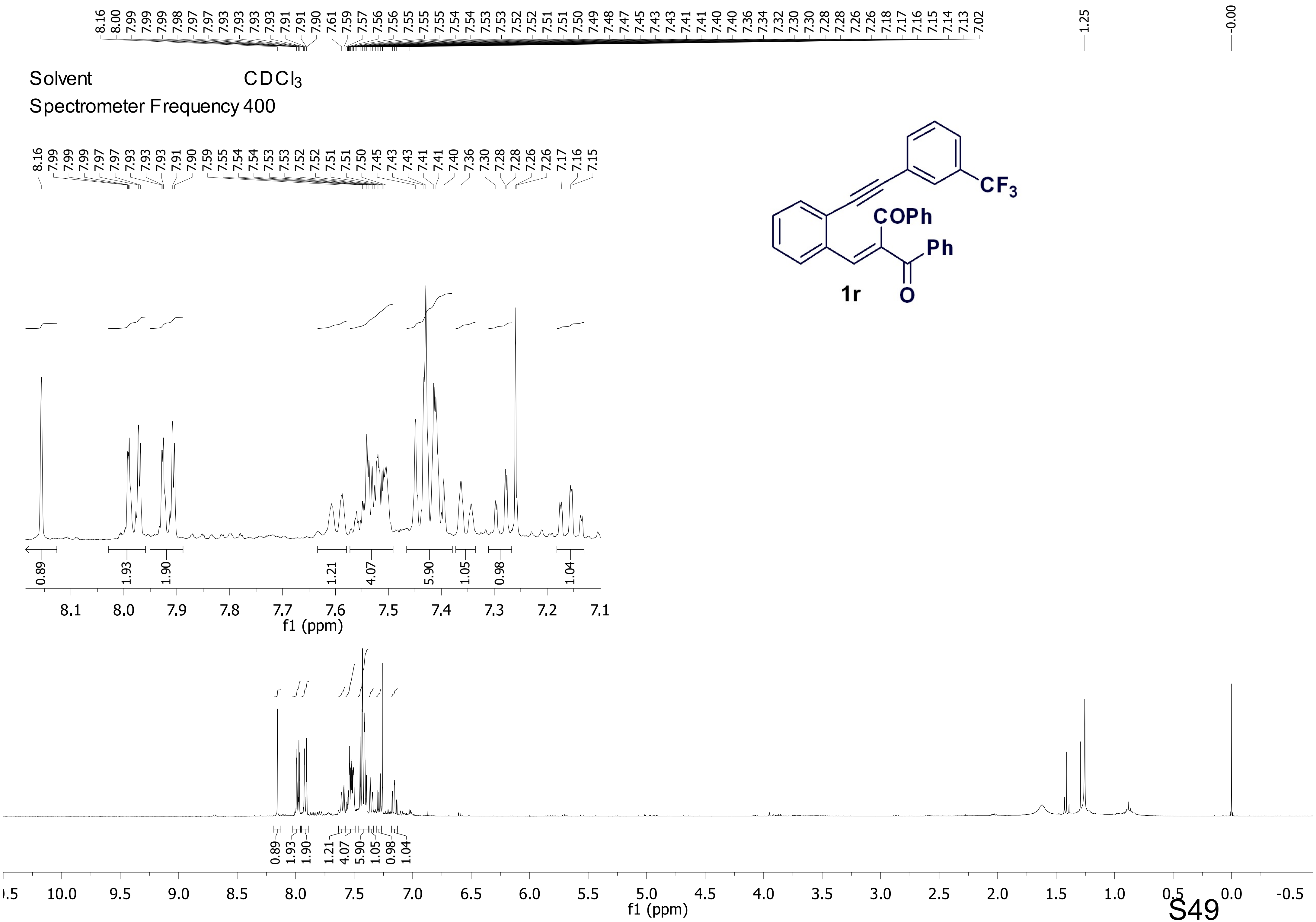
Solvent CDCl3
Spectrometer Frequency 400
CF3
COPh
Ph
O
1r
f1 (ppm)

Solvent CDCl$_3$

Spectrometer Frequency 100

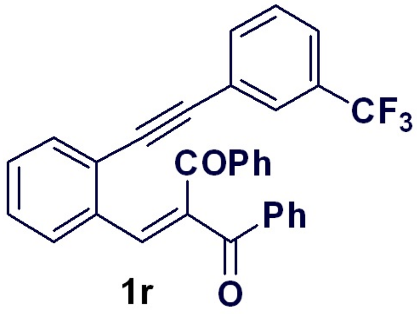

1r

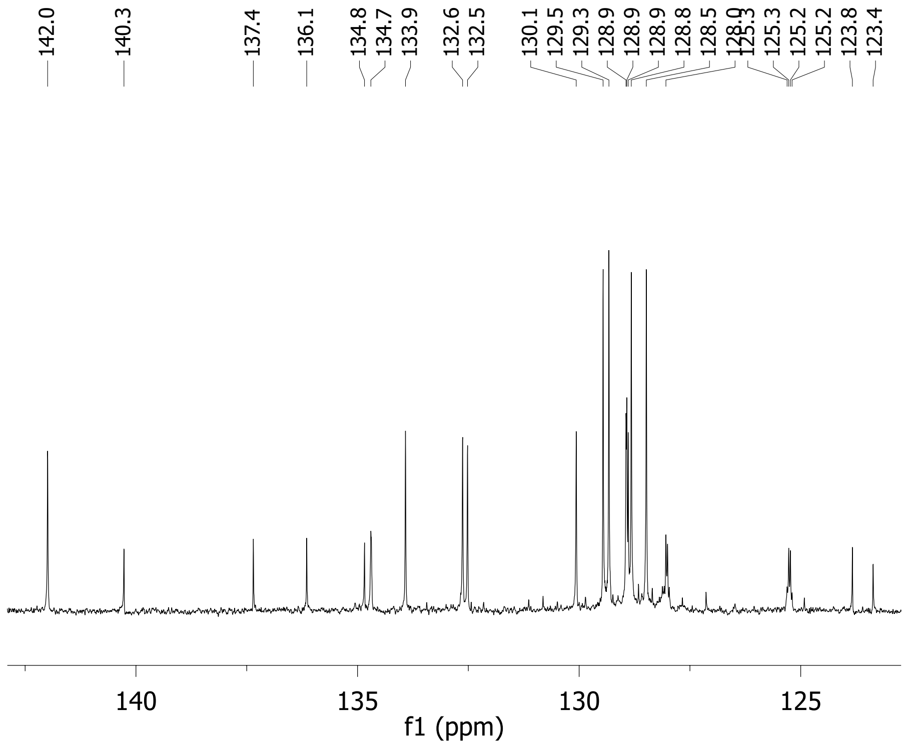

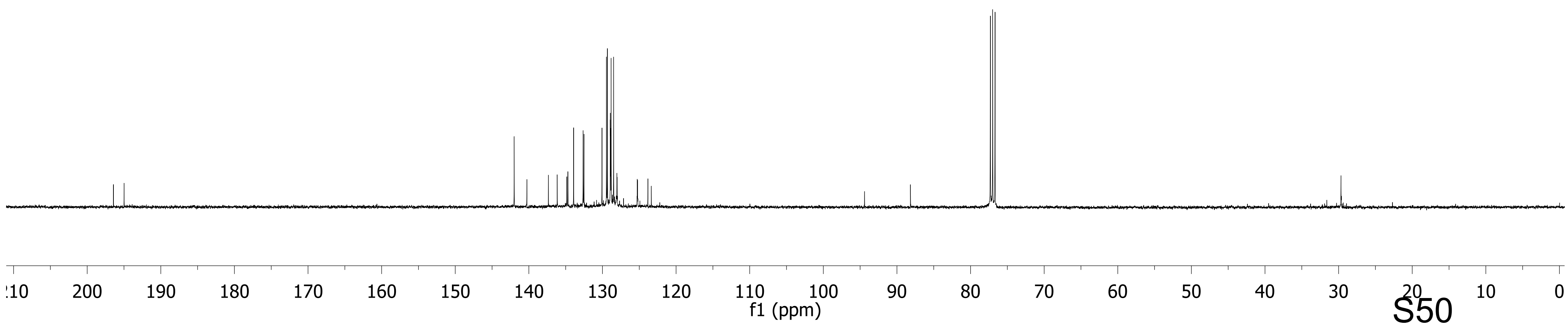

Solvent $CDCl_3$
Spectrometer Frequency 400

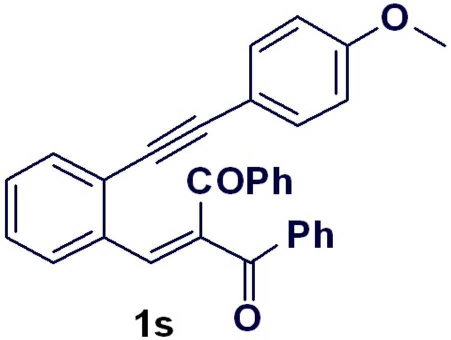

1s

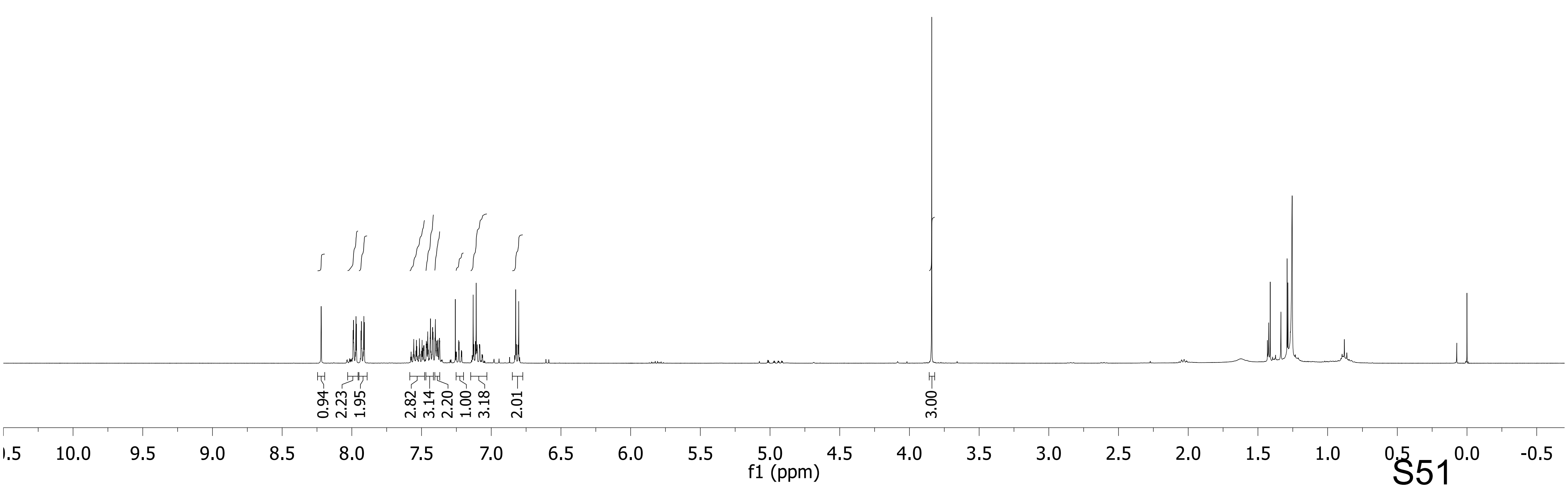

196.7 195.1 160.0 142.7 139.7 137.5 136.2 134.5 133.8 133.0 132.3 132.1 130.0 129.5 129.3 128.9 128.8 128.5 128.0 114.5 114.0 114.0 96.6 85.7 77.3 77.0 76.7 55.3 29.7

Solvent CDCl$_3$

Spectrometer Frequency 100

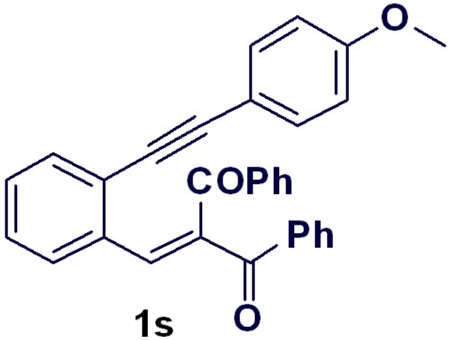

1s

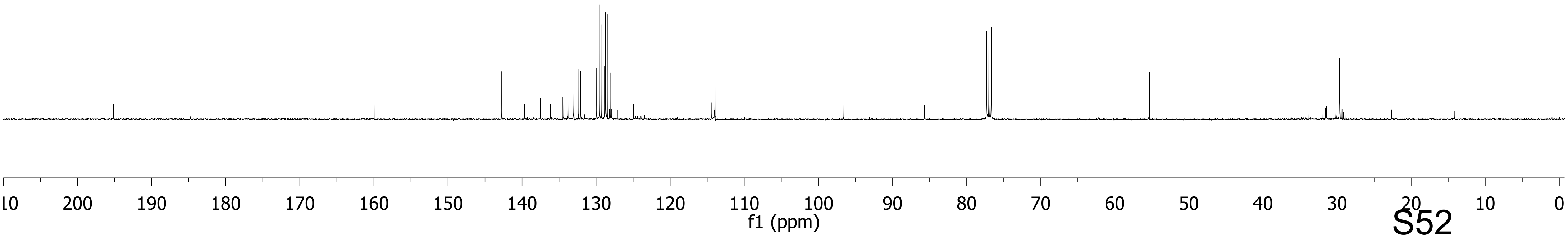

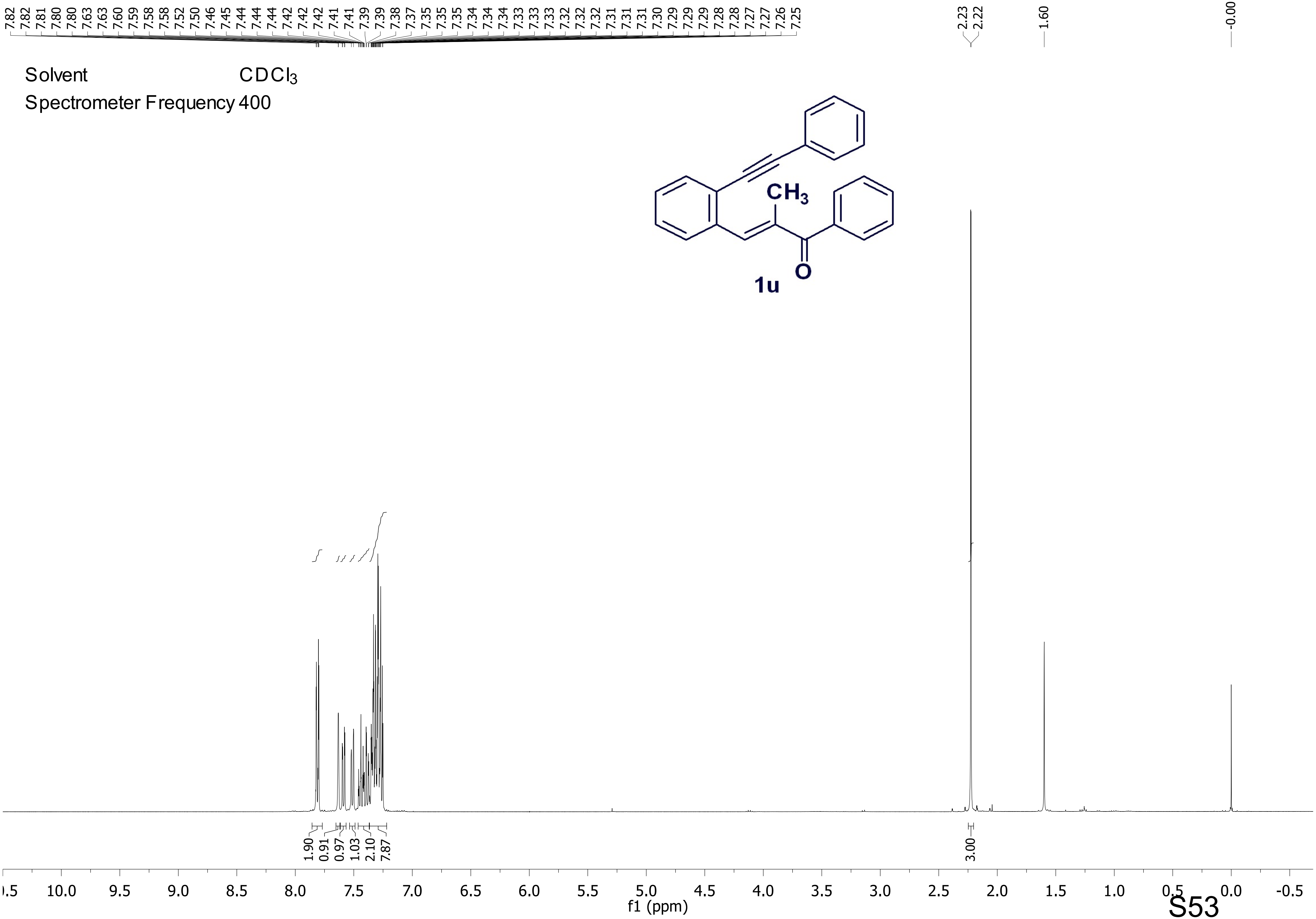

7.82 7.82 7.81 7.80 7.80 7.63 7.63 7.60 7.59 7.58 7.58 7.52 7.50 7.46 7.45 7.44 7.44 7.44 7.42 7.42 7.42 7.41 7.41 7.39 7.39 7.38 7.37 7.35 7.35 7.34 7.34 7.34 7.33 7.33 7.33 7.32 7.32 7.32 7.31 7.31 7.31 7.30 7.29 7.29 7.29 7.28 7.28 7.27 7.27 7.26 7.25
2.23 2.22
1.60
-0.00
Solvent CDCl3
Spectrometer Frequency 400
CH3
O
1u
1.90 0.91 0.97 1.03 2.10 7.87
3.00
10.0 9.5 9.0 8.5 8.0 7.5 7.0 6.5 6.0 5.5 5.0 4.5 4.0 3.5 3.0 2.5 2.0 1.5 1.0 0.5 0.0 -0.5
f1 (ppm)

Solvent CDCl$_3$
Spectrometer Frequency 100

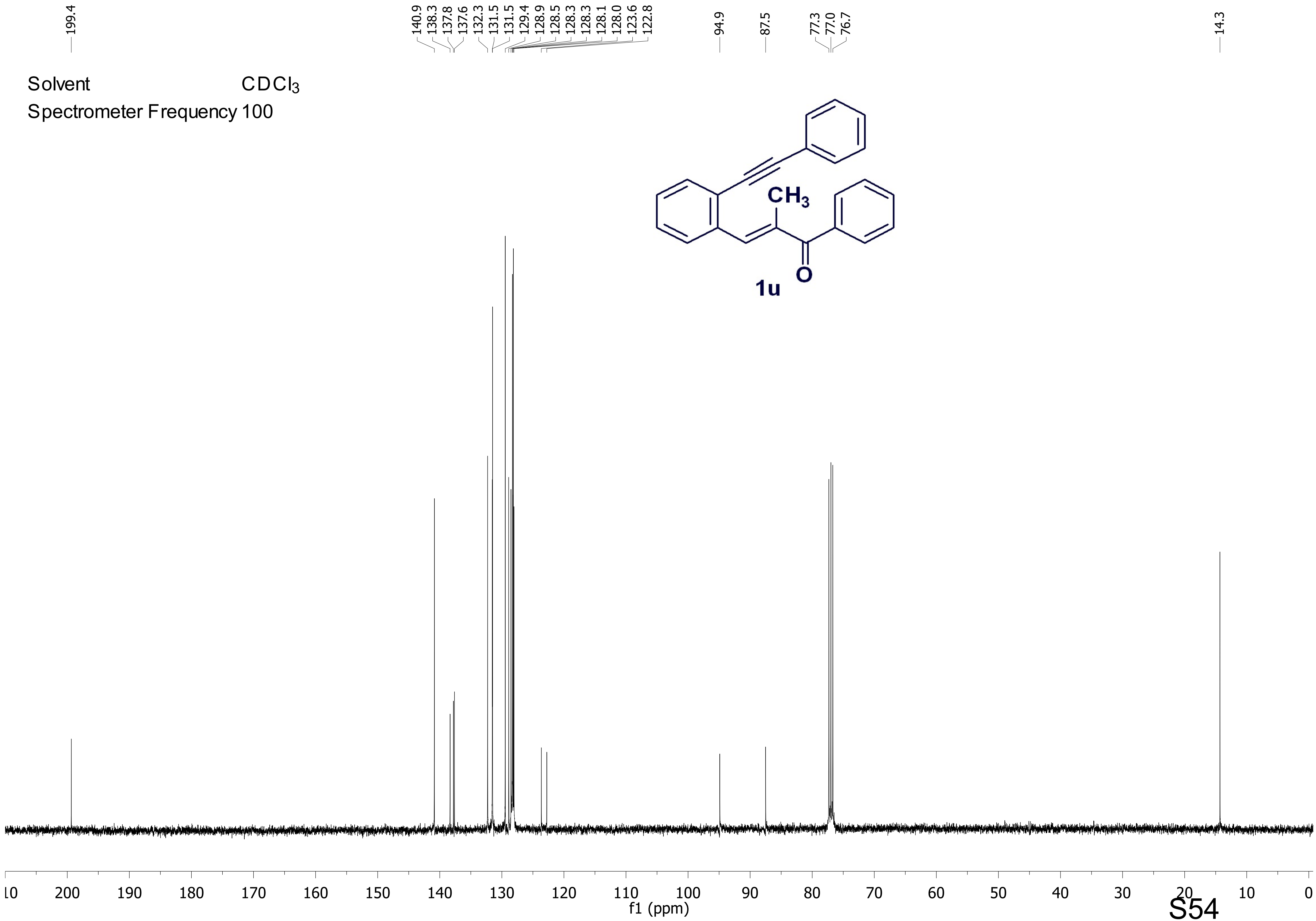

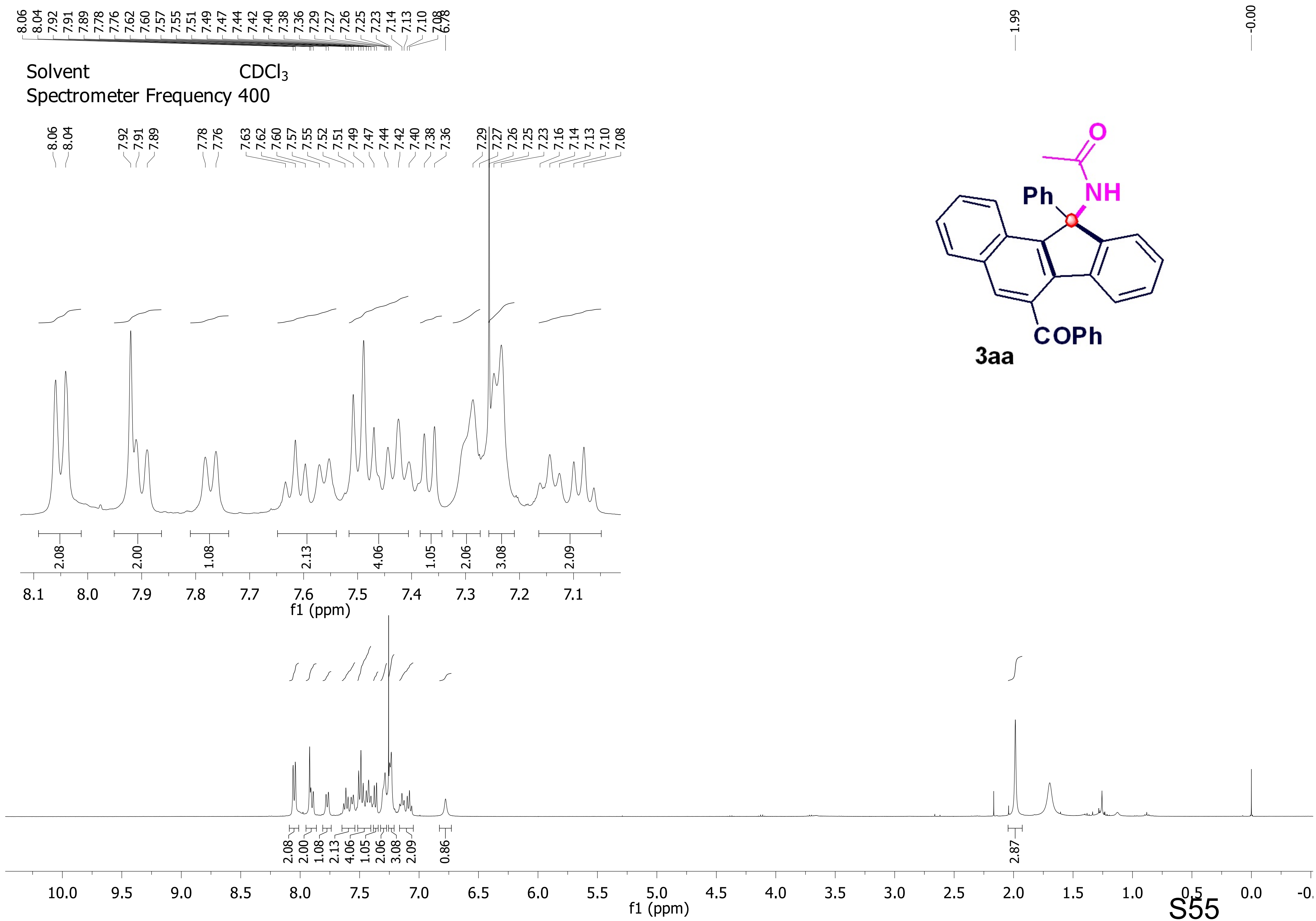
Solvent CDCl3
Spectrometer Frequency 400
Ph
NH
O
COPh
3aa
f1 (ppm)

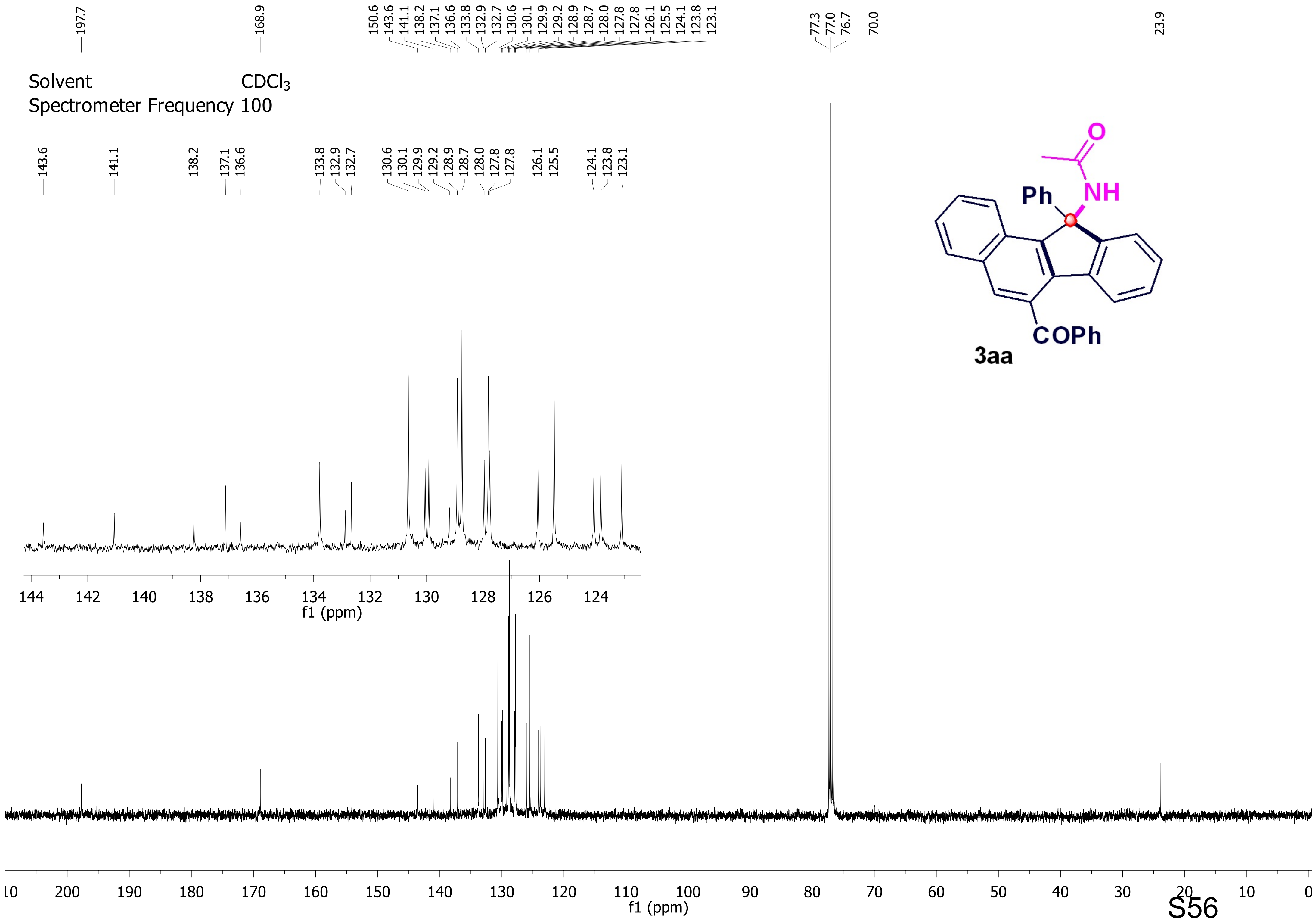

197.7
168.9
150.6 143.6 141.1 138.2 137.1 136.6 133.8 132.9 132.7 130.6 130.1 129.9 129.2 128.9 128.7 128.0 127.8 127.8 126.1 125.5 124.1 123.8 123.1
77.3 77.0 76.7 70.0
23.9
Solvent CDCl3
Spectrometer Frequency 100
143.6 141.1 138.2 137.1 136.6 133.8 132.9 132.7 130.6 130.1 129.9 129.2 128.9 128.7 128.0 127.8 127.8 126.1 125.5 124.1 123.8 123.1
f1 (ppm)
O
NH
Ph
COPh
3aa
f1 (ppm)

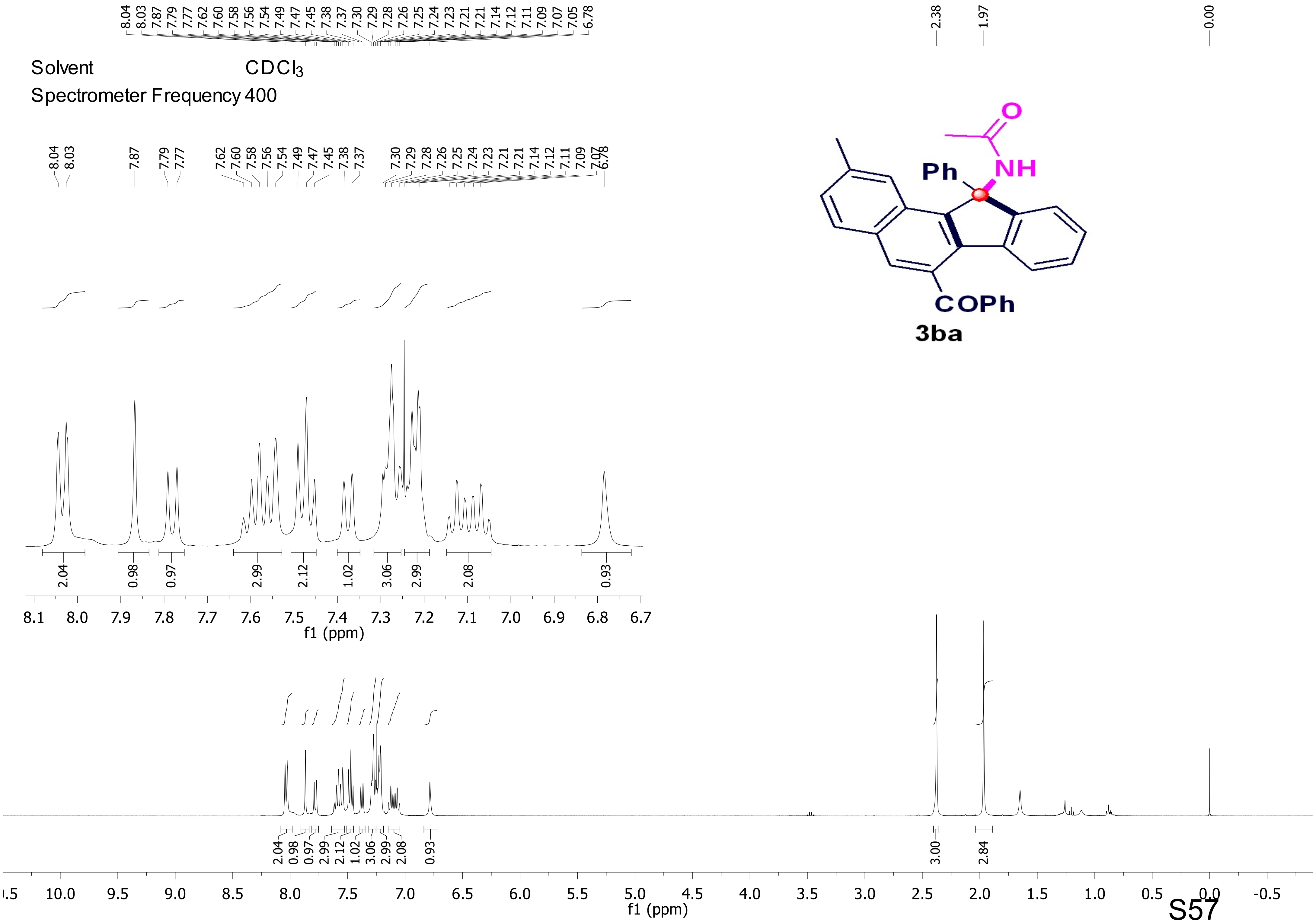
Solvent CDCl$_3$
Spectrometer Frequency 400
Ph
NH
O
COPh
3ba
f1 (ppm)

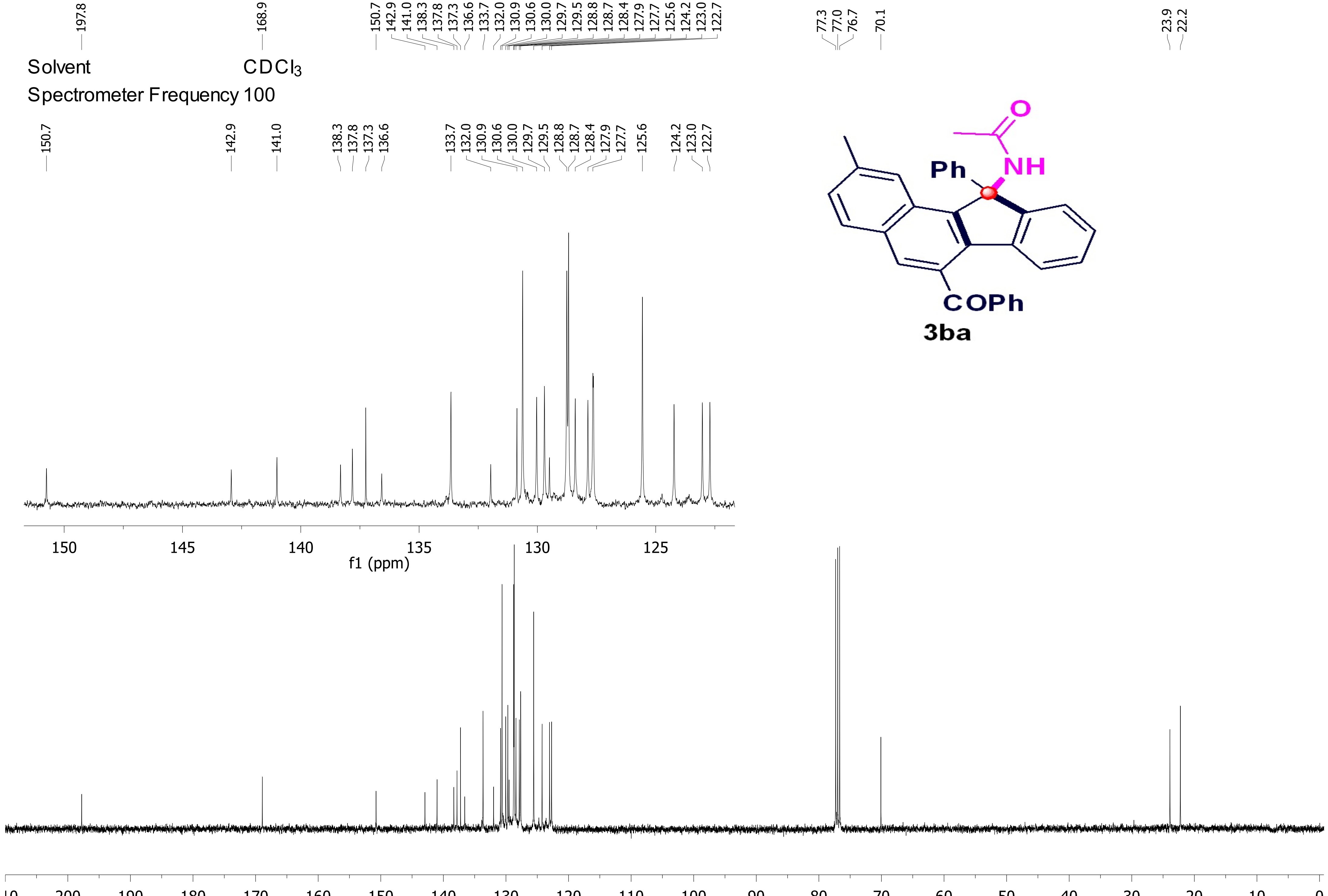

197.8
168.9
150.7 142.9 141.0 138.3 137.8 137.3 136.6 133.7 132.0 130.9 130.6 130.0 129.7 129.5 128.8 128.7 128.4 127.9 127.7 125.6 124.2 123.0 122.7
77.3 77.0 76.7 70.1
23.9 22.2
Solvent CDCl3
Spectrometer Frequency 100
O
NH
Ph
COPh
3ba
f1 (ppm)
f1 (ppm)

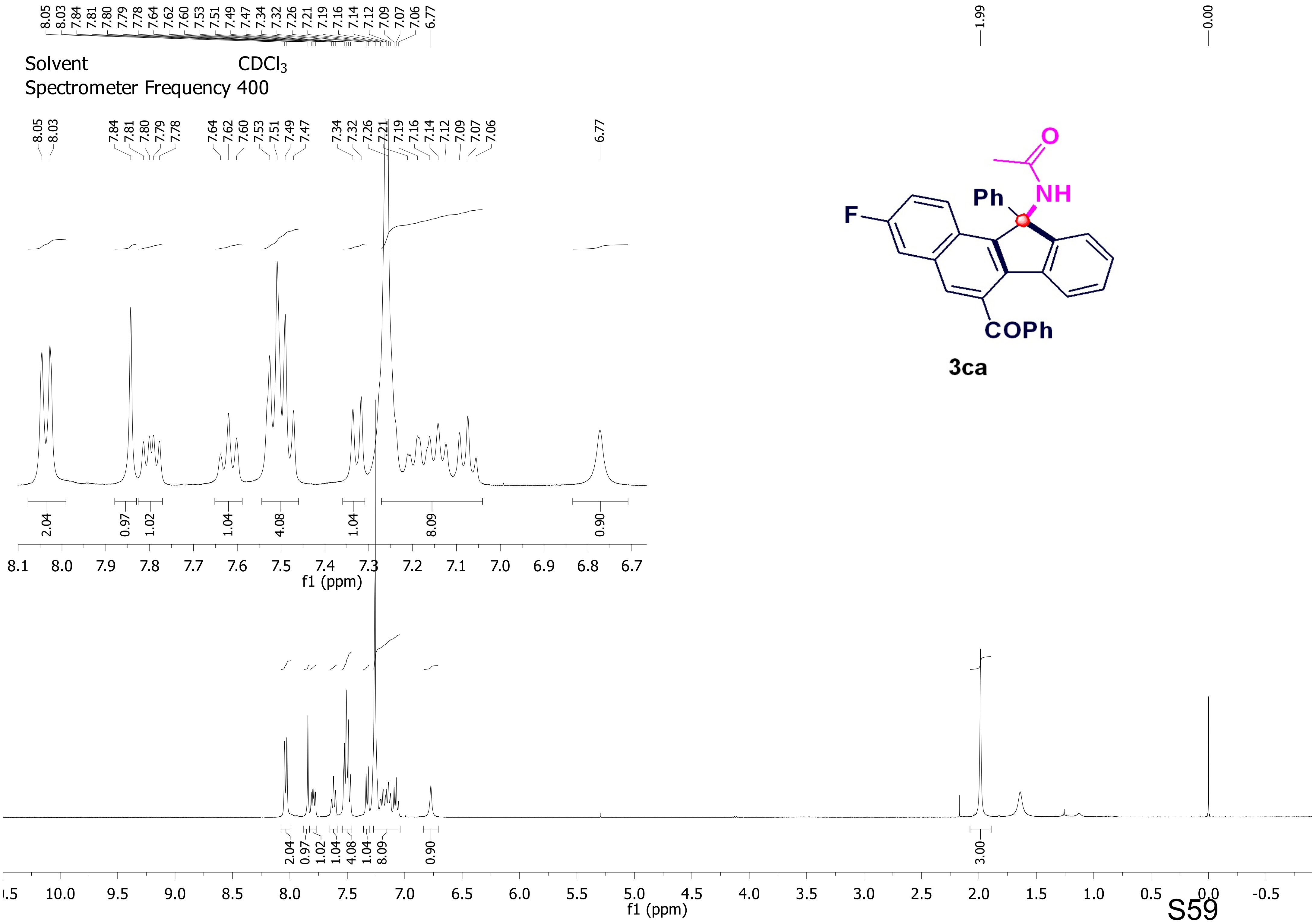
8.05 8.03 7.84 7.81 7.80 7.79 7.78 7.64 7.62 7.60 7.53 7.51 7.49 7.47 7.34 7.32 7.26 7.21 7.19 7.16 7.14 7.12 7.09 7.07 7.06 6.77
1.99
0.00
Solvent CDCl3
Spectrometer Frequency 400
2.04
0.97
1.02
1.04
4.08
1.04
8.09
0.90
3.00
f1 (ppm)
F
Ph
NH
O
COPh
3ca

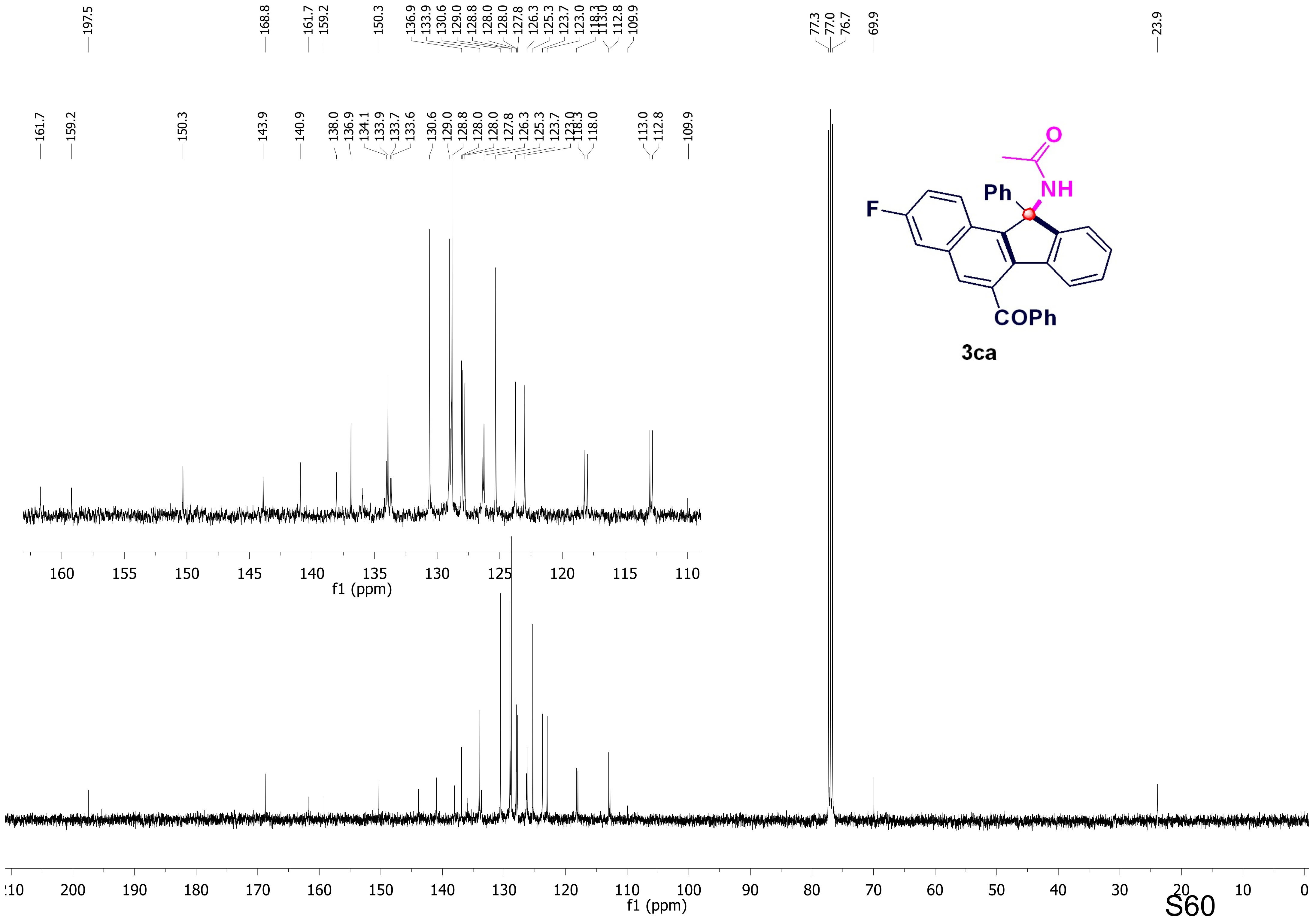
197.5
168.8
161.7
159.2
150.3
136.9
133.9
130.6
129.0
128.8
128.0
128.0
127.8
126.3
125.3
123.7
123.0
118.3
113.0
112.8
109.9
77.3
77.0
76.7
69.9
23.9
161.7
159.2
150.3
143.9
140.9
138.0
136.9
134.1
133.9
133.7
133.6
130.6
129.0
128.8
128.0
128.0
127.8
126.3
125.3
123.7
123.0
118.3
118.0
113.0
112.8
109.9
F
Ph
NH
O
COPh
f1 (ppm)
f1 (ppm)

3ca

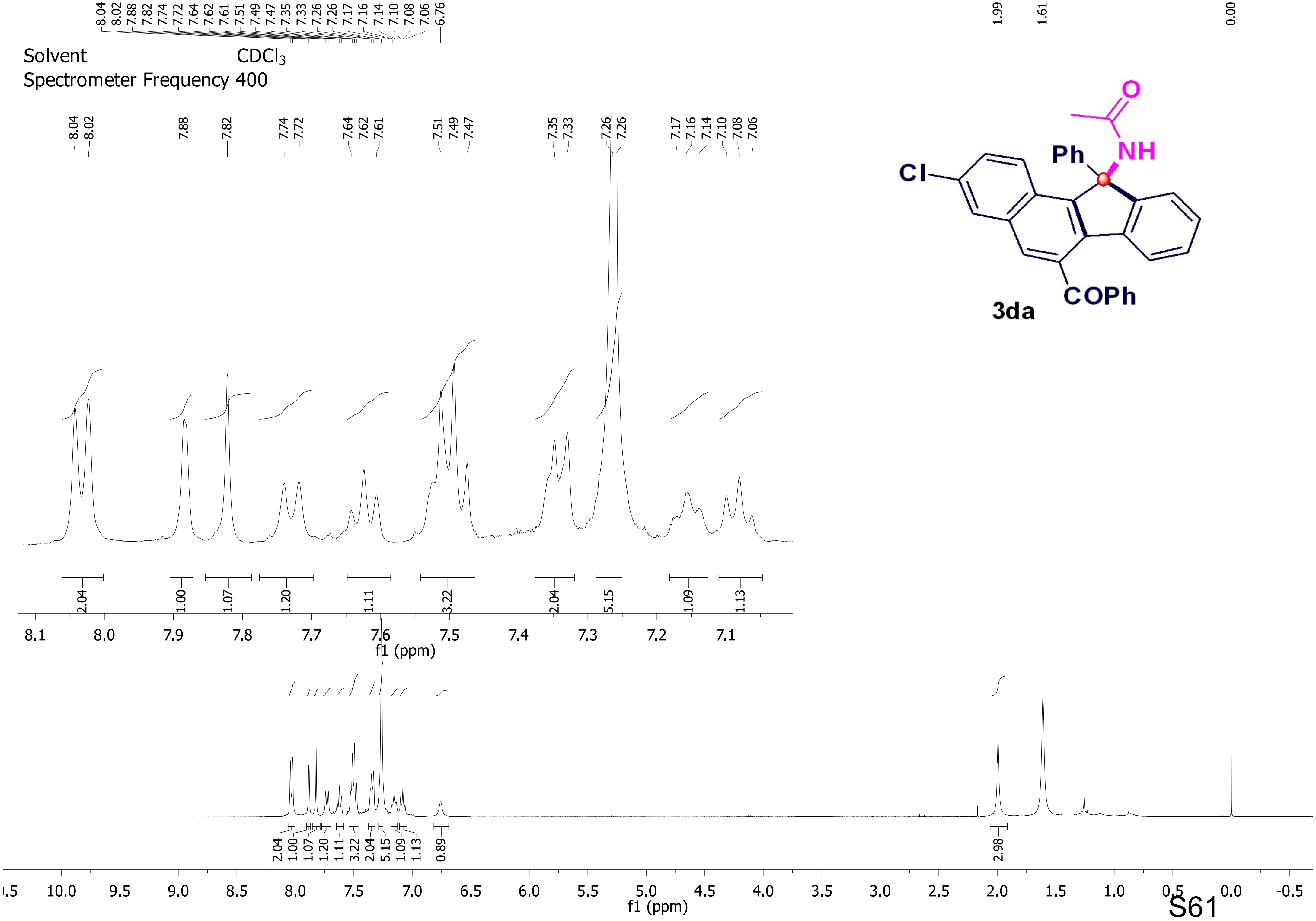

Solvent CDCl$_3$
Spectrometer Frequency 400
8.04 8.02 7.88 7.82 7.74 7.72 7.64 7.62 7.61 7.51 7.49 7.47 7.35 7.33 7.26 7.26 7.17 7.16 7.14 7.10 7.08 7.06 6.76
1.99
1.61
0.00
Cl
Ph
NH
O
COPh
3da
2.04 1.00 1.07 1.20 1.11 3.22 2.04 5.15 1.09 1.13 0.89
2.98
f1 (ppm)

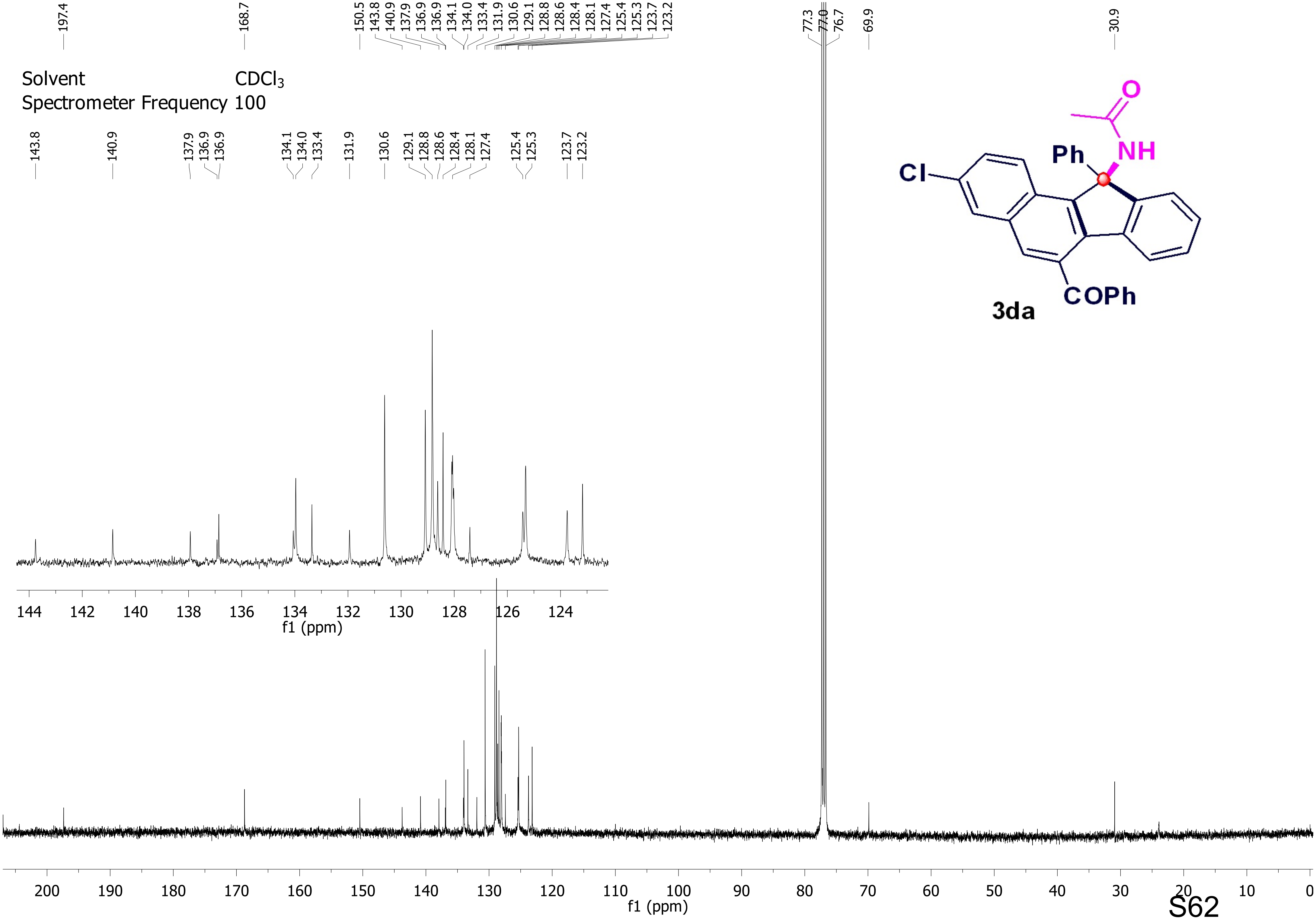
197.4
168.7
150.5
143.8
140.9
137.9
136.9
136.9
134.1
134.0
133.4
131.9
130.6
129.1
128.8
128.6
128.4
128.1
127.4
125.4
125.3
123.7
123.2
77.3
77.0
76.7
69.9
30.9
Solvent CDCl3
Spectrometer Frequency 100
143.8
140.9
137.9
136.9
136.9
134.1
134.0
133.4
131.9
130.6
129.1
128.8
128.6
128.4
128.1
127.4
125.4
125.3
123.7
123.2
O
NH
Ph
Cl
COPh
3da
144 142 140 138 136 134 132 130 128 126 124
f1 (ppm)
200 190 180 170 160 150 140 130 120 110 100 90 80 70 60 50 40 30 20 10 0
f1 (ppm)

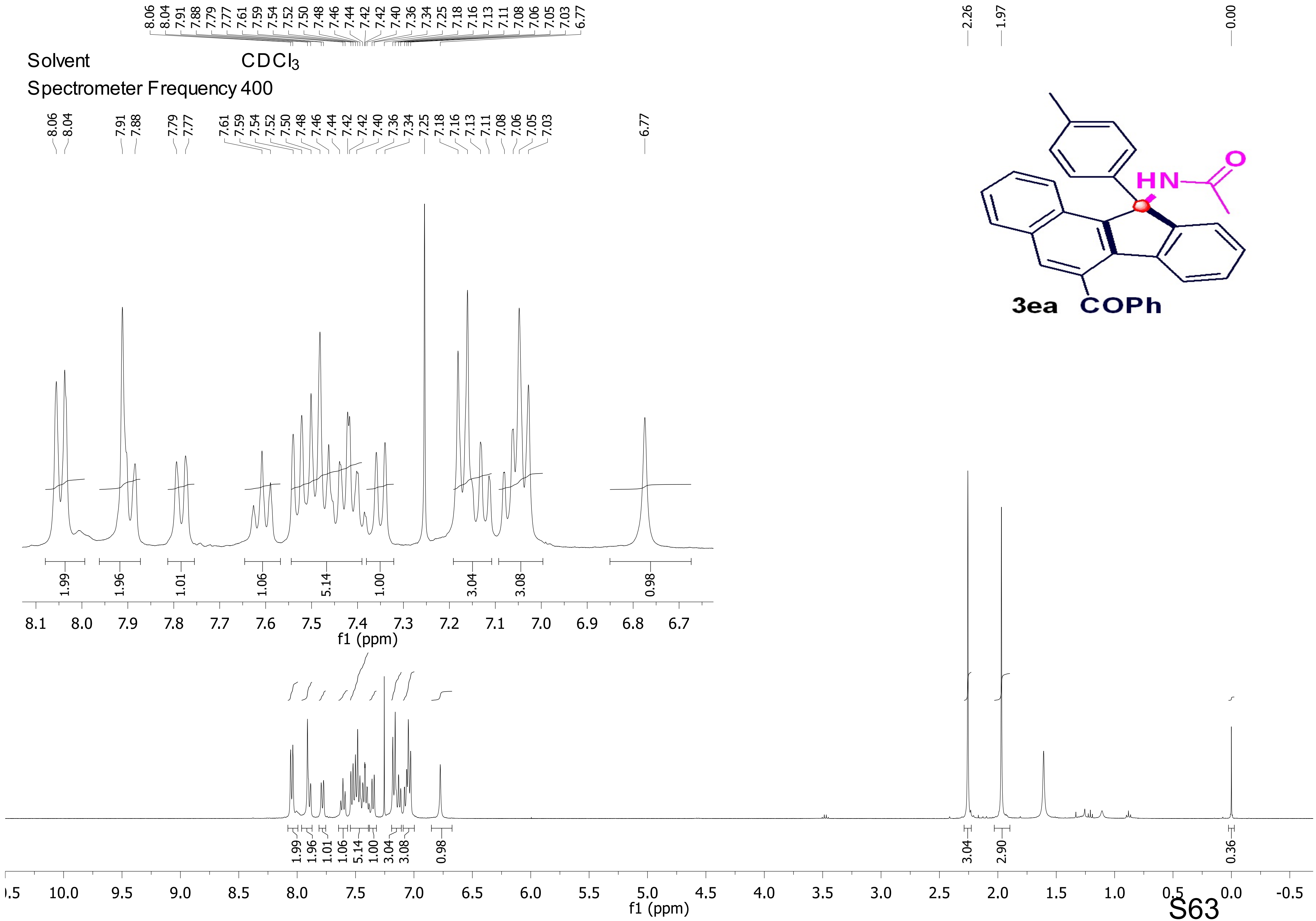

Solvent CDCl$_3$
Spectrometer Frequency 400
8.06 8.04 7.91 7.88 7.79 7.77 7.61 7.59 7.54 7.52 7.50 7.48 7.46 7.44 7.42 7.42 7.40 7.36 7.34 7.25 7.18 7.16 7.13 7.11 7.08 7.06 7.05 7.03 6.77
2.26
1.97
0.00
1.99 1.96 1.01 1.06 5.14 1.00 3.04 3.08 0.98
3.04 2.90 0.36
HN
O
3ea
COPh
f1 (ppm)

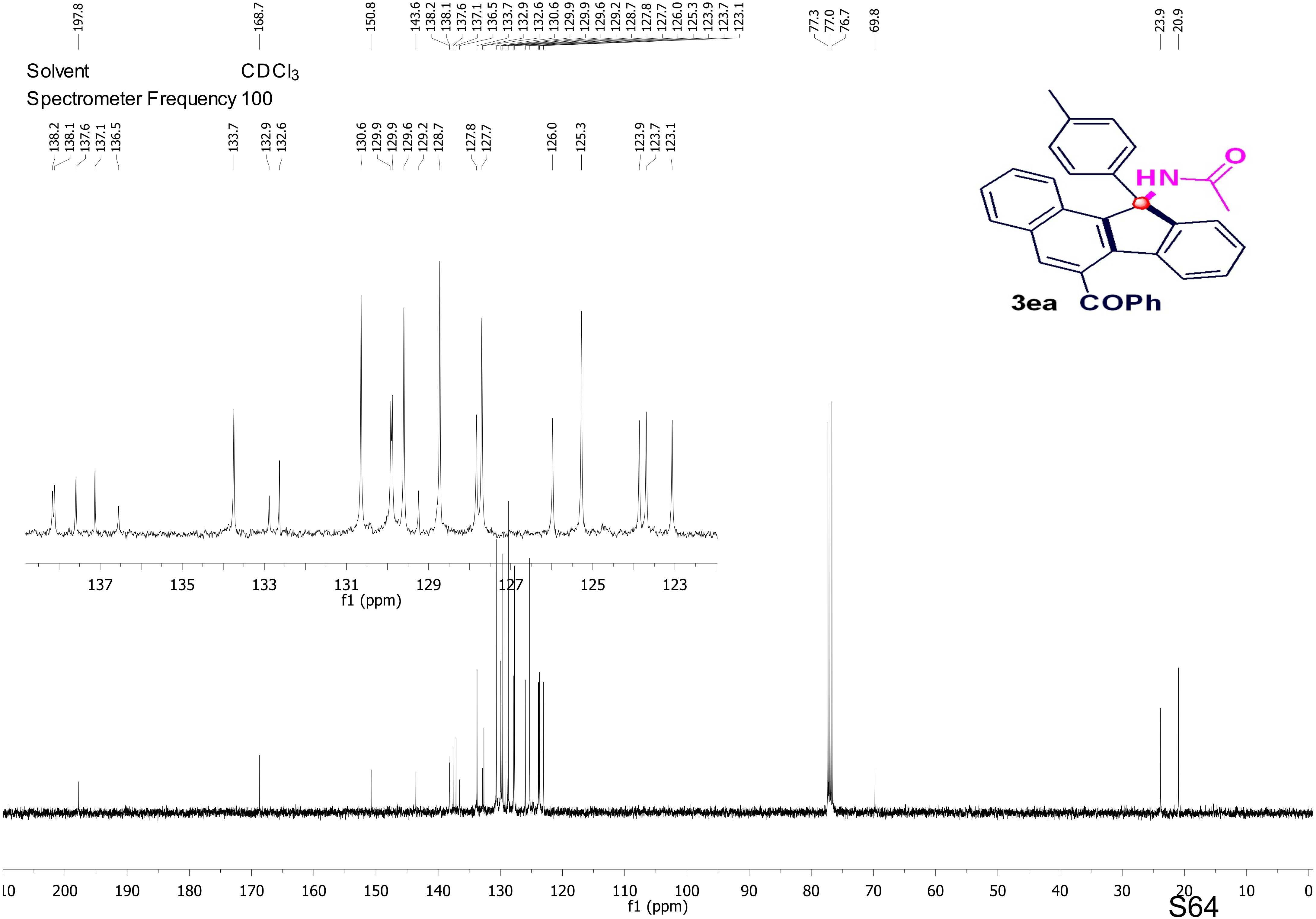
197.8
168.7
150.8
143.6
138.2
138.1
137.6
137.1
136.5
133.7
132.9
132.6
130.6
129.9
129.9
129.6
129.2
128.7
127.8
127.7
126.0
125.3
123.9
123.7
123.1
77.3
77.0
76.7
69.8
23.9
20.9
Solvent CDCl3
Spectrometer Frequency 100
HN
O
3ea
COPh
f1 (ppm)
f1 (ppm)

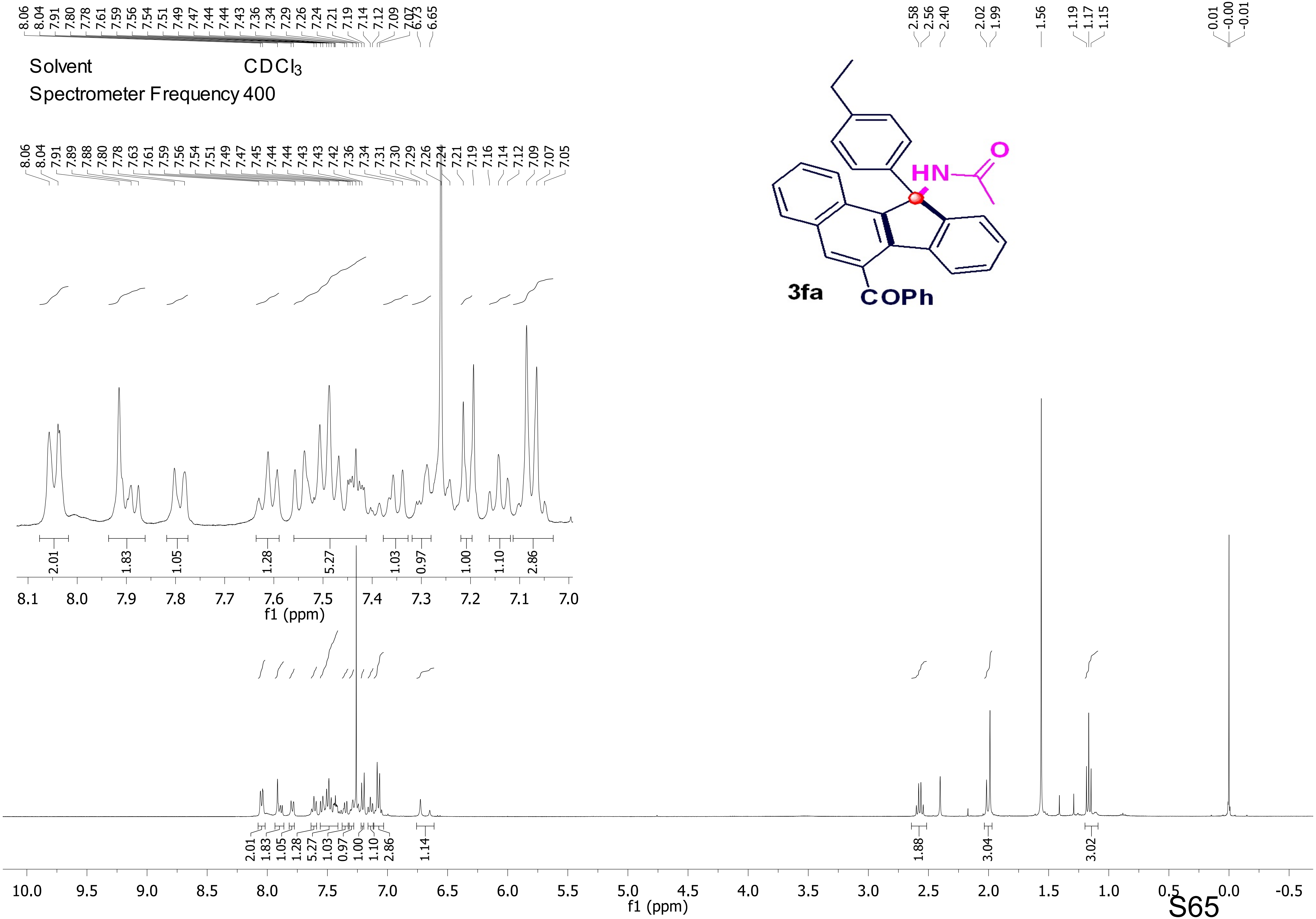
Solvent CDCl3
Spectrometer Frequency 400
HN
O
3fa
COPh
f1 (ppm)

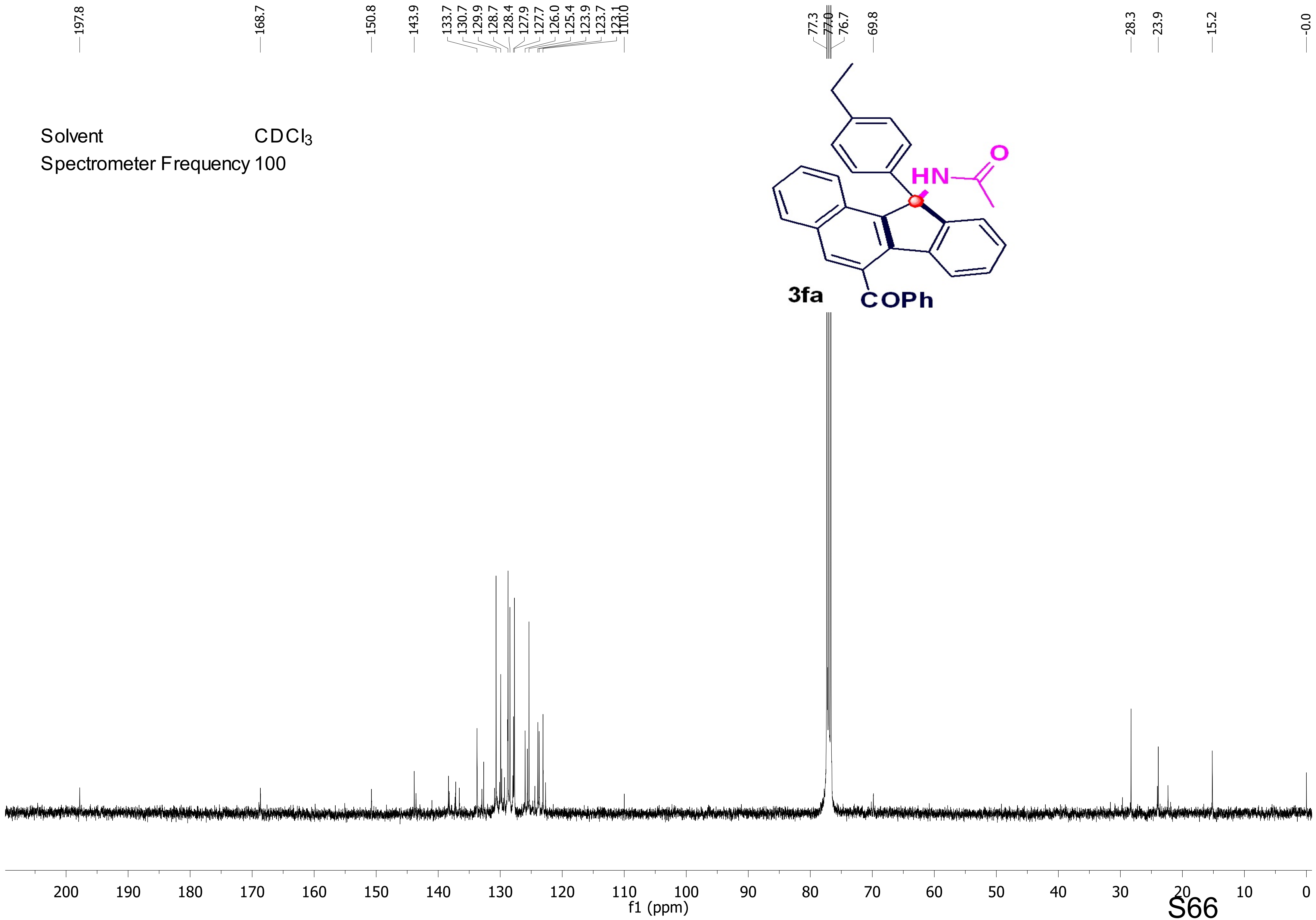
197.8
168.7
150.8
143.9
133.7
130.7
129.9
128.7
128.4
127.9
127.7
126.0
125.4
123.9
123.7
123.1
110.0
77.3
77.0
76.7
69.8
28.3
23.9
15.2
-0.0
Solvent CDCl3
Spectrometer Frequency 100
HN
O
3fa
COPh
200 190 180 170 160 150 140 130 120 110 100 90 80 70 60 50 40 30 20 10 0
f1 (ppm)

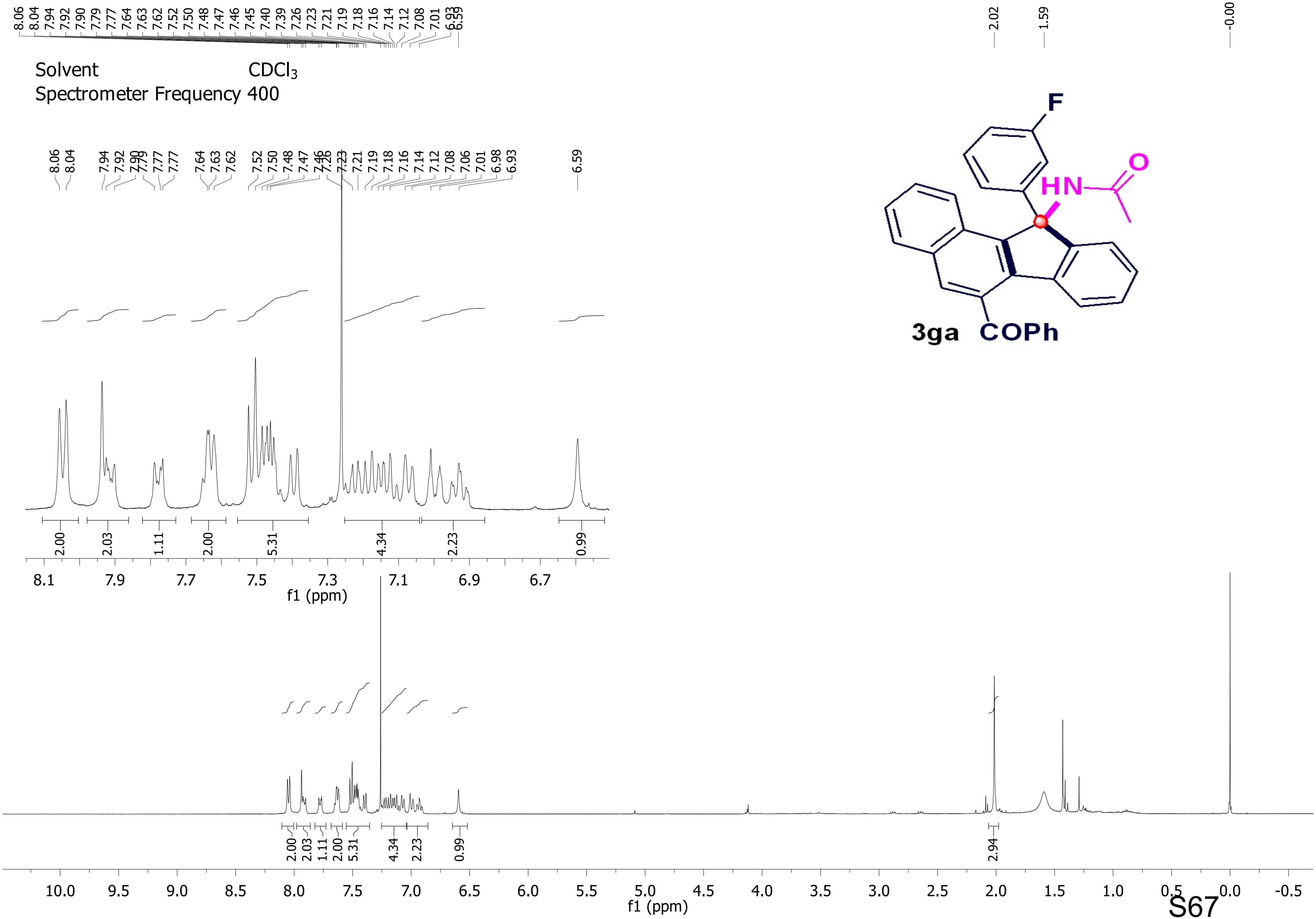
8.06 8.04 7.94 7.92 7.90 7.79 7.77 7.64 7.63 7.62 7.52 7.50 7.48 7.47 7.46 7.45 7.40 7.39 7.26 7.23 7.21 7.19 7.18 7.16 7.14 7.12 7.08 7.01 6.93 6.59
2.02
1.59
-0.00
Solvent CDCl3
Spectrometer Frequency 400
F
HN
O
3ga
COPh
2.00 2.03 1.11 2.00 5.31 4.34 2.23 0.99
2.94
f1 (ppm)

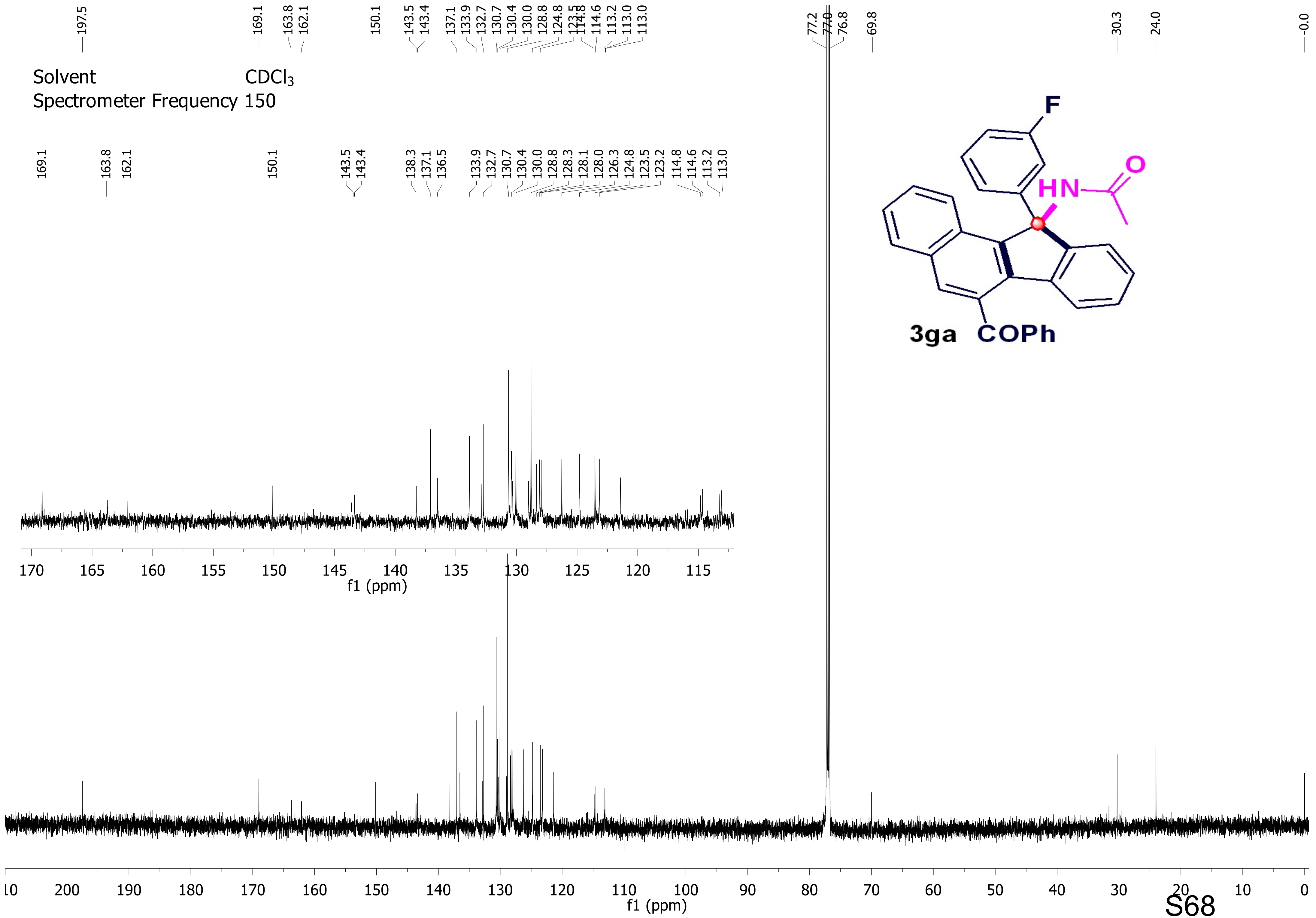
197.5
169.1
163.8
162.1
150.1
143.5
143.4
137.1
133.9
132.7
130.7
130.4
130.0
128.8
124.8
123.5
114.8
114.6
113.2
113.0
113.0
77.2
77.0
76.8
69.8
30.3
24.0
-0.0
Solvent CDCl3
Spectrometer Frequency 150
169.1
163.8
162.1
150.1
143.5
143.4
138.3
137.1
136.5
133.9
132.7
130.7
130.4
130.0
128.8
128.3
128.1
128.0
126.3
124.8
123.5
123.2
114.8
114.6
113.2
113.0
f1 (ppm)
F
HN
O
3ga
COPh
f1 (ppm)

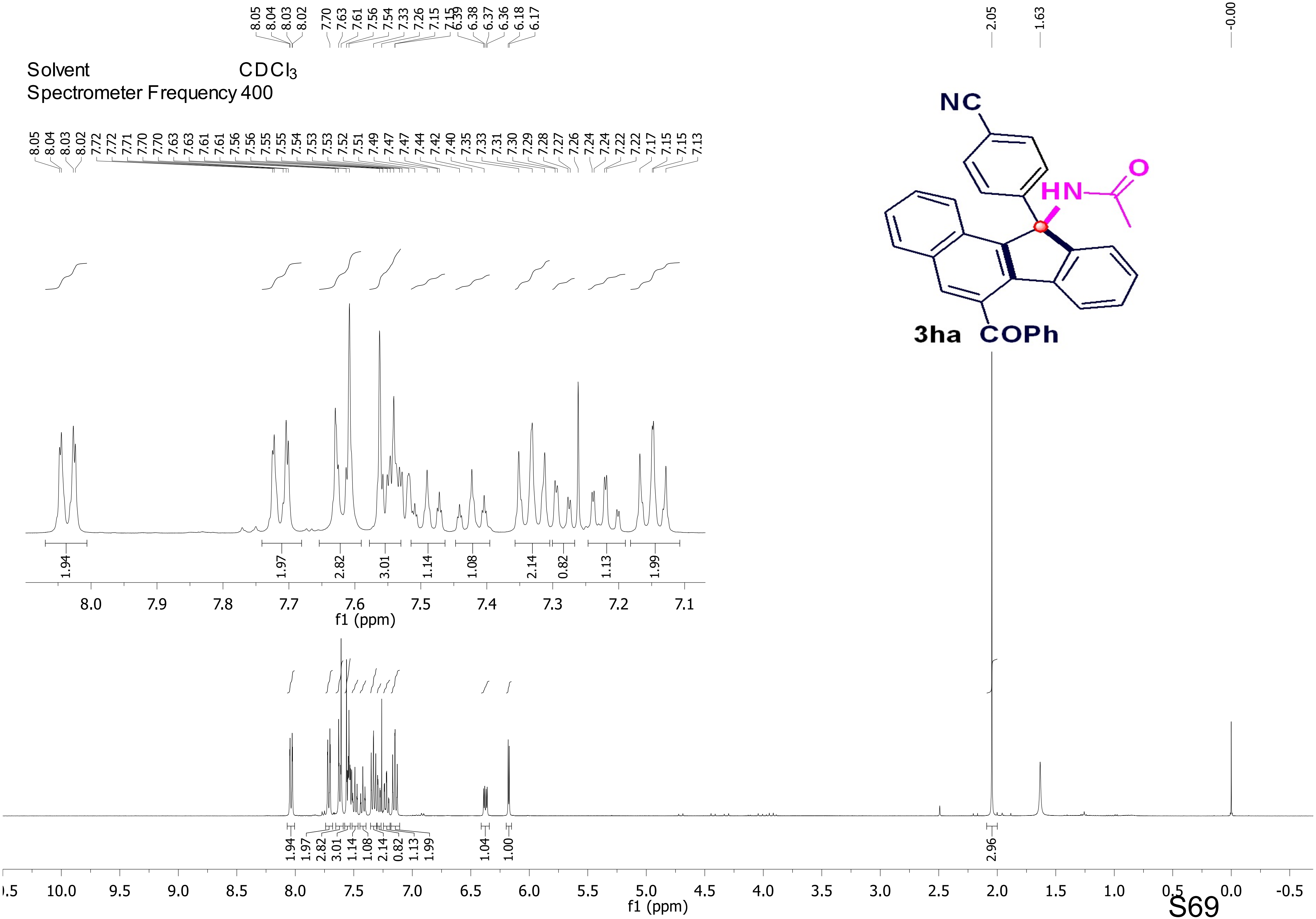
Solvent CDCl3
Spectrometer Frequency 400
NC
HN
O
3ha
COPh
f1 (ppm)

Solvent $CDCl_3$
Spectrometer Frequency 100

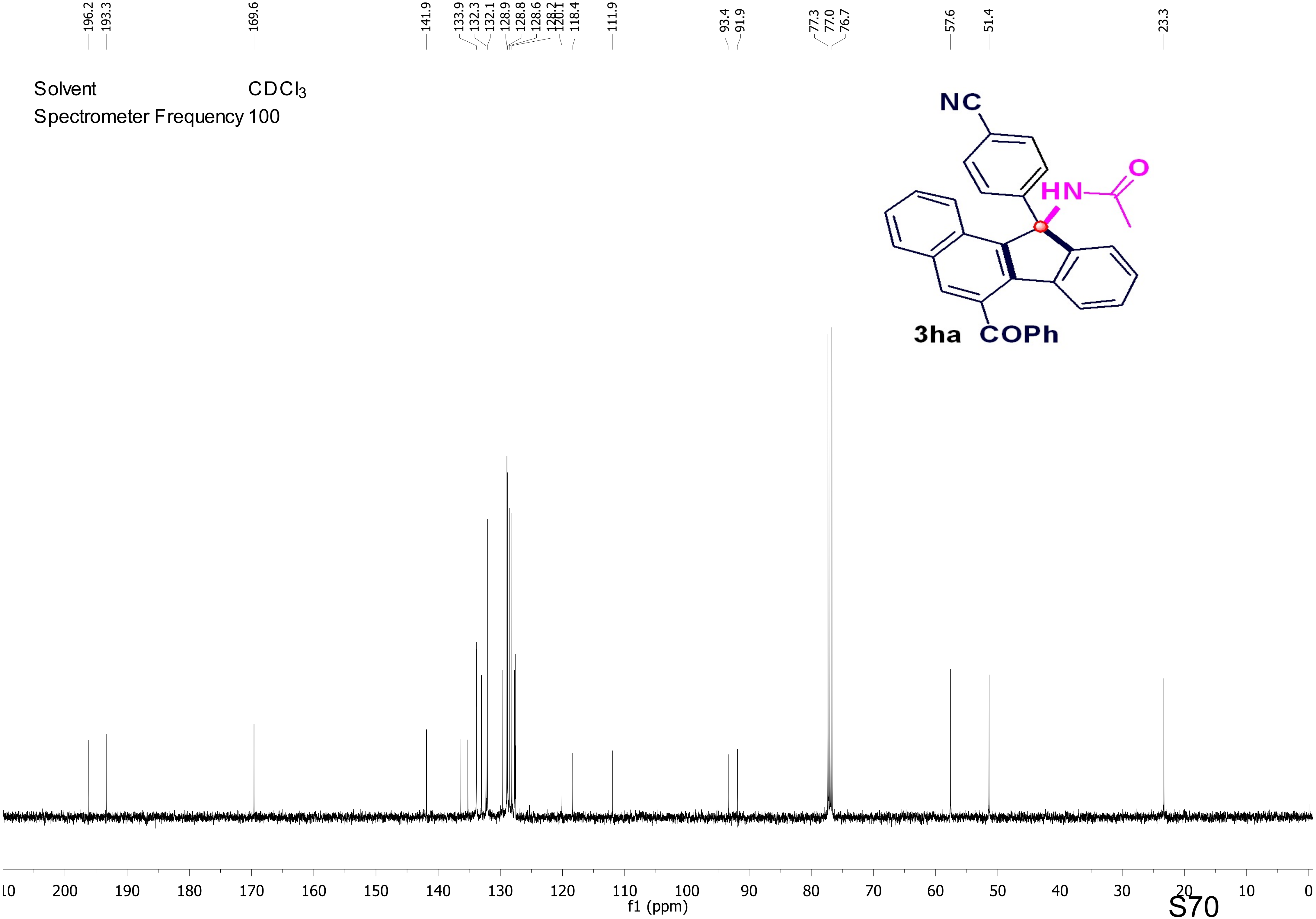

3ha

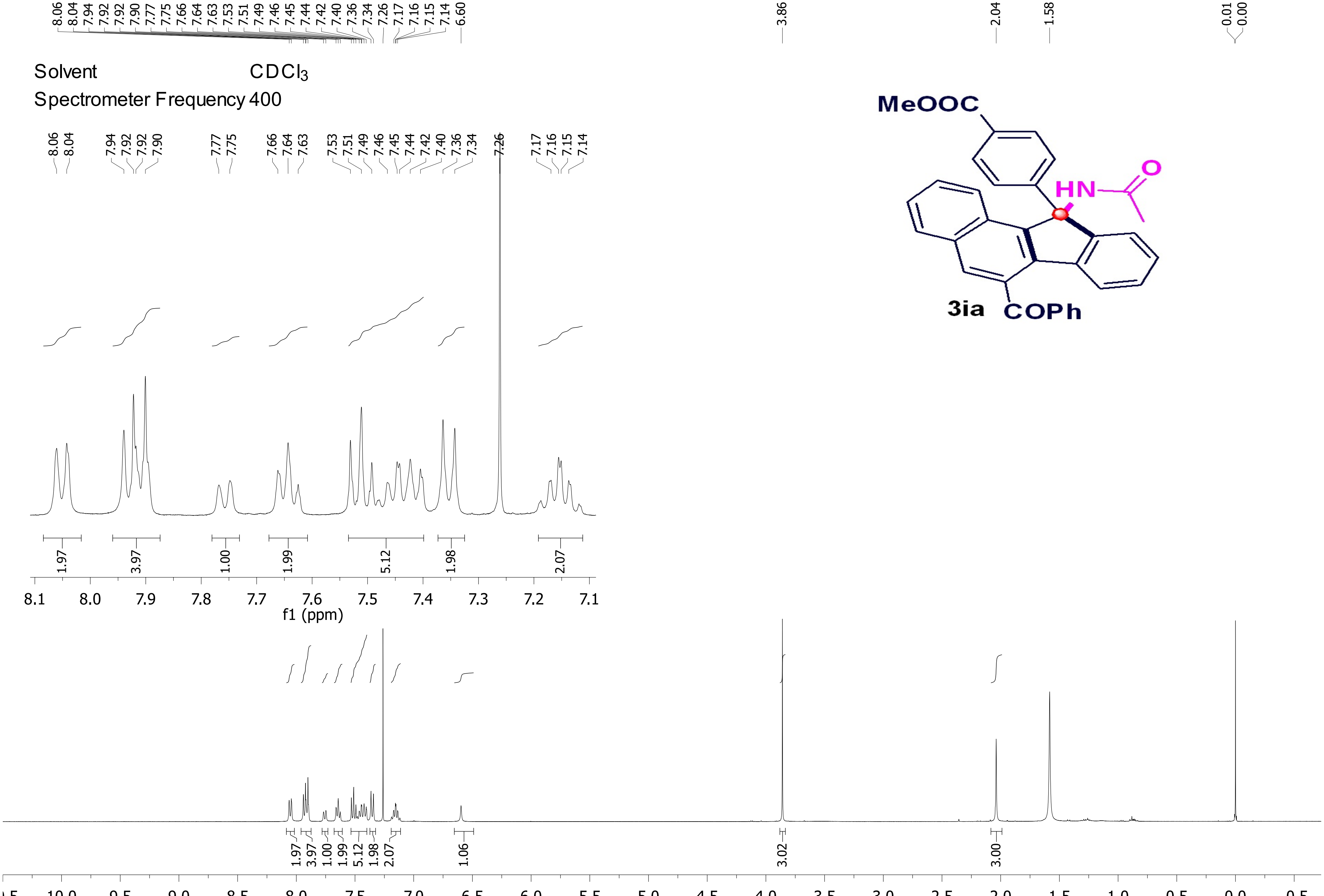
8.06 8.04 7.94 7.92 7.92 7.90 7.77 7.75 7.66 7.64 7.63 7.53 7.51 7.49 7.46 7.45 7.44 7.42 7.40 7.36 7.34 7.26 7.17 7.16 7.15 7.14 6.60
3.86
2.04
1.58
0.01 0.00
Solvent CDCl3
Spectrometer Frequency 400
MeOOC
HN
O
3ia
COPh
1.97 3.97 1.00 1.99 5.12 1.98 2.07
f1 (ppm)
1.97 3.97 1.00 1.99 5.12 1.98 2.07 1.06 3.02 3.00
f1 (ppm)

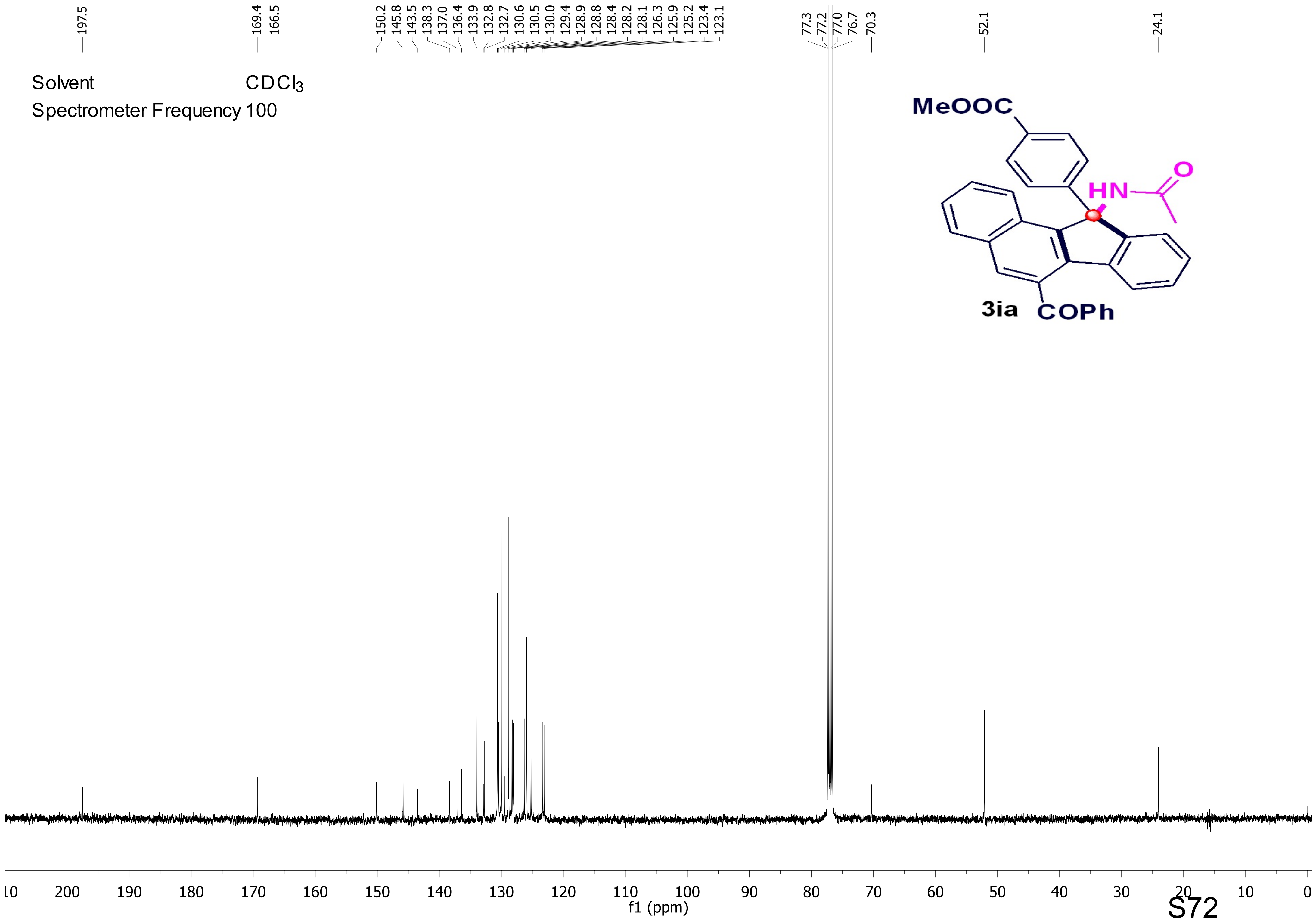
197.5
169.4
166.5
150.2
145.8
143.5
138.3
137.0
136.4
133.9
132.8
132.7
130.6
130.5
130.0
129.4
128.9
128.8
128.4
128.2
128.1
126.3
125.9
125.2
123.4
123.1
77.3
77.2
77.0
76.7
70.3
52.1
24.1
Solvent CDCl3
Spectrometer Frequency 100
MeOOC
HN
O
3ia
COPh
200 190 180 170 160 150 140 130 120 110 100 90 80 70 60 50 40 30 20 10 0
f1 (ppm)

Solvent CDCl3
Spectrometer Frequency 400

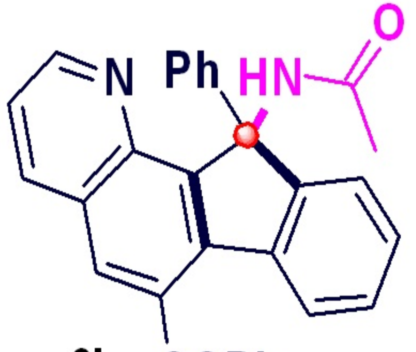

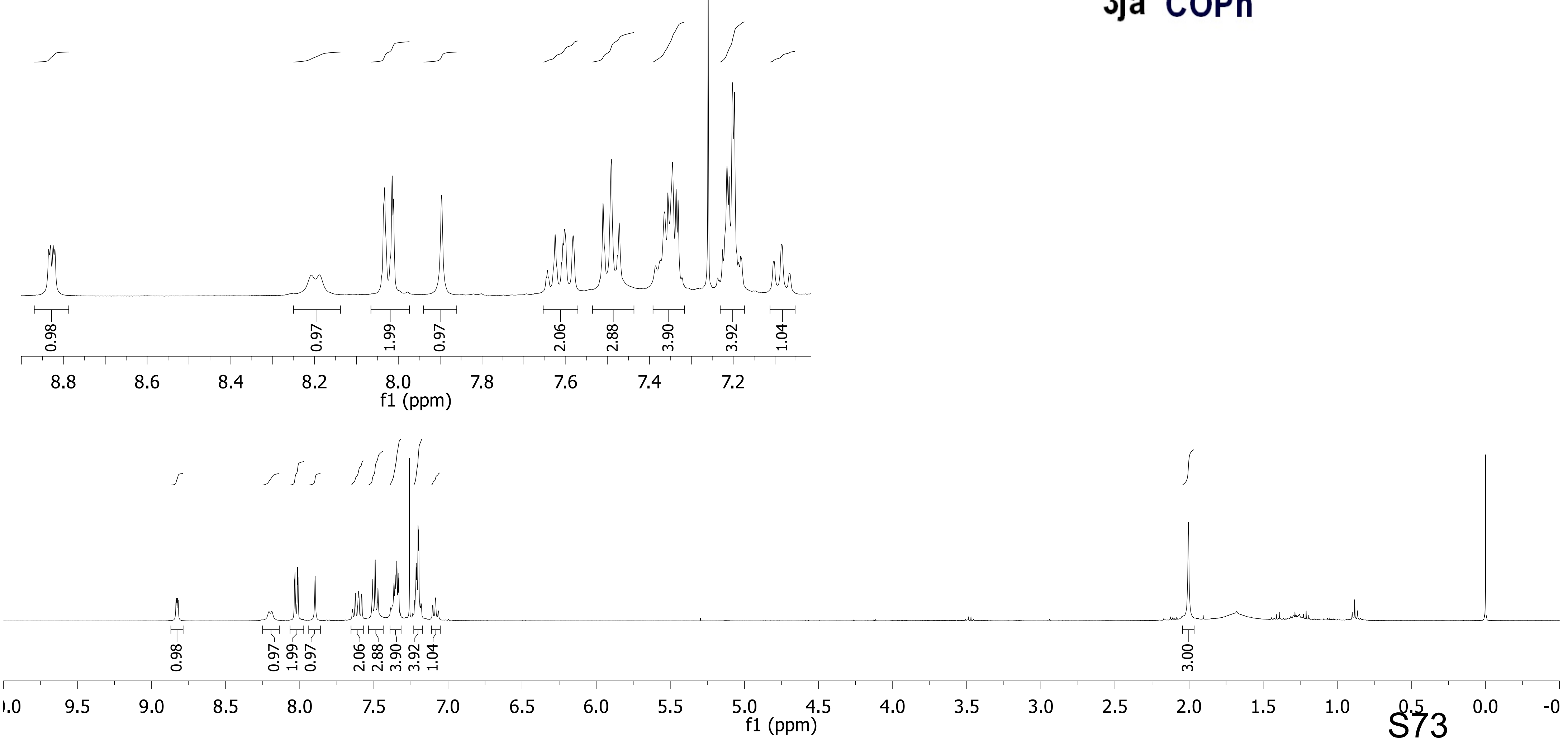

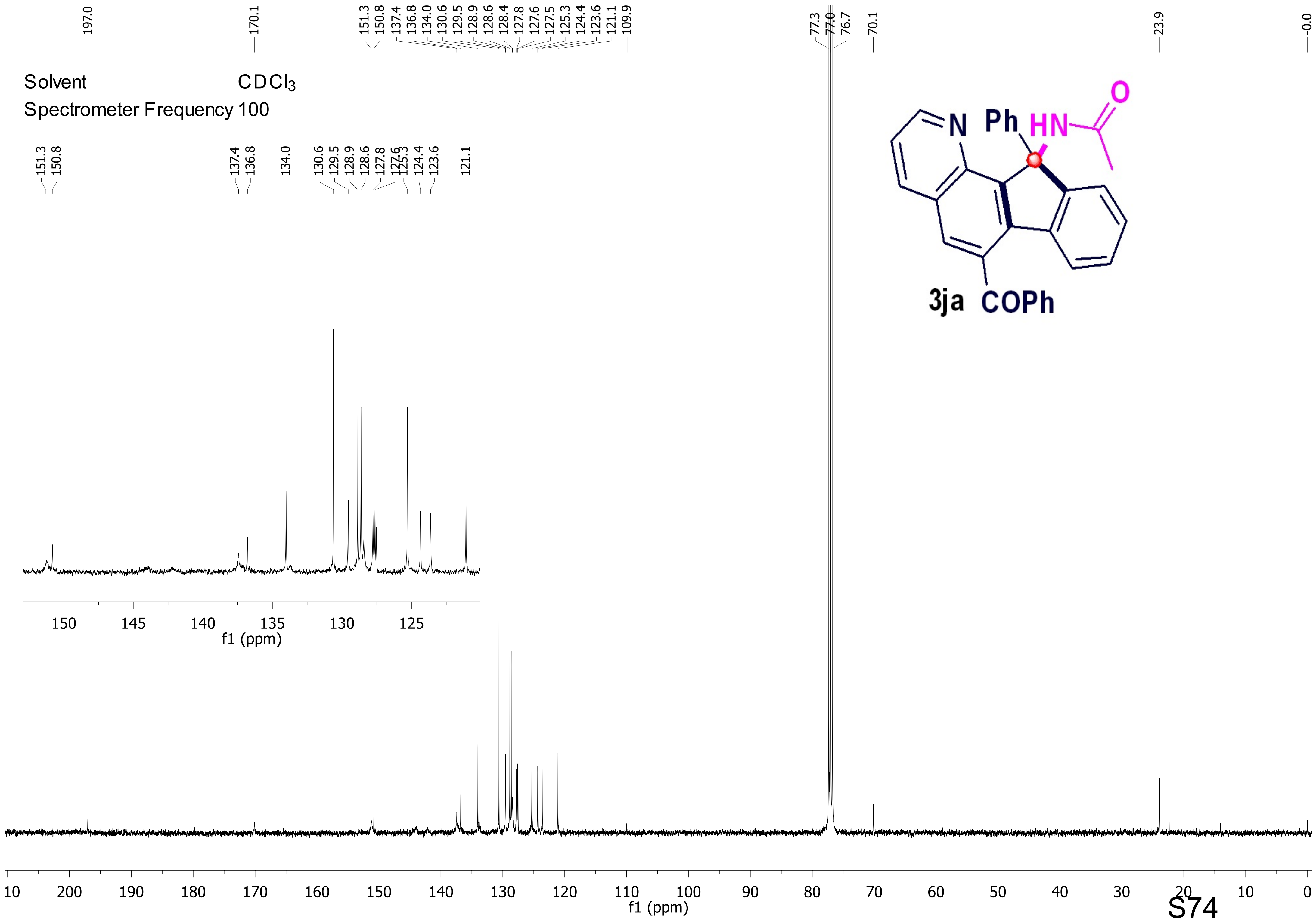
197.0
170.1
151.3 150.8 137.4 136.8 134.0 130.6 129.5 128.9 128.6 128.4 127.8 127.6 127.5 125.3 124.4 123.6 121.1 109.9
77.3 77.0 76.7 70.1
23.9
-0.0
Solvent CDCl3
Spectrometer Frequency 100
151.3 150.8 137.4 136.8 134.0 130.6 129.5 128.9 128.6 127.8 127.6 127.5 125.3 124.4 123.6 121.1
150 145 140 135 130 125
f1 (ppm)
N
Ph
HN
O
3ja
COPh
10 200 190 180 170 160 150 140 130 120 110 100 90 80 70 60 50 40 30 20 10 0
f1 (ppm)

Solvent CDCl3
Spectrometer Frequency 400

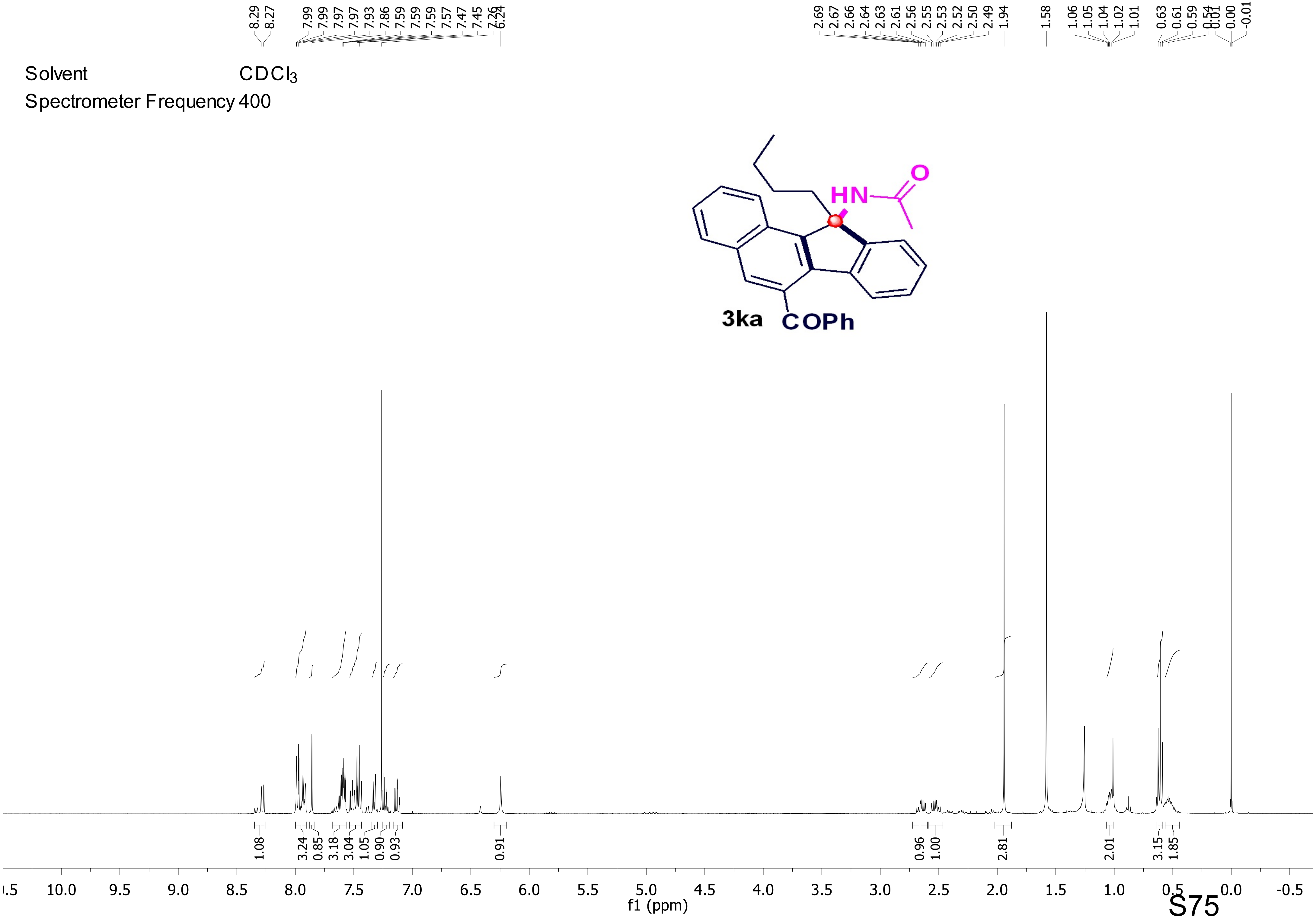

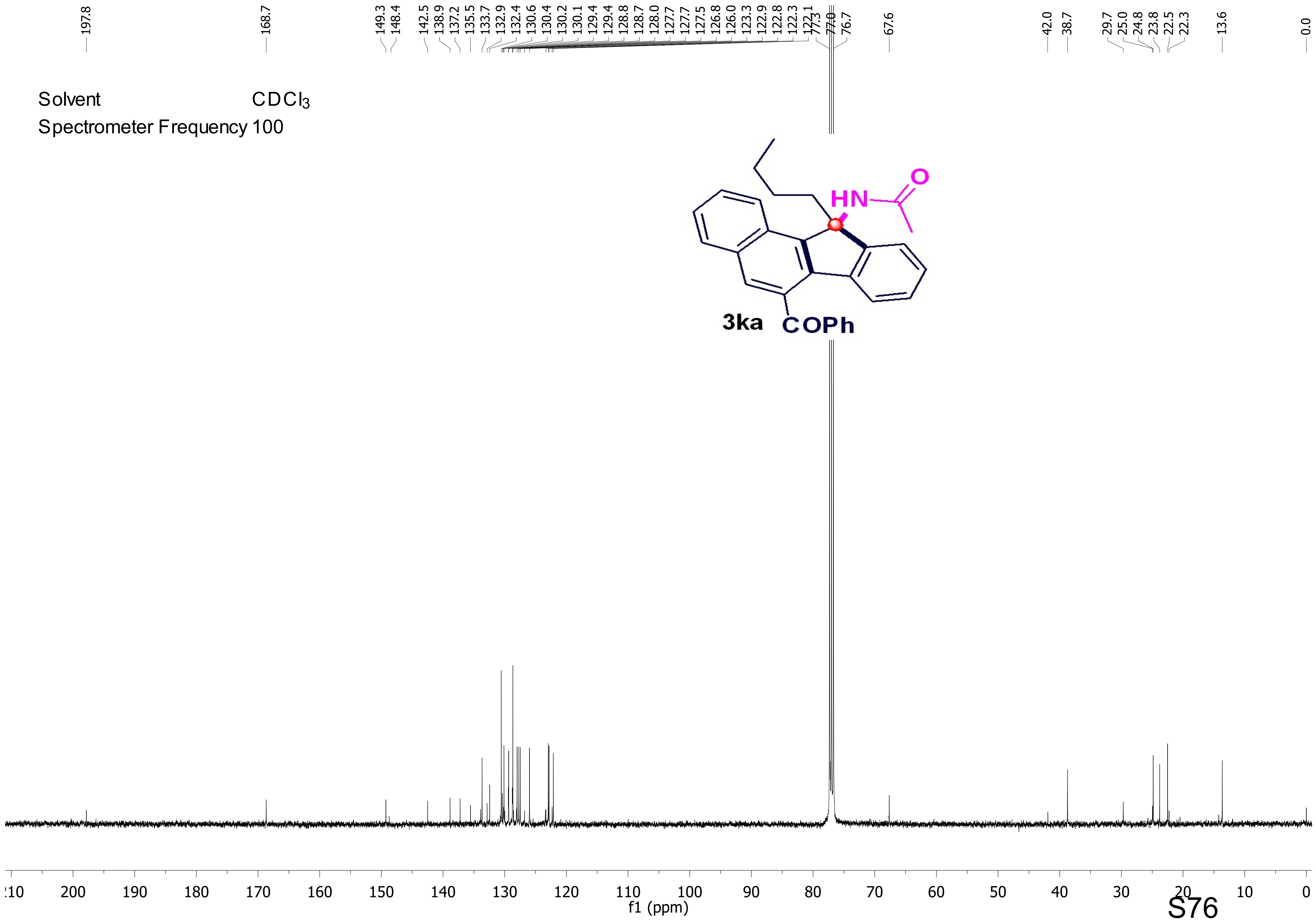

197.8
168.7
149.3
148.4
142.5
138.9
137.2
135.5
133.7
132.9
132.4
130.6
130.4
130.2
130.1
129.4
129.4
128.8
128.7
128.0
127.7
127.7
127.5
126.8
126.0
123.3
122.9
122.8
122.3
122.1
77.3
77.0
76.7
67.6
42.0
38.7
29.7
25.0
24.8
23.8
22.5
22.3
13.6
0.0
Solvent CDCl3
Spectrometer Frequency 100
HN
O
3ka
COPh
f1 (ppm)

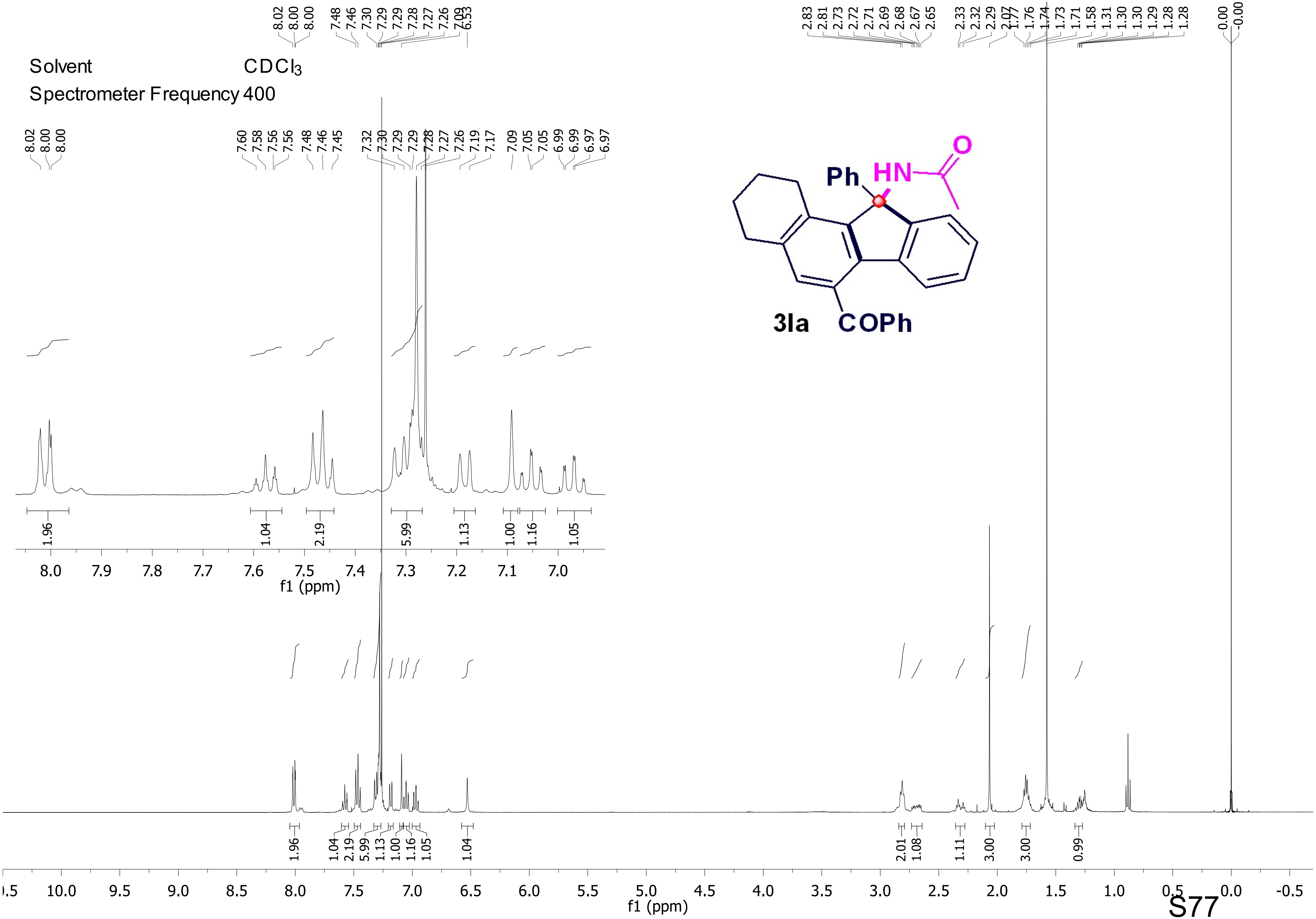
Solvent CDCl$_3$
Spectrometer Frequency 400
Ph
HN
O
3la
COPh
f1 (ppm)

Solvent CDCl$_3$
Spectrometer Frequency 100

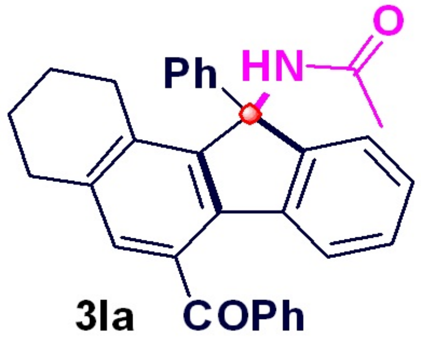

3la

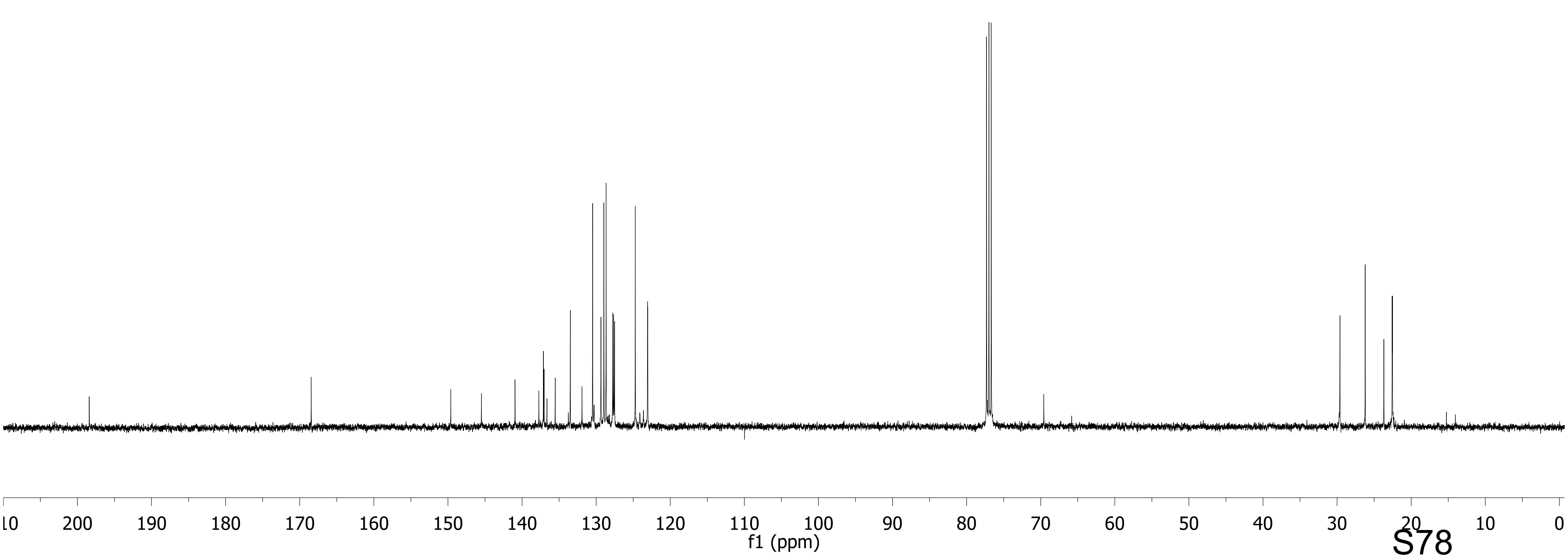

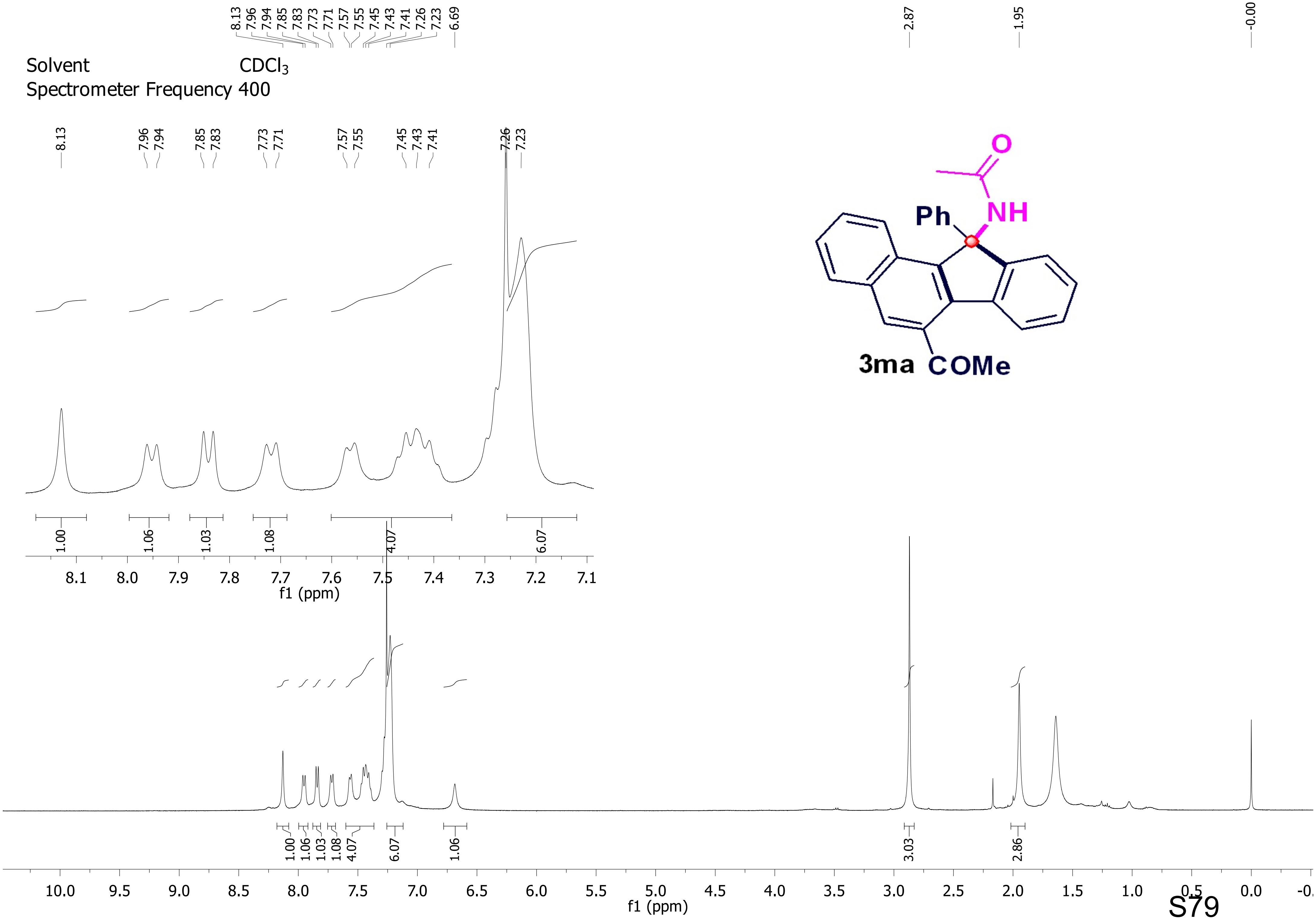
Solvent CDCl3
Spectrometer Frequency 400
8.13 7.96 7.94 7.85 7.83 7.73 7.71 7.57 7.55 7.45 7.43 7.41 7.26 7.23 6.69
2.87
1.95
-0.00
1.00 1.06 1.03 1.08 4.07 6.07
1.00 1.06 1.03 1.08 4.07 6.07 1.06 3.03 2.86
f1 (ppm)
O
NH
Ph
3ma
COMe

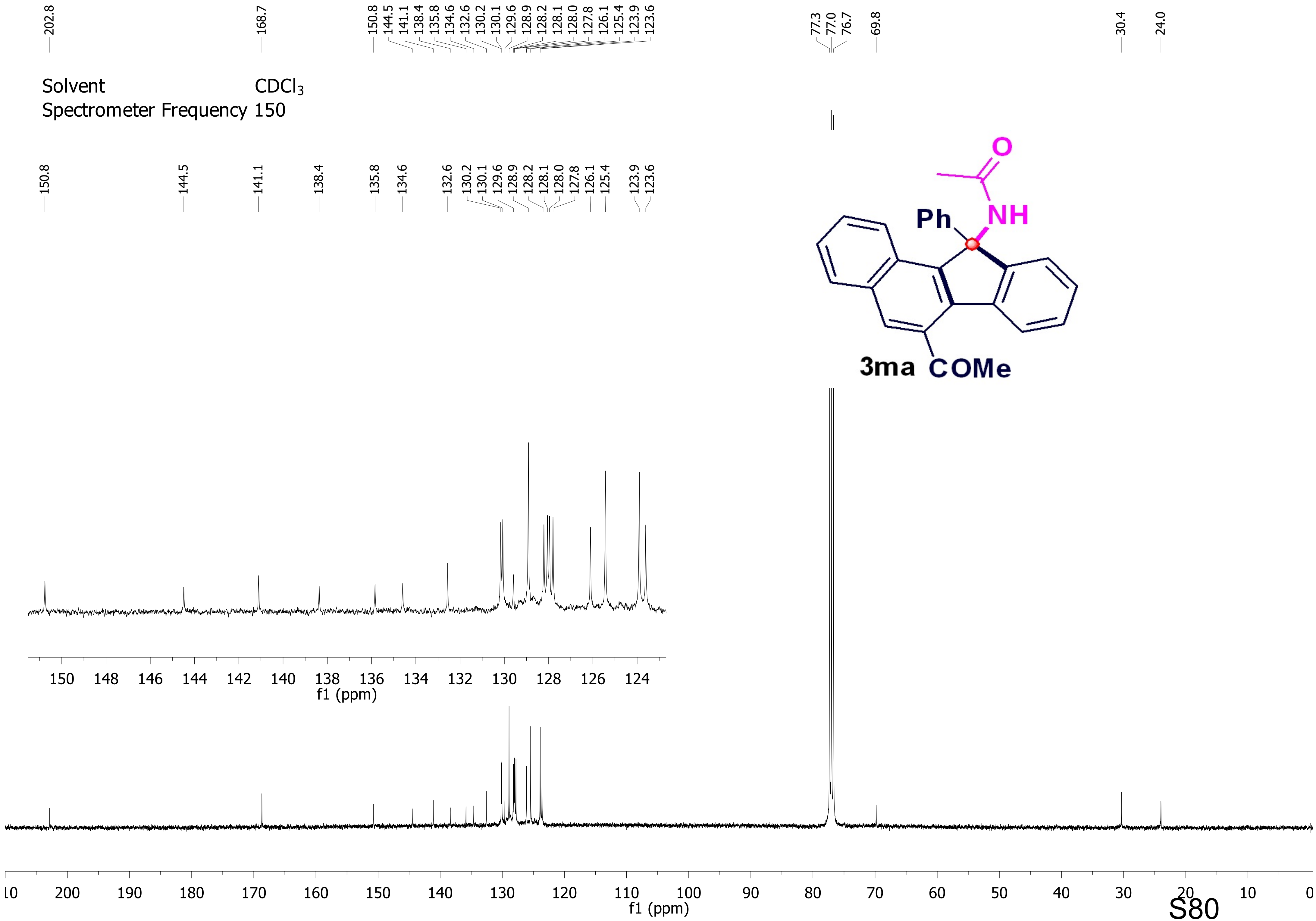
202.8
168.7
150.8
144.5
141.1
138.4
135.8
134.6
132.6
130.2
130.1
129.6
128.9
128.2
128.1
128.0
127.8
126.1
125.4
123.9
123.6
77.3
77.0
76.7
69.8
30.4
24.0
Solvent CDCl3
Spectrometer Frequency 150
O
NH
Ph
3ma
COMe
f1 (ppm)
f1 (ppm)

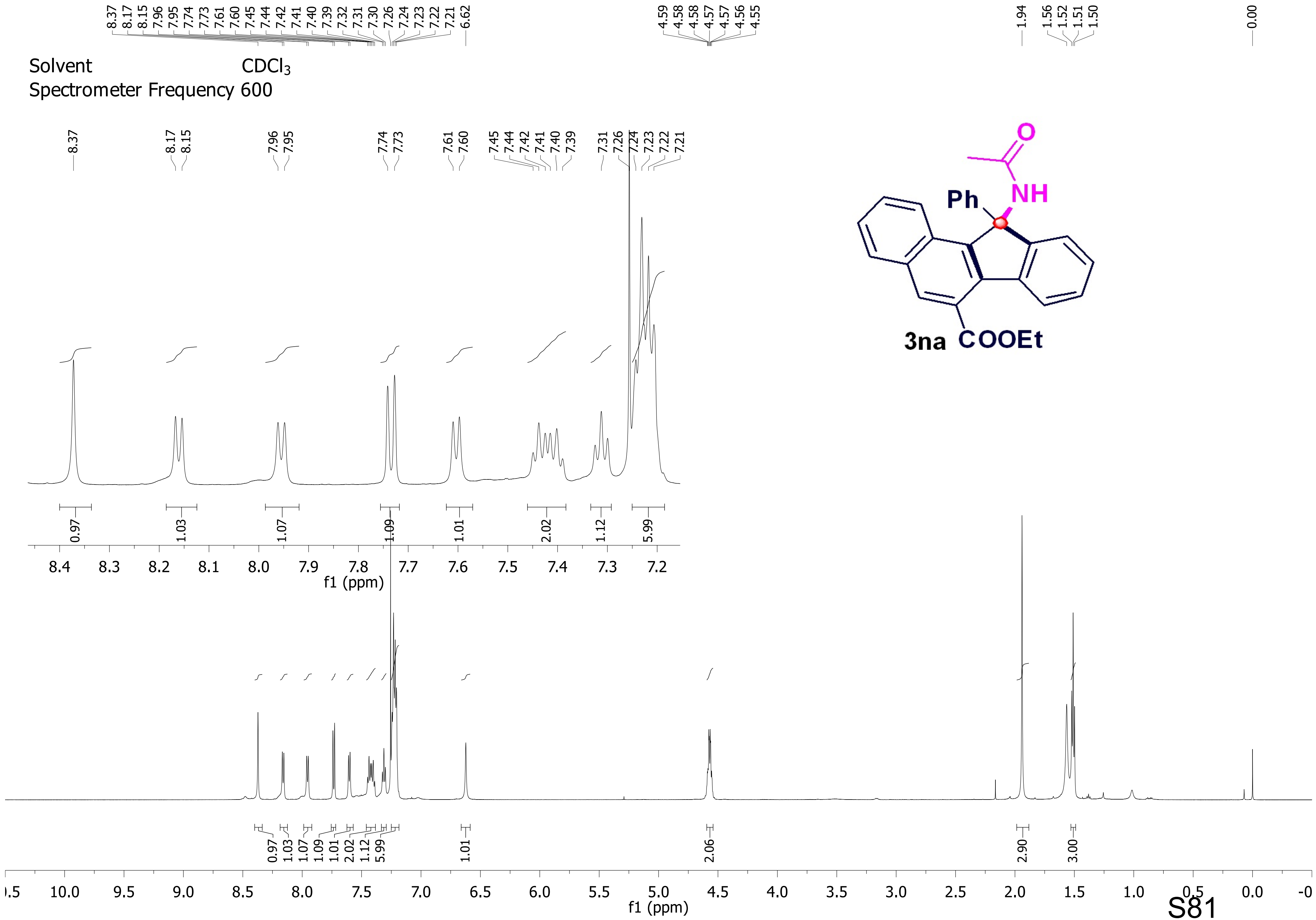
8.37 8.17 8.15 7.96 7.95 7.74 7.73 7.61 7.60 7.45 7.44 7.42 7.41 7.40 7.39 7.32 7.31 7.30 7.26 7.24 7.23 7.22 7.21 6.62
4.59 4.58 4.58 4.57 4.57 4.56 4.55
1.94 1.56 1.52 1.51 1.50
0.00
Solvent CDCl3
Spectrometer Frequency 600
Ph
NH
O
3na
COOEt
0.97 1.03 1.07 1.09 1.01 2.02 1.12 5.99
1.01 2.06 2.90 3.00
f1 (ppm)

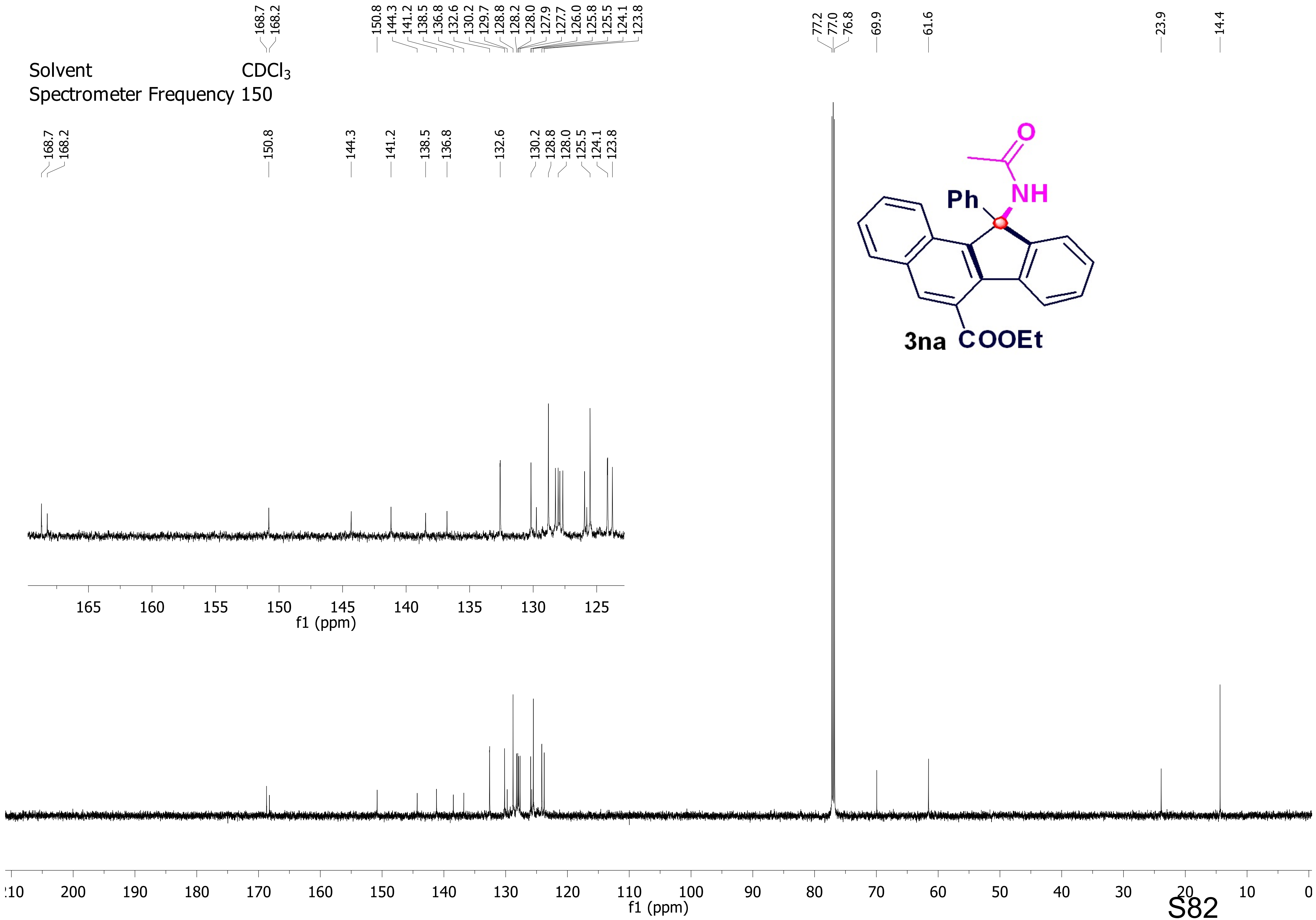
168.7
168.2
150.8
144.3
141.2
138.5
136.8
132.6
130.2
129.7
128.8
128.2
128.0
127.9
127.7
126.0
125.8
125.5
124.1
123.8
77.2
77.0
76.8
69.9
61.6
23.9
14.4
Solvent CDCl3
Spectrometer Frequency 150
O
NH
Ph
3na
COOEt
f1 (ppm)

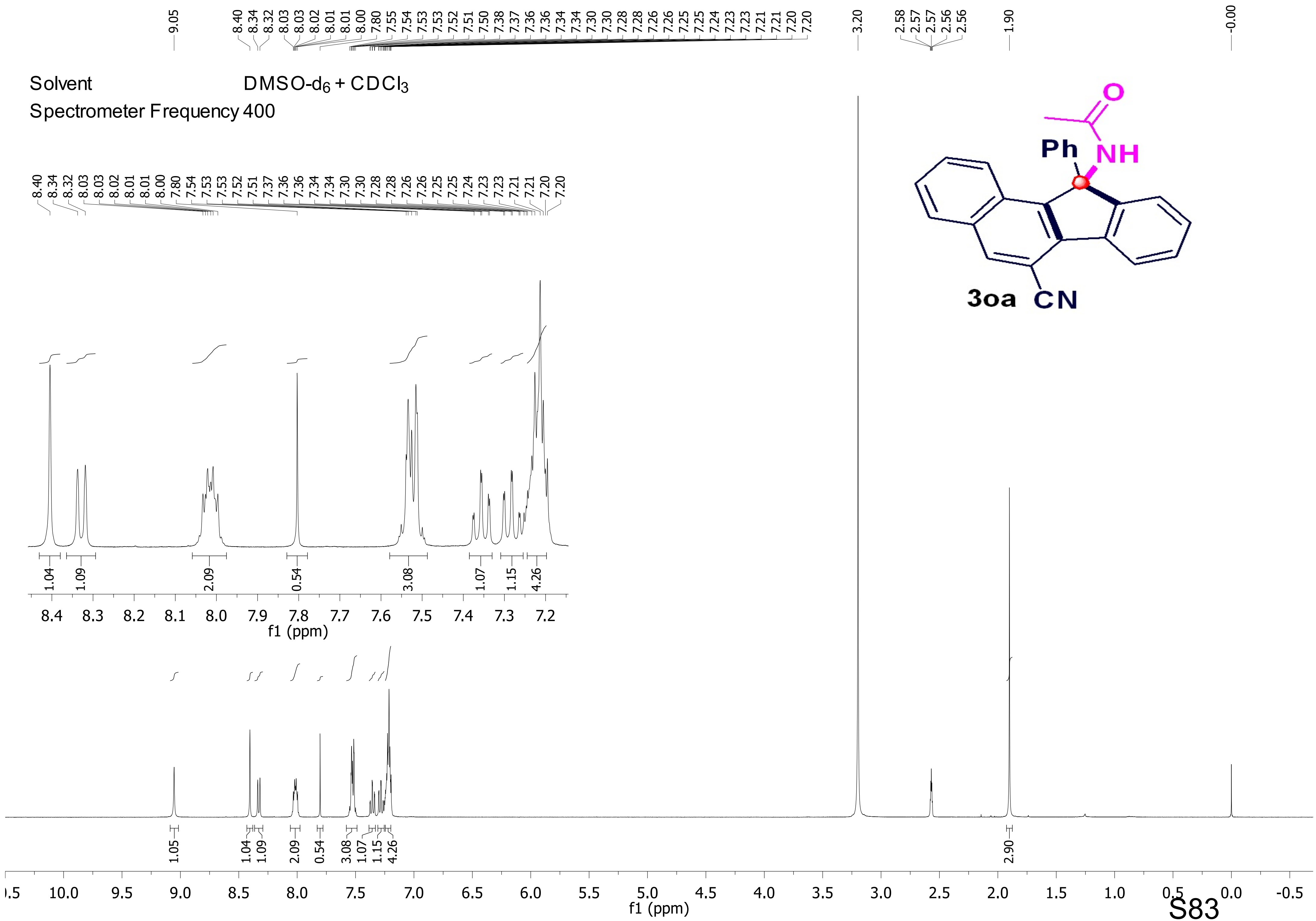
Solvent DMSO-d6 + CDCl3
Spectrometer Frequency 400
Ph
NH
O
CN
3oa
f1 (ppm)

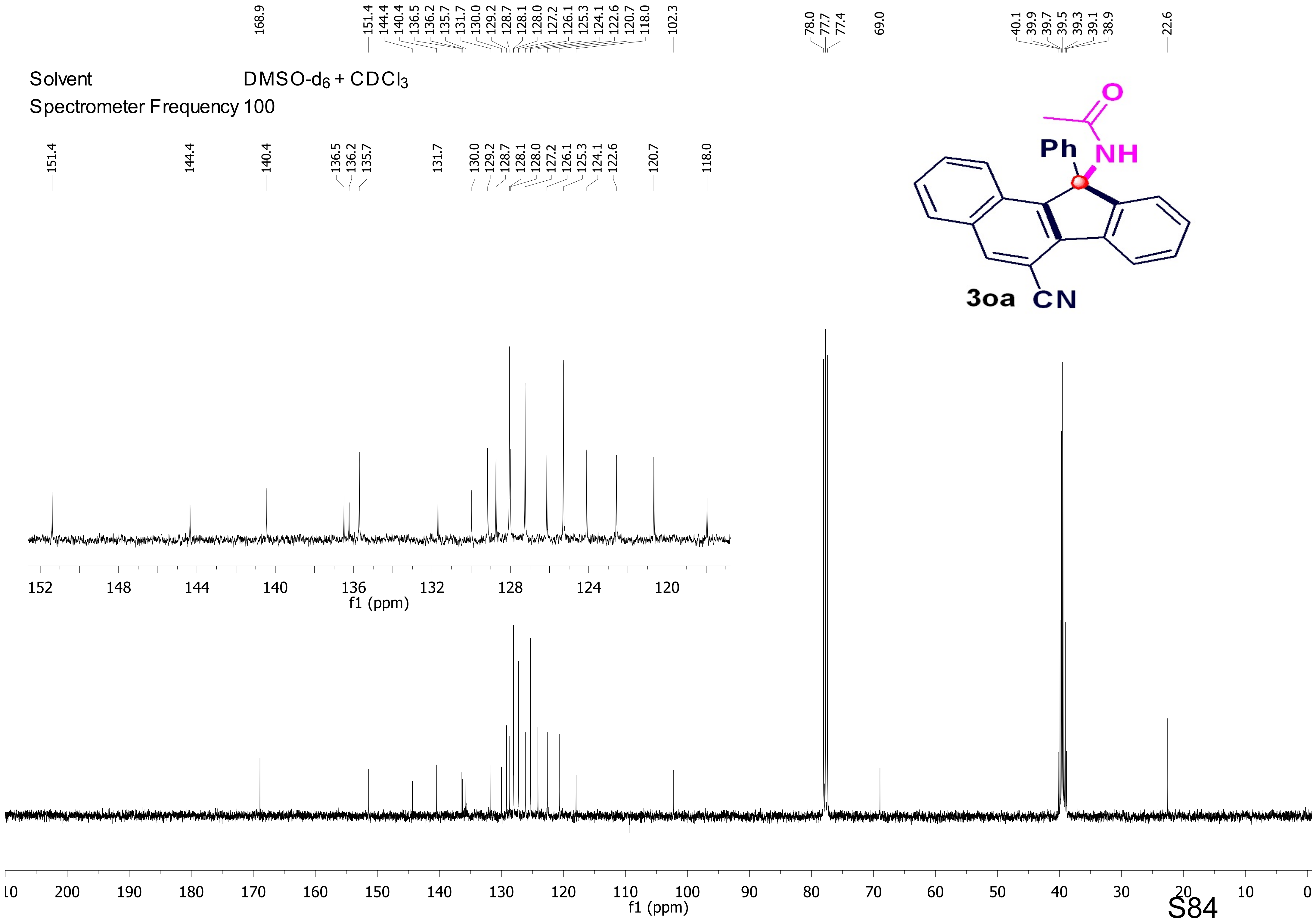
168.9
151.4
144.4
140.4
136.5
136.2
135.7
131.7
130.0
129.2
128.7
128.1
128.0
127.2
126.1
125.3
124.1
122.6
120.7
118.0
102.3
78.0
77.7
77.4
69.0
40.1
39.9
39.7
39.5
39.3
39.1
38.9
22.6
Solvent DMSO-d6 + CDCl3
Spectrometer Frequency 100
O
Ph
NH
3oa
CN
f1 (ppm)
f1 (ppm)

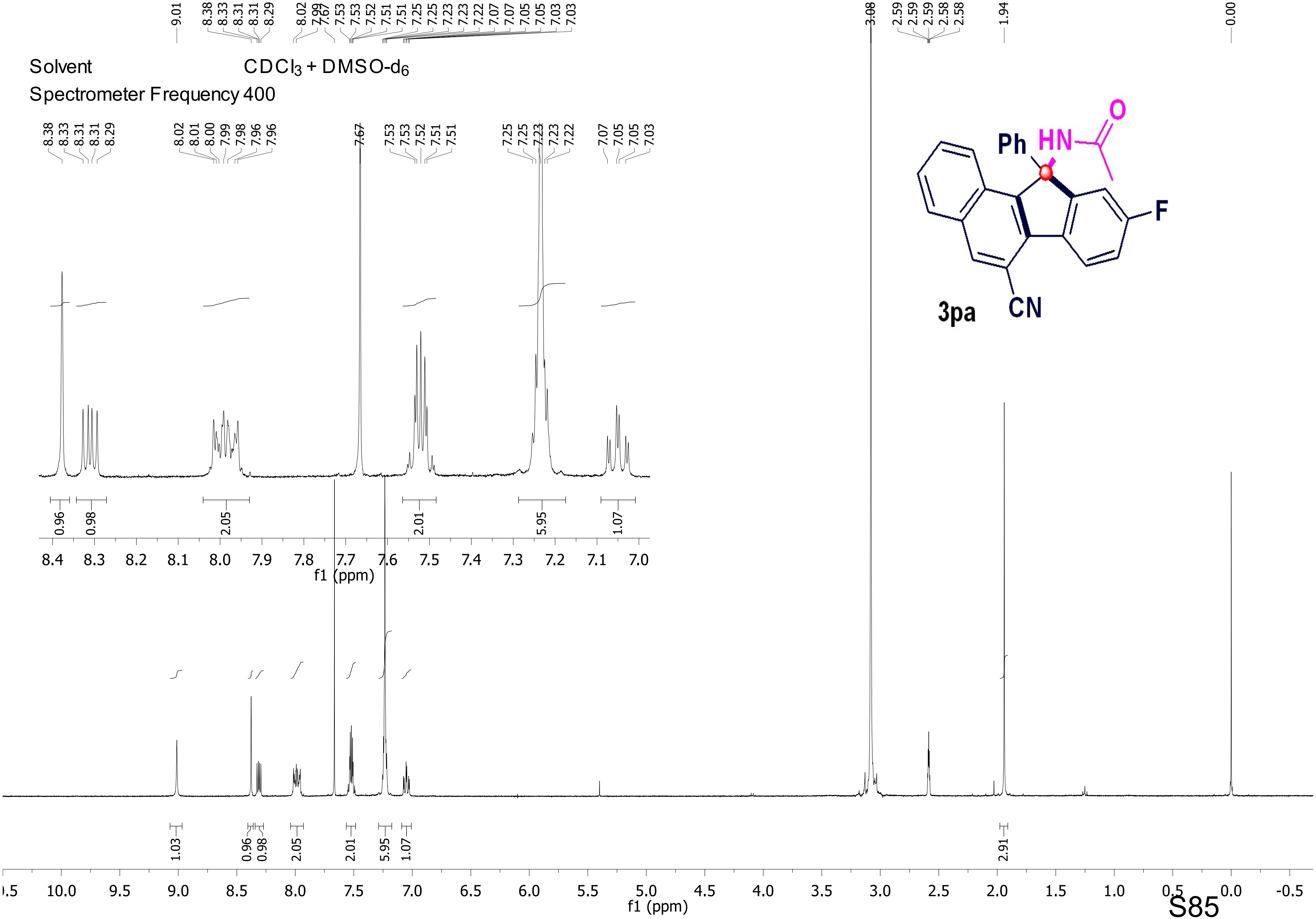
9.01 8.38 8.33 8.31 8.31 8.29 8.02 7.99 7.67 7.53 7.53 7.52 7.51 7.51 7.25 7.25 7.23 7.23 7.22 7.07 7.07 7.05 7.05 7.03 7.03
3.08 2.59 2.59 2.59 2.58 2.58 1.94 0.00
Solvent CDCl3 + DMSO-d6
Spectrometer Frequency 400
8.38 8.33 8.31 8.31 8.29 8.02 8.01 8.00 7.99 7.98 7.96 7.96 7.67 7.53 7.53 7.52 7.51 7.51 7.25 7.25 7.23 7.23 7.22 7.07 7.05 7.05 7.03
0.96 0.98 2.05 2.01 5.95 1.07
8.4 8.3 8.2 8.1 8.0 7.9 7.8 7.7 7.6 7.5 7.4 7.3 7.2 7.1 7.0
f1 (ppm)
Ph
HN
O
F
CN
3pa
1.03 0.96 0.98 2.05 2.01 5.95 1.07 2.91
10.0 9.5 9.0 8.5 8.0 7.5 7.0 6.5 6.0 5.5 5.0 4.5 4.0 3.5 3.0 2.5 2.0 1.5 1.0 0.5 0.0 -0.5
f1 (ppm)

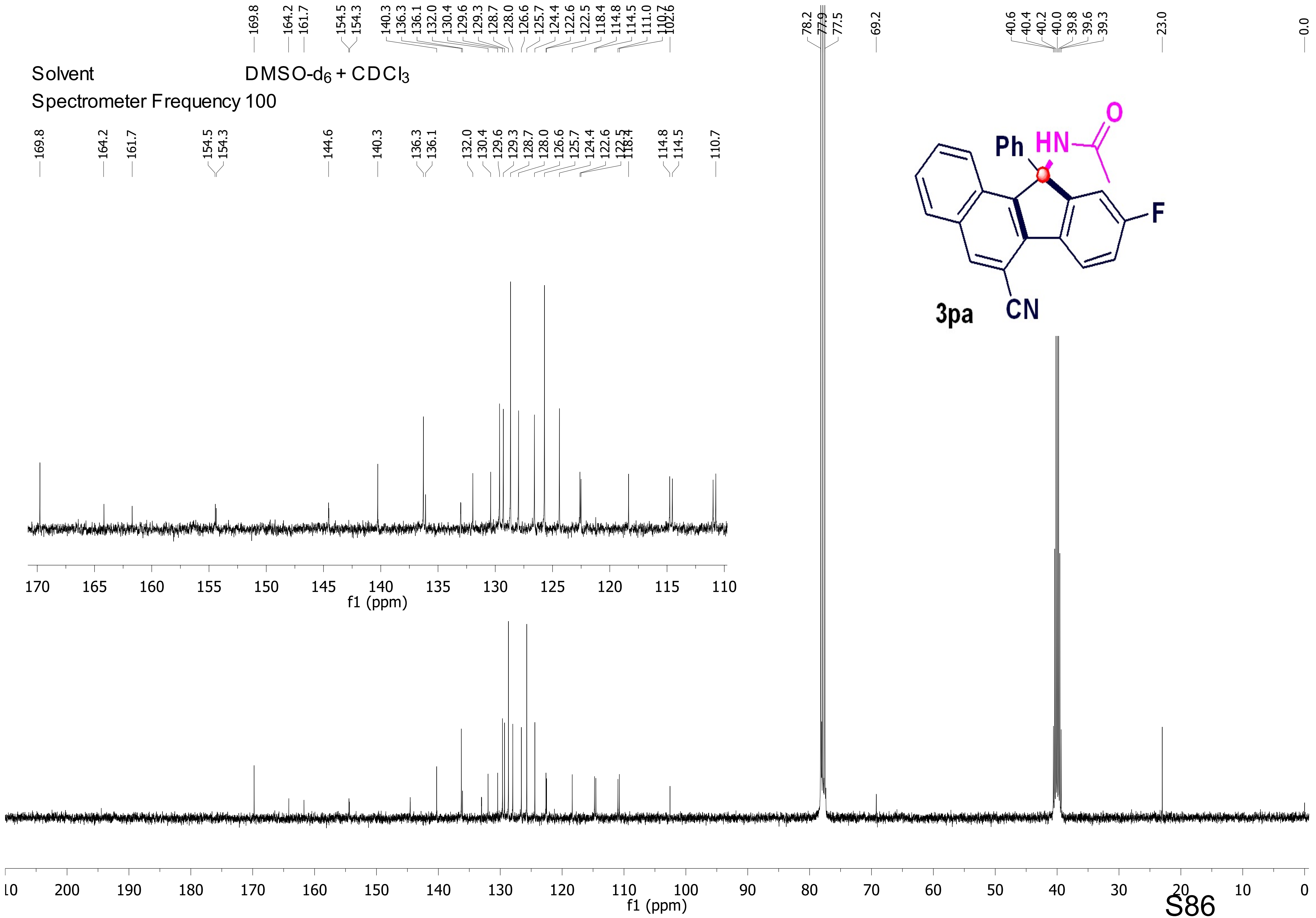
Solvent DMSO-d_6 + $CDCl_3$
Spectrometer Frequency 100
169.8 164.2 161.7 154.5 154.3 140.3 136.3 136.1 132.0 130.4 129.6 129.3 128.7 128.0 126.6 125.7 124.4 122.6 122.5 118.4 114.8 114.5 111.0 110.7 102.6
78.2 77.9 77.5 69.2
40.6 40.4 40.2 40.0 39.8 39.6 39.3 23.0 0.0
169.8 164.2 161.7 154.5 154.3 144.6 140.3 136.3 136.1 132.0 130.4 129.6 129.3 128.7 128.0 126.6 125.7 124.4 122.6 122.5 118.4 114.8 114.5 110.7
Ph
HN
O
F
CN
3pa
f1 (ppm)
f1 (ppm)

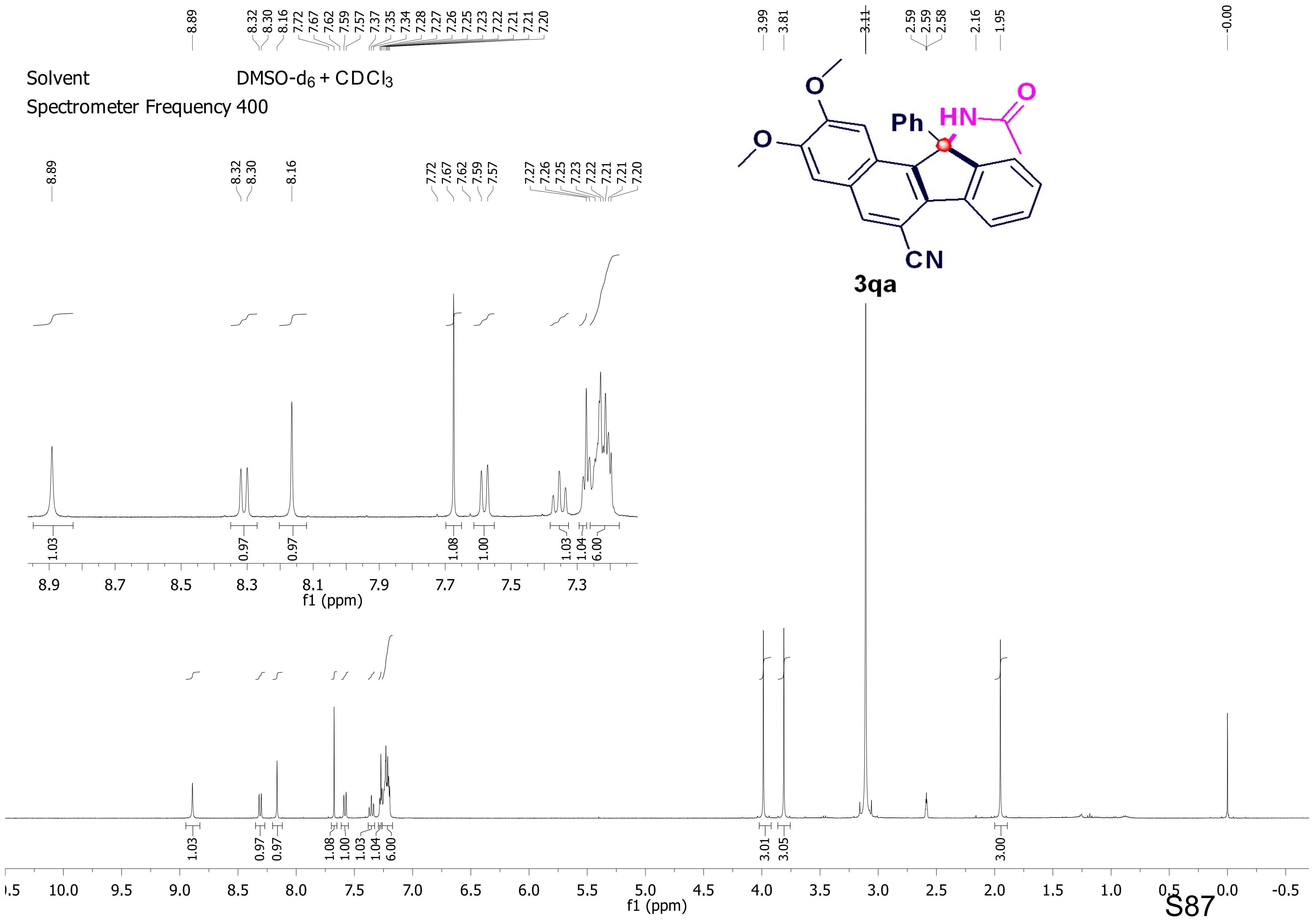
Solvent DMSO-d6 + CDCl3
Spectrometer Frequency 400
8.89
8.32
8.30
8.16
7.72
7.67
7.62
7.59
7.57
7.37
7.35
7.34
7.28
7.27
7.26
7.25
7.23
7.22
7.21
7.21
7.20
3.99
3.81
3.11
2.59
2.59
2.58
2.16
1.95
-0.00
O
O
Ph
HN
O
CN
3qa
1.03
0.97
0.97
1.08
1.00
1.03
1.04
6.00
3.01
3.05
3.00
f1 (ppm)

Solvent DMSO-d_6 + $CDCl_3$

Spectrometer Frequency 100

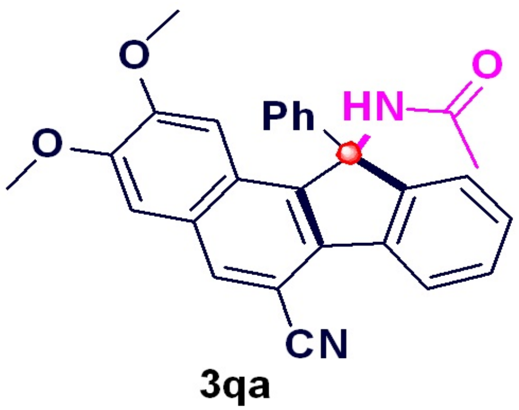

3qa

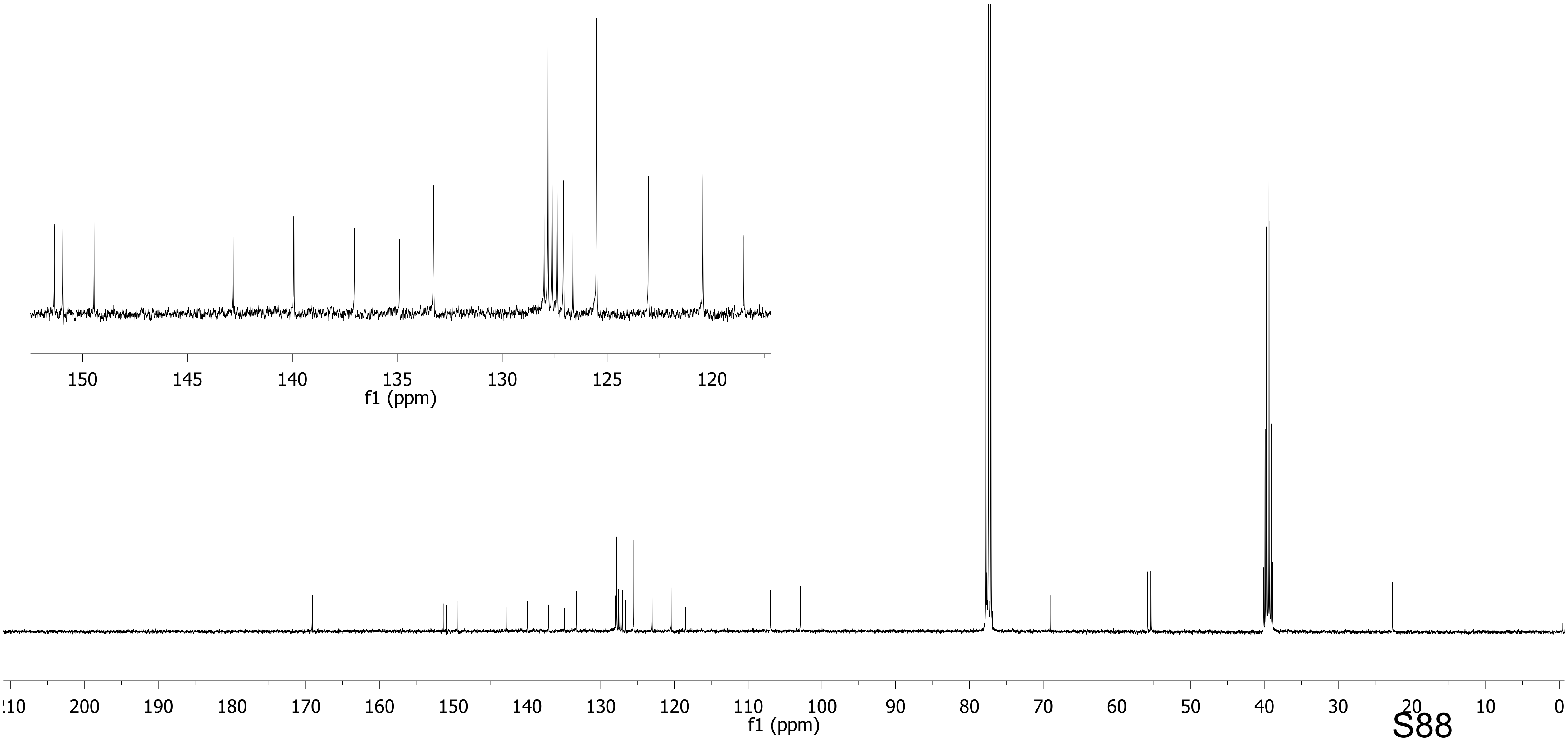

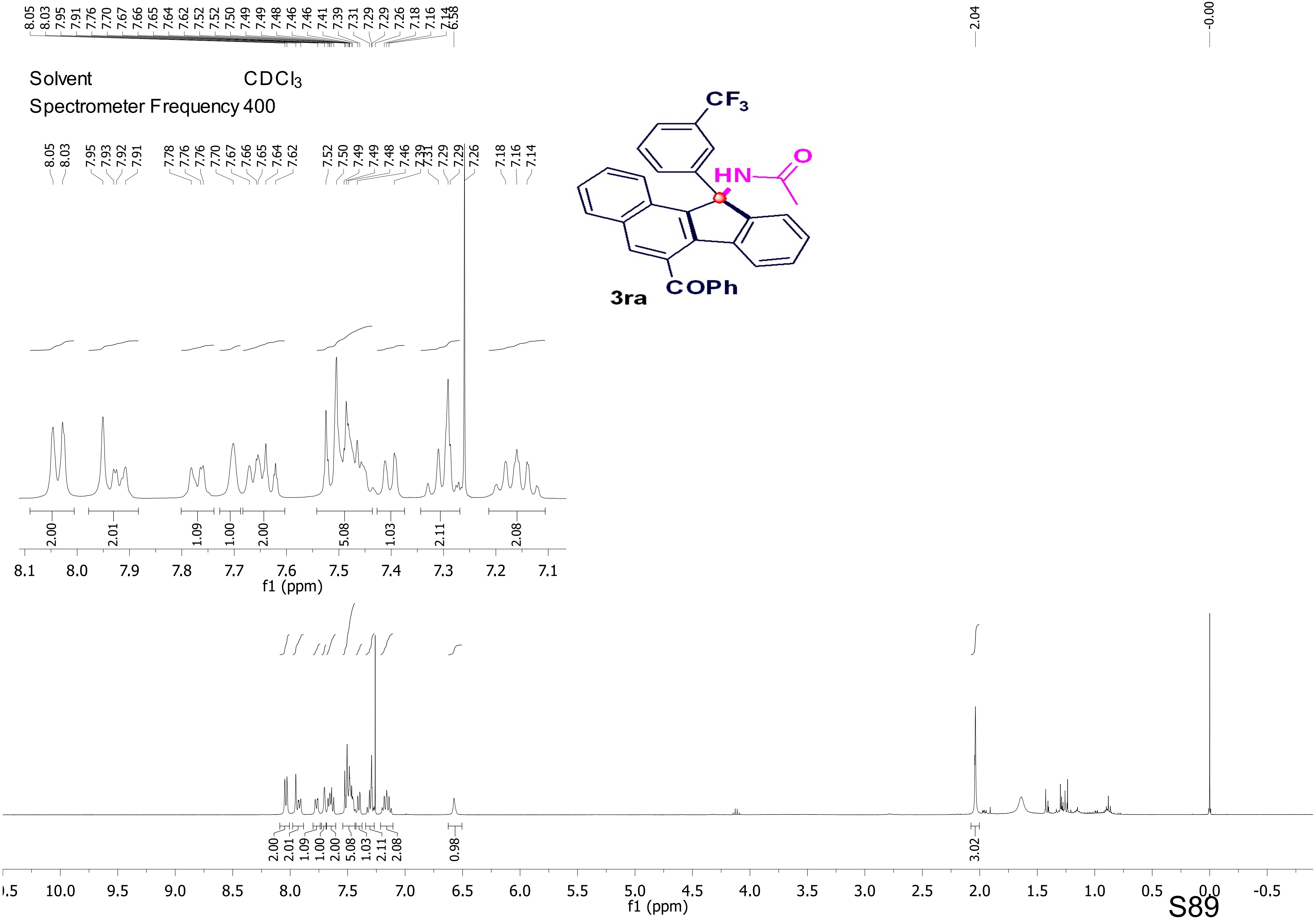
8.05
8.03
7.95
7.91
7.76
7.70
7.67
7.66
7.65
7.64
7.62
7.52
7.52
7.50
7.49
7.49
7.48
7.46
7.46
7.41
7.39
7.31
7.29
7.29
7.26
7.18
7.16
7.14
6.58
2.04
-0.00
Solvent CDCl3
Spectrometer Frequency 400
CF3
HN
O
COPh
3ra
2.00
2.01
1.09
1.00
2.00
5.08
1.03
2.11
2.08
0.98
3.02
f1 (ppm)

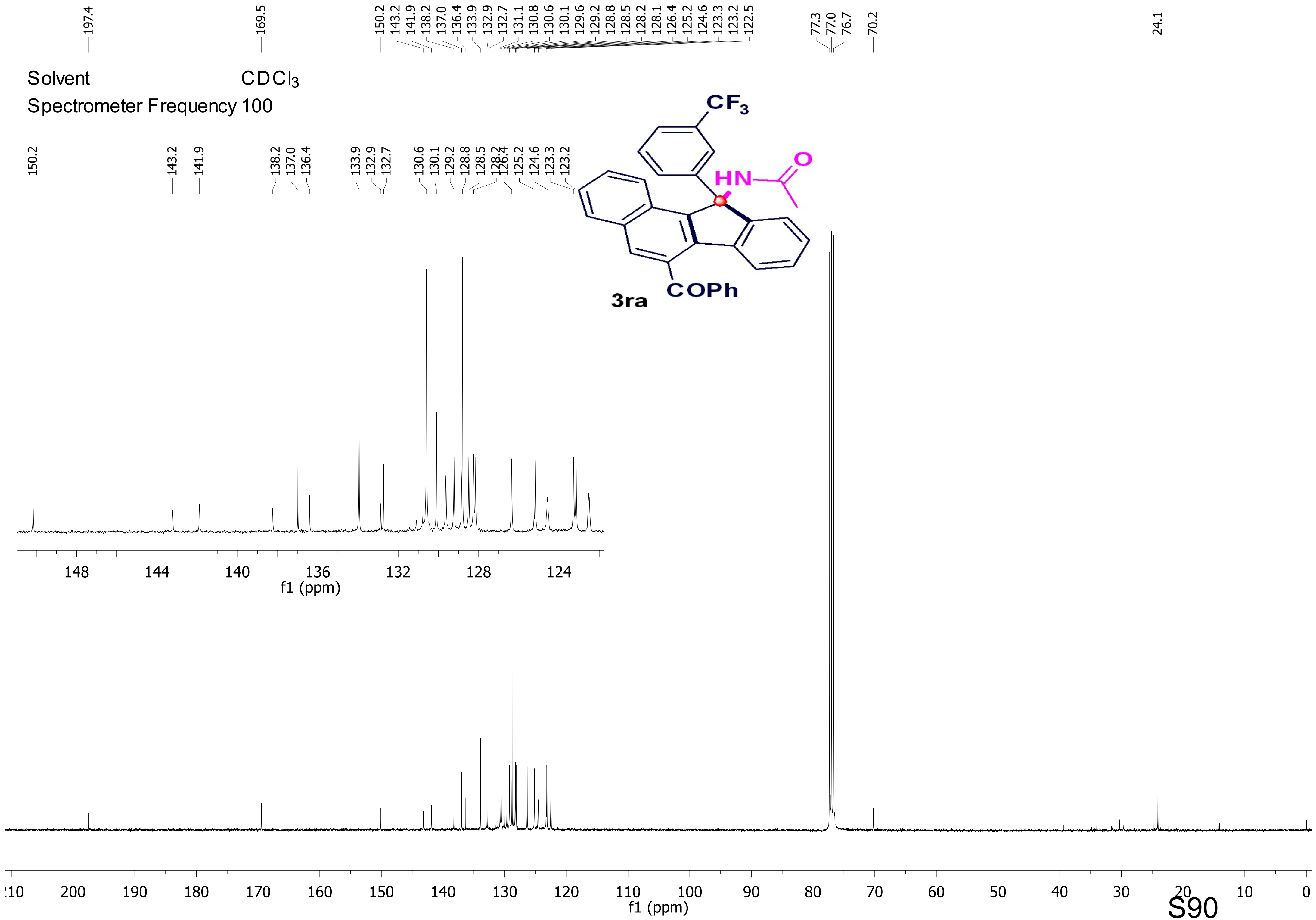
197.4
169.5
150.2
143.2
141.9
138.2
137.0
136.4
133.9
132.9
132.7
131.1
130.8
130.6
130.1
129.6
129.2
128.8
128.5
128.2
128.1
126.4
125.2
124.6
123.3
123.2
122.5
77.3
77.0
76.7
70.2
24.1
Solvent CDCl3
Spectrometer Frequency 100
CF3
HN
O
COPh
3ra
f1 (ppm)
f1 (ppm)

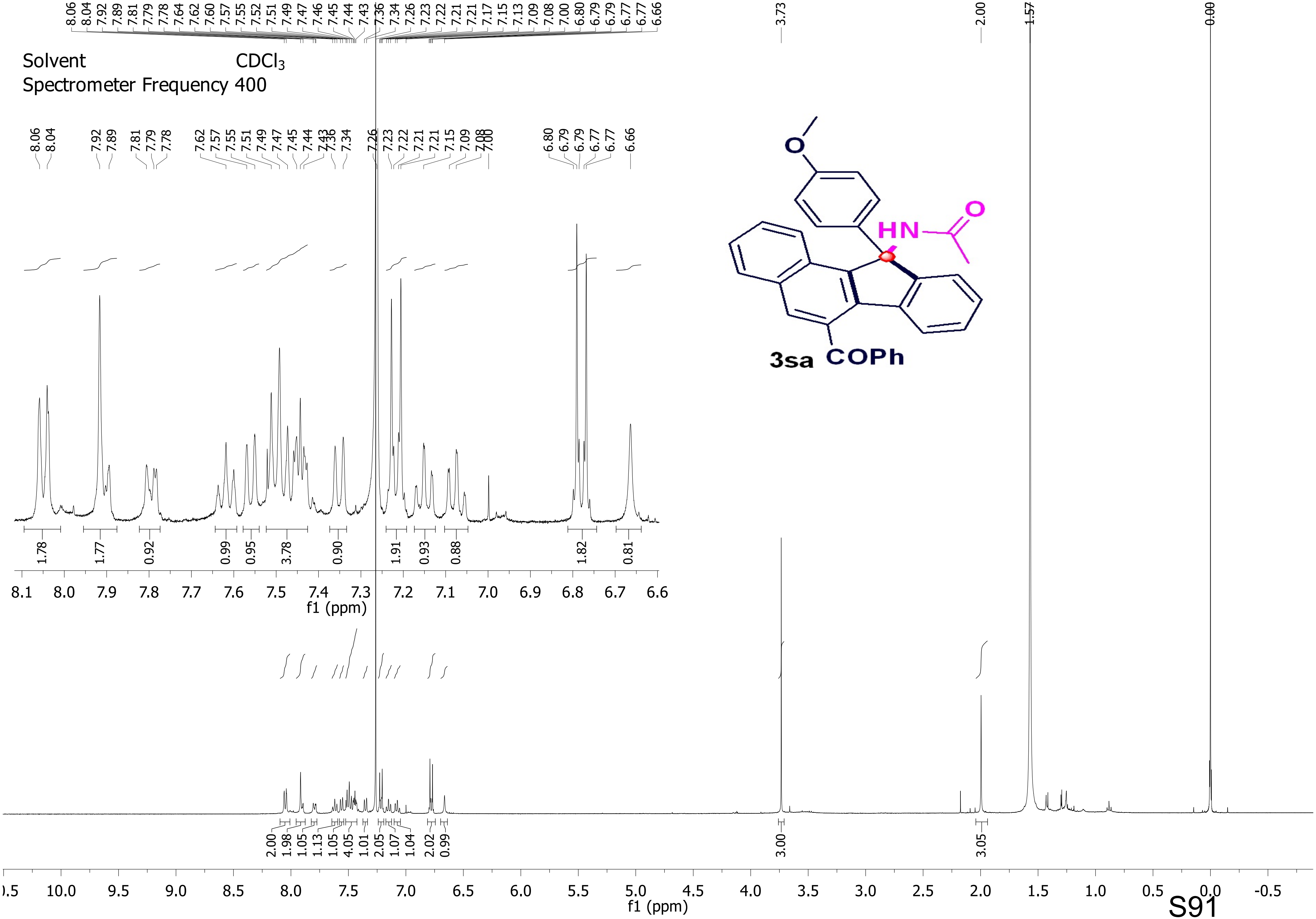
Solvent CDCl$_3$
Spectrometer Frequency 400
3sa
COPh
HN
O
f1 (ppm)
f1 (ppm)

Solvent CDCl$_3$

Spectrometer Frequency 150

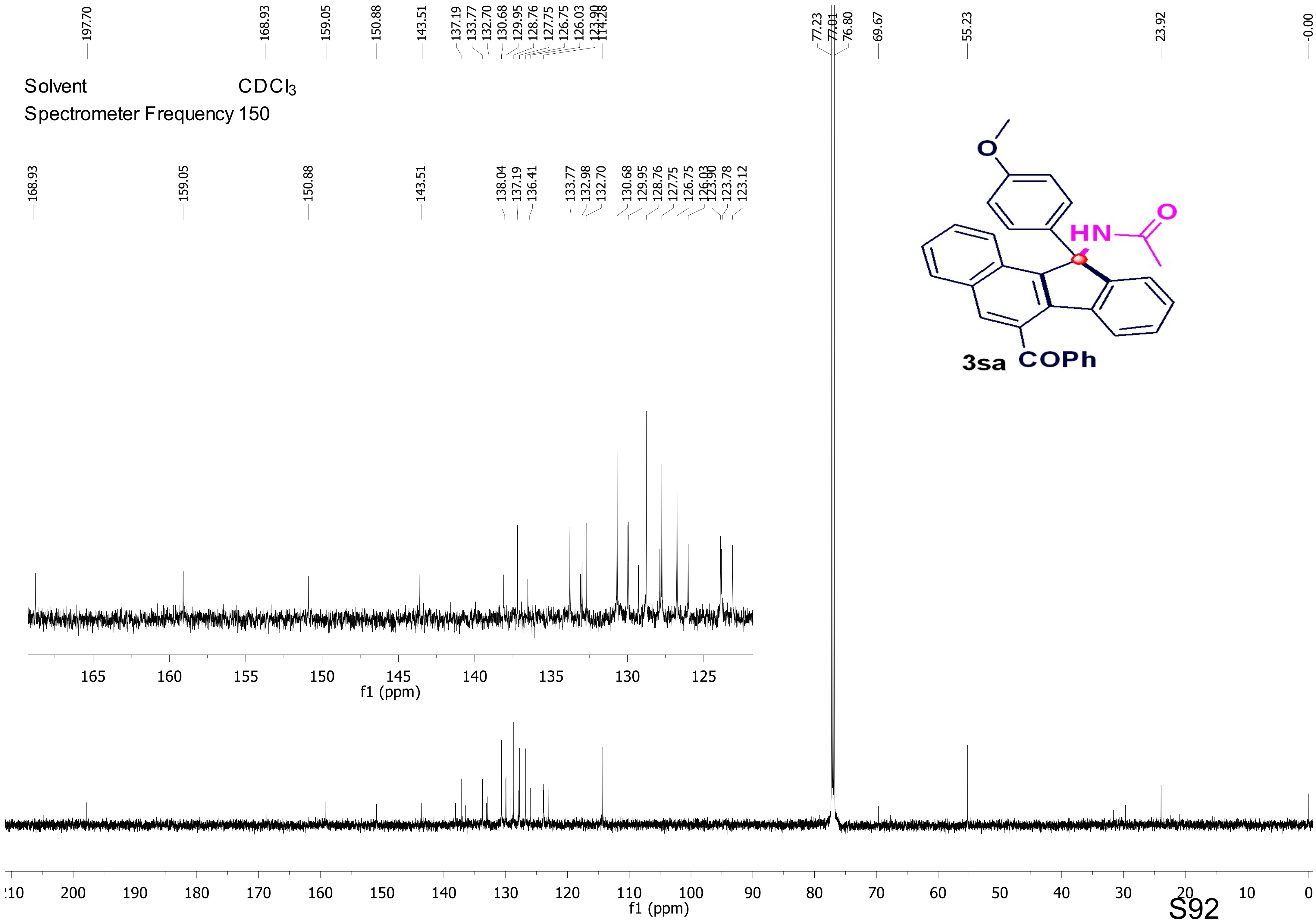

3sa

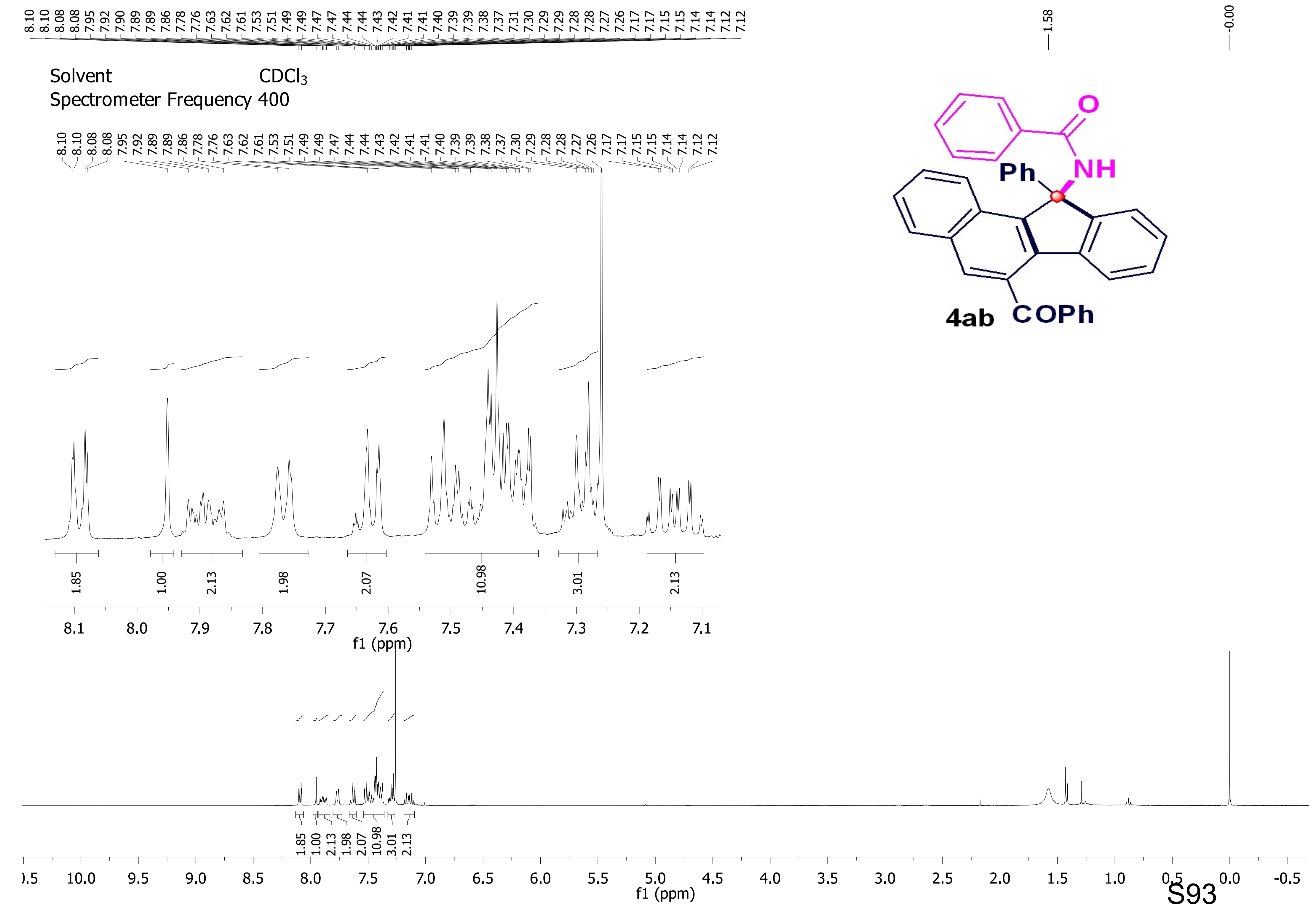
Solvent CDCl$_3$
Spectrometer Frequency 400
8.10 8.10 8.08 8.08 7.95 7.92 7.90 7.89 7.89 7.86 7.78 7.76 7.63 7.62 7.61 7.53 7.51 7.49 7.49 7.47 7.47 7.44 7.44 7.43 7.42 7.41 7.41 7.40 7.39 7.39 7.38 7.37 7.31 7.30 7.29 7.29 7.28 7.28 7.27 7.26 7.17 7.17 7.15 7.15 7.14 7.14 7.12 7.12
1.58
-0.00
O
NH
Ph
4ab
COPh
1.85
1.00
2.13
1.98
2.07
10.98
3.01
2.13
f1 (ppm)

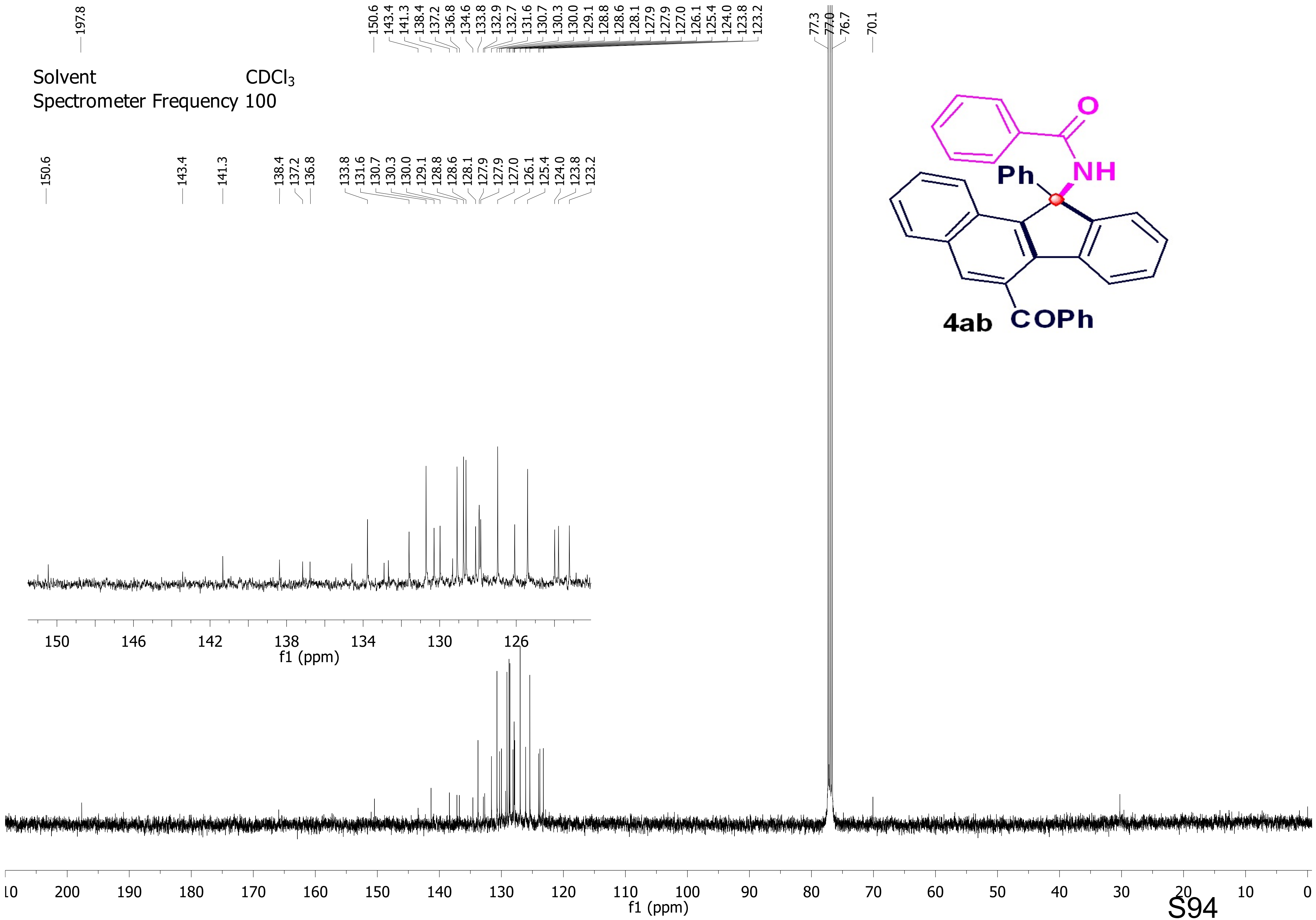
197.8
150.6 143.4 141.3 138.4 137.2 136.8 134.6 133.8 132.9 132.7 131.6 130.7 130.3 130.0 129.1 128.8 128.6 128.1 127.9 127.9 127.0 126.1 125.4 124.0 123.8 123.2
77.3 77.0 76.7
70.1
Solvent CDCl3
Spectrometer Frequency 100
150.6 143.4 141.3 138.4 137.2 136.8 133.8 131.6 130.7 130.3 130.0 129.1 128.8 128.6 128.1 127.9 127.9 127.0 126.1 125.4 124.0 123.8 123.2
150 146 142 138 134 130 126
f1 (ppm)
O
NH
Ph
4ab
COPh
200 190 180 170 160 150 140 130 120 110 100 90 80 70 60 50 40 30 20 10 0
f1 (ppm)

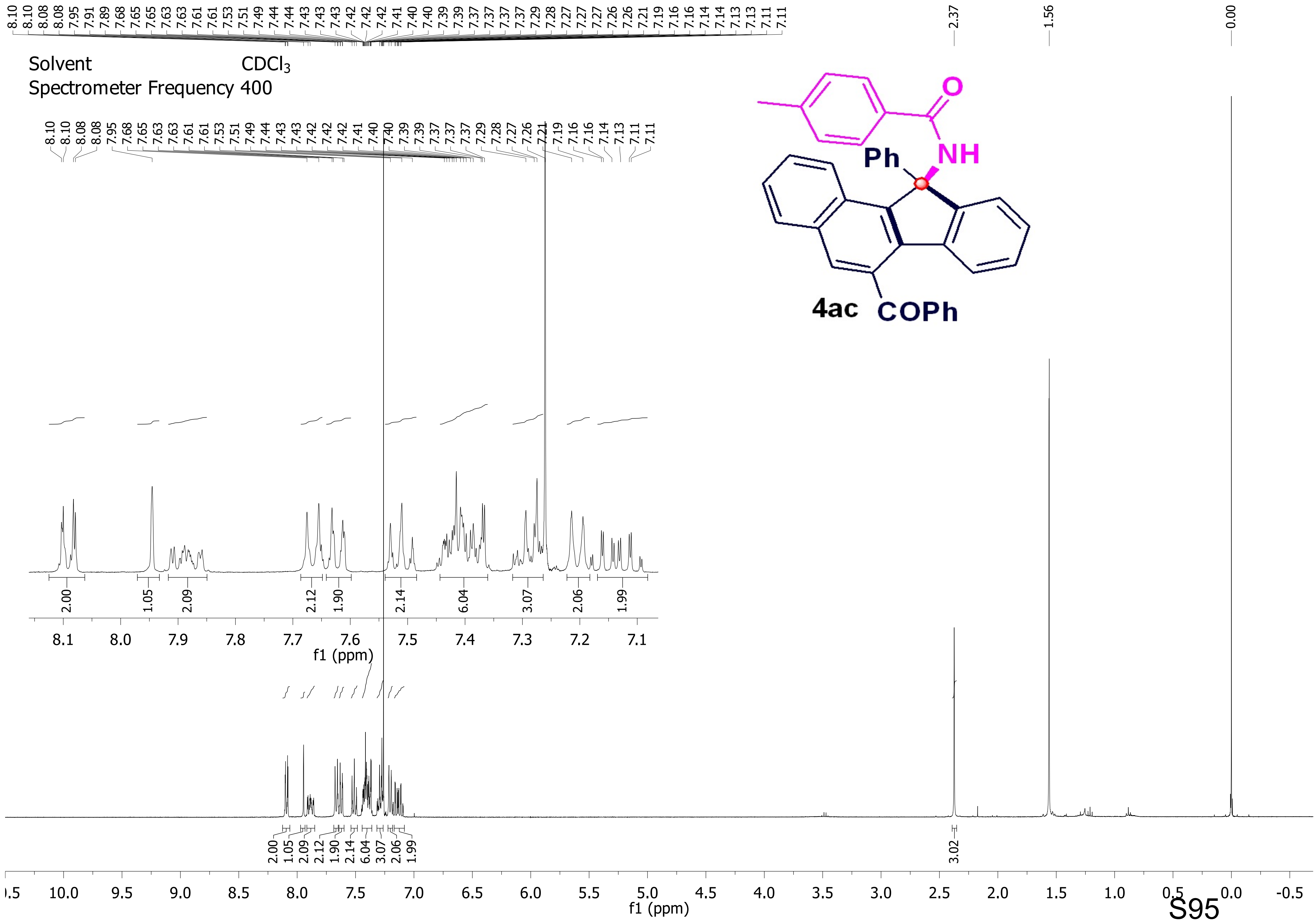

Solvent CDCl3
Spectrometer Frequency 400
4ac
Ph
NH
O
COPh
f1 (ppm)

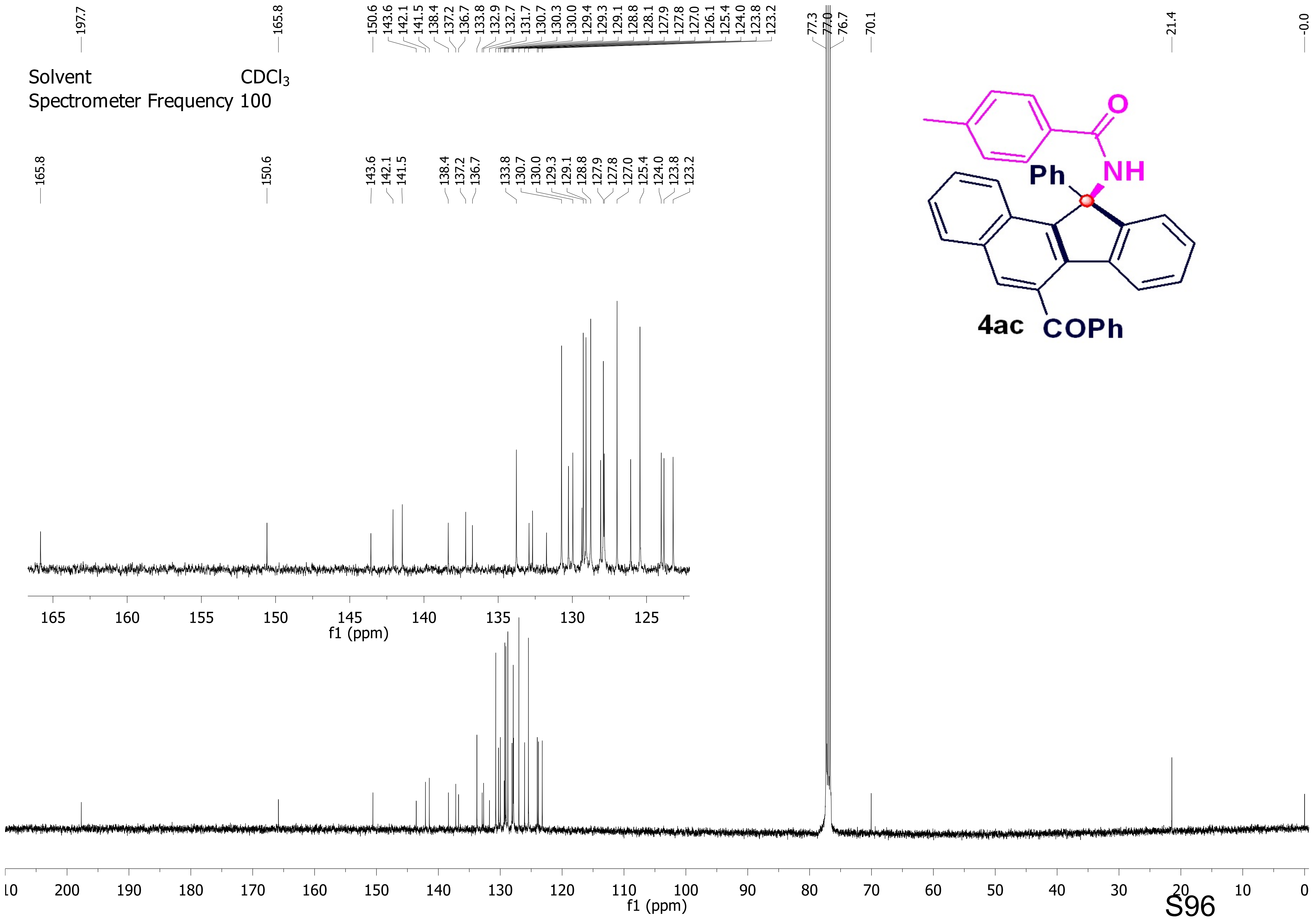
197.7
165.8
150.6
143.6
142.1
141.5
138.4
137.2
136.7
133.8
132.9
132.7
131.7
130.7
130.3
130.0
129.4
129.3
129.1
128.8
128.1
127.9
127.8
127.0
126.1
125.4
124.0
123.8
123.2
77.3
77.0
76.7
70.1
21.4
-0.0
Solvent CDCl3
Spectrometer Frequency 100
4ac
Ph
NH
O
COPh
f1 (ppm)
f1 (ppm)

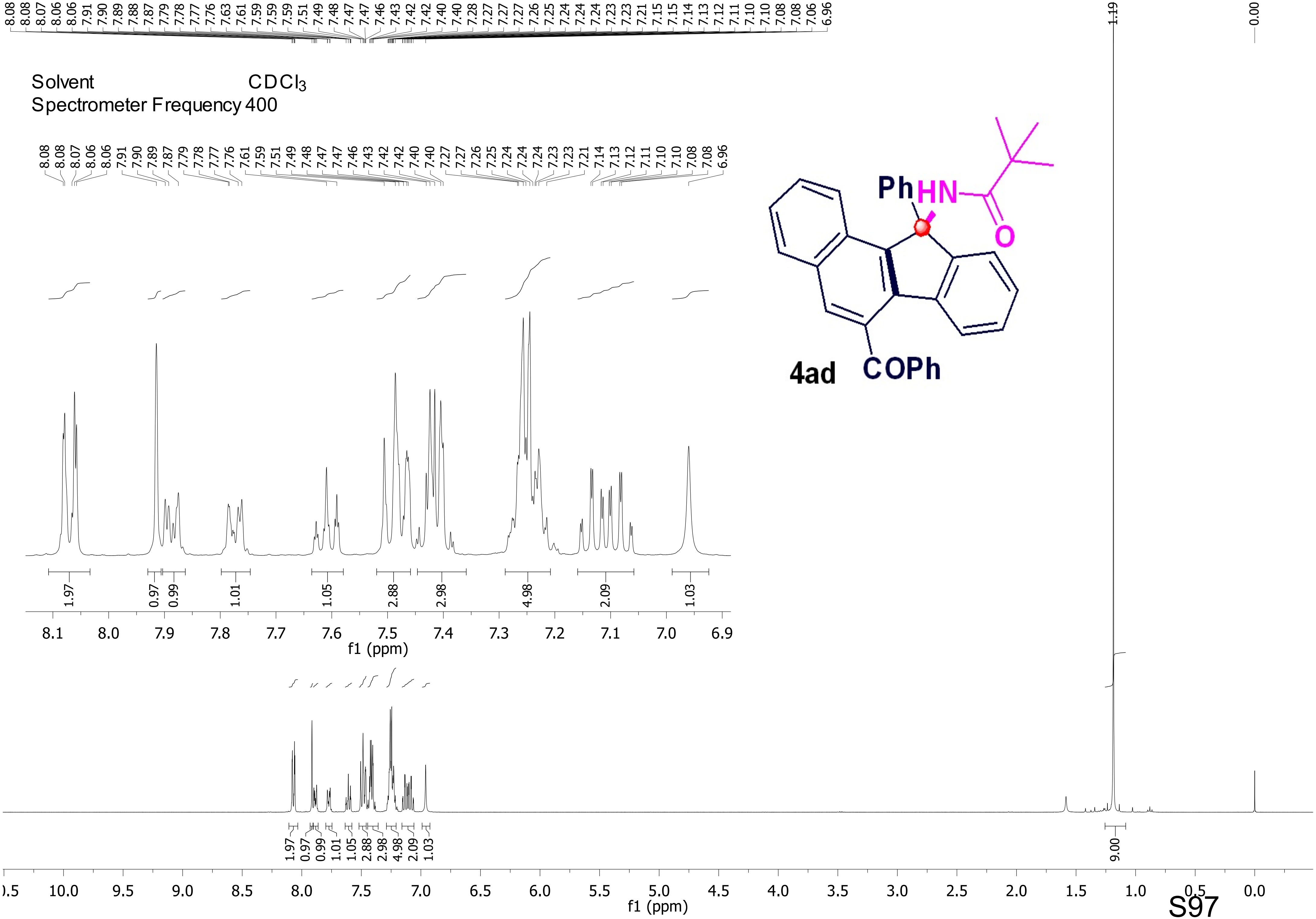
Solvent CDCl3
Spectrometer Frequency 400
Ph
HN
O
4ad
COPh
8.08 8.08 8.07 8.06 8.06 7.91 7.90 7.89 7.88 7.87 7.79 7.78 7.77 7.76 7.63 7.61 7.59 7.59 7.59 7.51 7.49 7.48 7.47 7.47 7.46 7.43 7.42 7.42 7.40 7.40 7.28 7.27 7.27 7.27 7.26 7.25 7.24 7.24 7.24 7.23 7.23 7.21 7.15 7.15 7.14 7.13 7.12 7.11 7.10 7.10 7.08 7.08 7.06 6.96
1.19
0.00
1.97 0.97 0.99 1.01 1.05 2.88 2.98 4.98 2.09 1.03
9.00
f1 (ppm)

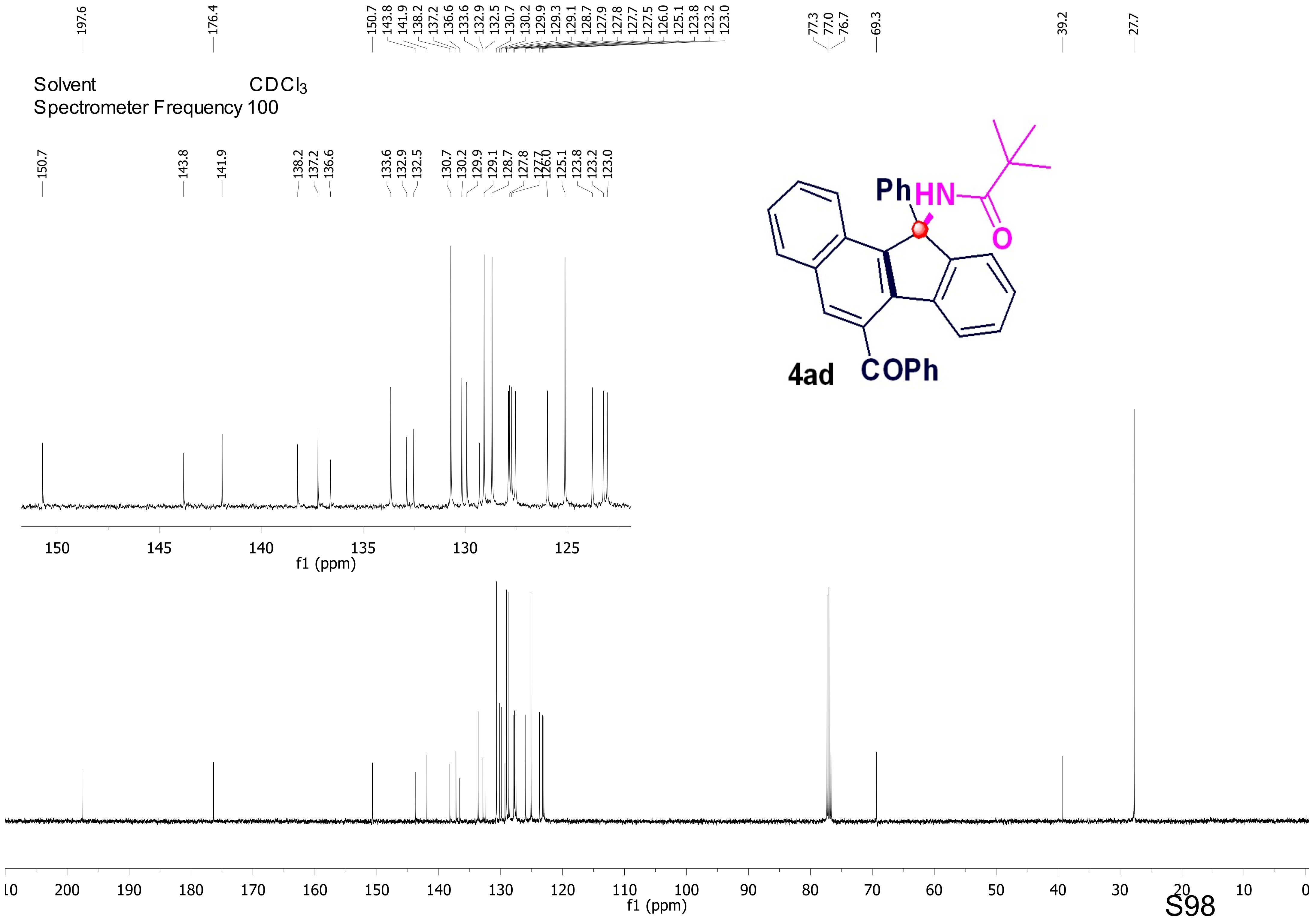

Solvent CDCl3
Spectrometer Frequency 100
197.6
176.4
150.7
143.8
141.9
138.2
137.2
136.6
133.6
132.9
132.5
130.7
130.2
129.9
129.3
129.1
128.7
127.9
127.8
127.7
127.5
126.0
125.1
123.8
123.2
123.0
77.3
77.0
76.7
69.3
39.2
27.7
Ph
HN
O
4ad
COPh
f1 (ppm)

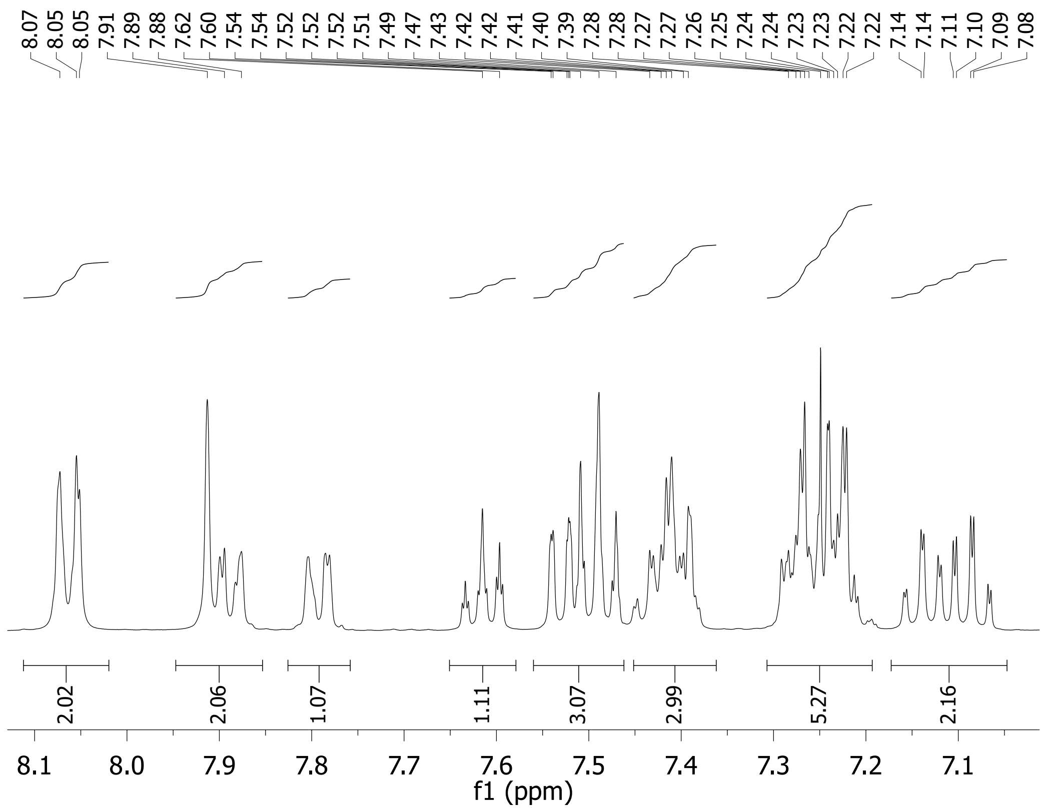

Solvent CDCl$_3$
Spectrometer Frequency 400

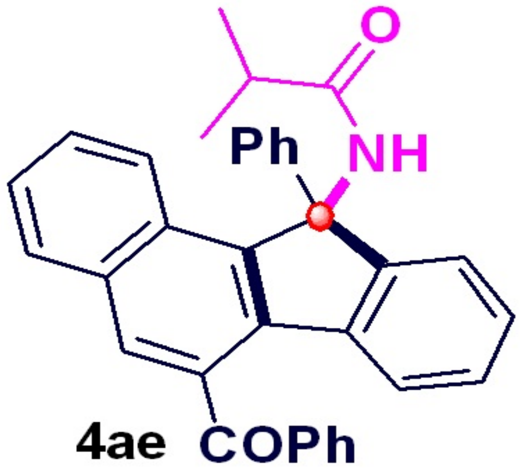

4ae

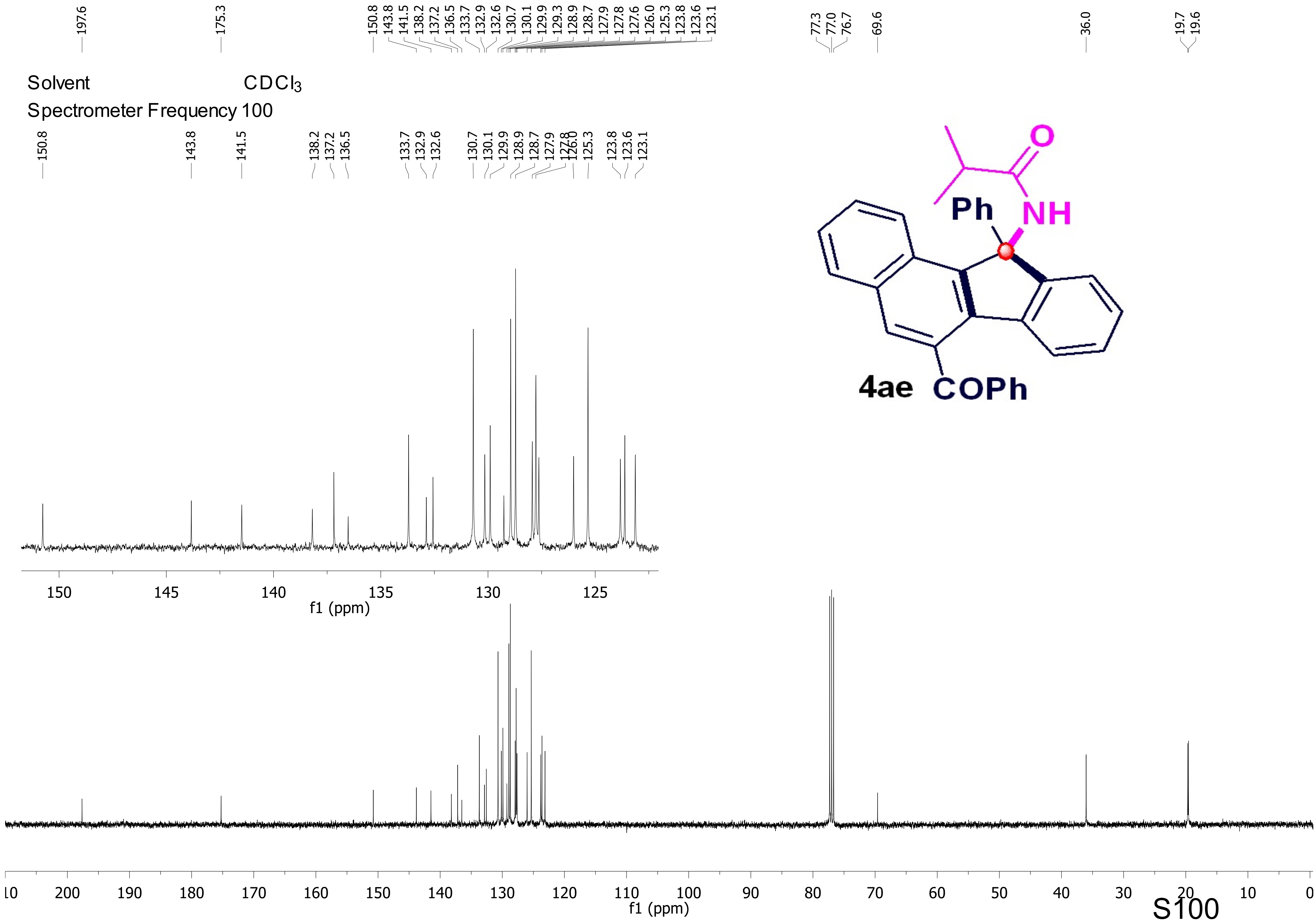
197.6
175.3
150.8 143.8 141.5 138.2 137.2 136.5 133.7 132.9 132.6 130.7 130.1 129.9 129.3 128.9 128.7 127.9 127.8 127.6 126.0 125.3 123.8 123.6 123.1
77.3 77.0 76.7
69.6
36.0
19.7 19.6
Solvent CDCl3
Spectrometer Frequency 100
150.8 143.8 141.5 138.2 137.2 136.5 133.7 132.9 132.6 130.7 130.1 129.9 128.9 128.7 127.9 127.8 126.0 125.3 123.8 123.6 123.1
O
NH
Ph
COPh
4ae
f1 (ppm)
f1 (ppm)

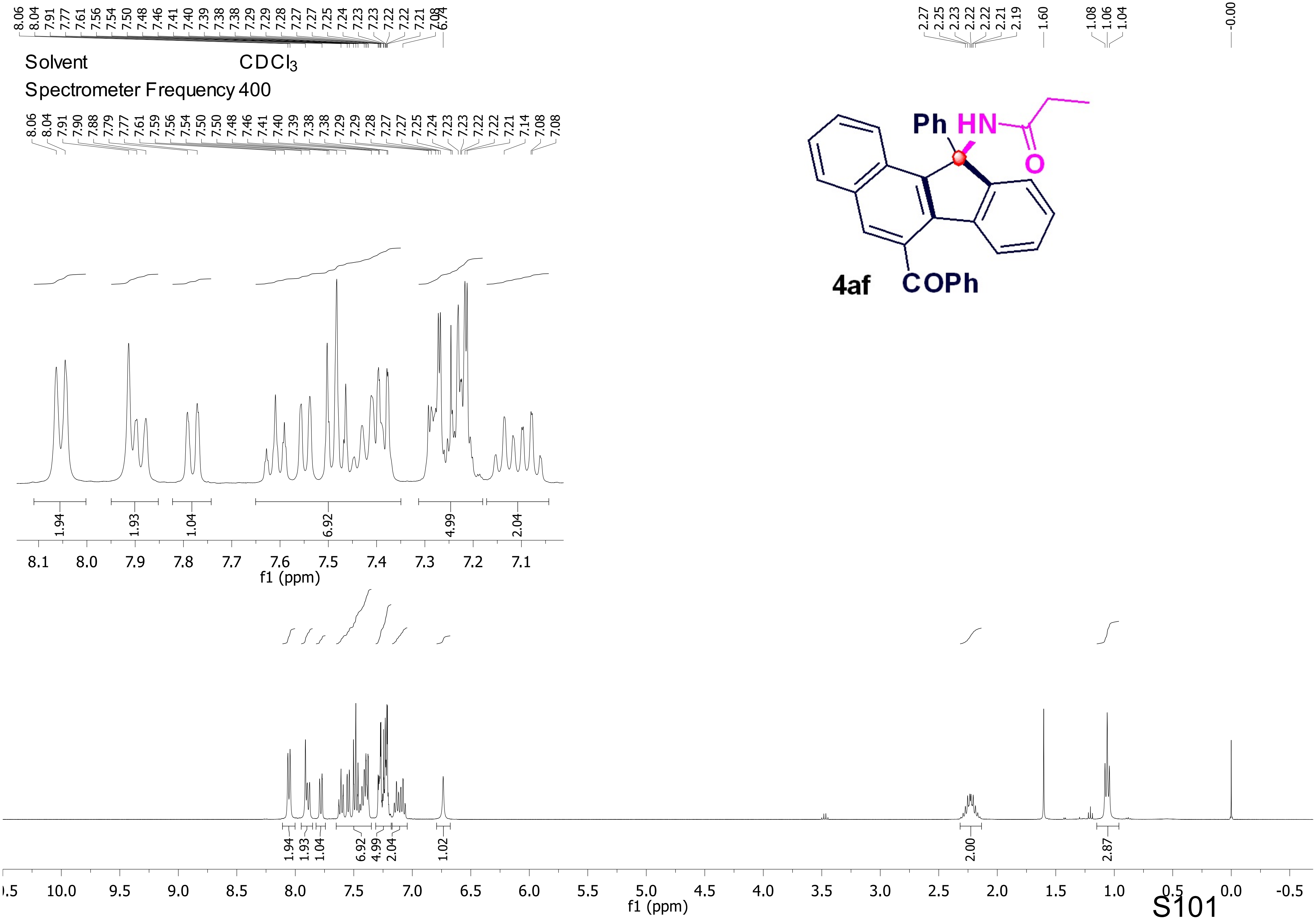
Solvent CDCl3
Spectrometer Frequency 400
Ph HN
O
4af
COPh
1.94
1.93
1.04
6.92
4.99
2.04
1.02
2.00
2.87
f1 (ppm)

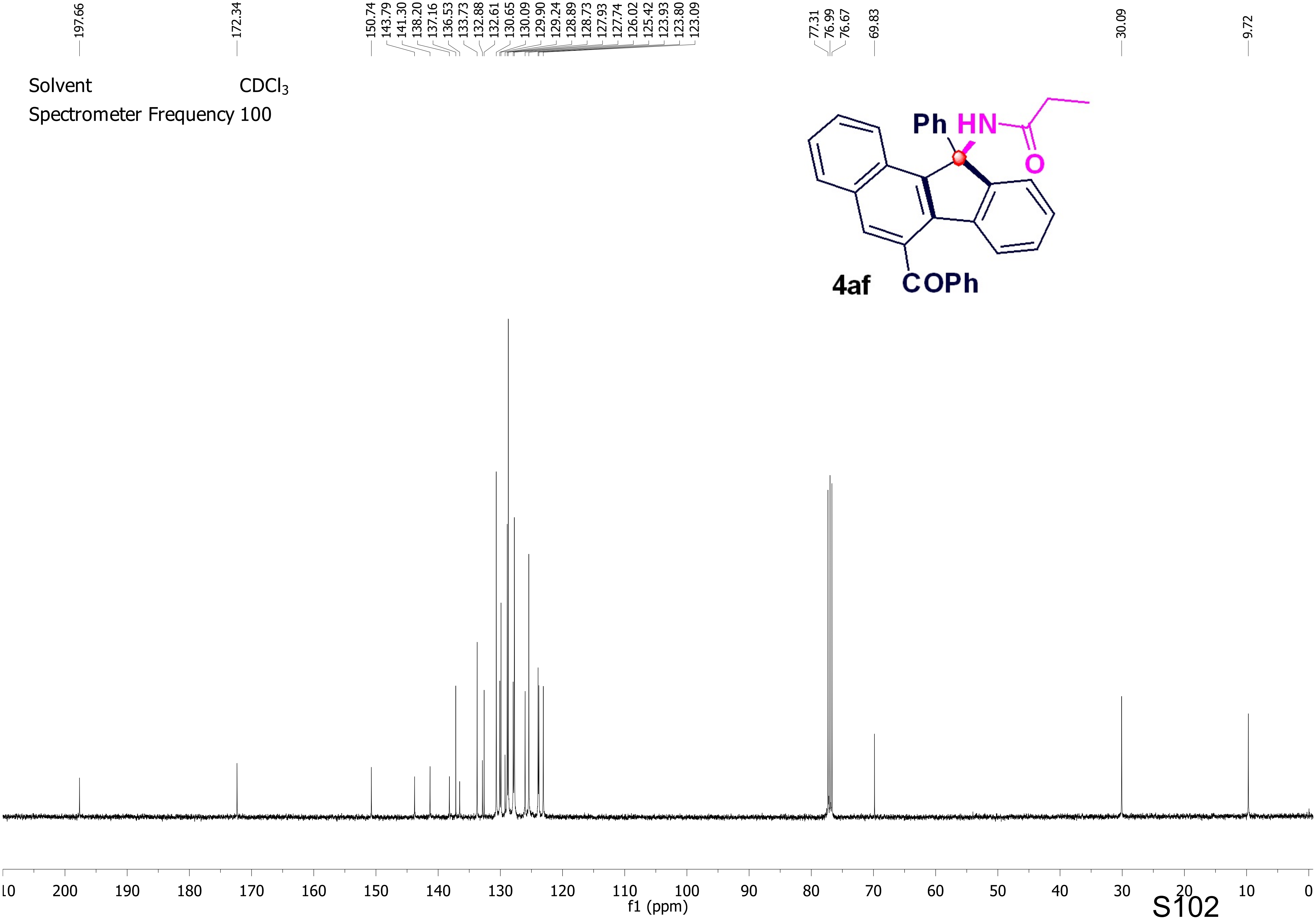
197.66
172.34
150.74
143.79
141.30
138.20
137.16
136.53
133.73
132.88
132.61
130.65
130.09
129.90
129.24
128.89
128.73
127.93
127.74
126.02
125.42
123.93
123.80
123.09
77.31
76.99
76.67
69.83
30.09
9.72
Solvent CDCl$_3$
Spectrometer Frequency 100
Ph
HN
O
4af
COPh
f1 (ppm)

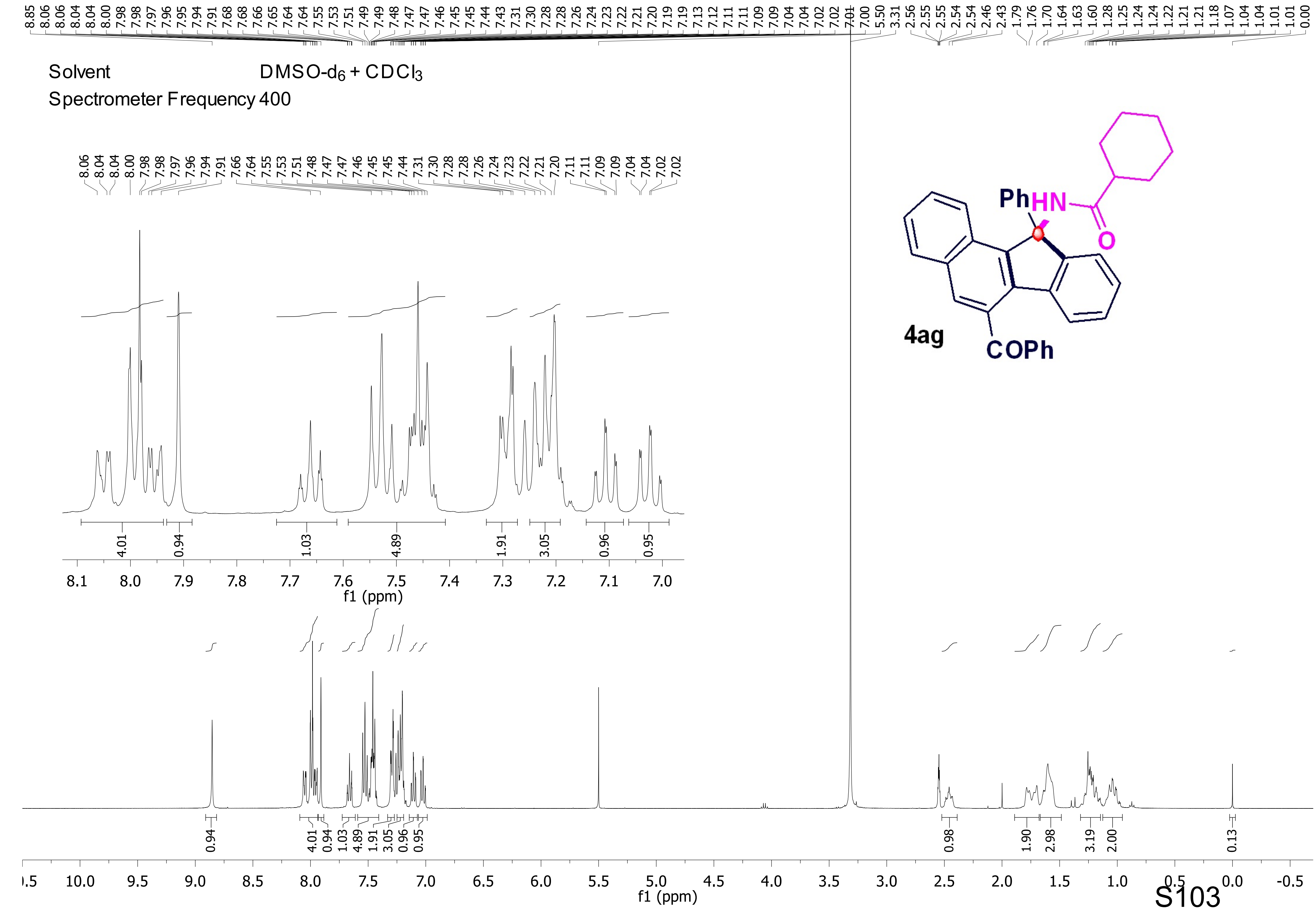
8.85 8.06 8.06 8.04 8.04 8.00 7.98 7.98 7.97 7.96 7.95 7.94 7.91 7.68 7.66 7.65 7.64 7.64 7.55 7.53 7.51 7.49 7.49 7.48 7.47 7.47 7.46 7.45 7.45 7.44 7.43 7.31 7.30 7.28 7.28 7.26 7.24 7.23 7.22 7.21 7.20 7.19 7.19 7.13 7.12 7.11 7.11 7.09 7.09 7.04 7.04 7.02 7.02 7.01 7.00 5.50 3.31 2.56 2.55 2.55 2.54 2.54 2.46 2.43 1.79 1.76 1.70 1.64 1.63 1.60 1.28 1.25 1.24 1.24 1.22 1.21 1.21 1.18 1.07 1.04 1.04 1.01 1.01 0.00
Solvent DMSO-d6 + CDCl3
Spectrometer Frequency 400
Ph
HN
O
4ag
COPh
4.01 0.94 1.03 4.89 1.91 3.05 0.96 0.95
f1 (ppm)
0.94 4.01 0.94 1.03 4.89 1.91 3.05 0.96 0.95 0.98 1.90 2.98 3.19 2.00 0.13
f1 (ppm)

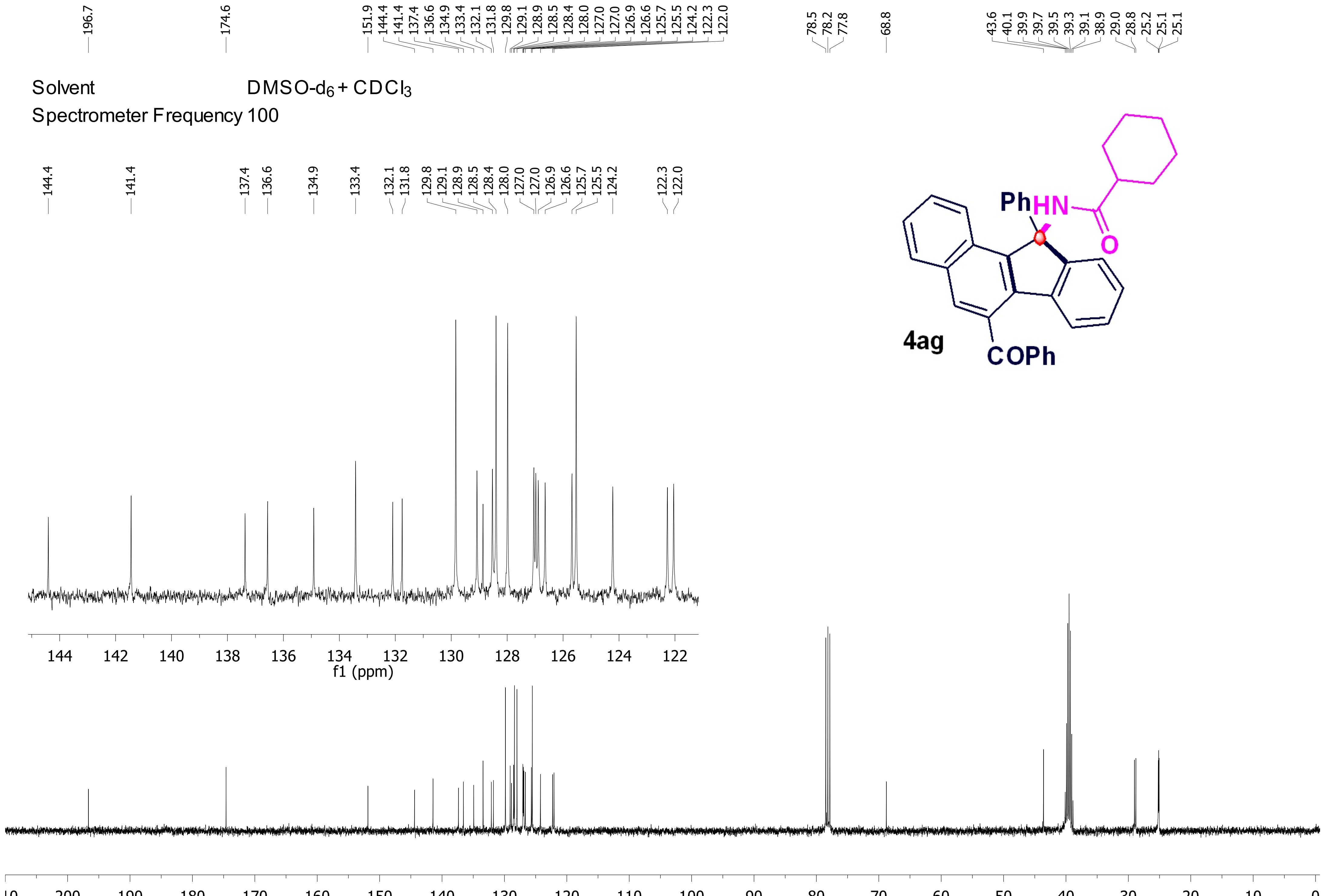
196.7
174.6
151.9
144.4
141.4
137.4
136.6
134.9
133.4
132.1
131.8
129.8
129.1
128.9
128.5
128.4
128.0
127.0
127.0
126.9
126.6
125.7
125.5
124.2
122.3
122.0
78.5
78.2
77.8
68.8
43.6
40.1
39.9
39.7
39.5
39.3
39.1
38.9
29.0
28.8
25.2
25.1
25.1
Solvent DMSO-d6 + CDCl3
Spectrometer Frequency 100
Ph
HN
O
COPh
4ag
f1 (ppm)
f1 (ppm)

Solvent CDCl$_3$
Spectrometer Frequency 400

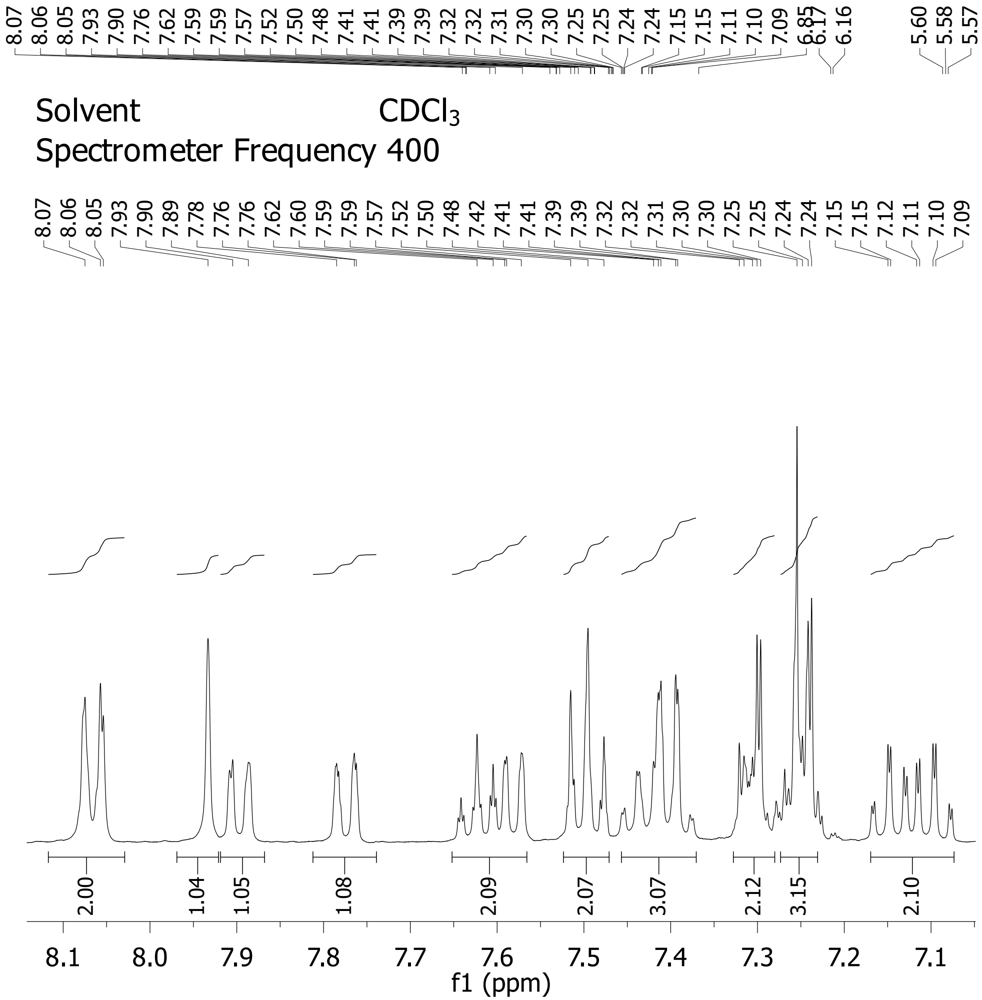

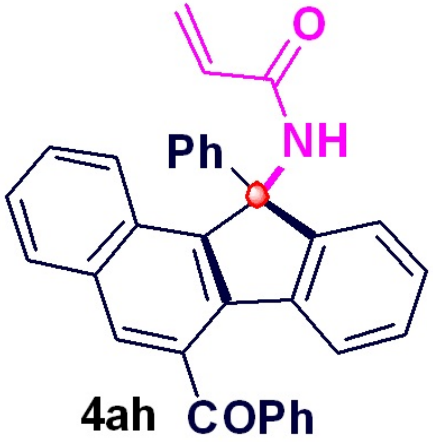

4ah

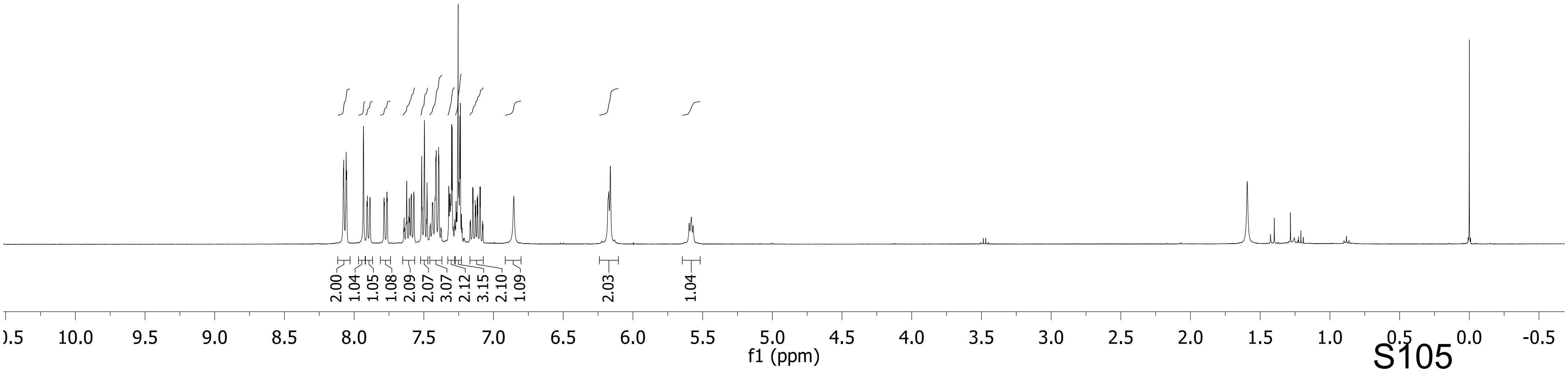

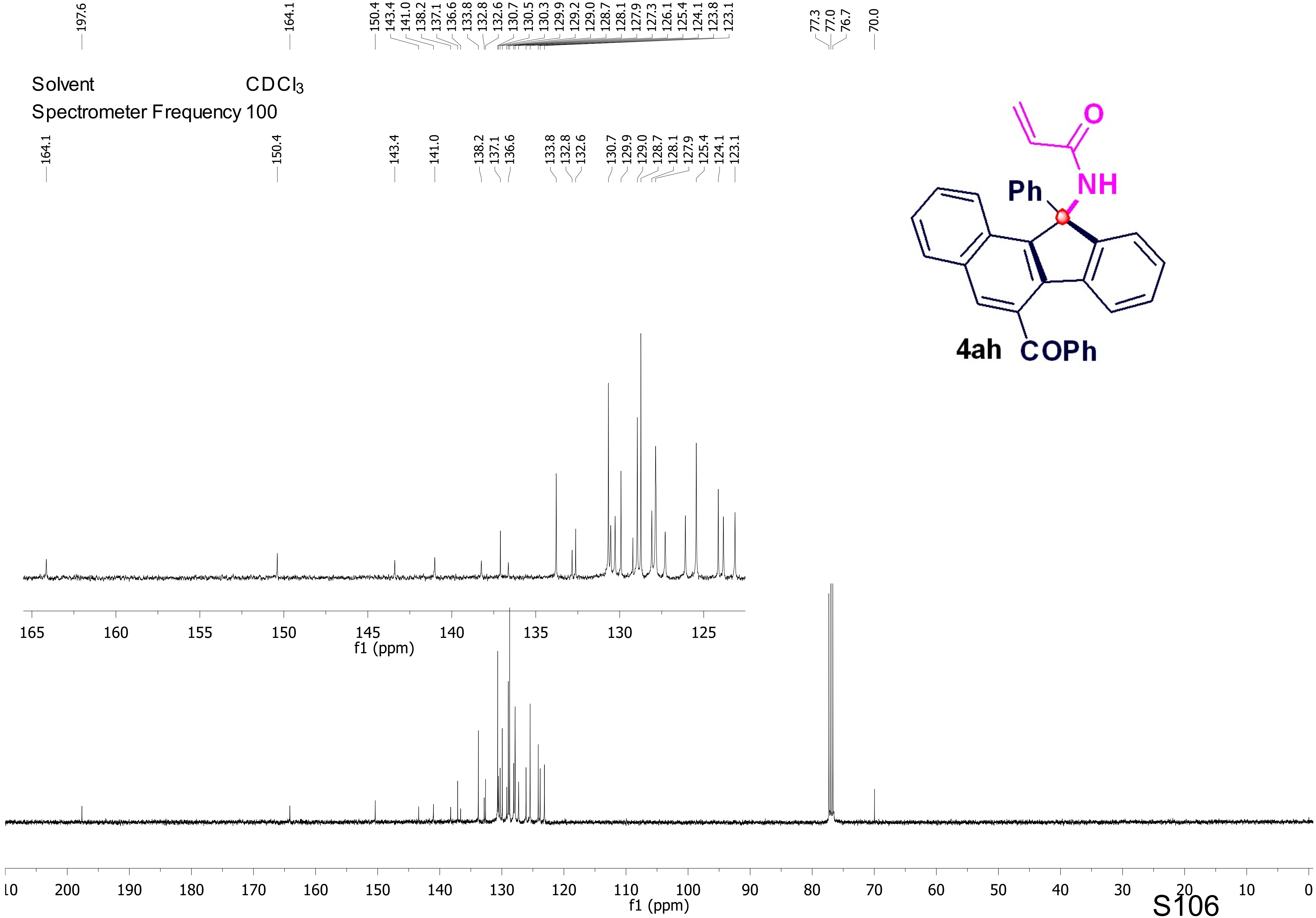
197.6
164.1
150.4 143.4 141.0 138.2 137.1 136.6 133.8 132.8 132.6 130.7 130.5 130.3 129.9 129.2 129.0 128.7 128.1 127.9 127.3 126.1 125.4 124.1 123.8 123.1
77.3 77.0 76.7
70.0
Solvent CDCl3
Spectrometer Frequency 100
164.1 150.4 143.4 141.0 138.2 137.1 136.6 133.8 132.8 132.6 130.7 129.9 129.0 128.7 128.1 127.9 125.4 124.1 123.1
165 160 155 150 145 140 135 130 125
f1 (ppm)
200 190 180 170 160 150 140 130 120 110 100 90 80 70 60 50 40 30 20 10 0
f1 (ppm)
O
NH
Ph
COPh

4ah

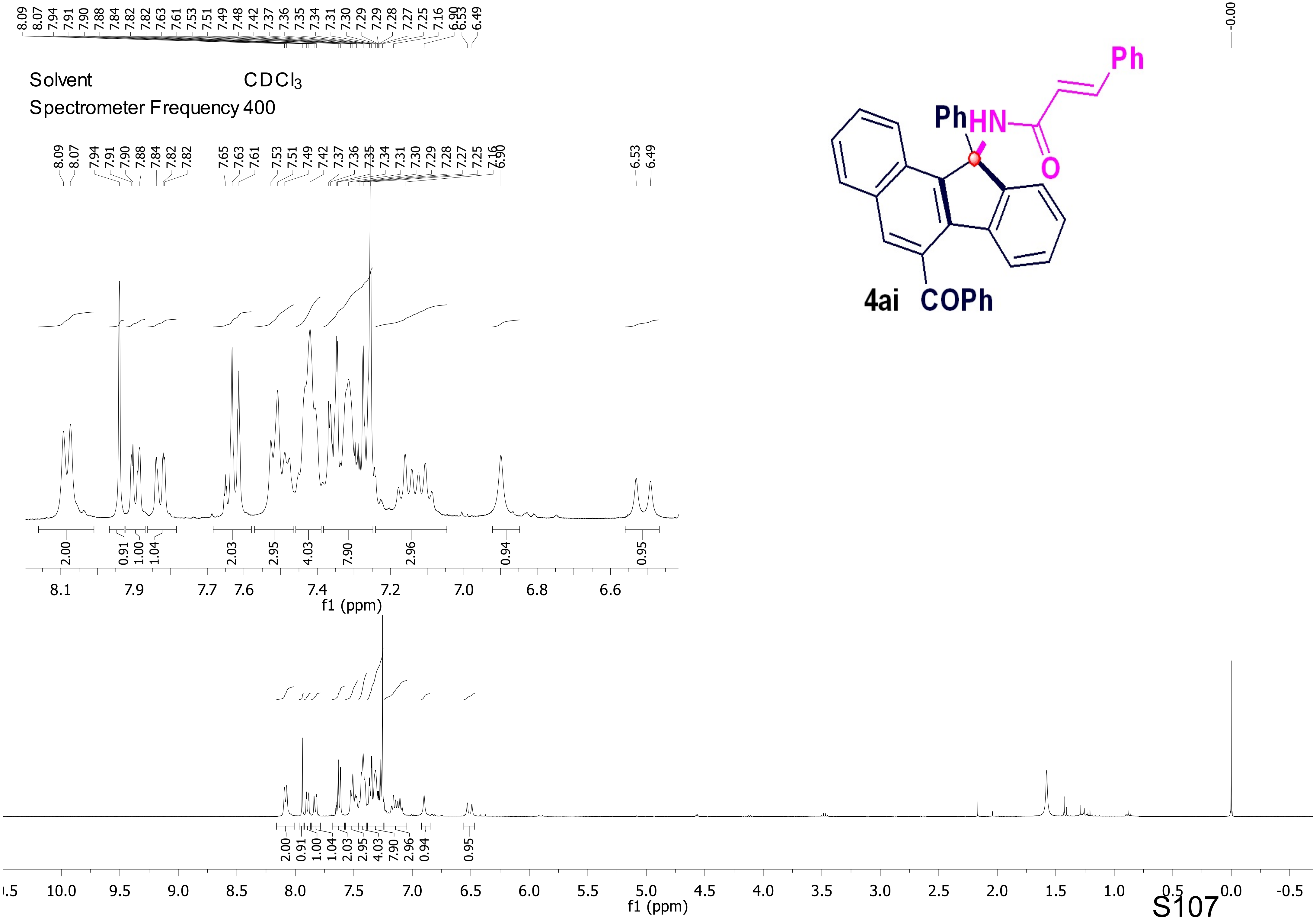
Solvent CDCl$_3$
Spectrometer Frequency 400
Ph
HN
O
Ph
4ai
COPh
f1 (ppm)

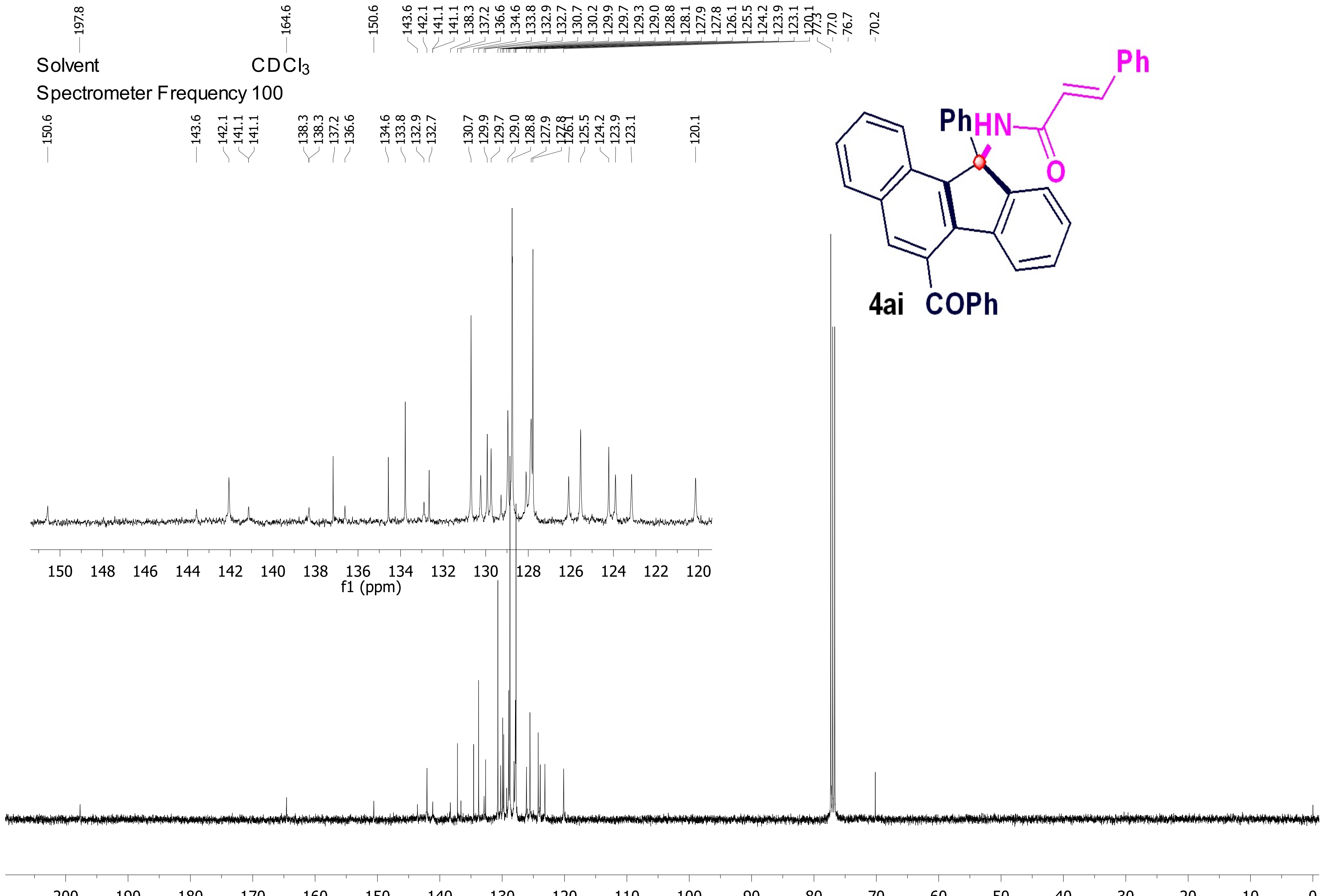
Solvent CDCl3
Spectrometer Frequency 100
197.8
164.6
150.6
143.6
142.1
141.1
141.1
138.3
137.2
136.6
134.6
133.8
132.9
132.7
130.7
130.2
129.9
129.7
129.3
129.0
128.8
128.1
127.9
127.8
126.1
125.5
124.2
123.9
123.1
120.1
77.3
77.0
76.7
70.2
150.6
143.6
142.1
141.1
141.1
138.3
138.3
137.2
136.6
134.6
133.8
132.9
132.7
130.7
129.9
129.7
129.0
128.8
127.9
127.8
126.1
125.5
124.2
123.9
123.1
120.1
Ph
HN
O
Ph
4ai
COPh
f1 (ppm)
f1 (ppm)

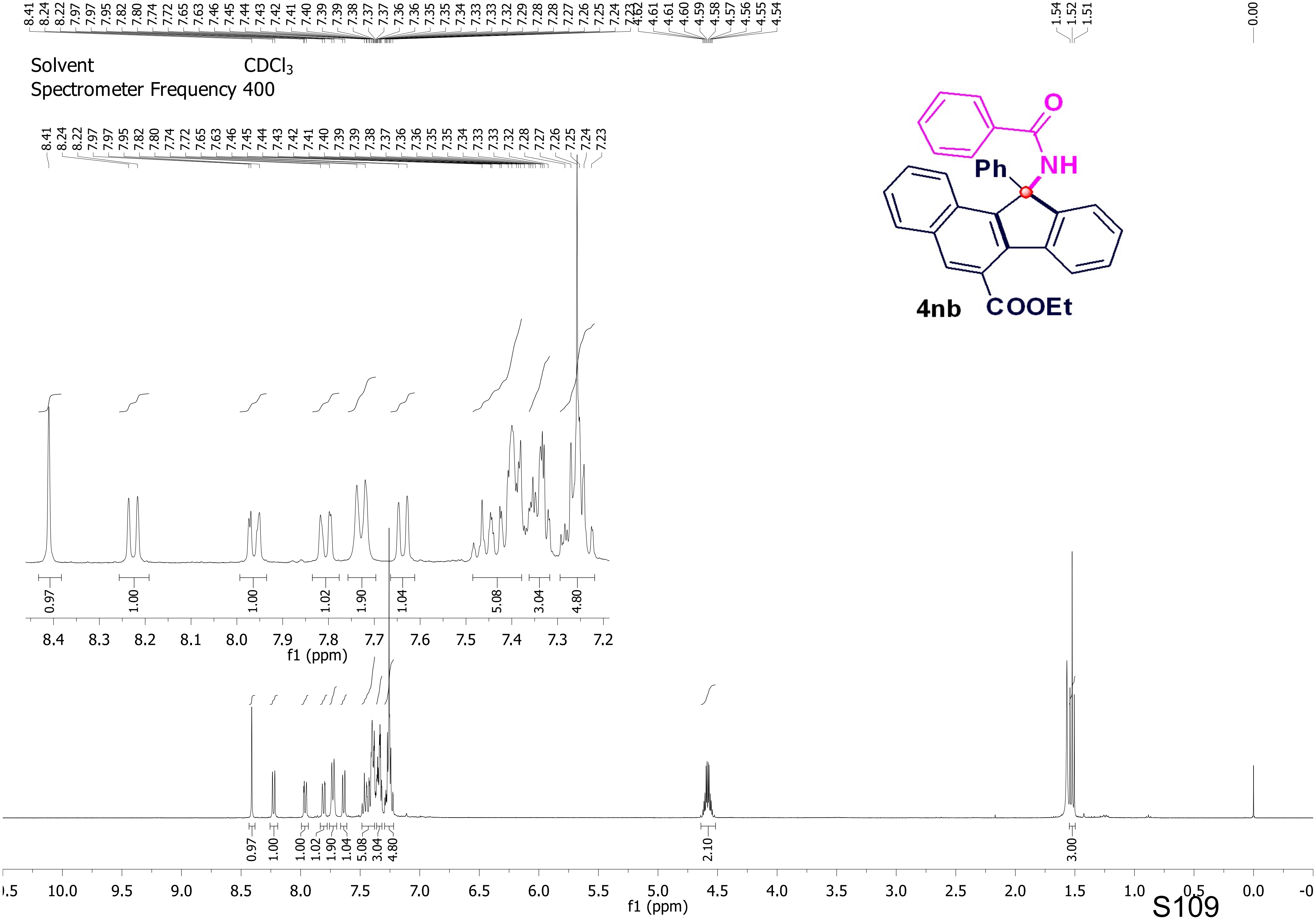
8.41 8.24 8.22 7.97 7.97 7.95 7.82 7.80 7.74 7.72 7.65 7.63 7.46 7.45 7.44 7.43 7.42 7.41 7.40 7.39 7.39 7.38 7.37 7.37 7.36 7.36 7.35 7.35 7.34 7.33 7.33 7.32 7.29 7.28 7.28 7.27 7.26 7.25 7.24 7.23 4.62 4.61 4.61 4.60 4.59 4.58 4.57 4.56 4.55 4.54
1.54 1.52 1.51
0.00
Solvent CDCl3
Spectrometer Frequency 400
O
NH
Ph
4nb
COOEt
0.97 1.00 1.00 1.02 1.90 1.04 5.08 3.04 4.80
f1 (ppm)
2.10
3.00
f1 (ppm)

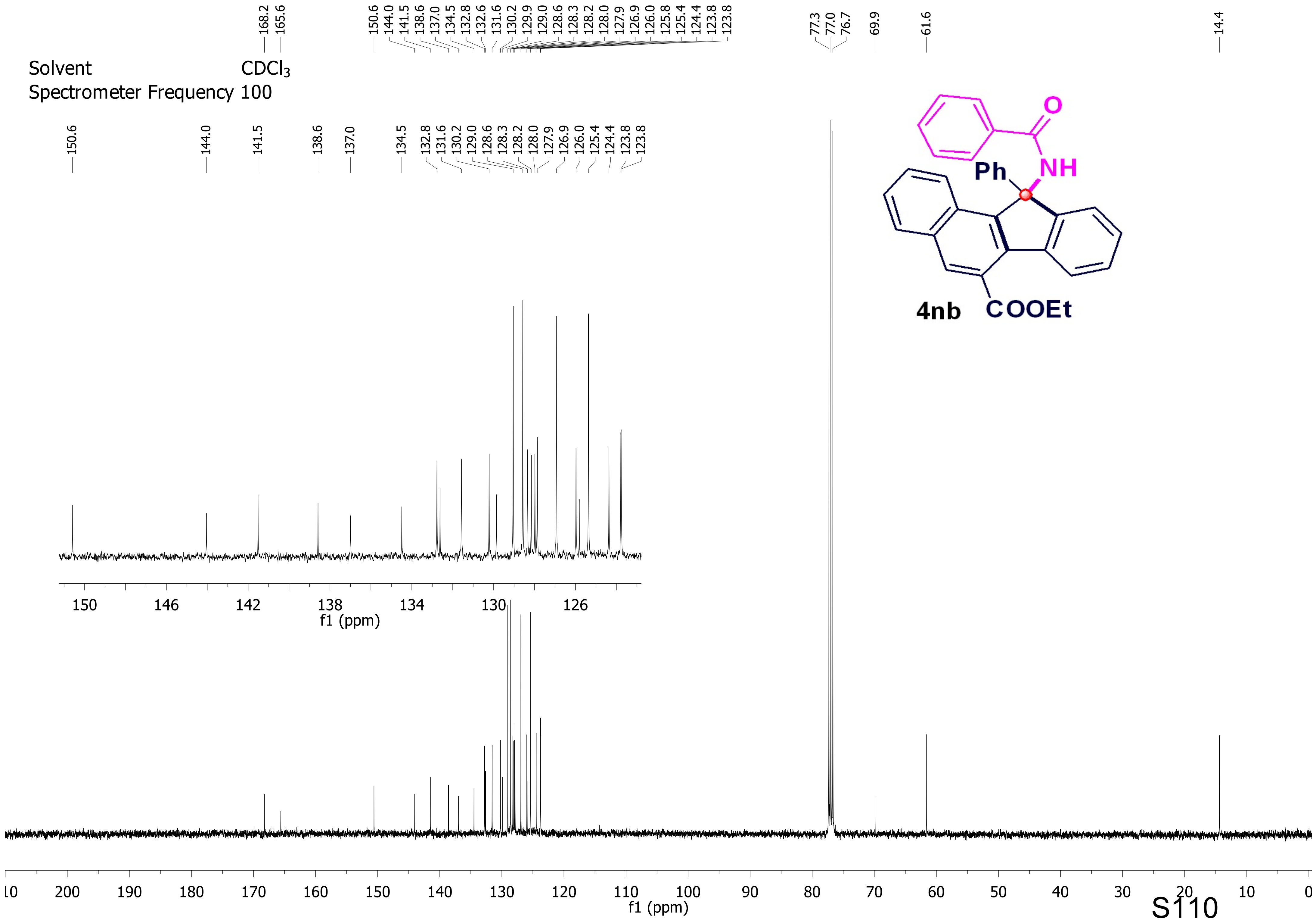
Solvent CDCl$_3$
Spectrometer Frequency 100
168.2
165.6
150.6
144.0
141.5
138.6
137.0
134.5
132.8
132.6
131.6
130.2
129.9
129.0
128.6
128.3
128.2
128.0
127.9
126.9
126.0
125.8
125.4
124.4
123.8
123.8
77.3
77.0
76.7
69.9
61.6
14.4
O
NH
Ph
4nb
COOEt
f1 (ppm)

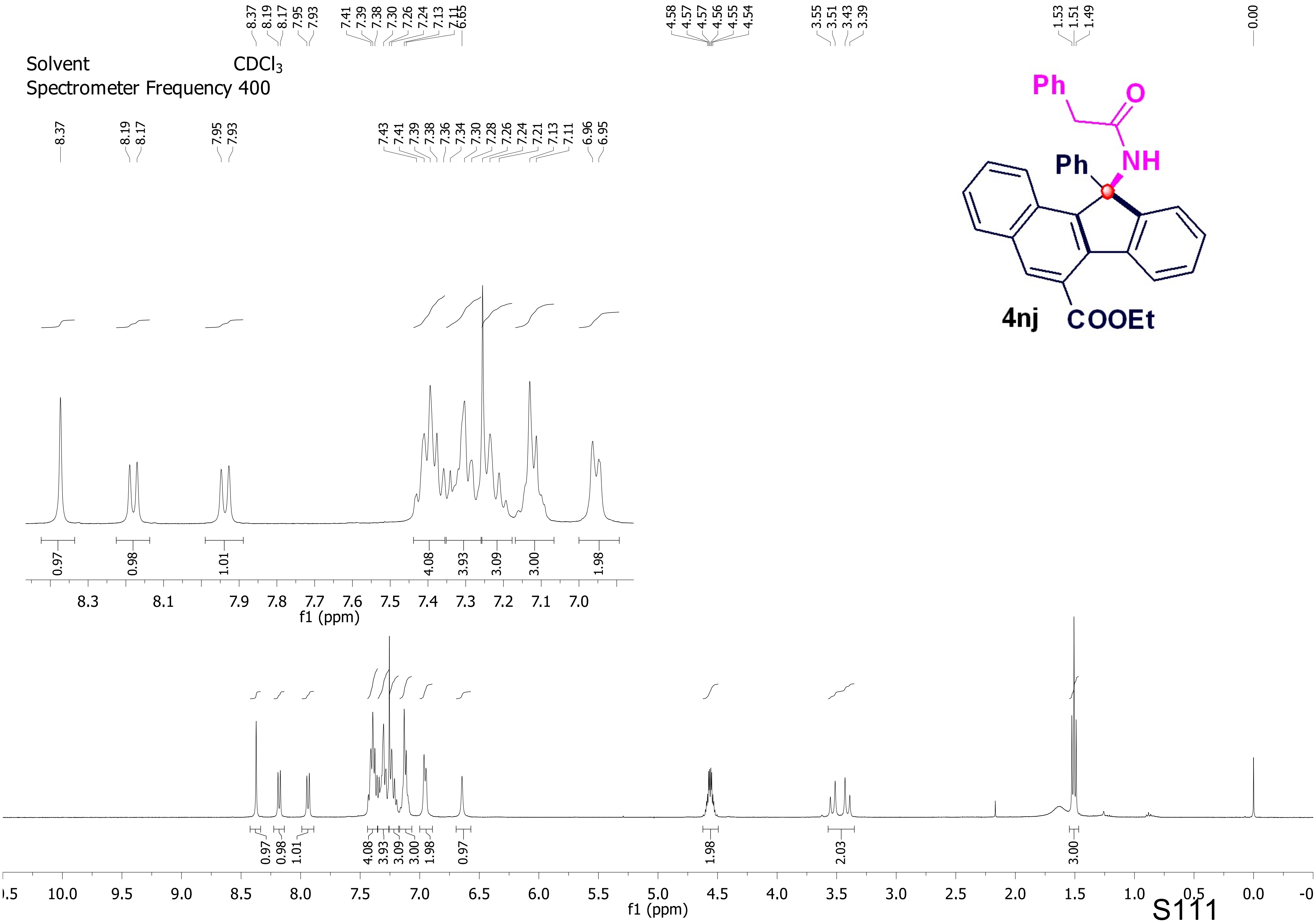
Solvent CDCl3
Spectrometer Frequency 400
8.37 8.19 8.17 7.95 7.93 7.41 7.39 7.38 7.30 7.26 7.24 7.13 7.11 6.65
4.58 4.57 4.57 4.56 4.55 4.54
3.55 3.51 3.43 3.39
1.53 1.51 1.49
0.00
8.37 8.19 8.17 7.95 7.93 7.43 7.41 7.39 7.38 7.36 7.34 7.30 7.28 7.26 7.24 7.21 7.13 7.11 6.96 6.95
0.97 0.98 1.01 4.08 3.93 3.09 3.00 1.98
f1 (ppm)
0.97 0.98 1.01 4.08 3.93 3.09 3.00 1.98 0.97 1.98 2.03 3.00
f1 (ppm)
Ph
O
NH
Ph
4nj
COOEt

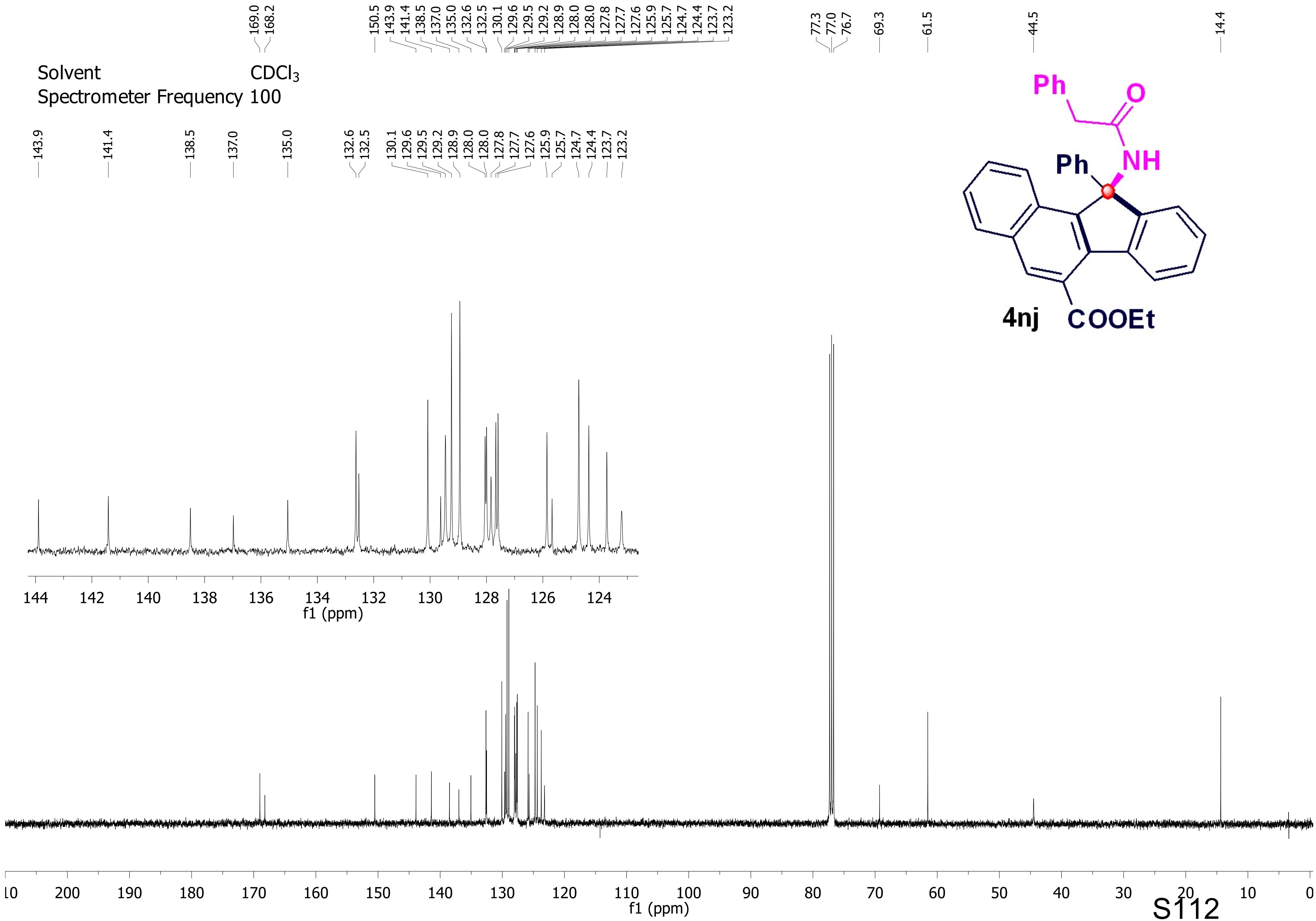
169.0
168.2
150.5
143.9
141.4
138.5
137.0
135.0
132.6
132.5
130.1
129.6
129.5
129.2
128.9
128.0
128.0
127.8
127.7
127.6
125.9
125.7
124.7
124.4
123.7
123.2
77.3
77.0
76.7
69.3
61.5
44.5
14.4
Solvent CDCl3
Spectrometer Frequency 100
Ph
O
NH
Ph
4nj
COOEt
f1 (ppm)
f1 (ppm)

Solvent CDCl$_3$
Spectrometer Frequency 400

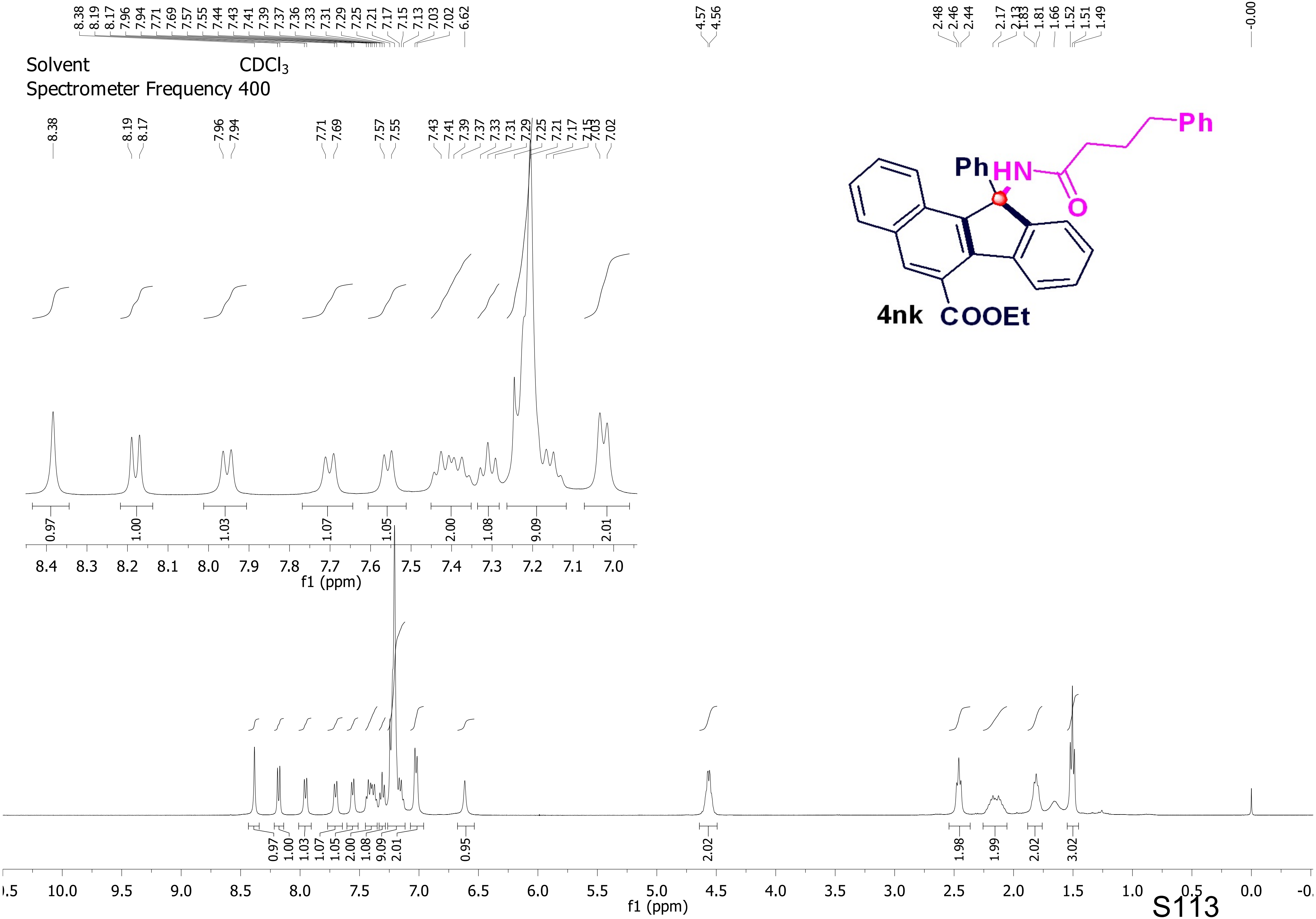

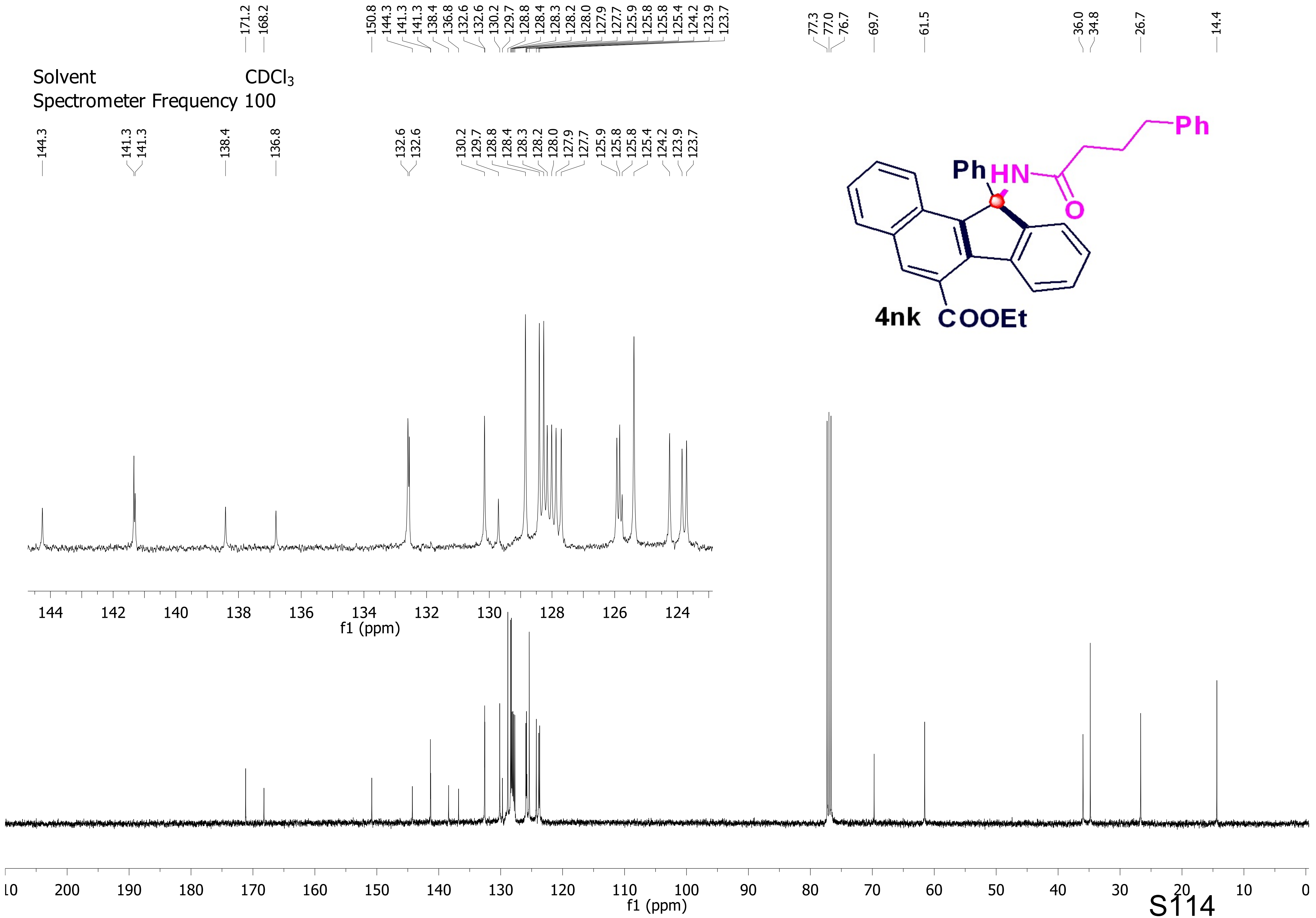
171.2
168.2
150.8
144.3
141.3
141.3
138.4
136.8
132.6
132.6
130.2
129.7
128.8
128.4
128.3
128.2
128.0
127.9
127.7
125.9
125.8
125.8
125.4
124.2
123.9
123.7
77.3
77.0
76.7
69.7
61.5
36.0
34.8
26.7
14.4
Solvent CDCl3
Spectrometer Frequency 100
Ph
HN
O
Ph
4nk
COOEt
f1 (ppm)
f1 (ppm)

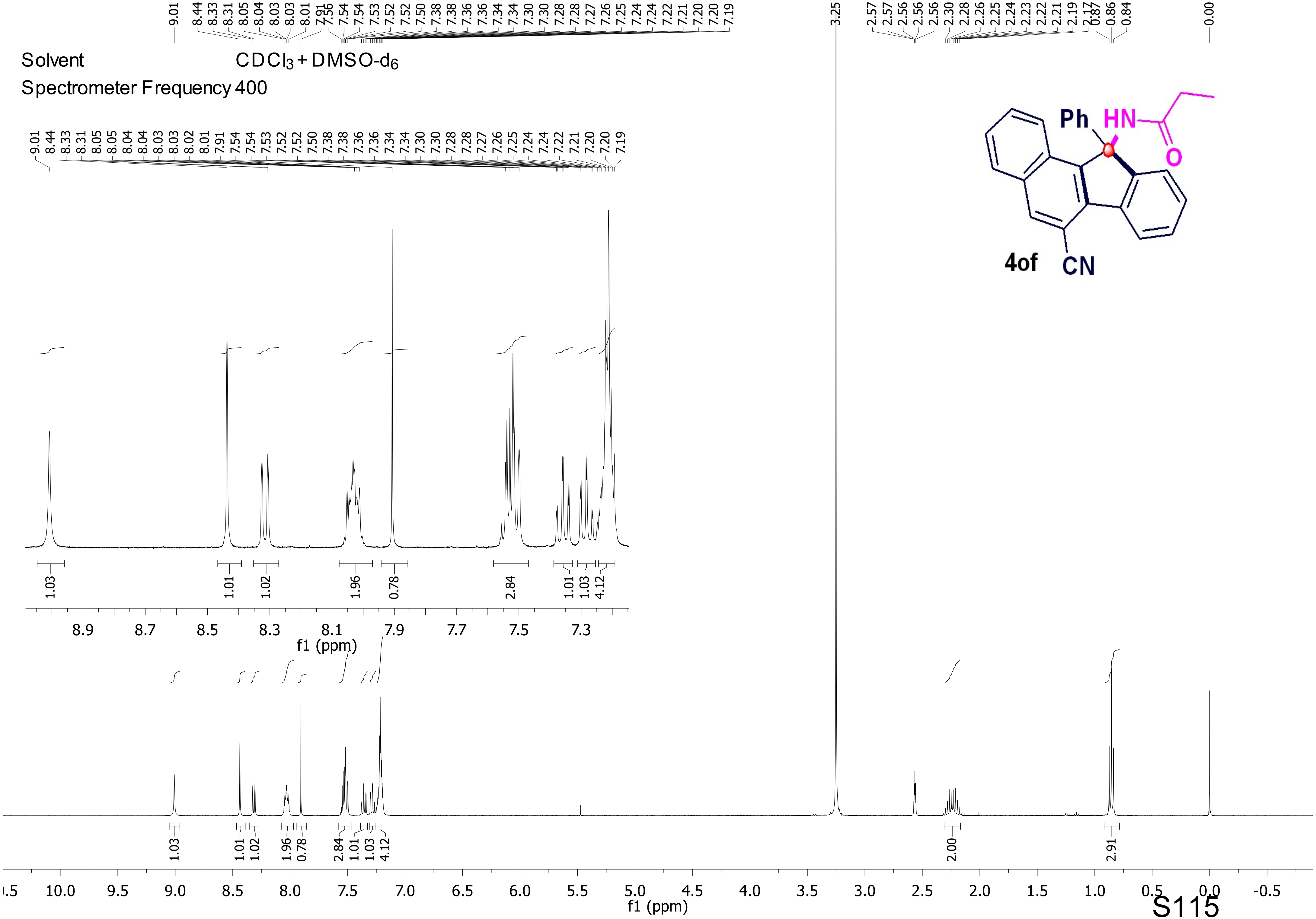
Solvent CDCl3 + DMSO-d6
Spectrometer Frequency 400
Ph
HN
O
CN
4of
f1 (ppm)

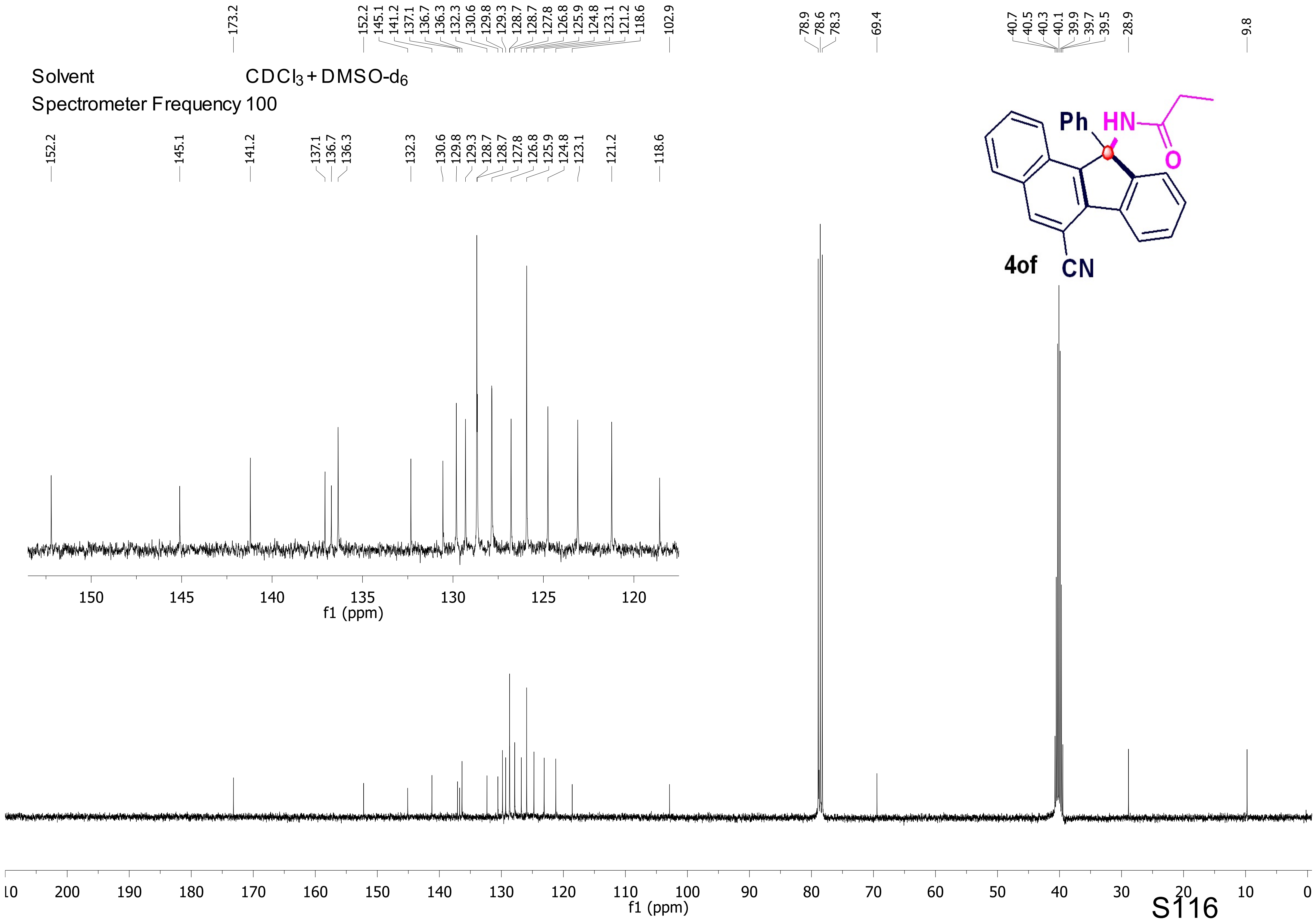
173.2
152.2 145.1 141.2 137.1 136.7 136.3 132.3 130.6 129.8 129.3 128.7 128.7 127.8 126.8 125.9 124.8 123.1 121.2 118.6
102.9
78.9 78.6 78.3
69.4
40.7 40.5 40.3 40.1 39.9 39.7 39.5
28.9
9.8
Solvent CDCl3 + DMSO-d6
Spectrometer Frequency 100
152.2 145.1 141.2 137.1 136.7 136.3 132.3 130.6 129.8 129.3 128.7 128.7 127.8 126.8 125.9 124.8 123.1 121.2 118.6
Ph
HN
O
CN
4of
f1 (ppm)
f1 (ppm)

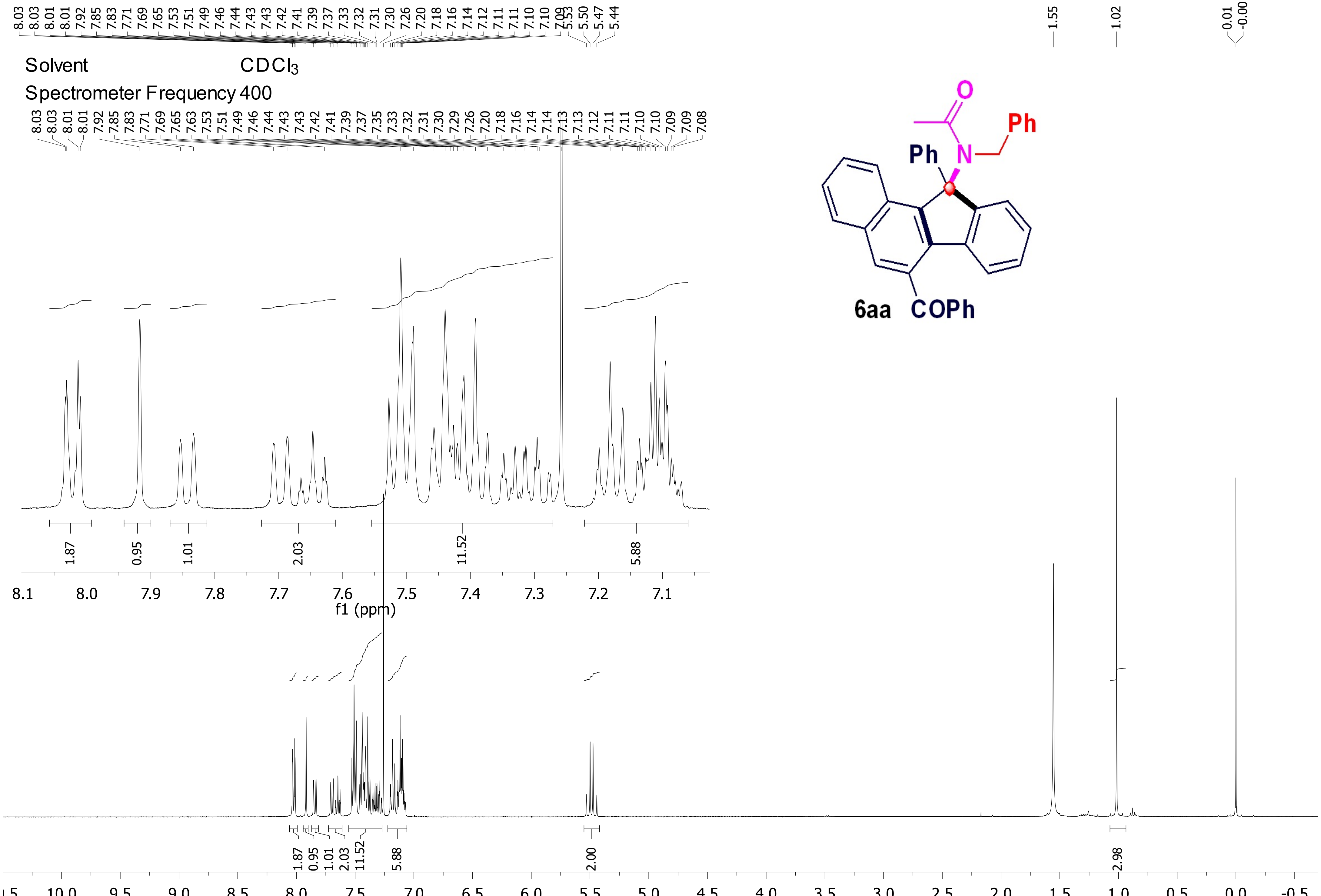
8.03 8.03 8.01 8.01 7.92 7.85 7.83 7.71 7.69 7.65 7.53 7.51 7.49 7.46 7.44 7.43 7.43 7.42 7.41 7.39 7.37 7.33 7.32 7.31 7.30 7.26 7.20 7.18 7.16 7.14 7.12 7.11 7.11 7.10 7.10 7.09 5.53 5.50 5.47 5.44
1.55
1.02
0.01 -0.00
Solvent CDCl3
Spectrometer Frequency 400
8.03 8.03 8.01 8.01 7.92 7.85 7.83 7.71 7.69 7.65 7.63 7.53 7.51 7.49 7.46 7.44 7.43 7.43 7.42 7.41 7.39 7.37 7.35 7.33 7.32 7.31 7.30 7.29 7.26 7.20 7.18 7.16 7.14 7.14 7.13 7.13 7.12 7.11 7.11 7.10 7.10 7.09 7.09 7.08
O
Ph
Ph
N
6aa
COPh
1.87
0.95
1.01
2.03
11.52
5.88
f1 (ppm)
2.00
2.98
f1 (ppm)

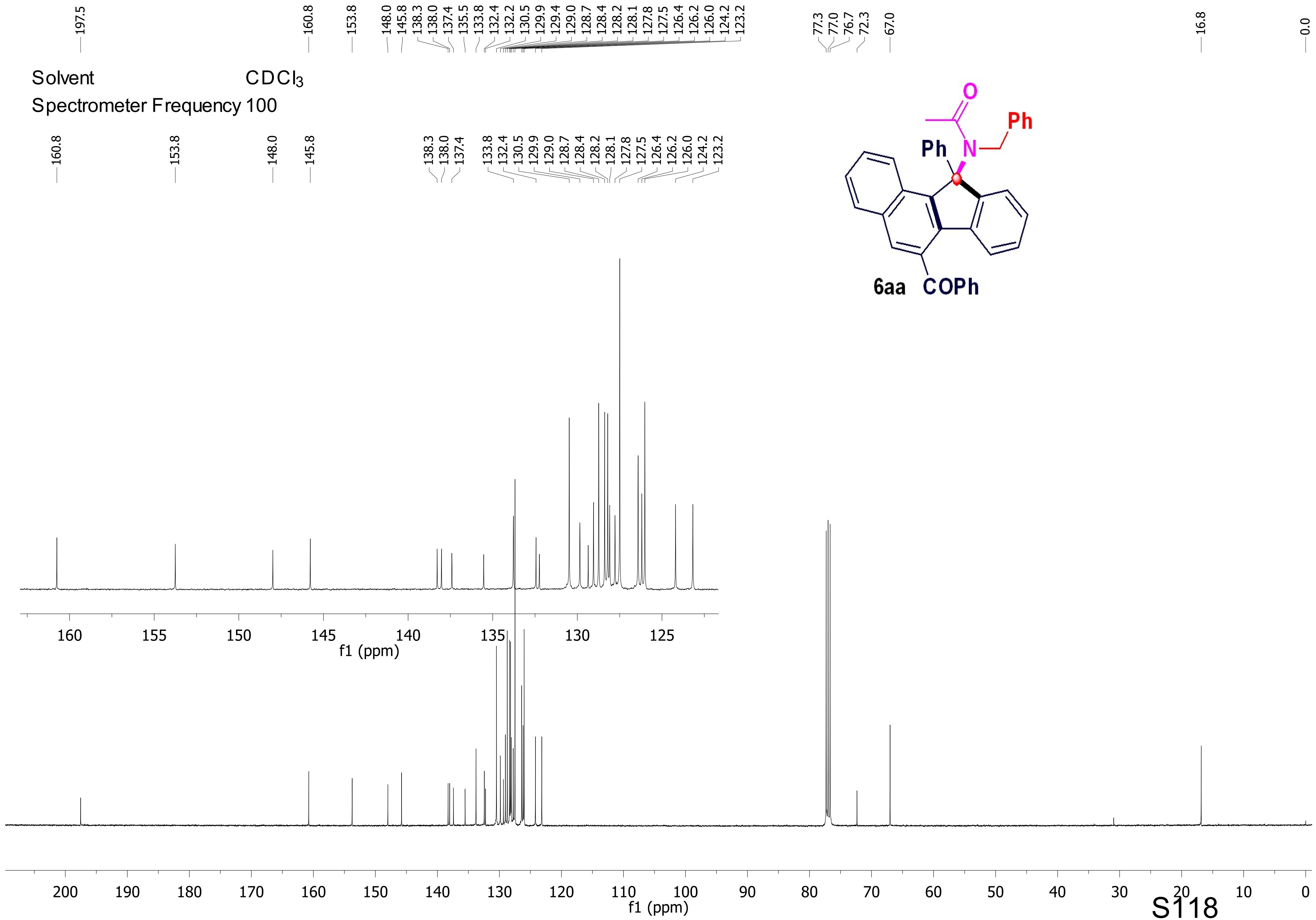
197.5
160.8
153.8
148.0
145.8
138.3
138.0
137.4
135.5
133.8
132.4
132.2
130.5
129.9
129.4
129.0
128.7
128.4
128.2
128.1
127.8
127.5
126.4
126.2
126.0
124.2
123.2
77.3
77.0
76.7
72.3
67.0
16.8
0.0
Solvent CDCl3
Spectrometer Frequency 100
O
Ph
Ph
N
6aa
COPh
f1 (ppm)
f1 (ppm)

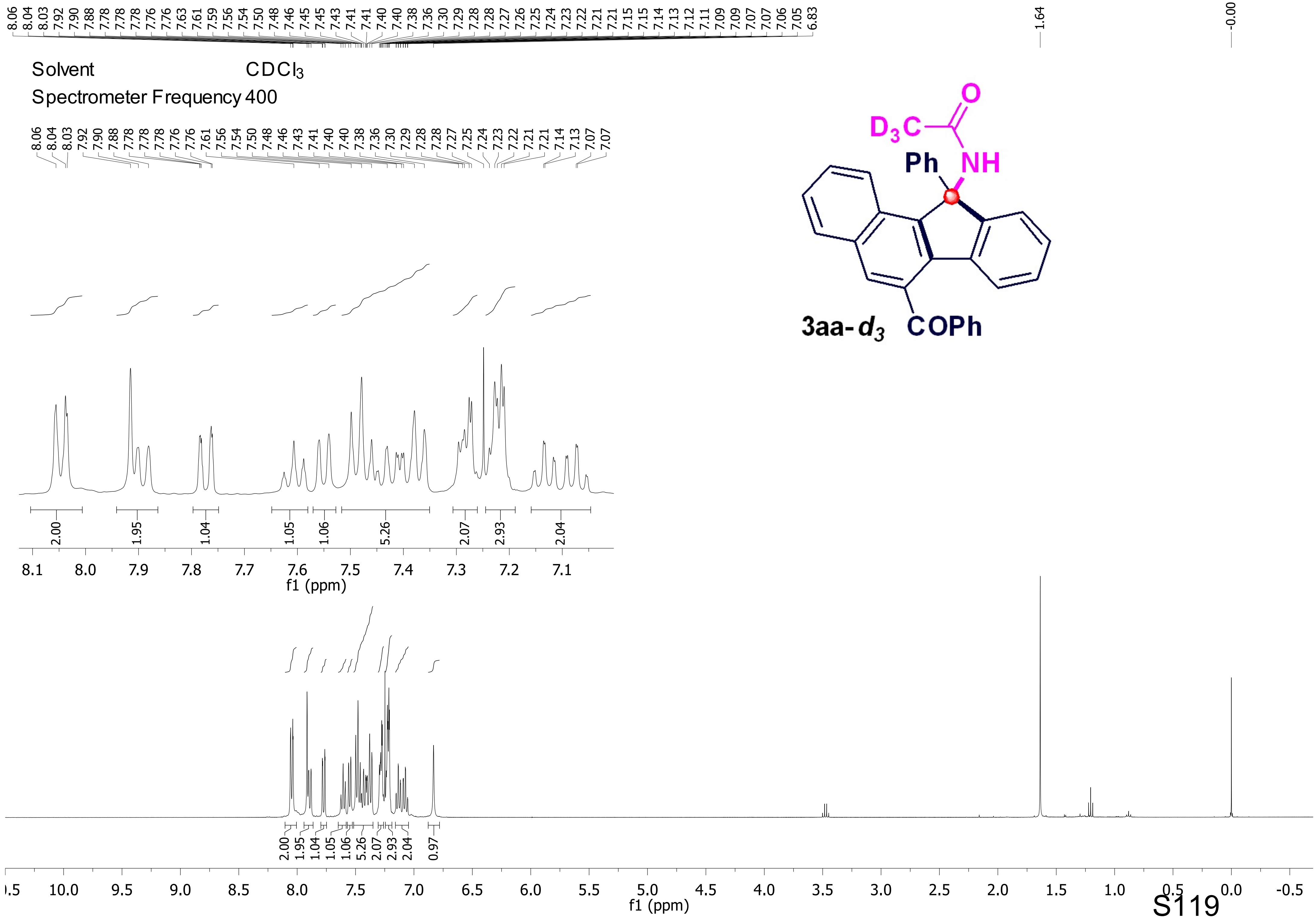
8.06 8.04 8.03 7.92 7.90 7.88 7.78 7.78 7.78 7.76 7.76 7.63 7.61 7.59 7.56 7.54 7.50 7.48 7.46 7.45 7.45 7.43 7.41 7.41 7.40 7.40 7.38 7.36 7.30 7.29 7.28 7.28 7.27 7.26 7.25 7.24 7.23 7.22 7.21 7.21 7.15 7.15 7.14 7.13 7.12 7.11 7.09 7.09 7.07 7.07 7.06 7.05 6.83
1.64
-0.00
Solvent CDCl3
Spectrometer Frequency 400
D3C
O
NH
Ph
3aa-d3
COPh
2.00 1.95 1.04 1.05 1.06 5.26 2.07 2.93 2.04
2.00 1.95 1.04 1.05 1.06 5.26 2.07 2.93 2.04 0.97
f1 (ppm)
f1 (ppm)

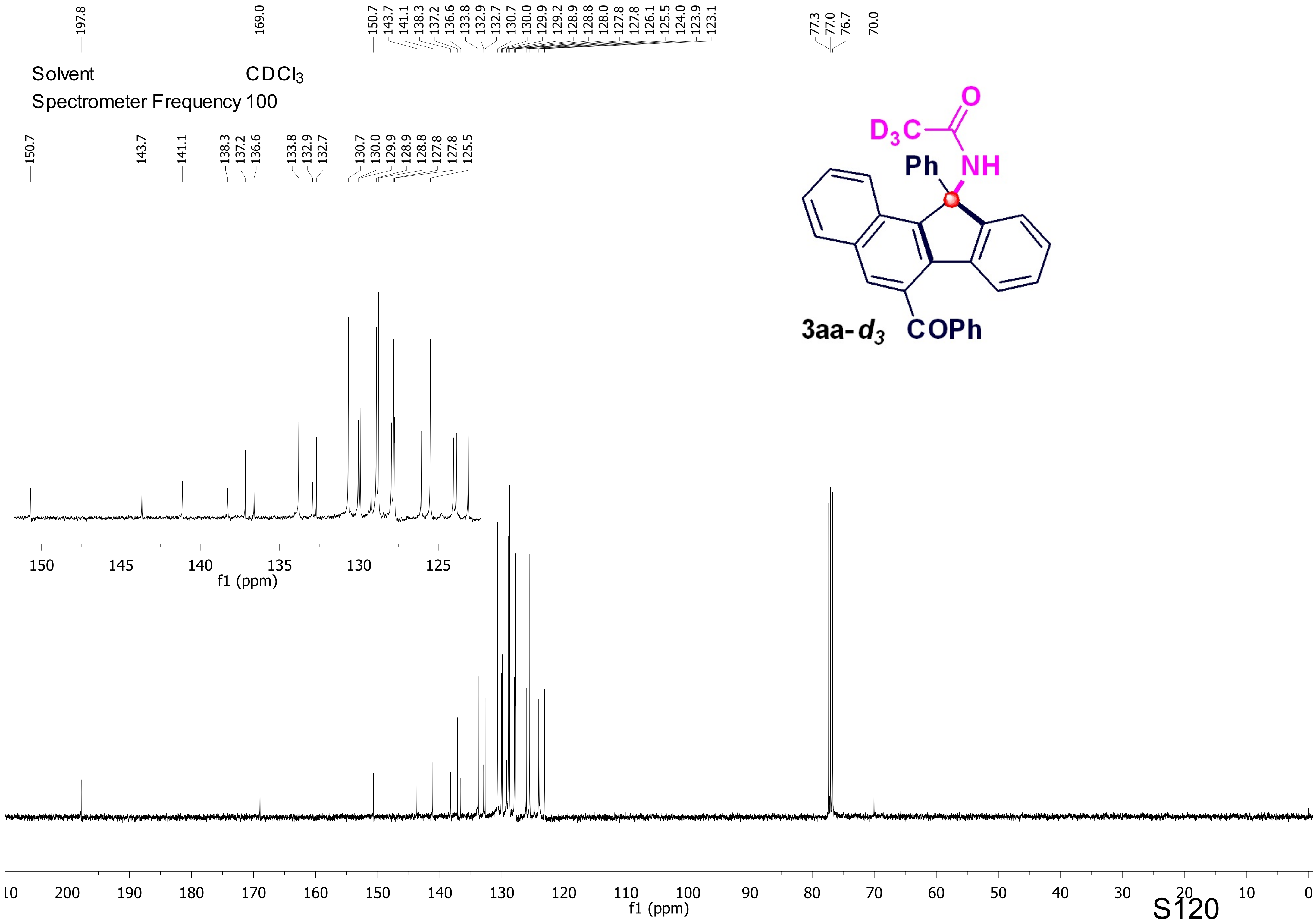
197.8
169.0
150.7
143.7
141.1
138.3
137.2
136.6
133.8
132.9
132.7
130.7
130.0
129.9
129.2
128.9
128.8
128.0
127.8
127.8
126.1
125.5
124.0
123.9
123.1
77.3
77.0
76.7
70.0
Solvent CDCl3
Spectrometer Frequency 100
150.7
143.7
141.1
138.3
137.2
136.6
133.8
132.9
132.7
130.7
130.0
129.9
128.9
128.8
127.8
127.8
125.5
f1 (ppm)
D3C
O
NH
Ph
3aa-d3
COPh
f1 (ppm)

checkCIF/PLATON report

You have not supplied any structure factors. As a result the full set of tests cannot be run.

THIS REPORT IS FOR GUIDANCE ONLY. IF USED AS PART OF A REVIEW PROCEDURE FOR PUBLICATION, IT SHOULD NOT REPLACE THE EXPERTISE OF AN EXPERIENCED CRYSTALLOGRAPHIC REFEREE.

No syntax errors found.   CIF dictionary   Interpreting this report

Datablock: I

Bond precision:	C-C = 0.0023 A		Wavelength=0.71073
Cell:	a=12.2731(3)	b=11.6422(3)	c=17.3487(4)
	alpha=90	beta=92.982(2)	gamma=90
Temperature:	113 K		

	Calculated	Reported
Volume	2475.53(10)	2475.53(10)
Space group	P 21/c	P 1 21/c 1
Hall group	-P 2ybc	-P 2ybc
Moiety formula	C33 H25 N O3	C33 H25 N O3
Sum formula	C33 H25 N O3	C33 H25 N O3
Mr	483.54	483.54
Dx,g cm-3	1.297	1.297
Z	4	4
Mu (mm-1)	0.083	0.083
F000	1016.0	1016.0
F000'	1016.45	
h,k,lmax	14,13,20	14,13,20
Nref	4362	4355
Tmin,Tmax	0.979,0.984	0.710,1.000
Tmin'	0.979	

Correction method= # Reported T Limits: Tmin=0.710 Tmax=1.000
AbsCorr = MULTI-SCAN

Data completeness= 0.998   Theta(max)= 24.998

R(reflections)= 0.0439(3136)   wR2(reflections)= 0.1216(4355)

S = 1.086   Npar= 335

The following ALERTS were generated. Each ALERT has the format
test-name_ALERT_alert-type_alert-level.
Click on the hyperlinks for more details of the test.

Alert level C

```
PLAT761_ALERT_1_C CIF Contains no X-H Bonds ......................     Please Check
PLAT762_ALERT_1_C CIF Contains no X-Y-H or H-Y-H Angles ..........     Please Check
```

Alert level G

```
PLAT003_ALERT_2_G Number of Uiso or Uij Restrained non-H Atoms ...         37 Report
PLAT005_ALERT_5_G No Embedded Refinement Details Found  in the CIF     Please Do !
PLAT007_ALERT_5_G Number of Unrefined Donor-H Atoms ..............          1 Report
PLAT793_ALERT_4_G Model has Chirality at C8          (Centro SPGR)          S Verify
PLAT860_ALERT_3_G Number of Least-Squares Restraints .............       1098 Note
```

0 **ALERT level A** = Most likely a serious problem - resolve or explain
0 **ALERT level B** = A potentially serious problem, consider carefully
2 **ALERT level C** = Check. Ensure it is not caused by an omission or oversight
5 **ALERT level G** = General information/check it is not something unexpected

2 ALERT type 1 CIF construction/syntax error, inconsistent or missing data
1 ALERT type 2 Indicator that the structure model may be wrong or deficient
1 ALERT type 3 Indicator that the structure quality may be low
1 ALERT type 4 Improvement, methodology, query or suggestion
2 ALERT type 5 Informative message, check

checkCIF publication errors

Alert level A

```
PUBL004_ALERT_1_A  The contact author's name and address are missing,
            _publ_contact_author_name and _publ_contact_author_address.
PUBL005_ALERT_1_A  _publ_contact_author_email, _publ_contact_author_fax and
            _publ_contact_author_phone are all missing.
            At least one of these should be present.
PUBL006_ALERT_1_A  _publ_requested_journal is missing
            e.g. 'Acta Crystallographica Section C'
PUBL008_ALERT_1_A  _publ_section_title is missing. Title of paper.
PUBL009_ALERT_1_A  _publ_author_name is missing. List of author(s) name(s).
PUBL010_ALERT_1_A  _publ_author_address is missing. Author(s) address(es).
PUBL012_ALERT_1_A  _publ_section_abstract is missing.
            Abstract of paper in English.
```

7 **ALERT level A** = Data missing that is essential or data in wrong format
0 **ALERT level G** = General alerts. Data that may be required is missing

Publication of your CIF

You should attempt to resolve as many as possible of the alerts in all categories. Often the minor alerts point to easily fixed oversights, errors and omissions in your CIF or refinement strategy, so attention to these fine details can be worthwhile. In order to resolve some of the more serious problems it may be necessary to carry out additional measurements or structure refinements. However, the nature of your study may justify the reported deviations from journal submission requirements and the more serious of these should be commented upon in the discussion or experimental section of a paper or in the "special_details" fields of the CIF. *checkCIF* was carefully designed to identify outliers and unusual parameters, but every test has its limitations and alerts that are not important in a particular case may appear. Conversely, the absence of alerts does not guarantee there are no aspects of the results needing attention. It is up to the individual to critically assess their own results and, if necessary, seek expert advice.

If level A alerts remain, which you believe to be justified deviations, and you intend to submit this CIF for publication in a journal, you should additionally insert an explanation in your CIF using the Validation Reply Form (VRF) below. This will allow your explanation to be considered as part of the review process.

Validation response form

Please find below a validation response form (VRF) that can be filled in and pasted into your CIF.

```
# start Validation Reply Form
_vrf_PUBL004_GLOBAL
;
PROBLEM: The contact author’s name and address are missing,
RESPONSE: ...
;
_vrf_PUBL005_GLOBAL
;
PROBLEM: _publ_contact_author_email, _publ_contact_author_fax and
RESPONSE: ...
;
_vrf_PUBL006_GLOBAL
;
PROBLEM: _publ_requested_journal is missing
RESPONSE: ...
;
_vrf_PUBL008_GLOBAL
;
PROBLEM: _publ_section_title is missing. Title of paper.
RESPONSE: ...
;
_vrf_PUBL009_GLOBAL
;
PROBLEM: _publ_author_name is missing. List of author(s) name(s).
RESPONSE: ...
;
_vrf_PUBL010_GLOBAL
;
PROBLEM: _publ_author_address is missing. Author(s) address(es).
```

```
RESPONSE: ...
;
_vrf_PUBL012_GLOBAL
;
PROBLEM: _publ_section_abstract is missing.
RESPONSE: ...
;
# end Validation Reply Form
```

If you wish to submit your CIF for publication in Acta Crystallographica Section C or E, you should upload your CIF via the web. If you wish to submit your CIF for publication in IUCrData you should upload your CIF via the web. If your CIF is to form part of a submission to another IUCr journal, you will be asked, either during electronic submission or by the Co-editor handling your paper, to upload your CIF via our web site.

PLATON version of 13/07/2021; check.def file version of 13/07/2021

Datablock I - ellipsoid plot

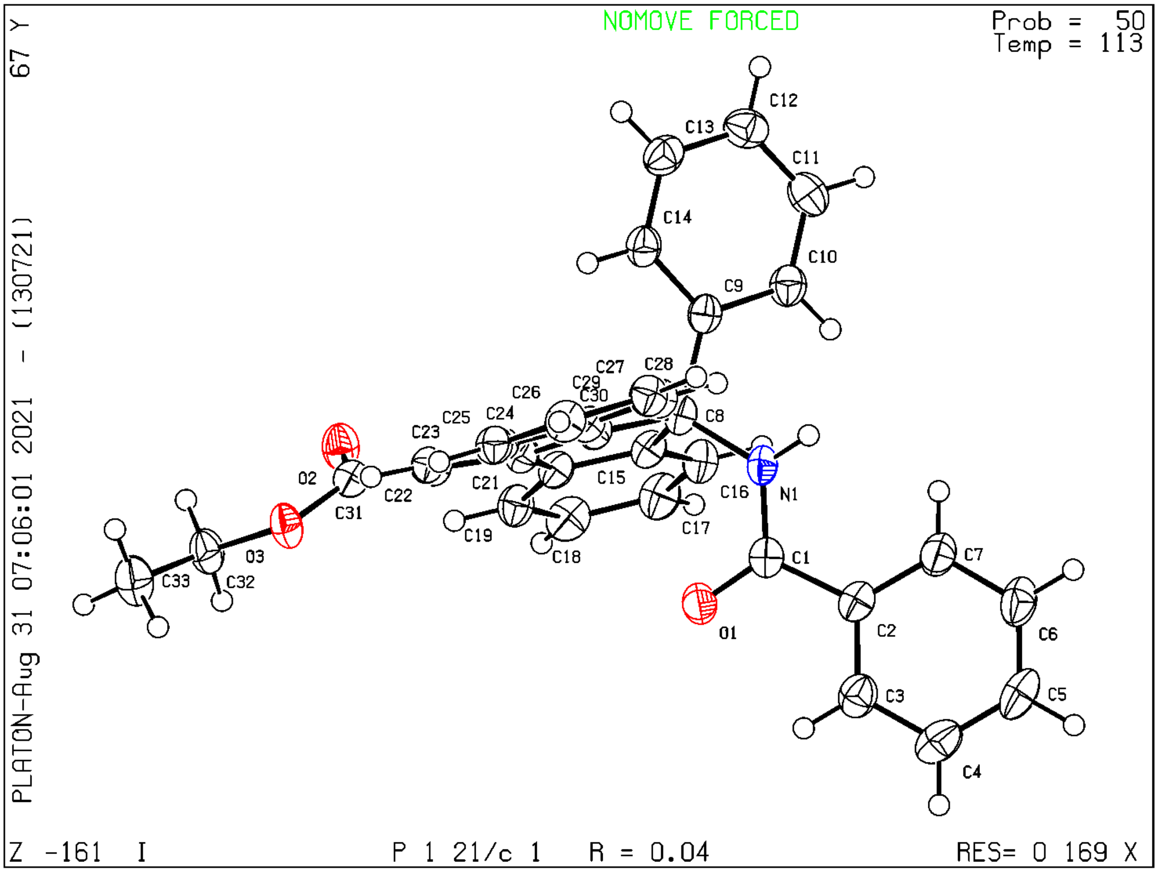

checkCIF/PLATON report

Structure factors have been supplied for datablock(s) I

THIS REPORT IS FOR GUIDANCE ONLY. IF USED AS PART OF A REVIEW PROCEDURE FOR PUBLICATION, IT SHOULD NOT REPLACE THE EXPERTISE OF AN EXPERIENCED CRYSTALLOGRAPHIC REFEREE.

No syntax errors found. CIF dictionary Interpreting this report

Datablock: I

Bond precision: C-C = 0.0018 A Wavelength=0.71073

Cell:	a=8.7837(2)	b=22.3485(3)	c=10.1446(2)
	alpha=90	beta=95.730(2)	gamma=90
Temperature:	113 K		

	Calculated	Reported
Volume	1981.46(7)	1981.46(7)
Space group	P 21/c	P 1 21/c 1
Hall group	-P 2ybc	-P 2ybc
Moiety formula	C27 H20 N2 O	C27 H20 N2 O
Sum formula	C27 H20 N2 O	C27 H20 N2 O
Mr	388.45	388.45
Dx,g cm-3	1.302	1.302
Z	4	4
Mu (mm-1)	0.080	0.080
F000	816.0	816.0
F000'	816.31	
h,k,lmax	10,26,12	10,26,12
Nref	3489	3489
Tmin,Tmax	0.976,0.984	0.501,1.000
Tmin'	0.976	

Correction method= # Reported T Limits: Tmin=0.501 Tmax=1.000
AbsCorr = MULTI-SCAN

Data completeness= 1.000 Theta(max)= 24.996

R(reflections)= 0.0348(3192) wR2(reflections)= 0.0894(3489)

S = 1.056 Npar= 272

The following ALERTS were generated. Each ALERT has the format
test-name_ALERT_alert-type_alert-level.
Click on the hyperlinks for more details of the test.

Alert level G

```
PLAT003_ALERT_2_G Number of Uiso or Uij Restrained non-H Atoms ...         30 Report
PLAT007_ALERT_5_G Number of Unrefined Donor-H Atoms ..............          1 Report
PLAT178_ALERT_4_G The CIF-Embedded .res File Contains SIMU Records          1 Report
PLAT793_ALERT_4_G Model has Chirality at C1          (Centro SPGR)          S Verify
PLAT860_ALERT_3_G Number of Least-Squares Restraints .............        924 Note
PLAT909_ALERT_3_G Percentage of I>2sig(I) Data at Theta(Max) Still        83% Note
PLAT978_ALERT_2_G Number C-C Bonds with Positive Residual Density.         12 Info
```

---

0 **ALERT level A** = Most likely a serious problem - resolve or explain
0 **ALERT level B** = A potentially serious problem, consider carefully
0 **ALERT level C** = Check. Ensure it is not caused by an omission or oversight
7 **ALERT level G** = General information/check it is not something unexpected

0 ALERT type 1 CIF construction/syntax error, inconsistent or missing data
2 ALERT type 2 Indicator that the structure model may be wrong or deficient
2 ALERT type 3 Indicator that the structure quality may be low
2 ALERT type 4 Improvement, methodology, query or suggestion
1 ALERT type 5 Informative message, check

---

checkCIF publication errors

Alert level A

```
PUBL004_ALERT_1_A  The contact author's name and address are missing,
            _publ_contact_author_name and _publ_contact_author_address.
PUBL005_ALERT_1_A  _publ_contact_author_email, _publ_contact_author_fax and
            _publ_contact_author_phone are all missing.
            At least one of these should be present.
PUBL006_ALERT_1_A  _publ_requested_journal is missing
            e.g. 'Acta Crystallographica Section C'
PUBL008_ALERT_1_A  _publ_section_title is missing. Title of paper.
PUBL009_ALERT_1_A  _publ_author_name is missing. List of author(s) name(s).
PUBL010_ALERT_1_A  _publ_author_address is missing. Author(s) address(es).
PUBL012_ALERT_1_A  _publ_section_abstract is missing.
            Abstract of paper in English.
```

---

7 **ALERT level A** = Data missing that is essential or data in wrong format
0 **ALERT level G** = General alerts. Data that may be required is missing

Publication of your CIF

You should attempt to resolve as many as possible of the alerts in all categories. Often the minor alerts point to easily fixed oversights, errors and omissions in your CIF or refinement strategy, so attention to these fine details can be worthwhile. In order to resolve some of the more serious problems it may be necessary to carry out additional measurements or structure refinements. However, the nature of your study may justify the reported deviations from journal submission requirements and the more serious of these should be commented upon in the discussion or experimental section of a paper or in the "special_details" fields of the CIF. *checkCIF* was carefully designed to identify outliers and unusual parameters, but every test has its limitations and alerts that are not important in a particular case may appear. Conversely, the absence of alerts does not guarantee there are no aspects of the results needing attention. It is up to the individual to critically assess their own results and, if necessary, seek expert advice.

If level A alerts remain, which you believe to be justified deviations, and you intend to submit this CIF for publication in a journal, you should additionally insert an explanation in your CIF using the Validation Reply Form (VRF) below. This will allow your explanation to be considered as part of the review process.

Validation response form

Please find below a validation response form (VRF) that can be filled in and pasted into your CIF.

```
# start Validation Reply Form
_vrf_PUBL004_GLOBAL
;
PROBLEM: The contact author’s name and address are missing,
RESPONSE: ...
;
_vrf_PUBL005_GLOBAL
;
PROBLEM: _publ_contact_author_email, _publ_contact_author_fax and
RESPONSE: ...
;
_vrf_PUBL006_GLOBAL
;
PROBLEM: _publ_requested_journal is missing
RESPONSE: ...
;
_vrf_PUBL008_GLOBAL
;
PROBLEM: _publ_section_title is missing. Title of paper.
RESPONSE: ...
;
_vrf_PUBL009_GLOBAL
;
PROBLEM: _publ_author_name is missing. List of author(s) name(s).
RESPONSE: ...
;
_vrf_PUBL010_GLOBAL
;
PROBLEM: _publ_author_address is missing. Author(s) address(es).
RESPONSE: ...
;
_vrf_PUBL012_GLOBAL
;
```

```
PROBLEM: _publ_section_abstract is missing.
RESPONSE: ...
;
# end Validation Reply Form
```

If you wish to submit your CIF for publication in Acta Crystallographica Section C or E, you should upload your CIF via the web. If you wish to submit your CIF for publication in IUCrData you should upload your CIF via the web. If your CIF is to form part of a submission to another IUCr journal, you will be asked, either during electronic submission or by the Co-editor handling your paper, to upload your CIF via our web site.

PLATON version of 13/07/2021; check.def file version of 13/07/2021

Datablock I - ellipsoid plot

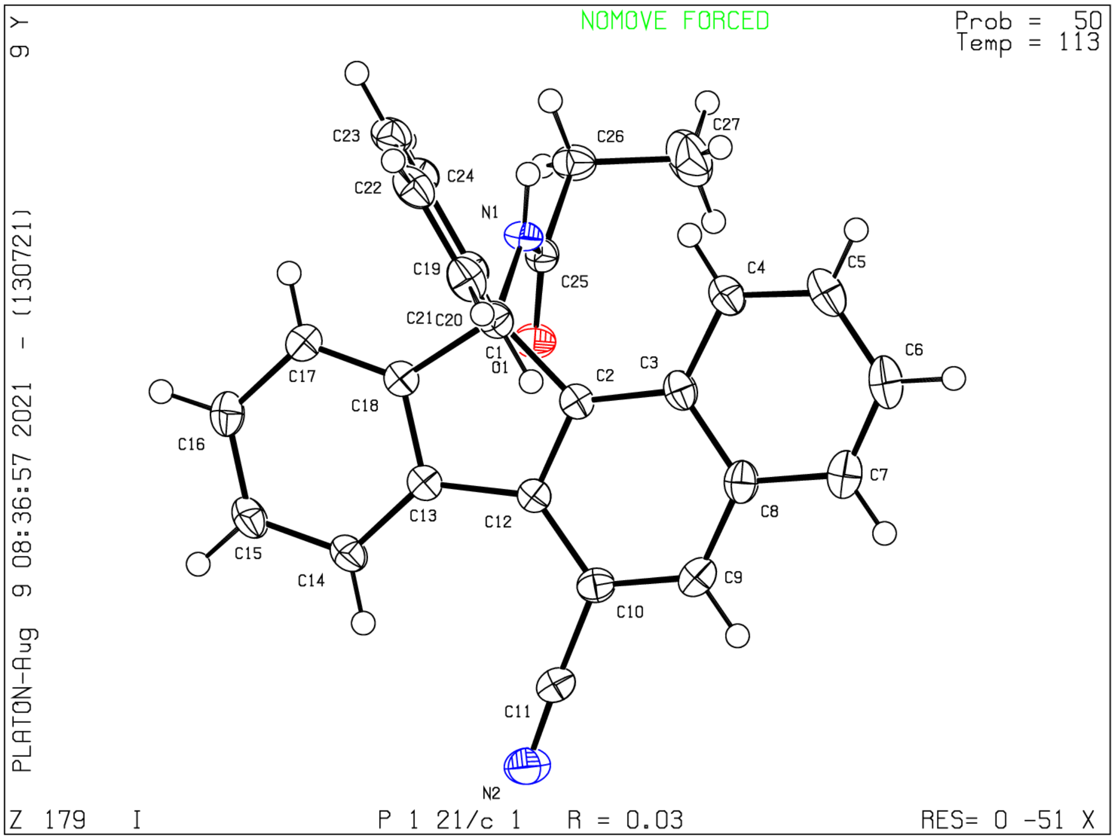

Supplement: Supplementary file 1 [file jo5c02263_si_001.pdf]
